# Medications that reduce emergency hospital admissions: an overview of systematic reviews and prioritisation of treatments

**DOI:** 10.1186/s12916-018-1104-9

**Published:** 2018-07-26

**Authors:** Niklas Bobrovitz, Carl Heneghan, Igho Onakpoya, Benjamin Fletcher, Dylan Collins, Alice Tompson, Joseph Lee, David Nunan, Rebecca Fisher, Brittney Scott, Jack O’Sullivan, Oliver Van Hecke, Brian D. Nicholson, Sarah Stevens, Nia Roberts, Kamal R. Mahtani

**Affiliations:** 1 Nuffield Department of Primary Care Health Sciences, University of Oxford, Radcliffe Observatory Quarter, Woodstock Road, Oxford, OX2 6GG United Kingdom; 2 Centre for Evidence-Based Medicine, University of Oxford, Oxford, United Kingdom; 3 Faculty of Medicine, University of British Columbia, Vancouver, Canada; 4 Faculty of Public Health and Policy, London School of Hygiene and Tropical Medicine, London, United Kingdom; 5 The Health Foundation, London, United Kingdom; 6 Department of Critical Care Medicine, University of Calgary, Calgary, Canada; 7 Snyder Institute for Chronic Diseases, University of Calgary, Calgary, Canada; 8 Bodelian Libraries, University of Oxford, Oxford, UK

**Keywords:** Hospital admissions, Unplanned admissions, Emergency admissions, Unscheduled admissions, Pharmacology, Medication, Drug, Systematic review, Overview, Clinical guidelines

## Abstract

**Background:**

Rates of emergency hospitalisations are increasing in many countries, leading to disruption in the quality of care and increases in cost. Therefore, identifying strategies to reduce emergency admission rates is a key priority. There have been large-scale evidence reviews to address this issue; however, there have been no reviews of medication therapies, which have the potential to reduce the use of emergency health-care services. The objectives of this study were to review systematically the evidence to identify medications that affect emergency hospital admissions and prioritise therapies for quality measurement and improvement.

**Methods:**

This was a systematic review of systematic reviews. We searched MEDLINE, PubMed, the Cochrane Database of Systematic Reviews & Database of Abstracts of Reviews of Effects, Google Scholar and the websites of ten major funding agencies and health charities, using broad search criteria. We included systematic reviews of randomised controlled trials that examined the effect of any medication on emergency hospital admissions among adults. We assessed the quality of reviews using AMSTAR. To prioritise therapies, we assessed the quality of trial evidence underpinning meta-analysed effect estimates and cross-referenced the evidence with clinical guidelines.

**Results:**

We identified 140 systematic reviews, which included 1968 unique randomised controlled trials and 925,364 patients. Reviews contained 100 medications tested in 47 populations. We identified high-to moderate-quality evidence for 28 medications that reduced admissions. Of these medications, 11 were supported by clinical guidelines in the United States, the United Kingdom and Europe. These 11 therapies were for patients with heart failure (angiotensin-converting-enzyme inhibitors, angiotensin II receptor blockers, aldosterone receptor antagonists and digoxin), stable coronary artery disease (intensive statin therapy), asthma exacerbations (early inhaled corticosteroids in the emergency department and anticholinergics), chronic obstructive pulmonary disease (long-acting muscarinic antagonists and long-acting beta-2 adrenoceptor agonists) and schizophrenia (second-generation antipsychotics and depot/maintenance antipsychotics).

**Conclusions:**

We identified 11 medications supported by strong evidence and clinical guidelines that could be considered in quality monitoring and improvement strategies to help reduce emergency hospital admission rates. The findings are relevant to health systems with a large burden of chronic disease and those managing increasing pressures on acute health-care services.

**Electronic supplementary material:**

The online version of this article (10.1186/s12916-018-1104-9) contains supplementary material, which is available to authorized users.

## Background

Emergency hospital admissions place a major burden on patients and health-care systems. Large increases in emergency admissions can cause delays and cancellations of elective procedures, prolong emergency department waiting times and increase the risk of hospital-acquired infections [1–5]. Emergency admissions, which comprise 10% of the total health-care budget in some countries, also have significant financial effects [6, 7]. Rates of emergency hospital admissions are rising in many countries, creating emergency-care crises [6, 8, 9].

Identifying interventions that reduce emergency hospital admissions is, therefore, a priority for health services globally, and there have been large-scale evidence reviews to address this [10, 11]. However, a major gap in these systematic assessments has been the omission of medication therapy, which has the potential to reduce use of emergency health-care services. For example, a systematic review of randomised controlled trials (RCTs) has shown that aldosterone receptor antagonists reduce emergency admissions for heart failure by 21% over 20 months [12]. Despite these robust data, there have been no comprehensive reviews to identify and compare medications that effectively and safely prevent hospital admissions in different patient populations. Systematically identifying these beneficial medications is the first step towards monitoring and improving their use in practice. Given that there are existing mechanisms for quality measurement and improvement of clinical practices in many health systems, monitoring and improving medication use may be a feasible and efficient strategy to alleviate some of the burden of emergency admissions compared with the lengthy and expensive process of developing, testing and implementing new complex interventions.

The objectives of this study were to review systematically the evidence to identify medications that affect hospital admissions and prioritise therapies for quality improvement by assessing the quality of evidence and cross-referencing the findings with clinical guidelines.

## Methods

### Protocol and registration

We developed the methods using guidance on systematic reviews and overviews described in the *Cochrane Handbook of Systematic Reviews of Interventions* [13]. The protocol was registered (PROSPERO: CRD42014014779) [14] and published [15]. In our protocol, we specified that we would search for any type of intervention. This overview focuses on medications. Our subsequent overviews will describe the evidence for other types of interventions.

### Types of reviews

We included systematic reviews of RCTs published in English that examined the effect of any medication on emergency hospital admissions in adults (16 years or older). We included reviews that searched two or more electronic databases and assessed and reported the quality of included studies. We defined a medication as any administered chemical or biological product. We included only reviews that reported at least one meta-analysed effect estimate for emergency hospital admissions that was not part of a composite measure. We defined an emergency hospital admission as an unanticipated admission or readmission to hospital that occurred at short notice because of a perceived need for immediate health care [16]. We did not consider admission to an emergency department or an observational unit to be a hospital admission. We excluded studies reporting only scheduled or elective hospital admissions. We excluded superseded Cochrane reviews. We excluded non-Cochrane reviews if all the RCT data on hospital admissions were included in a more recent review of the same intervention and patients. When two reviews reported identical clinical trial data, we selected the review that reported more detailed information, as judged by two of the authors through discussion and consensus (NB and IO).

### Search strategy and study selection

We searched MEDLINE (OvidSP), PubMed, the Cochrane Database of Systematic Reviews & Database of Abstracts of Reviews of Effects, Google Scholar and the websites of ten national funding agencies and health charities, using broad search criteria from inception to February 2016. The search strategy was developed by a library and information scientist (NR). The websites of national funding agencies and health charities were identified using Google searches and by our academic-clinician co-authors. We also contacted experts in emergency admissions and reviewed the reference lists of included reviews to identify additional studies. Details of the search can be found in Additional file 1: Table S1 in the online supplement. Three authors (NB, IO and BF) independently screened titles, abstracts and full-text articles for inclusion. Discrepancies in article inclusion were resolved by discussion. Inter-rater reliability for agreement between authors for title/abstract screening and full-text screening was quantified using Cohen’s kappa statistic .

### Data extraction and quality assessment of reviews

In pairs, we independently extracted information in duplicate on the characteristics of the reviews and assessed their quality using the Assessing the Methodological Quality of Systematic Reviews (AMSTAR) tool [17]. We have provided summary AMSTAR scores when describing review characteristics to give a broad indication of quality; however, full results are also provided as summary scores may obscure important strengths or weaknesses [17]. One minor revision to the AMSTAR tool was made: for item 2 (was there duplicate study selection and data extraction?) reviews did not score ‘yes’ if data selection or extraction was done by one reviewer and checked by another. Information on specific treatment comparisons in the reviews was extracted by one author (NB) and verified by a second (IO, BF, DC, AT, JL, RF, DN, BS, JO, OH, BN or SS). Discrepancies in extracted information or quality assessment scores were resolved by discussion. All reviews and each extracted treatment comparison were assigned a unique identifying number (e.g. review 100 or comparisons 100a and 100b).

### Prioritising therapies through quality assessment of meta-analysed effect estimates and cross-referencing treatments with clinical guidelines

We assessed the quality of meta-analysed effect estimates showing statistically significant effects on admissions. We used criteria from the Grading of Recommendations Assessment, Development and Evaluation (GRADE) Working Group [18]. Using these ratings, we prioritised therapies based on the strength of their evidence base. Quality assessments were completed by one reviewer (NB) and verified by a second (DN). Two generalist clinicians (JL and IO) provided judgements for the indirectness domain, which included assessments of the comparability of populations, interventions, comparators and outcomes between studies and of the applicability of the body of studies to the aims of this overview.

A minimal important threshold in effect had to be defined to utilise the GRADE method, specifically, the imprecision domain [18]. To our knowledge, there is no consensus on what defines a minimal important threshold for hospital admissions. The goal of this research is to manage rising rates of emergency hospital admissions; therefore, we picked a threshold that would achieve this: a 3% relative risk reduction. This threshold is equivalent to the population-adjusted average year-on-year increase in admissions in the UK over the past five years (2011–2016) [9, 19]. The UK is facing the worst emergency-care crisis of any high- or middle-income country that we are aware of. If we were able to identify and implement interventions for every patient group that reduced admission rates by 3%, then the overall admissions rate would cease to rise in the UK. In countries where annual increases are less than 3%, which includes most other high- and middle-income countries facing emergency-care pressures, these interventions would operate to reduce the annual rate of admissions.

We assessed the quality of subgroup effect estimates only if the subgroup analyses were pre-specified and met one or more of the following criteria:

- The subgroup estimate was calculated to explain the presence of substantial heterogeneity in the summary estimate (*I*^2^ ≥ 50% or chi-squared *P* < 0.1) [20].
- The subgroup estimate was calculated from a subset of trials at low risk of bias (as assessed by the original review authors).
- The subgroup estimate showed a significant reduction or increase in hospital admissions, while the summary estimate found no effect.

We considered high and moderate GRADE ratings to represent strong evidence, since the effect estimates are unlikely to change if additional studies are conducted [18, 21].

To prioritise the therapies further, we cross-referenced the list of medications supported by high- or moderate-quality evidence with clinical guidelines. We conducted this analysis to ensure that the overall balance of benefit to harm for the therapies had been judged acceptable by key health-care stakeholders. We first cross-referenced the list with National Institute for Health and Care Excellence (NICE) clinical guidelines [22]. NICE is the largest UK-based organisation providing guidance on clinical care across all disciplines. It was selected based on a consensus among our academic-clinician co-authors. NICE recommends therapies based on their clinical appropriateness, safety, cost-effectiveness and feasibility as judged by clinical experts, health economists, administrators, regulators and patients [23]. We then cross-referenced the short list of NICE-recommended treatments with guidelines in Europe and America. We identified the most recent guidelines by searching the National Guideline Clearinghouse maintained by the Agency for Healthcare Research and Quality. If we could not find a relevant guideline, we then searched for national medical associations and professional societies providing guidance on the treatment for the patient population of interest. The following guidelines were selected from search results by consensus among our clinical-academic co-authors: European Society of Cardiology [24, 25], European Atherosclerosis Society [26], American Heart Association/American College of Cardiology [27–29], European Respiratory Society [30], American Thoracic Society [31], European Psychiatric Association [32] and the American Psychiatric Association [33]. To be considered guideline-based, the treatment must have been recommended by NICE and at least one of the American or European guidelines.

### Data analysis

To standardise reporting, we used international classification systems. We described patient populations using the World Health Organization’s International Statistical Classification of Diseases and Related Health Problems 10th Revision (WHO ICD-10) [34]. We classified medications using the WHO Anatomical Therapeutic Chemical (WHO ATC) classification system [35]. We used the disease definitions and relevant thresholds provided in the reviews, for example, the cut-off for reduced left ventricular ejection fraction in heart failure (i.e., 45%). When possible, we converted reported effect estimates into risk ratios (see Additional file 1: Table S2 for conversion methods) [36–39].

For significant effect estimates that underwent quality assessment, we calculated the number needed to treat to avoid one hospitalisation and the number needed to treat to cause one hospitalisation. For each estimate, we used the median control-group event rate from the RCTs in the meta-analysis [15, 33, 38–40]. When data on event rates were unclear or not reported by the review authors, we obtained the original RCTs and extracted the data.

We used Excel for data extraction and management. For the quantitative data analysis, we used STATA 14 [41]. The results are reported in accordance with PRISMA guidance [42].

## Results

**Fig. 1:**
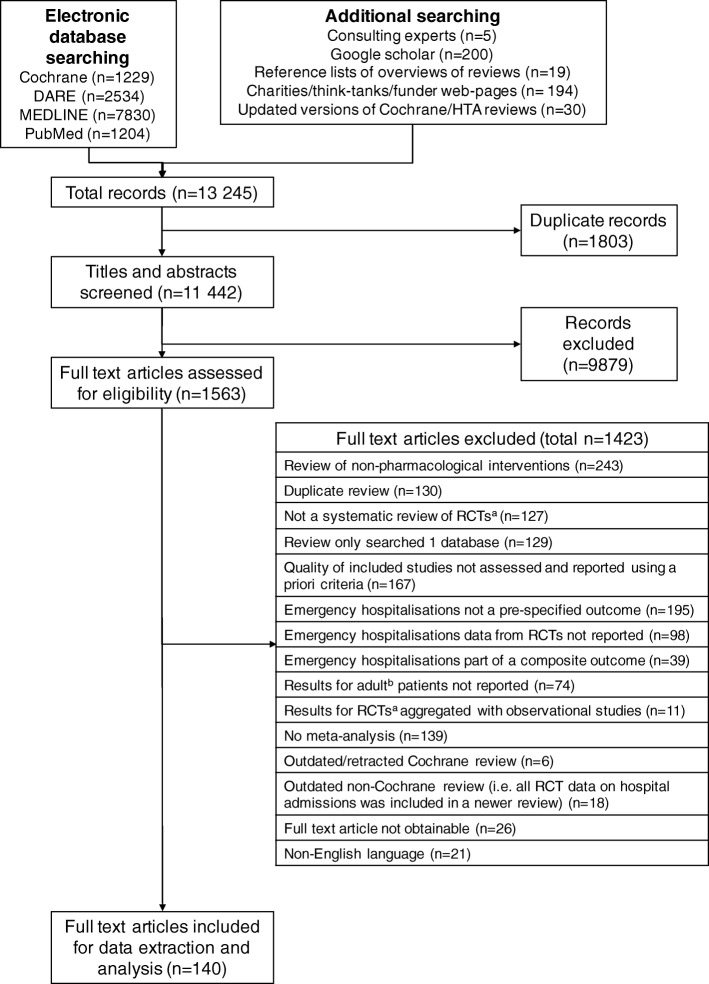
PRISMA flow diagram of study selection. ^a^Randomised controlled trial. ^b^Individual RCTs were defined as adult if they had inclusion criteria of 16 years of age or older for participants. We considered results from a meta-analysis as adult if the included participants’ mean age was 18 years or more across all included trials. RCT randomised controlled trial

We screened 11,442 titles and abstracts and 1563 full text articles (Fig. 1). Of these, 140 systematic reviews met the inclusion criteria. Agreement between reviewers was good for both the title/abstract screen (kappa = 0.85, 95% confidence interval, CI 0.83 to 0.87) and full-text screen (kappa = 0.77, 95% CI 0.73 to 0.82). Complete references, detailed information and full AMSTAR results for each review are included in Additional file 1: Tables S3, S4 and S5.

Table 1 describes the summary characteristics of the 140 reviews. The median AMSTAR score for review quality was 8/11 (interquartile range 6 to 9). Review quality was most often downgraded because the review authors did not state their potential conflicts of interest (AMSTAR criterion 11).

**Table 1:** Summary characteristics of included systematic reviews and the randomised controlled trial data captured by the reviews

Characteristic	Reviews *n* (%)
Review level information (*n* = 140)	
Cochrane reviews	49 (35)
Number of unique medications investigated^a^	100
Number of unique patient populations investigated^b^	47
Reviews focusing on medications for patients with chronic diseases	125 (89)
Patient population^c^	
Diseases of the respiratory system	56 (40)
Diseases of the circulatory system	53 (38)
Factors influencing health status and contact with health services^d^	8 (6)
Diseases of the digestive system	8 (6)
Mental and behavioural disorders	7 (5)
Diseases of the genitourinary system	5 (4)
Pregnancy, childbirth and puerperium	5 (4)
Endocrine, nutritional and metabolic diseases	5 (4)
Mixed patient populations	3 (2)
Multi-morbidity	2 (1)
Other^e^	4 (3)
Hospitalisation was a primary outcome	61 (44)
Review reported pooled effect estimates showing significant reductions in hospital admissions	78 (56)
AMSTAR score for review quality, median (IQR)	8 (6 to 9)
Review year of publication	
2010–2016	96 (69)
2000–2009	42 (30)
1991–1999	2 (1)
Randomised controlled trial (RCT) information	
Number of unique RCTs	1968
Number of unique patients	925,364
Number of unique RCTs reporting admissions data	690
Number of unique patients with admissions data	577,604
Number of patients per RCT reporting hospital admission outcomes, median (IQR)	190 (62 to 603)
Number of treatment comparisons reporting hospital admission outcomes	517

*AMSTAR* Assessing the Methodological Quality of Systematic Reviews, *IQR* interquartile range, *RCT* randomised controlled trial

^a^Unique at the level of pharmacological subgroup in the World Health Organization’s Anatomical Therapeutic Chemical classification system

^b^Unique at the three-character coding level using the 10th revision of the World Health Organization’s International Statistical Classification of Diseases and Related Health Problems (ICD-10)

^c^These specific classifications represent the summary level of coding in ICD-10

^d^For example, patients receiving day surgery

^e^Infection and parasitic disease (*n* = 1 review), diseases of the musculoskeletal system and connective tissue (*n* = 1 review), and diseases of the nervous system (*n* = 2 reviews)

The reviews included an underlying evidence base of 1968 unique RCTs (925,364 patients), of which 690 RCTs reported hospital admission outcomes for 577,604 patients. The number of RCTs underpinning the treatment comparisons ranged from 1 to 184 (median 3), with patient sample sizes ranging from 18 to 88,367 (median 1116). The reviews contained data on 100 unique medications tested in 47 patient populations (Additional file 1: Table S6). Altogether, 125 reviews (89%) examined therapies for patients with chronic diseases. Much of the evidence was for patients with circulatory diseases (53 reviews, 38%) or respiratory diseases (56 reviews, 40%). The most common conditions were heart failure (35 reviews, 25%), chronic obstructive pulmonary disease (COPD; 27 reviews, 19%), acute exacerbations of asthma (20 reviews, 14%) and chronic asthma (14 reviews, 10%). Hospital admissions were identified as a primary outcome in 61 of the reviews (44%). Seventy-eight reviews (56%) reported significantly fewer hospital admissions in intervention arms compared with control arms, while a small minority (8 reviews, 6%) reported significant increases because of the intervention.

### Prioritised list of evidence- and guideline-based medications that significantly reduce emergency hospital admissions

**Fig. 2:**
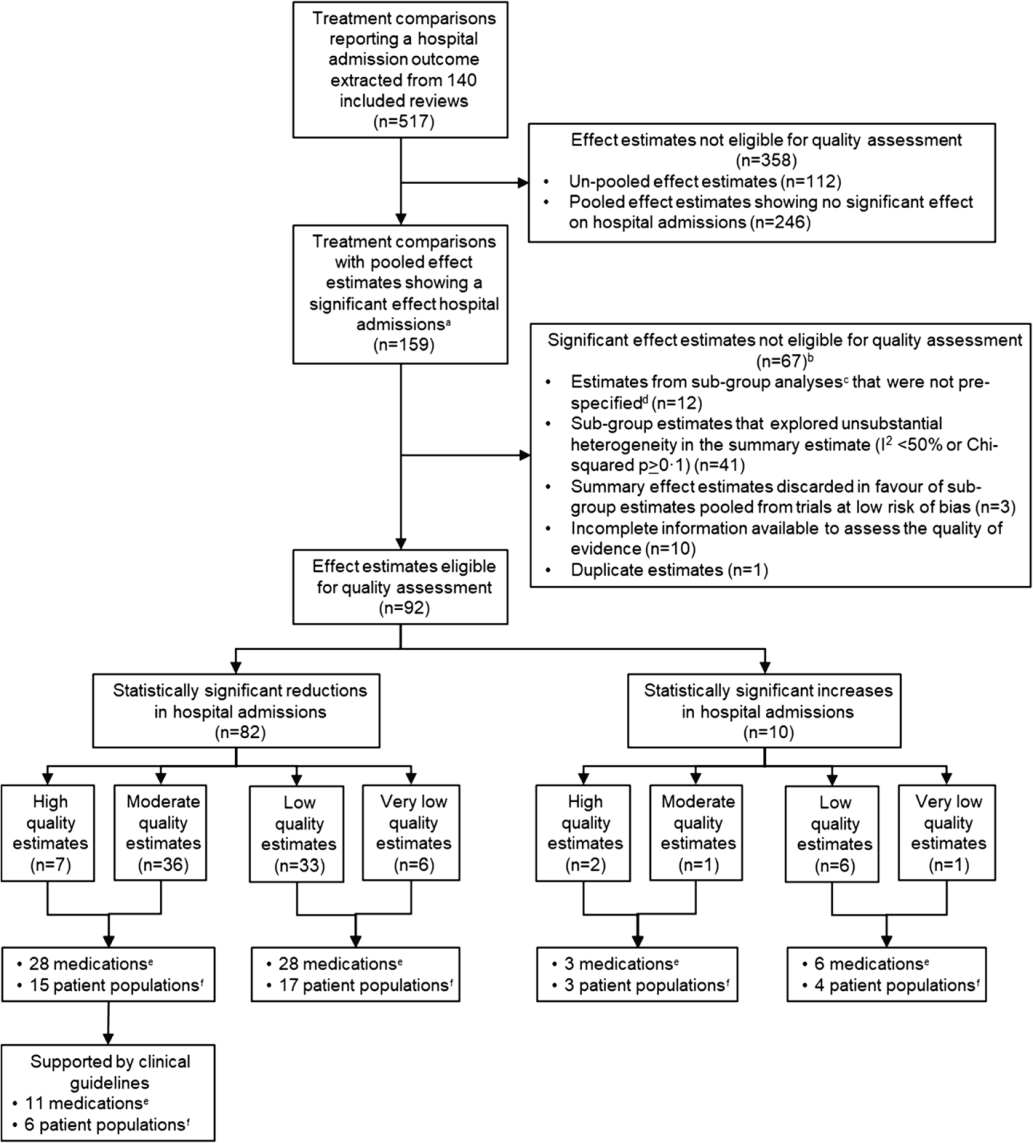
Flow diagram of the process to identify effective medications supported by high- or moderate-quality evidence. ^a^Statistically significant at the *p* < 0.05 level. ^b^Estimates were for subgroup estimates that did not meet our criteria for quality assessment. ^c^Subgroup or sensitivity analysis as defined by authors. ^d^Pre-specified in the methods section of the review article. ^e^Unique at the level of pharmacological subgroup in the World Health Organization’s Anatomical Therapeutic Chemical classification system. ^f^Unique at the three-character coding level using the World Health Organization’s 10th revision of the International Statistical Classification of Diseases and Related Health Problems

From the 140 included reviews, we extracted 517 treatment comparisons that reported hospital admission outcomes (Fig. 2). All treatment comparisons are available in the online database supplement (Additional file 2: Database 1). Of the 517 comparisons, 159 had pooled effect estimates showing a statistically significant effect on hospital admissions. Using GRADE criteria, we identified high and moderate evidence for 28 medications that significantly reduced hospital admissions in 15 patient populations. Evidence summaries for all graded estimates showing a significant reduction in admissions are given in Additional file 1: Table S7.

Of the 28 medications with high- or moderate-quality evidence, 11 were supported by clinical guidelines from the United States, United Kingdom or Europe. Table 2 shows the evidence summaries for these therapies. These 11 medications were tested in 12 comparisons; aldosterone antagonists were tested in two different heart failure groups. Nine treatments were tested against a placebo and three against an active comparator.

**Table 2:** Evidence- and guideline-based medications that reduce emergency hospital admissions

Author, Year^ID No.^	Patient population	Medication treatment	Control	Patients (RCTs)	Mean age^a^	Outcome and mean follow-up^b^	NNT (95% CI) RR (95% CI)	I^2^%	Quality of evidence^c^	Clinical guideline support		
										America	UK	Europe
Reduces hospital admissions from out-patient, day-procedure or community settings												
Xie, 2016^8916a^	Heart failure with reduced left ejection fraction or left ventricular dysfunction	ACE inhibitors	Placebo	12,763 (5)	62 ± 4	HF hospitalisation at 32 months	NNT: 19 (16 to 25) RR: 0.71 (0.66 to 0.78)	0	Moderate	AHA, ACC	NICE	ESC
Xie, 2016^8916b^		Angiotensin-II receptor blockers	Placebo	9878 (4)	65 ± 2	HF hospitalisation at 26 months	NNT: 19 (14 to 30) RR: 0.77 (0.70 to 0.86)	30	Moderate	AHA, ACC	NICE	ESC
Xie, 2016^8916c^		Aldosterone antagonists	Placebo	11,477 (4)	65 ± 3	HF hospitalisation at 18 months	NNT: 22 (15 to 55) RR: 0.71 (0.57 to 0.88	74	Moderate	AHA, ACC	NICE	ESC
Hood, 2014^3415^		Digoxin^d^	Placebo	7262 (4)	61 ± 3	Hospitalisation at 12 months	NNT: 28 (23 to 37) RR: 0.71 (0.64 to 0.77)	29	High	AHA, ACC	NICE	ESC
Le, 2016^8817a^	Heart failure with prior MI	Aldosterone antagonists	Placebo	15,786 (13)	63 ± 6	Hospitalisation at 15 months	NNT: 67 (39 to 233) RR: 0.93 (0.88 to 0.98)	35	Moderate	AHA, ACC	NICE	
Afilalo, 2007^100c^	Stable coronary artery disease	Intensive statin therapy	Moderate statin therapy	27,547 (4)	61 ± 2	HF hospitalisation at 41 months	NNT: 115 (84 to 189) RR: 0.73 (0.63 to 0.83)	4	Moderate	AHA, ACC	NICE	ESC; EAS
Kew, 2013^4002h^	Stable COPD	Long-acting beta 2 agonists	Placebo	3804 (7)	62 ± 2	COPD hospitalisation at 9 months	NNT: 77 (47 to 420) RR: 0.74 (0.57 to 0.95)	35	Moderate	ATS^e^	NICE	ESR^e^
Ni, 2014^8850a^		Long-acting muscarinic antagonists^f^	Placebo	5624 (10)	64 ± 3	COPD hospitalisation at 9 months	NNT: 106 (70 to 321) RR: 0.65 (0.47 to 0.88)	0	High	ATS	NICE	ESR
Reduces hospital admissions from the emergency department												
Edmonds, 2012^2159^	Acute asthma exacerbation	Early use of inhaled corticosteroids	Placebo	377 (5)	38 ± 8	Hospitalisation, Follow-up unclear	NNT: 7 (5 to 12) RR: 0.42 (0.25 to 0.67)	0	Moderate	ATS	NICE	
Rodrigo, 2005^6568a^		Anticholinergics	Beta 2 agonists	1556 (9)	18 to 55	Hospitalisation at ED discharge	NNT: 12 (8 to 26) RR: 0.68 (0.53 to 0.86)	14	Moderate	ATS	NICE	
Reduces readmissions after an index hospital admission												
Leucht, 2012^4508a^	Schizophrenia	Antipsychotic maintenance therapy	No maintenance or placebo	2090 (16)	38 ± 9	Rehospitalisation at 11 months	NNT: 5 (4 to 7) RR: 0.38 (0.27 to 0.55)	45	High	APA	NICE	EPA^g^
Kishimoto, 2013^4065a^		Second-generation antipsychotics	First-generation antipsychotics	2869 (12)	33 ± 6	Hospitalisation at 13 months	NNT: 17 (12 to 48) RR: 0.72 (0.58 to 0.90)	18	Moderate	APA	NICE	EPA^g^

*ID No.* identification number, *RCT* randomised controlled trial, *NNT* number needed to treat to avoid one emergency hospital admission, *RR* risk ratio, *CI* confidence interval, *ED* emergency department, *ACE* angiotensin-converting enzyme, *HF* heart failure, *COPD* chronic obstructive pulmonary disease, *MI* myocardial infarction, *NICE* National Institute for Health and Care Excellence, *ESC* European Society of Cardiology, *AHA* American Heart Association, *ACC* American College of Cardiology, *EAS* European Atherosclerosis Society, *ATS* American Thoracic Society, *ESR* European Respiratory Society, *APA* American Psychiatric Association, *EPA* European Psychiatric Association

^a^Means and standard deviations for patient ages in years. Mean of means and median ages in trials contributing to the pooled estimate. Not all included trials reported age in a form that could be averaged

^b^Mean length of follow-up across trials, unless otherwise specified. Mean of means, medians and total study durations reported in trials contributing to the pooled estimate. Not all included trials reported follow-up in a form that could be averaged. Rounded to the nearest whole month

^c^Moderate- or high-quality evidence according to the Grading of Recommendations Assessment, Development and Evaluation working group criteria

^d^For patients with normal sinus rhythm

^e^The ESR/ATS suggested in their joint 2012 guideline that long-acting beta 2 agonists are a clinically acceptable treatment for stable COPD. However, in their 2017 guideline on preventing exacerbations, they recommended monotherapy with long-acting muscarinic antagonists in preference to long-acting beta 2 agonists if the treatment goal is to prevent a future exacerbation. They indicated that this recommendation places less emphasis on symptomatic relief and that the differential effect on mortality or adverse events is unknown

^f^Aclidinium bromide. Four other meta-analyses with moderate-quality evidence examined other long-acting muscarinic antagonists (e.g. tiotropium) and reported similar effects

^g^The European Psychiatric Association has not published a clinical guideline for schizophrenia but has published a review of national guidelines across Europe and found significant agreement with respect to use of antipsychotic maintenance therapy and of second-generation antipsychotics

There were seven treatments that reduced admissions from out-patient, day-procedure or community settings in patients with heart failure, stable coronary artery disease or stable COPD, while two treatments reduced admissions among patients with acute asthma in the emergency department, and two treatments reduced readmissions after index hospitalisation among patients with schizophrenia.

Information on the drugs, dosing, length of follow-up, ages and event rates from the RCTs that contributed data to the high- and moderate-quality effect estimates is listed in Additional file 1: Table S8. Information on the GRADE quality assessments for each estimate is presented in Additional file 1: Table S9.

### Medications that increase admissions

While reviewing the evidence for medications that reduced admissions, we also identified high- and moderate-quality evidence for three therapies that significantly increased hospital admissions: cyclooxygenase-2 (COX-2) inhibitors in patients for whom non-steroidal anti-inflammatory drugs (NSAIDs) were indicated, intermittent antipsychotic drug therapy in patients with schizophrenia and fluticasone in patients with COPD (Table 3). Evidence summaries for graded estimates showing a significant increase in admissions are given in Additional file 1: Table S10. Information on the drugs, dosing, length of follow-up, ages and event rates from the RCTs that contributed data to the high- and moderate-quality effect estimates is listed in Additional file 1: Table S8. Information on the GRADE quality assessments for each estimate is presented in Additional file 1: Table S9.

**Table 3:** Medications that increase emergency hospital admissions

Author, Year^ID No.^	Patient population	Medication treatment	Control	Patients (RCTs)	Mean age^a^	Outcome mean follow-up^b^	NNH (95% CI) RR (95% CI)	I^2^%	Quality of the evidence^c^
Increase hospital admissions from out-patient, day-procedure, or community settings									
Baigent, 2013^1653a^	Patients indicated for NSAID treatment	COX2-inhibitors	Placebo	88,367 (184)	Unclear	HF hospitalisation, Follow-up unclear	NNH: 3 (2 to 6) rr: 2.28 (1.62 to 3.20)^d^	Unclear	Moderate
Sampson, 2013^6806f^	Schizophrenia	Intermittent antipsychotic therapy	Maintenance antipsychotic therapy	661 (6)	35 ± 5	Hospitalisation at 21 months	NNH: 7 (4 to 15) RR: 1.58 (1.28 to 1.97)	19	High
Kew, 2014^4004z^	Moderate to severe stable COPD	Fluticasone	Placebo	16,338 (15)	64 ± 1	Pneumonia hospitalisation at 12 months	NNH: 164 (114 to 259) RR: 1.81 (1.51 to 2.17)	0	High

*ID No.* identification number, *RCT* randomised controlled trial, *NNH* number needed to treat to cause one emergency hospital admission, *RR* risk ratio, *CI* confidence interval, *NSAID* non-steroidal anti-inflammatory drugs, *COX2* Cyclooxygenase 2, *HF* heart failure, *rr* rate ratio, *COPD* chronic obstructive pulmonary disease

^a^Means and standard deviations for patient ages in years. Mean of means and median ages in trials contributing to the pooled estimate. Not all included trials reported age in a form that could be averaged

^b^Mean length of follow-up across trials, unless otherwise specified. Mean of means, medians and total study durations reported in trials contributing to the pooled estimate. Not all included trials reported follow-up in a form that could be averaged. Rounded to the nearest whole month

^c^Moderate- or high-quality evidence according to the Grading of Recommendations Assessment, Development and Evaluation working group criteria

^d^Rate ratio

## Discussion

### Summary of findings

We examined 140 reviews of 100 medications, and identified high- to moderate-quality evidence for 28 therapies that significantly reduced admissions in 15 different populations. Eleven of these therapies were supported by major clinical guidelines from the United States, the United Kingdom or Europe. We also identified high- and moderate-quality evidence for three medications that increased admissions.

### In the context of the literature

Previous reviews of interventions to reduce hospital admissions have focused on non-pharmacological initiatives [10, 43–45]. Our study, therefore, fills an important gap as the first systematic investigation of medications that affect hospital admissions. We mapped a large evidence base and prioritised therapies based on the quality of the evidence and support of clinical guidelines.

Our results complement a growing body of evidence about drug-related hospital admissions. Several systematic reviews of observational studies have shown that approximately 3% of all emergency hospitalisations are related to suspected adverse drug reactions and drug–drug interactions [46–51]. Drugs often associated with hospital admissions include antiplatelet drugs, diuretics, renin-angiotensin system blockers, NSAIDs and anticoagulants. However, the reviews have not considered the number of admissions these drugs help to avoid and therefore, have not provided evidence about their net effects on admissions. Our study complements this literature, as it shows a strong body of evidence supporting a beneficial reduction in admissions for renin-angiotensin system blockers [angiotensin-converting-enzyme (ACE) inhibitors and angiotensin II receptor blockers] and aldosterone receptor antagonists. We have also identified strong evidence for a harmful increase in cause-specific admissions due to heart failure from the use of COX2 inhibitors and pneumonia from the use of inhaled corticosteroids in COPD patients. Many of the other drugs commonly associated with drug-related admissions did not appear in our study. Several reviews of RCTs focusing on antiplatelet drugs and anticoagulants were excluded, because they reported admissions as part of composite outcomes. The net effect of antiplatelet drugs and anticoagulants on hospital admissions is, therefore, not clear from the published systematic review literature.

### Implications for practice

Policymakers and commissioners may use these results to prioritise quality measurement and improvement efforts. We have systematically identified a list of 11 evidence- and guideline-based treatments for five major chronic diseases: heart failure, stable coronary artery disease, COPD, asthma and schizophrenia. These diseases cause millions of hospital admissions each year globally [52–56] and account for about 5% of all emergency admissions in high-income countries [57–59]. Yet, there is evidence of significant variation in the prescribing of some these 11 therapies in the United States and Europe, including under-dosing and under-prescribing [60–62]. Therefore, improving utilisation of these medications could translate to substantial reductions in hospital admissions. Potential improvement targets include minimising gaps in prescribing, correcting over- and under-dosing, and improving patient adherence, although the specific target of any improvement initiatives should be driven by locally identified shortfalls in care.

The results of this study may be fed into existing mechanisms for tracking and improving clinical practices in health systems. Prescribing data for some of the 11 evidence- and guideline-based medications are currently monitored in several health systems, for example, use of ACE inhibitors, angiotensin II receptor blockers and beta blockers is currently measured as part of the UK Quality and Outcomes Framework, a pay-for-performance incentive structure that has demonstrably reduced prescribing gaps for these medications and improved prescribing efficiency for other medications [63–65]. All of the top 11 medications we identified could be considered for inclusion in these types of quality assurance and incentive structures. This pathway to optimising medication utilisation may be a feasible strategy to help reduce emergency hospital admissions.

For some of the therapies in this review, bridging treatment gaps and improving prescribing may result in immediate benefits and may rely only on a small number of stakeholders. For example, we found evidence that treating patients with acute exacerbations of asthma in the emergency department with inhaled corticosteroids or anticholinergics helped to avoid hospital admissions. Optimal utilisation of these medications could be achieved through direct efforts by emergency department physicians and hospital pharmacists.

However, many of the therapies in this review form part of ongoing chronic disease management, and beneficial effects occur after months of use. Optimal utilisation of these medications would require coordination of multiple stakeholders, including physicians in different specialties, home care or case management nurses, community pharmacists and patients.

We can estimate potential reductions in hospital admissions by combining the results of our study with data on existing treatment gaps and disease prevalence. For example, in studies of prescribing for heart failure in the United States and Europe, 13% of patients with reduced left ventricular ejection fractions who are eligible for treatment do not receive the first-line therapy [60, 66]. It has been estimated that 1.5% of people in developed countries have heart failure, of whom 40% have reduced ejection fractions [52, 67–69]. Therefore, about 400,000 Europeans and 250,000 Americans with heart failure and reduced ejection fractions are eligible for first-line therapy but are not receiving it [70]. Based on the numbers needed to treat and baseline hospitalisation event rates that we have reported for ACE inhibitors and angiotensin II receptor blockers, closing these treatment gaps could help to avoid approximately 28,000 (95% CI 24,000 to 37,000) hospital admissions in Europe and 18,000 (95% CI 15,000 to 24,000) admissions in the United States per year.

Our results also reinforce the dangers of prescribing COX2 inhibitors, particularly for patients at risk of cardiovascular disease, inhaled corticosteroids in patients with moderate to severe stable COPD, and intermittent antipsychotic drug therapy for patients with schizophrenia. It is, therefore, reassuring that the harms associated with the use of these drugs and steps to ensure that they are used appropriately, if at all, have been reported widely in clinical and government prescribing guidelines [71–73].

In addition to high- and moderate-quality evidence, this study identified 28 medications with low- and very low-quality evidence for reducing admissions. According to the GRADE working group, these estimates for reducing admissions are likely to change if additional research were conducted [18]. Therefore, given the uncertainty around the estimates of effects for these 28 medications, we would not recommend prioritising these for reducing emergency admissions. However, we recognise that the level of evidence required to act may vary depending on the circumstances, the stakeholders involved and the available evidence (which may change over time). If limited evidence is available and there is a pressing need to act, lower-quality evidence may be sufficient to justify cautious implementation of an intervention. Our prioritisation of medications with high- and moderate-quality evidence does not prevent stakeholders from using the low- and very low-quality evidence if justified in the context of their health-care settings; however, we recommend a robust evaluation to ensure resources are appropriately allocated to those interventions most likely to impact on practice.

### Implications for research

Only 1% of the reviews examined the effect of medications in patients with multi-morbidity. Given the challenges of effective clinical management and high hospitalisation risk for patients with multiple diseases [47, 74], we need to identify which medication combinations most help multi-morbid patients to avoid hospitalisation.

Low- and very low-quality evidence indicates the need for high-quality research to increase confidence in the reliability of effect estimates. Some of the medications in this overview were supported by low- and very low-quality evidence, suggesting a need for additional high-quality research. Hospitalisations, however, are only one important patient and health system outcome. A larger set of core outcomes that reflect patient and health system priorities should be considered when establishing research priorities, including assessment of mortality, adverse events, quality of life and cost.

Similarly, there were 17 drugs with high- and moderate-quality evidence that were not supported by clinical guidelines. We did not record whether the medications had been evaluated. In formulating guidelines, the effect of an intervention on reducing emergency admissions would form only one of many criteria considered by multi-disciplinary panels of stakeholders. We would not recommend that drugs be included in guidelines or considered for inclusion solely because they reduce hospital admissions.

Researchers should consider reporting rates of hospital admissions, as opposed to ratio measures; 476 of the 517 (92%) effect estimates we reviewed were reported as odds or risk ratios. These are crude measures of hospitalisation, as they assess admission as a binary outcome: present or absent. These effect measures equate a patient who has had one admission during follow-up with a patient who has had five admissions. In patients with chronic diseases, such as heart failure or COPD, for which hospital admissions are common, rate-based measures may have greater utility in evaluating the effectiveness of interventions.

### Strengths and limitations of the study

This study has three key strengths. First, it was comprehensive. We identified, analysed and synthesised information on nearly one million patients to identify the highest quality evidence for medications that affect emergency hospital admissions. Secondly, we minimised the impact of duplicate RCT evidence between reviews by excluding outdated reviews; every review we included has a unique set of hospital admission data. Thirdly, we classified all patient populations using ICD-10 and therapies using WHO ATC. This helped to homogenise and simplify the data, which was heterogeneously reported in the systematic reviews. When possible, we also converted quantitative data to comparable measures and units (i.e. risk ratios for estimates and months for follow-up duration). This will enable users of our review to navigate and interpret this large body of evidence.

This study has some limitations. First, although we extracted and reported all effect estimates, we have conducted only quality assessments on significant estimates. While it may be useful for decision makers to know the quality of evidence for all tested interventions, our aim was to support decision-making by identifying and prioritising therapies for which an effect has been demonstrated. All the estimates are listed in the online database (Additional file 2). Secondly, we planned to examine secondary outcomes, such as mortality and cost; however, feasibility concerns emerged during the conduct of the review. Therefore, we analysed only our primary outcome, hospital admissions. To provide information about other outcomes, we have extracted and presented conclusions from the abstract of each review. Furthermore, medications that were supported by high- or moderate-quality evidence were cross-referenced with clinical guidelines to identify those for which the overall balance of benefit to harm was judged to be acceptable by key health-care stakeholders. Thirdly, we excluded reviews that reported hospital admissions only as part of a composite end point. We may, therefore, have excluded potentially valuable therapies. However, by excluding composite outcomes, we can be confident that the effective therapies we identified have a significant effect on hospital admissions. Fourthly, we analysed clinical guidelines from at least one national organisation in the UK, Europe or America and identified support for 11 of the medications in this overview. However, it is possible that there are other relevant clinical guidelines that we did not analyse that support additional medications from this study. Readers of the overview may combine the findings with relevant clinical guidelines in their field to identify additional medications that may be considered for quality measurement and improvement to reduce hospital admissions. Fifthly, our GRADE ratings of indirectness were assessed by generalist clinicians and it is possible that clinical specialists (e.g. cardiologists) may have different opinions regarding the comparability of certain subgroups of patients and interventions. Finally, we planned to include reviews in all languages; however, feasibility concerns emerged during the conduct of the review and as a result we included only reviews published in English. Therefore, we may have missed effective therapies examined in non-English reviews.

## Conclusions

We identified 11 medications supported by strong evidence and clinical guidelines that could be considered in quality monitoring and improvement strategies to help reduce emergency hospital admission rates. The findings are relevant to health systems with a large burden of chronic disease and those managing increasing pressures on acute health-care services.

## Additional files

Additional file 1: **Table S1.** Search strategies and results of searches. **Table S2.** Formulae and calculations used in analyses. **Table S3.** Complete references. **Table S4.** Information about included reviews. **Table S5.** Full results for AMSTAR review quality assessment. **Table S6.** Distribution of evidence by patient population. **Table S7.** GRADE estimates showing reductions in admissions. **Table S8.** Trial information underpinning GRADE estimates. **Table S9.** Details of GRADE ratings. **Table S10.** GRADE estimates showing an increase in admissions. (PDF 47591 kb)

Additional file 2: Database 1. (XLSX 130 kb)
