# Supplementary material for: Medications that reduce emergency hospital admissions: an overview of systematic reviews and prioritisation of treatments
Source: BMC Med. 2018 Jul 26;16:115. doi: 10.1186/s12916-018-1104-9 (PMC6060538; doi:10.1186/s12916-018-1104-9)
Supplement: Supplementary file 1 — Table S1. Search strategies and results of searches. Table S2. Formulae and calculations used in analyses. Table S3. Complete references. Table S4. Information about included reviews. Table S5. Full results for AMSTAR review quality assessment. Table S6. Distribution of evidence by patient population. Table S7. GRADE estimates showing reductions in admissions. Table S8. Trial information underpinning GRADE estimates. Table S9. Details of GRADE ratings. Table S10. GRADE estimates showing an increase in admissions. (PDF 47591 kb) [file 12916_2018_1104_MOESM1_ESM.pdf]

Chapter 3 Appendix

Contents

Table S1. Search strategies and results of searches 2
Table S2. Formulae and calculations used in analyses 4
Table S3. Complete references 5
Table S4. Information about included reviews 11
Table S5. Full Results for AMSTAR Review Quality Assessment....... 33
Table S6. Distribution of evidence by patient population 37
Table S7. GRADED estimates showing reductions in admissions. 42
Table S8. Trial information underpinning GRADED estimates 52
Table S9. Details of GRADE ratings 112
Table S9 cont. Inconsistency: domain 2 for downgrading 118
Table S9 cont. Indirectness: domain 3 for downgrading. 121
Table S9 cont. Imprecision: domain 4 for downgrading 125
Table S9 cont. Publication bias: domain 5 for downgrading 128
Table S10. GRADED estimates showing an increase in admissions 131

Table S1. Search strategies and results of searches

**Electronic databases with peer-reviewed literature**	
**MEDLINE**<br>Inception to February 29, 2016 for medication reviews	
1	Hospitalization/
2	Patient admission/
3	Patient readmission/
4	(admission? or admitted or readmission? or re-admission? or readmitted or re-admitted or hospitali* or rehospitali* or re-hospitali*).ti,ab.
5	1 or 2 or 3 or 4
6	Cochrane database of systematic reviews.jn. or search.tw. or meta-analysis.pt. or Medline.tw. or systematic review.tw.
7	5 and 6
**Cochrane Database of Systematic Reviews**<br>Inception to February 29, 2016 for medication reviews	
1	MeSH: Hospitalization
2	MeSH: Patient Admission
3	(admission or admitted or readmission or re-admission or readmitted or re-admitted or hospitali* or rehospitali* or re-hospitali*):ti,ab
4	(#1 OR #2 OR #3)
**Database of Abstracts of Reviews of Effects**<br>Inception to February 29, 2016 for medication reviews	
1	MeSH: Hospitalization
2	MeSH: Patient Admission
3	(admission or admitted or readmission or re-admission or readmitted or re-admitted or hospitali* or rehospitali* or re-hospitali*):ti,ab
4	(#1 OR #2 OR #3)
**PubMed**<br>January 1, 2014 to July 1, 2014 AND August 29, 2015 to February 29, 2016 for medication reviews	
1	((((((((admission[Title/Abstract]) OR admitted[Title/Abstract]) OR readmission[Title/Abstract]) OR re-admission[Title/Abstract]) OR readmitted[Title/Abstract]) OR re-admitted[Title/Abstract]) OR hospitali*[Title/Abstract]) OR rehospitali*[Title/Abstract]) OR re-hospitali*[Title/Abstract]
2	Date limited to six months before search date (to capture articles not yet indexed in MEDLINE)
3	Limited using built in Systematic Reviews
**Grey literature**	
**Google scholar** targeted search<br>Inception to April 28, 2016 for medication reviews	
1	(((hospital* OR (hospital AND admi*) OR (readmi*) OR (re-admi*)) AND ((systematic review) OR (literature review) OR ((meta) AND (analy*))) AND ((random*) OR (trial) OR (RCT)))
2	Sorted by relevance
**Google scholar** broad search<br>Inception to April 28, 2016 for medication reviews	
1	Hospital admissions AND interventions AND systematic review
2	Sorted by relevance
**National Institutes of Health Research Website**<br>https://www.journalslibrary.nihr.ac.uk/programmes/<br>Journals library; ongoing research; programme studies, all statuses; health technology assessment programmes<br>Inception to April 28, 2016 for medication reviews	
1	hospital admissions
**Research Council United Kingdom Website** (seven major funding agencies)<br>http://gtr.rcuk.ac.uk/search/publication?term=admission+AND+systematic+review&fetchSize=25&selectedSortableField=score&selectedSortOrder=DESC&fields=per.on%2Cper.fn%2Cpub.a%2Cper.sn%2Cper.org.n%2Cpub.t%2Cper.pro.abs%2Cper.pro.t%2Cper.fnsn%2Cpro.t%2Corg.n%2Cpro.gr%2Cpro.a	

Inception to April 28, 2016 for medication reviews	
1	Hospital admissions (journal article/review)
1	Admission AND systematic review
1	Admission AND review
**King's Fund Website** http://kingsfund.koha-ptfs.eu<br>Inception to April 28, 2016 for medication reviews	
1	Unplanned hospital admissions
2	Unscheduled hospital admissions
3	Emergency hospital admissions
4	1 OR 2 OR 3
5	Review
6	4 AND 5
**Nuffield Trust Website** http://www.nuffieldtrust.org.uk/<br>Inception to April 28, 2016 for medication reviews	
1	Admissions
1	Emergency
1	Unplanned
1	Unscheduled
1	Hospitalised
1	Hospitalisation
**Other sources**	
**Experts**<br>June 2014 to April 28, 2016 for medication reviews	
1	Approached researchers that presented on unscheduled hospital admissions at a King's fund conference, Evidence Live 2015 conference, and the 2015 Society for Academic Primary Care Conference. I described the research and asked the researchers to identify relevant systematic reviews.
**Reference lists of included reviews**	
1	All included systematic reviews were assessed.

Table S2. Formulae and calculations used in analyses

Desired conversion	Formula		Source
Calculating the risk ratio from the odds ratio	$RR = \frac{OR}{(1 - CER) + (CER \times OR))}$		Zhang et al.[1]
Calculating the corresponding intervention group event rate per 100 from the median control group event rate from risk ratios, rate ratios, hazard ratios, odds ratios, number needed to treat, absolute difference, and mean risk difference	$Corresponding\ intervention\ group\ risk = 100\ \times median\ CER\ \times RR$		The Cochrane Collaboration[2]
	$Corresponding\ intervention\ group\ risk = 100\ \times median\ CER\ \times rate\ ratio$		
	$Corresponding\ intervention\ group\ risk = 100\ \times median\ CER\ \times HR$<br>*At the end of follow-up or last time point		
	$Corresponding\ intervention\ group\ risk = \frac{OR\ \times CER}{(1 - CER) + (OR \times CER))}$		
	$Corresponding\ intervention\ group\ risk = \left(\frac{Median\ CER}{per\ 100\ patients} - \frac{NNT}{per\ 100\ patients}\right)$		Derived
	$Corresponding\ intervention\ group\ risk = 100 \times (CER + Difference\ in\ risk)$		
	$Corresponding\ intervention\ group\ risk = 100 \times (CER + Mean\ difference\ in\ risk)$		
Calculating the number needed to treat from an odds ratio	NNT =((1-(CER x (1 - OR))) / ((1 - CER) x (CER) x (1 - OR)))		The Centre for Evidence-Based Medicine[3]
Calculating the number needed to harm from an odds ratio	NNH = ((CER x (OR - 1))+1) / (CER x (OR - 1) x (1 - CER))		
Calculating the number needed to treat from a hazard ratio	NNT = $\frac{1}{((1-CER(t))^{HR})-(1-CER(t))}$	*Note: Where t is the time point of interest	Derived from Altman et al.[4]
Calculating the number needed to harm from a hazard ratio	NNH = $\frac{1}{((1-CER(t))^{HR})-(1-CER(t))} \times -1$	*Note: To be used when the hazard ratio >1 indicating an increase in hospital admissions associated with the intervention; Where t is the time point of interest	
Calculating the absolute risk difference from the risk ratio	$Absolute\ risk\ difference = \lvert CER\ x\ (RR - 1)\rvert$		Newcombe et al.[5]
Standardising follow-up times to months	30.4 days per month and 4.3 weeks per month.		
Number needed to treat from absolute risk difference	NNT = $\frac{1}{Absolute\ risk\ difference}$		The Centre for Evidence-Based Medicine[7]

Abbreviations: RR= risk ratio; OR= odds ratio; CER= control event rate; NNT= Number needed to treat; NNH= Number needed to harm; HR= Hazard ratio; t=time point of interest

---

[1]Zhang J, Yu KF. What's the relative risk? A method of correcting the odds ratio in cohort studies of common outcomes. *JAMA*. 1998;280(19):1690-1691. doi:10.1001/jama.280.19.1690.

[2]The Cochrane Collaboration. Chapter 11.5.5 Statistical considerations in "Summary of findings" tables. Cochrane Handbook for Systematic Reviews of Interventions. 2011. www.cochrane-handbook.org

[3]The Centre for Evidence-Based Medicine. Number Needed to Treat (NNT). http://www.cebm.net/number-needed-to-treat-nnt/. Published 2012. Accessed June 1, 2014.

[4]Altman DG, Andersen PK. Calculating the number needed to treat for trials where the outcome is time to an event. *Bmj*. 1999;319(7223):1492-1495. doi:10.1136/bmj.319.7223.1492.

[5] Newcombe RG, Bender R. Implementing GRADE: calculating the risk difference from the baseline risk and the relative risk. *Evid Based Med*. 2014;19(1):6-8. doi:10.1136/eb-2013-101340.

Table S3. Complete references

Author, Year, Review ID	Reference
Afilalo, 2007, 100	Afilalo J, Majdan AA, Eisenberg MJ. Intensive statin therapy in acute coronary syndromes and stable coronary heart disease: a comparative meta-analysis of randomised controlled trials. Heart. 2007;93(8):914-921. doi:10.1136/hrt.2006.112508.
Ahmed, 2014, 129	Ahmed F, Lindley MC, Allred N, Weinbaum CM, Grohskopf L. Effect of influenza vaccination of healthcare personnel on morbidity and mortality among patients: Systematic review and grading of evidence. Clin Infect Dis. 2014;58(1):50-57. doi:10.1093/cid/cit580.
Akioyamen, 2016, 8711	Akioyamen L, Levine M, Sherifali D, et al. Cardiovascular and cerebrovascular outcomes of long-term angiotensin receptor blockade: Meta-analyses of trials in essential hypertension. J Am Soc Hypertens. 2016;10(1):55-69. doi:10.1016/j.jash.2015.11.005.
Anderson, 2015, 8714	Anderson D, Kew K, Boyter A. Long-actingmuscarinic antagonists (LAMA) added to inhaled corticosteroids (ICS) versus the same dose of ICS alone for adults with asthma. Cochrane Database Syst Rev. 2015;(8). doi:10.1002/14651858.CD011397.pub2.
Assasi, 2009, 378	Assasi N, Blackhouse G, Xie F, et al. Anti-TNF-α Drugs for Refractory Inflammatory Bowel Disease: Clinical- and Cost-Effectiveness Analyses [Technology Report Number 120]. Ottawa; 2009.
Badve, 2011, 446	Badve S V., Roberts MA, Hawley CM, et al. Effects of beta-adrenergic antagonists in patients with chronic kidney disease: A systematic review and meta-analysis. J Am Coll Cardiol. 2011;58(11):1152-1161. doi:10.1016/j.jacc.2011.04.041.
Baigent, 2013, 1653	Baigent C, Bhala N, Emberson J, et al. Vascular and upper gastrointestinal effects of non-steroidal anti-inflammatory drugs: Meta-analyses of individual participant data from randomised trials. Lancet. 2013;382(9894):769-779. doi:10.1016/S0140-6736(13)60900-9.
Barr, 2006, 548	Barr RG, Bourbeau J, Camargo C a, Ram FS. Tiotropium for stable chronic obstructive pulmonary disease. Cochrane Database Syst Rev. 2005;(2):Cd002876. doi:10.1002/14651858.CD002876.pub2.
Baumeister, 2011, 593	Baumeister H, Hutter N, Bengel J. Psychological and pharmacological interventions for depression in patients with coronary artery disease. Cochrane Database Syst Rev. 2011;(9). http://www.mrw.interscience.wiley.com/cochrane/clsysrev/articles/CD008012/pdf_fs.html%5Cnhttp://ovidsp.ovid.com/ovidweb.cgi?T=JS&PAGE=reference&D=emed9&NEWS=N&AN=2010193126.
Beck, 2013, 615	Beck AM, Holst M, Rasmussen HH. Oral nutritional support of older (65 years+) medical and surgical patients after discharge from hospital: Systematic review and meta-analysis of randomized controlled trials. Cochrane Database Syst Rev. 2013;27:19-27. doi:10.1177/0269215512445396.
Blitz, 2005, 813	Blitz M. Aerosolized Magnesium Sulfate for Acute Asthma. CHEST J. 2005;128(1):337. doi:10.1378/chest.128.1.337.
Bonsu, 2016, 8729	Bonsu KO, Reidpath DD, Kadirvelu A. Lipophilic Statin Versus Rosuvastatin (Hydrophilic) Treatment for Heart Failure: a Meta-Analysis and Adjusted Indirect Comparison of Randomised Trials. Cardiovasc Drugs Ther. 2016;30(2):177-188. doi:10.1007/s10557-015-6636-z.
Briasoulis, 2015, 8731	Briasoulis A, Palla M, Afonso L. Meta-Analysis of the Effects of Carvedilol Versus Metoprolol on All-Cause Mortality and Hospitalizations in Patients With Heart Failure. Am J Cardiol. 2015;115(8):1111-1115. doi:10.1016/j.amjcard.2015.01.545.
Brophy, 2001, 1021	Brophy J, Joseph L, Rouleau J. B-Blockers in Congestive Heart Failure. Ann Intern Med. 2001.
Burch, 2009, 1080	Burch J, Paulden M, Conti S, et al. Antiviral drugs for the treatment of influenza: a systematic review and economic evaluation. Health Technol Assess. 2009;13(58):1-265, iii-iv. doi:10.3310/hta13580.
Cammarano, 2016, 8735	Cammarano C, Silva M, Comee M, Donovan JL, Malloy MJ. Meta-analysis of Ivabradine in Patients with Stable Coronary Artery Disease with and Without Left Ventricular Dysfunction. Clin Ther. 2016;38(2):387-395. doi:10.1016/j.clinthera.2015.12.018.
Campschroer, 2014, 1164	Campschroer T, Zhu Y, Duijvesz D, Grobbee DE, Lock MTWT. Alpha-blockers as medical expulsive therapy for ureteral stones. Cochrane database Syst Rev. 2014;4(4):CD008509. doi:10.1002/14651858.CD008509.pub2.
Cates, 2013, 1249	Cates CJ, Rowe BH, Bara A. Holding chambers versus nebulisers für beta-agonist treatment of acute asthma (Cochrane Review). Cochrane database Syst Rev (Online Updat Software). 2013;(3):3-6. doi:10.1002/14651858.CD000052.pub3.Copyright.
Cawood, 2012, 1264	Cawood AL, Elia M, Stratton RJ. Systematic review and meta-analysis of the effects of high protein oral nutritional supplements. Ageing Res Rev. 2012;11(2):278-296. doi:10.1016/j.arr.2011.12.008.
Ceron-Litvoc, 2009, 1281	Ceron-Litvoc D, Soares B, Geddes J, Litvoc J, Silva de Lima M. Comparison of carbamazepine and lithium in treatment of bipolar disorder: a systematic review of randomized controlled trials. Hum Psychopharmacol. 2009;24:19-28.
Chauhan, 2012, 1348	Chauhan BF, Ducharme FM. Addition to inhaled corticosteroids of long-acting beta 2 -agonists versus anti-leukotrienes for chronic asthma. Cochrane Database Syst Rev. 2014;(1). doi:10.1002/14651858.CD003137.pub5.
Chauhan, 2014, 8681	Chauhan B, Ducharme F. Anti-leukotriene agents compared to inhaled corticosteroids in the management of recurrent and / or chronic asthma in adults and children (Review). Cochrane Database Syst Rev. 2012;(4). doi:10.1002/14651858.CD002314.pub3.Anti-leukotriene.
Chen, 2015, 8742	Chen Y, Wang H, Lu Y, Huang X, Liao Y, Bin J. Effects of mineralocorticoid receptor antagonists in patients with preserved ejection fraction: a meta-analysis of randomized clinical trials. BMC Med. 2015;13:10. doi:10.1186/s12916-014-0261-8.
Cheyne, 2015, 8744	Cheyne L, Irvin-Sellers MJ, White J. Tiotropium versus ipratropium bromide for chronic obstructive pulmonary disease. Cochrane Database Syst Rev. 2015;(9):N.PAG. doi:10.1002/14651858.CD009552.pub3.www.cochranelibrary.com.

Chong, 2012, 1438	Chong J, Karner C, Poole P. Tiotropium versus long-acting beta-agonists for stable chronic obstructive pulmonary disease. Cochrane Database Syst Rev. 2012;9(9):CD009157. doi:10.1002/14651858.CD009157.pub2.
Coeytaux, 2014, 8747	Coeytaux R, Schmit K, Kraft B, et al. Comparative Effectiveness and Safety of Drug Therapy for Pulmonary Arterial Hypertension: A Systematic Review and Meta-analysis. Chest. 2014;145(5):1055-1063. doi:10.1378/chest.13-1864.
Cordina, 2005, 1606	Cordina J, Mead G. Pharmacological cardioversion for atrial fibrillation and flutter. Indian Pacing Electrophysiol J. 2005;6(1):31. http://www.ncbi.nlm.nih.gov/pubmed/16943892.
Costa, 2013, 1629	Costa J, Magro F, Caldeira D, Alarcao J, Sousa R, Vaz-Carneiro A. Infliximab reduces hospitalizations and surgery interventions in patients with inflammatory bowel disease: a systematic review and meta-analysis. Inflamm Bowel Dis. 2013;19(10):2098-2110.
Danchin, 2006, 1752	Danchin N, Cucherat M, Thuillez C, Durand E, Kadri Z, Steg PG. Angiotensin-converting enzyme inhibitors in patients with coronary artery disease and absence of heart failure or left ventricular systolic dysfunction: An overview of long-term randomized controlled trials. Arch Intern Med. 2006;166(7):787-796. doi:10.1001/archinte.166.7.787.
DiNicolantonio, 2013, 1991	Dinicolantonio JJ, Lavie CJ, Fares H, Menezes AR, O'Keefe JH. Meta-analysis of carvedilol versus beta 1 selective beta-blockers (atenolol, bisoprolol, metoprolol, and nebivolol). Am J Cardiol. 2013;111(5):765-769. doi:10.1016/j.amjcard.2012.11.031.
Doyle, 2009, 2055	Doyle JF, Ho KM. Benefits and risks of long-term amiodarone therapy for persistent atrial fibrillation: a meta-analysis. Mayo Clin Proc. 2009;84(3):234-242. doi:10.1016/S0025-6196(11)61140-3.
Ducharme, 2010, 2095	Ducharme FM, Ni Chroinin M, Greenstone I, Lasserson TJ. Addition of long-acting beta2-agonists to inhaled steroids versus higher dose inhaled steroids in adults and children with persistent asthma. Cochrane Database Syst Rev. 2010;(4):CD005533. doi:10.1002/14651858.CD005533.pub2.
Ebell, 2013, 2148	Ebell MH, Call M, Shinholser J. Effectiveness of oseltamivir in adults: A meta-analysis of published and unpublished clinical trials. Fam Pract. 2013;30(2):125-133. doi:10.1093/fampra/cms059.
Edmonds, 2012, 2158	Edmonds M, Milan S, Brenner B, Camargo Jr C, Rowe B. Inhaled steroids for acute asthma following emergency department discharge (Review). Cochrane Database Syst Rev. 2012;(12). doi:10.1002/14651858.CD002316.pub2.Copyright.
Edmonds, 2012, 2159	Edmonds M, Milan S, Camargo Jr C, Pollack C, Rowe B. Early use of inhaled corticosteroids in the emergency department treatment of acute asthma (Review). Cochrane Database Syst Rev. 2012;(12). doi:10.1002/14651858.CD002308.pub2.Copyright.
Ezekowitz, 2009, 2304	Ezekowitz JA, McAlister FA. Aldosterone blockade and left ventricular dysfunction: A systematic review of randomized clinical trials. Eur Heart J. 2009;30(4):469-477. doi:10.1093/eurheartj/ehn543.
Farne, 2015, 8767	Farne H, Cates C. Long-acting beta 2 -agonist in addition to tiotropium versus either tiotropium or long-acting beta 2 -agonist alone for chronic obstructive pulmonary disease. Cochrane Database Syst Rev. 2015;10(6):CD008989. doi:10.1002/14651858.CD008989.pub3.
Filippini, 2003, 2402	Filippini G, Munari L, Incorvaia B, et al. Interferons in relapsing remitting multiple sclerosis: a systematic review. Lancet. 2003;361(9357):545-552. doi:10.1016/S0140-6736(03)12512-3.
Fisher, 2014, 8769	Fisher S, Zhang H, Doree C, Mathur A, Martin-Rendon E. Stem cell treatment for acute myocardial infarction. Cochrane database Syst Rev. 2015;(9).
Fisher, 2015, 8770	Fisher SA, Doree C, Mathur A, Martin-Rendon E. Meta-analysis of cell therapy trials for patients with heart failure. Circ Res. 2015;116(8):1361-1377. doi:10.1161/CIRCRESAHA.116.304386.
Fisher, 2015, 8771	Fisher SA, Brunskill SJ, Doree C, Mathur A, Taggart DP, Martin-Rendon E. Stem cell therapy for chronic ischaemic heart disease and congestive heart failure. Cochrane Database Syst Rev. 2014;(4). doi:10.1002/14651858.CD007888.pub2.www.cochranelibrary.com.
Fox, 2011, 2496	Fox BD, Shimony A, Langleben D. Meta-analysis of monotherapy versus combination therapy for pulmonary arterial hypertension. Am J Cardiol. 2011;108(8):1177-1182. doi:10.1016/j.amjcard.2011.06.021.
Fu, 2012, 2549	Fu M, Zhou J, Sun A, et al. Efficacy of ACE inhibitors in chronic heart failure with preserved ejection fraction - A meta analysis of 7 prospective clinical studies. Int J Cardiol. 2012;155(1):33-38. doi:10.1016/j.ijcard.2011.01.081.
Gandhi, 2014, 2599	Gandhi S, Mosleh W, Myers RBH. Hypertonic saline with furosemide for the treatment of acute congestive heart failure: A systematic review and meta-analysis. Int J Cardiol. 2014;173(2):139-145. doi:10.1016/j.ijcard.2014.03.020.
Gao, 2014, 8775	Gao YH, Guan WJ, Xu G, et al. Macrolide therapy in adults and children with non-cystic fibrosis bronchiectasis: A systematic review and meta-analysis. PLoS One. 2014;9(3):1-12. doi:10.1371/journal.pone.0090047.
Garside, 2007, 2640	Garside R, Pitt M, Anderson R, et al. The Effectiveness and Cost-Effectiveness of Cinacalcet for Secondary Hyperparathyroidism in End-Stage Renal Disease Patients on Dialysis: A Systematic Review and Economic Evaluation. Vol 11.; 2007. doi:10.1038/ncpneph0039.
Grimwade, 2003, 2933	Grimwade K, Swingler. Cotrimoxazole prophylaxis for opportunistic infections in adults with HIV. Cochrane Database Syst Rev. 2003;(3):Cd003108. doi:10.1002/14651858.cd003108.
Grooten, 2015, 8780	Grooten IJ, Vinke ME, Roseboom TJ, Painter RC. A Systematic Review and Meta-Analysis of the Utility of Corticosteroids in the Treatment of Hyperemesis Gravidarum. Nutr Metab Insights. 2015;8(Suppl 1):23-32. doi:10.4137/NMI.S29532.
Hemkens, 2016, 8784	Hemkens LG, Ewald H, Gloy VL, et al. Colchicine for prevention of cardiovascular events. Cochrane database Syst Rev. 2016;(1):CD011047. doi:10.1002/14651858.CD011047.pub2.
Heran, 2012, 3275	Heran B, Musini V, Bassett K, Taylor R, Wright J. Angiotensin receptor blockers for heart failure. Cocherane Databased Syst Rev. 2012;(4). doi:10.1002/14651858.CD003040.pub2.

Hood, 2014, 3415	Hood W, Dans A, Guyatt G, Jaeschke R, McMurray J. Digitalis for treatment of heart failure in patients in sinus rhythm. Cochrane Database Syst Rev. 2014;(4).
Jaeschke, 2008, 3639	Jaeschke R, O'Byrne PM, Mejza F, et al. The safety of long-acting b-agonists among patients with asthma using inhaled corticosteroids: Systematic review and metaanalysis. Am J Respir Crit Care Med. 2008;178(10):1009-1016. doi:10.1164/rccm.200804-494OC.
Jaeschke, 2008, 3640	Jaeschke R, O'Byrne PM, Mejza F, et al. The safety of long-acting beta-agonists among patients with asthma using inhaled corticosteroids: systematic review and metaanalysis. Am J Respir Crit Care Med. 2008;178:1009-1016. doi:10.1164/rccm.200804-494OC.
James, 2008, 3657	James MT, Conley J, Tonelli M, Manns BJ, Macrae J. Meta-analysis : Antibiotics for Prophylaxis against Hemodialysis Catheter – Related Infections. Ann Intern Med. 2008;148:596-605.
Jankowska, 2016, 8790	Jankowska EA, Suchocki T, Tkaczyszyn M, et al. Effects of Intravenous Iron Therapy in Iron Deficient Patients With Systolic Heart Failure: Meta-Analysis of Randomized Control Trials. J Am Coll Cardiol. 2016;65(10):A854. doi:10.1016/S0735-1097(15)60854-3.
Jefferson, 2014, 3696	Jefferson T, Ma J, Doshi P, et al. Neuraminidase inhibitors for preventing and treating influenza in healthy adults and children (Review) Neuraminidase inhibitors for preventing and treating influenza in healthy adults and children. Cochrane Database Syst Rev. 2014;(4):4-8. doi:10.1002/14651858.CD008965.pub4.Copyright.
Jong, 2002, 3781	Jong P, Demers C, McKelvie RS, Liu PP. Angiotensin receptor blockers in heart failure: Meta-analysis of randomized controlled trials. J Am Coll Cardiol. 2002;39(3):463-470. doi:http://dx.doi.org/10.1016/S0735-1097%2801%2901775-2.
Kang, 2007, 3877	Kang S, Yang Y, CJ L, Gao R. Effectiveness and tolerability of administration of granulocyte colony-stimulating factor on left ventricular function in patients with myocardial infarction: a meta-analysis of randomized controlled trials. Clin Ther. 2007;29(11):2406-2418 13p. http://proxy.lib.sfu.ca/login?url=http://search.ebscohost.com/login.aspx?direct=true&db=ccm&AN=105856986&site=ehost-live.
Kang, 2008, 3879	Kang S, Yang Y-J, Li C-J, Gao R-L. Effects of intracoronary autologous bone marrow cells on left ventricular function in acute myocardial infarction: a systematic review and meta-analysis for randomized controlled trials. Coron Artery Dis. 2008;19(5):327-335. doi:10.1097/MCA.0b013e328300dbd3.
Kansagara, 2013, 3885	Kansagara D, Dyer E, Englander H, Fu R, Freeman M, Kagen D. Treatment of Anemia in Patients With Heart Disease. Ann Intern Med. 2013.
Karner, 2011, 3908	Karner C, Cates C. Combination inhaled steroid and long-acting beta2-agonist in addition to tiotropium versus tiotropium or combination alone for chronic obstructive pulmonary disease (Review). Cochrane Database Syst Rev. 2011;(3).
Karner, 2014, 8692	Karner C, Chong J, Poole P. Tiotropium versus placebo for chronic obstructive pulmonary disease. Cochrane Database Syst Rev. 2014;(7). doi:10.1002/14651858.CD009285.pub3.
Kaur, 2014, 3937	Kaur N, Pandey A, Negi H, et al. Effect of HDL-raising drugs on cardiovascular outcomes: A systematic review and meta-regression. PLoS One. 2014;9(4):1-11. doi:10.1371/journal.pone.0094585.
Kew, 2014, 4002	Kew KM, Kirtchuk L, Michell CI. Intravenous magnesium sulfate for treating adults with acute asthma in the emergency department. Cochrane Database Syst Rev. 2014;(5).
Kew, 2013, 4003	Kew KM, Mavergames C, Walters JA. Long-acting beta 2 -agonists for chronic obstructive pulmonary disease. Cochrane Database Syst Rev. 2013;(10). doi:10.1002/14651858.CD010177.pub2.
Kew, 2014, 4004	Kew KM, Seniukovich A. Inhaled steroids and risk of pneumonia for chronic obstructive pulmonary disease. Cochrane Database Syst Rev. 2014;(12). http://ovidsp.ovid.com/ovidweb.cgi?T=JS&PAGE=reference&D=coch&NEWS=N&AN=00075320-100000000-08440.
Kew, 2016, 8802	Kew K, Dahri K. Long-acting muscarinic antagonists (LAMA) added to combination long-acting beta 2 -agonists and inhaled corticosteroids (LABA / ICS) versus LABA / ICS for adults with asthma (Review) SUMMARY OF FINDINGS FOR THE MAIN COMPARISON. Cochrane Database Syst Rev. 2016;(1). doi:10.1002/14651858.CD011721.pub2.www.cochranelibrary.com.
Kew, 2015, 8803	Kew K, Evans D, Allison D, Boyter A. Long-acting muscarinic antagonists (LAMA) added to inhaled corticosteroids (ICS) versus addition of long-acting beta 2 - agonists (LABA) for adults with asthma (Review). Cochrane Database Syst Rev. 2015;(6). doi:10.1002/14651858.CD011438.pub2.www.cochranelibrary.com.
Kew, 2015, 8805	Kew K, Undela K, Kotortsi I, Ferrara G. Macrolides for chronic asthma (Review). Cochrane Libr. 2015;(3). doi:10.1002/14651858.CD002997.pub4.www.cochranelibrary.com.
Kishimoto, 2013, 4065	Kishimoto T, Agarwal V, Kishi T, Leucht S, Kane JM, Correll CU. Relapse prevention in schizophrenia: a systematic review and meta-analysis of second-generation antipsychotics versus first-generation antipsychotics. Mol Psychiatry. 2013;18(1):53-66. doi:10.1038/mp.2011.143.
Kishimoto, 2014, 4067	Kishimoto T, Robenzadeh A, Leucht C, et al. Long-acting injectable vs oral antipsychotics for relapse prevention in schizophrenia: A meta-analysis of randomized trials. Schizophr Bull. 2014;40(1):192-213. doi:10.1093/schbul/sbs150.
Komossa, 2010, 4123	Komossa K, Hunger H, Schmid F, et al. Olanzapine versus other atypical antipsychotics for schizophrenia (Review) Olanzapine versus other atypical antipsychotics for schizophrenia. Cochrane Database Syst Rev. 2010;(3). doi:10.1002/14651858.CD006654.pub2.Copyright.
Kuenzli, 2010, 4206	Kuenzli A, Bucher HC, Anand I, et al. Meta-analysis of combined therapy with angiotensin receptor antagonists versus ACE inhibitors alone in patients with heart failure. PLoS One. 2010;5(4). doi:10.1371/journal.pone.0009946.
Kumar, 2014, 8815	Kumar G, Stendall C, Mistry R, Gurusamy K, Walker D. A comparison of total intravenous anaesthesia using propofol with sevoflurane or desflurane in ambulatory surgery: Systematic review and meta-analysis. Anaesthesia. 2014;69(10):1138-1150. doi:10.1111/anae.12713.
Kuswardhani, 2011, 4239	Kuswardhani RA, Soejitno A. Bone marrow-derived stem cells as an adjunctive treatment for acute myocardial infarction: a systematic review and meta-analysis. Acta Med Indones. 2011;43(3):168-177. http://www.ncbi.nlm.nih.gov/pubmed/21979282.
La Mantia, 2010, 4260	La Mantia L, Munari L, Lovati R. Glatiramer acetate for multiple sclerosis. Cochrane Database Syst Rev. 2010;(5):N.PAG-N.PAG. doi:10.1002/14651858.CD004678.

Lasserson, 2011, 4362	Lasserson TJ, Cates CJ, Ferrara G. G, Casali L. L. Combination fluticasone and salmeterol versus fixed dose combination budesonide and formoterol for chronic asthma in adults and children. Cochrane Database Syst Rev. 2011;(12). doi:10.1002/14651858.CD004106.pub3.
Le, 2016, 8817	Le H-H, El-Khatib C, Mombled M, et al. Impact of Aldosterone Antagonists on Sudden Cardiac Death Prevention in Heart Failure and Post-Myocardial Infarction Patients: A Systematic Review and Meta-Analysis of Randomized Controlled Trials. PLoS One. 2016;11(2):e0145958. doi:10.1371/journal.pone.0145958.
Leucht, 2011, 4505	Leucht C, Heres S, Kane JM, Kissling W, Davis JM, Leucht S. Oral versus depot antipsychotic drugs for schizophrenia-A critical systematic review and meta-analysis of randomised long-term trials. Schizophr Res. 2011;127(1-3):83-92. doi:10.1016/j.schres.2010.11.020.
Leucht, 2012, 4508	Leucht S, Tardy M, Komossa K, et al. Maintenance treatment with antipsychotic drugs for schizophrenia (Review) Maintenance treatment with antipsychotic drugs for schizophrenia. Cochrane Database Syst Rev. 2012;(5):10-12. doi:10.1002/14651858.CD008016.pub2.Copyright.
Levy, 2010, 4518	Levy BF, Tilney HS, Dowson HMP, Rockall TA. A systematic review of postoperative analgesia following laparoscopic colorectal surgery. Color Dis. 2010;12(1):5-15. doi:10.1111/j.1463-1318.2009.01799.x.
Li, 2016, 8822	Li L, Li S, Liu J, et al. Glucagon-like peptide-1 receptor agonists and heart failure in type 2 diabetes: systematic review and meta-analysis of randomized and observational studies. BMC Cardiovasc Disord. 2016;16(1):91. doi:10.1186/s12872-016-0260-0.
Liew, 2014, 4577	Liew AY, Eikelboom JW, Connolly SJ, M OD, Hart RG. Efficacy and safety of warfarin vs. antiplatelet therapy in patients with systolic heart failure and sinus rhythm: a systematic review and meta-analysis of randomized controlled trials. Int J Stroke. 2014;9(2):199-206. doi:http://dx.doi.org/10.1111/ijs.12036.
Lipinski, 2009, 4615	Lipinski MJ, Cauthen CA, Biondi-Zoccai GGL, et al. Meta-Analysis of Randomized Controlled Trials of Statins Versus Placebo in Patients With Heart Failure. Am J Cardiol. 2009;104(12):1708-1716. doi:10.1016/j.amjcard.2009.07.055.
Liu, 2014, 4629	Liu F, Chen Y, Feng X, Teng Z, Yuan Y, Bin J. Effects of beta-blockers on heart failure with preserved ejection fraction: A meta-analysis. PLoS One. 2014;9(3). doi:10.1371/journal.pone.0090555.
Liu, 2014, 8826	Liu G, Zheng XX, Xu YL, Lu J, Hui RT, Huang XH. Effects of lipophilic statins for heart failure: A meta-analysis of 13 randomised controlled trials. Hear Lung Circ. 2014;23(10):970-977. doi:10.1016/j.hlc.2014.05.005.
Liu, 2014, 8827	Liu X, Wang D, Zheng L, Mou T, Liu H, Li G. Is Early Oral Feeding after Gastric Cancer Surgery Feasible ? A Systematic Review and Meta-Analysis of Randomized Controlled Trials. PLoS One. 2014;9(11). doi:10.1371/journal.pone.0112062.
Lopez, 2015, 8829	Lopez A, Ford A, Colombel J, Reinisch W, Sandborn W, Peyrin-Biroulet L. Efficacy of tumour necrosis factor antagonists on remission, colectomy and hospitalisations in ulcerative colitis: Meta-analysis of placebo-controlled trials. Dig Liver Dis. 2015;47(5):356-364. doi:10.1016/j.dld.2015.01.148.
Magee, 2003, 4812	Magee L, Duley L. Oral beta-blockers for mild to moderate hypertension during pregnancy (Review). Cochrane Database Syst Rev. 2003;(3).
Magee, 2000, 4815	Magee LA, Elran E, Bull SB, Logan A, Koren G. Risks and benefits of b-receptor blockers for pregnancy hypertension: Overview of the randomized trials. Eur J Obstet Gynecol Reprod Biol. 2000;88(1):15-26. doi:10.1016/S0301-2115(99)00113-X.
Makani, 2013, 4838	Makani H, Bangalore S, Desouza K a, Shah A, Messerli FH. Efficacy and safety of dual blockade of the renin-angiotensin system: meta-analysis of randomised trials. BMJ. 2013;346(January):f360. doi:10.1136/bmj.f360.
Makrides, 2014, 4844	Makrides M, Crowther CA. Magnesium supplementation in pregnancy. Cochrane database Syst Rev. 2014;(4):CD000937. doi:10.1002/14651858.CD000937.
Mannucci, 2008, 4889	Mannucci E, Monami M, Lamanna C, Gensini GF, Marchionni N. Pioglitazone and cardiovascular risk. A comprehensive meta-analysis of randomized clinical trials. Diabetes Obes Metab. 2008;10(12):1221-1238. doi:10.1111/j.1463-1326.2008.00892.x.
Medic, 2016, 8842	Medic G, Lindner L, van der Weijden M, Karabis A. Efficacy and Safety of Aclidinium/Formoterol versus Tiotropium in COPD: Results of an Indirect Treatment Comparison. Adv Ther. 2016;33(3):379-399. doi:10.1007/s12325-016-0299-4.
Mohammed, 2007, 5325	Mohammed S, Goodacre S. Intravenous and nebulised magnesium sulphate for acute asthma: systematic review and meta-analysis. Emerg Med J. 2007;24(12):823-830. doi:10.1136/emj.2007.052050.
Møiniche, 2003, 5330	Møiniche S, Rømsing J, Dahl JB, Tramèr MR. Nonsteroidal antiinflammatory drugs and the risk of operative site bleeding after tonsillectomy: a quantitative systematic review. Anesth Analg. 2003;96(1):68-77, table of contents. doi:10.1213/01.ANE.0000040583.65135.57.
Moore, 2006, 5371	Moore RA, Derry S. Systematic review and meta-analysis of randomised trials and cohort studies of mycophenolate mofetil in lupus nephritis. Arthritis Res Ther. 2006;8(6):R182. doi:10.1186/ar2093.
Nair, 2012, 5515	Nair P, Milan SJ, Rowe BH. Addition of intravenous aminophylline to inhaled beta2-agonists in adults with acute asthma. Cochrane Database Syst Rev. 2012;12(4). doi:10.1002/14651858.CD002742.pub2.Copyright.
Nannini, 2013, 5533	Nannini L, Poole P, Milan S, Kesterton A. Combined corticosteroid and long-acting beta2 -agonist in one inhaler versus inhaled corticosteroids alone for chronic obstructive pulmonary disease (Review). Cochrane Database Syst Rev. 2013;(8).
Nannini, 2013, 8694	Nannini L, Poole P, Sj M, Holmes R, Normansell R. Combined corticosteroid and long-acting beta2-agonist in one inhaler versus placebo for chronic obstructive pulmonary disease (Review). Cochrane Database Syst Rev. 2013;(11):CD003794.
Ngo, 2010, 5606	Ngo K, Kotecha D, Walters J, et al. Erythropoiesis-stimulating agents for anaemia in chronic heart failure patients. Cocherane Databased Syst Rev. 2010;(1).
Ni, 2009, 5611	Ni, Muireann, Greenstone I, Lasserson TJ, Ducharme FM. Addition of inhaled long-acting beta2-agonists to inhaled steroids as first line therapy for persistent asthma in steroid-naive adults and children. Cochrane database Syst Rev. 2009;(4):CD005307. doi:10.1002/14651858.CD005307.pub2.

Ni, 2014, 8850	Ni H, Soe Z, Moe S. Cochrane Database of Systematic Reviews Aclidinium bromide for stable chronic obstructive pulmonary disease (Review) Aclidinium bromide for stable chronic obstructive pulmonary disease (Review). Cochrane Database Syst Rev. 2014;(9). doi:10.1002/14651858.CD010509.pub2.
Ni, 2015, 9197	Ni W, Shao X, Cai X, et al. Prophylactic use of macrolide antibiotics for the prevention of chronic obstructive pulmonary disease exacerbation: A meta-analysis. PLoS One. 2015;10(3):1-13. doi:10.1371/journal.pone.0121257.
Pizzi, 2011, 6161	Pizzi C, Rutjes AWS, Costa GM, Fontana F, Mezzetti A, Manzoli L. Meta-analysis of selective serotonin reuptake inhibitors in patients with depression and coronary heart disease. Am J Cardiol. 2011;107(7):972-979. doi:10.1016/j.amjcard.2010.11.017.
Poole, 2015, 8867	Poole P, Chong J, Cates C. Mucolytic agents versus placebo for chronic bronchitis or chronic obstructive pulmonary disease. Cochrane Database Syst Rev. 2015;(7). doi:10.1002/14651858.CD001287.pub5.
Powell, 2015, 8868	Powell C, Sj M, Dwan K, Bax L, Walters N. Mepolizumab versus placebo for asthma (Review). Cochrane Database Syst Rev. 2015;(7). doi:10.1002/14651858.CD010834.pub2.www.cochranelibrary.com.
Rajagopalan, 2011, 6347	Rajagopalan S, Arora A, Shafiq N, et al. Pharmacotherapy of heart failure with normal ejection fraction (HFNEF) - a systematic review. Br J Clin Pharmacol. 2011;72(3):369-380. doi:10.1111/j.1365-2125.2011.03998.x.
Renner, 2012, 6442	Renner P, Milazzo S, Liu JP, Zwahlen M, Birkmann J, Horneber M. Primary prophylactic colony-stimulating factors for the prevention of chemotherapy-induced febrile neutropenia in breast cancer patients. Cochrane Database Syst Rev. 2012;(10). doi:10.1002/14651858.CD007913.pub2.
Rodrigo, 1999, 6564	Rodrigo GJ, Castro-Rodriguez JA. Anticholinergics in the treatment of children and adults with acute asthma: a systematic review with meta-analysis. Thorax. 2005;65(12):1118-1118. doi:10.1136/thx.2005.040444.
Rodrigo, 2000, 6566	Rodrigo G, Rodrigo C, Burschtin O. A Meta-analysis of the effects of ipratropium bromide in adults with acute asthma. J Med. 1999;108(7):596-597. http://ovidsp.ovid.com/ovidweb.cgi?T=JS&CSC=Y&NEWS=N&PAGE=fulltext&D=med4&AN=10866596.
Rodrigo, 2005, 6568	Rodrigo GJ, Nannini LJ. Tiotropium for the treatment of stable chronic obstructive pulmonary disease: a systematic review with meta-analysis. Pulm Pharmacol Ther. 2007;20(5):495-502. doi:10.1016/j.pupt.2006.02.003.
Rodrigo, 2002, 6576	Rodrigo GJ, Rodrigo C, Pollack C V, Rowe B. Use of Helium-Oxygen Mixtures in the Treatment of Acute Asthma: A Systematic Review Use of Helium-Oxygen Mixtures in the Treatment of Acute Asthma *. Chest. 2003:891-896. doi:10.1378/chest.123.3.891.
Rodrigo, 2003, 6578	Rodrigo G, Rodrigo C. Corticosteroids in the Emergency Department Therapy of Acute Adult Asthma*. Chest. 1999;116(2):285-295. doi:10.1378/chest.116.2.285.
Rowe, 2000, 6670	Rowe BH, Bretzlaff JA, Bourdon C, Bota GW, Camargo Jr. CA. Intravenous magnesium sulfate treatment for acute asthma in the emergency department: a systematic review of the literature. Ann Emerg Med. 2000;36(3):181-190. doi:10.1067/mem.2000.105659.
Rowe, 1992, 6672	Rowe BH, Keller JL, Oxman AD. Effectiveness of steroid therapy in acute exacerbations of asthma: A meta- analysis. Am J Emerg Med. 1992;10(4):301-310. http://www.embase.com/search/results?subaction=viewrecord&from=export&id=L22241536%5Cnhttp://dx.doi.org/10.1016/0735-6757(92)90007-K.
Rowe, 2001, 6676	Rowe BH, Spooner CH, Ducharme FM, Bretzlaff JA, Bota GW. Corticosteroids for preventing relapse following acute exacerbations of asthma. Cochrane Database Syst Rev. 2007;(2):Cd000195.
Rowe, 2007, 6677	Rowe B, Spooner C, Ducharme F, Bretzlaff F, Bota G. Early emergency department treatment of acute asthma with systemic corticosteroids. Cochrane Database Syst Rev. 2001;(2):Cd002178.
Saab, 2015, 8884	Saab S, Suraweera D, Au J, Saab EG, Alper TS, Tong MJ. Probiotics are helpful in hepatic encephalopathy: a meta-analysis of randomized trials. Liver Int. 2015;36(7):986-993. doi:10.1111/liv.13005.
Saha, 2007, 6746	Saha S a, Molnar J, Arora RR. Tissue angiotensin-converting enzyme inhibitors for the prevention of cardiovascular disease in patients with diabetes mellitus without left ventricular systolic dysfunction or clinical evidence of heart failure: a pooled meta-analysis of randomized place. Diabetes Obes Metab. 2008;10(1):41-52. doi:10.1111/j.1463-1326.2006.00688.x.
Saha , 2008, 6747	Saha S a, Molnar J, Arora RR. Tissue ACE inhibitors for secondary prevention of cardiovascular disease in patients with preserved left ventricular function: a pooled meta-analysis of randomized placebo-controlled trials. J Cardiovasc Pharmacol Ther. 2007;12(3):192-204. doi:10.1177/1074248407304791.
Salpeter, 2006, 6795	Salpeter SR, Buckley NS, Salpeter EE. Meta-analysis: Anticholinergics, but not β-agonists, reduce severe exacerbations and respiratory mortality in COPD. J Gen Intern Med. 2006;21(10):1011-1019. doi:10.1111/j.1525-1497.2006.00507.x.
Salpeter, 2006, 6797	Salpeter S, Buckley N, Ormiston T, Salpeter E. Meta-Analysis: Effect of Long-Acting B-Agonists on Severe Asthma Exacerbations and Asthma-Related Deaths. Ann Intern Med. 2006;(9):350-358.
Sampson, 2013, 6806	Sampson S, Joshi K, Mansour M, Adams CE. Intermittent drug techniques for schizophrenia. Schizophr Bull. 2013;39(5):960-961. doi:10.1093/schbul/sbt096.
Shan, 2013, 7029	Shan Z, Rong Y, Yang W, et al. Intravenous and nebulized magnesium sulfate for treating acute asthma in adults and children: A systematic review and meta-analysis. Respir Med. 2013;107(3):321-330. doi:10.1016/j.rmed.2012.12.001.
Stratton, 2013, 7484	Stratton RJ, Hébuterne X, Elia M. A systematic review and meta-analysis of the impact of oral nutritional supplements on hospital readmissions. Ageing Res Rev. 2013;12(4):884-897. doi:10.1016/j.arr.2013.07.002.
Su, 2014, 7504	Su Q, Li L, Naing KA, Sun Y. Safety and Effectiveness of Nitroprusside in Preventing No-Reflow During Percutaneous Coronary Intervention: A Systematic Review. Cell Biochem Biophys. 2014;68(1):201-206. doi:10.1007/s12013-013-9690-9.
Susantitaphon g, 2013, 7543	Susantitaphong P, Sewaralthahab K, Balk EM, Eiam-Ong S, Madias NE, Jaber BL. Efficacy and safety of combined vs. Single renin-angiotensin-aldosterone system blockade in chronic kidney disease: A meta-analysis. Am J Hypertens. 2013;26(3):424-441. doi:10.1093/ajh/hps038.

Taylor, 2001, 7647	Taylor FC, Cohen H, Ebrahim S. Systematic review of long term anticoagulation or antiplatelet treatment in patients with non-rheumatic atrial fibrillation.[Erratum appears in BMJ. 2001 Mar 10;322(7286):587]. BMJ. 2001;322(7282):321-326. http://ovidsp.ovid.com/ovidweb.cgi?T=JS&CSC=Y&NEWS=N&PAGE=fulltext&D=med4&AN=11159653.
Tonelli, 2008, 7787	Tonelli M, Klarenbach S, Wiebe N, Shrive F, Hemmelgarn B, Manns B. Erythropoiesis-Stimulating Agents for Anemia of Chronic Kidney Disease: Systematic Review and Economic Evaluation [Technology Report Number 106]. Ottawa; 2008.
Walters, 2014, 8704	Walters J a, Gibson PG, Wood-Baker R, Hannay M, Walters EH. Systemic corticosteroids for acute exacerbations of chronic obstructive pulmonary disease. Cochrane Database Syst Rev. 2014;(4):CD001288 ST-Systemic corticosteroids for acute. doi:10.1002/14651858.CD001288.pub3 [doi] ET - 2009/01/23.
Wilt, 2007, 8367	Wilt T, Niewoehner D, MacDonald R, RL K. Clinical guidelines. Management of stable chronic obstructive pulmonary disease: a systematic review for a clinical practice guideline. Ann Intern Med. 2007;147(9):639-I41 16p. http://search.ebscohost.com/login.aspx?direct=true&db=ccm&AN=105932449&site=ehost-live.
Xie, 2016, 8916	Xie W, Zheng F, Song X, Zhong B, Yan L. Renin-angiotensin-aldosterone system blockers for heart failure with reduced ejection fraction or left ventricular dysfunction: Network meta-analysis. Int J Cardiol. 2016;205:65-71. doi:10.1016/j.ijcard.2015.12.010.
Yang, 2015, 8918	Yang L, Gao L, Chen Y, et al. Prophylactic Antibiotics in Prostate Biopsy: A Meta-Analysis Based on Randomized Controlled Trials. Surg Infect (Larchmt). 2015;16(6):733-747. doi:10.1089/sur.2015.040.
Yohannes, 2011, 8511	Yohannes AM, Willgoss TG, Vestbo J. Tiotropium for treatment of stable COPD: a meta-analysis of clinically relevant outcomes. Respir Care. 2011;56:477-487. doi:10.4187/respcare.00852.
Zhang, 2011, 8604	Zhang PH, Jia CE, Lv Y, Gibson PG, Wang G. Montelukast for prevention and treatment of asthma exacerbations in adults: systematic review and meta-analysis. Allergy Asthma Proc. 2014;35(4):278-287.
Zhang, 2016, 8922	Zhang Q, Chen Y, Liu Q, Shan Q. Effects of renin–angiotensin–aldosterone system inhibitors on mortality, hospitalization, and diastolic function in patients with HFpEF: A meta-analysis of 13 randomized controlled trials. Herz. 2016;41(1):76-86. doi:10.1007/s00059-015-4346-8.
Zhang, 2014, 9143	Zhang S, Zhang L, Sun A, Jiang H, Qian J, Ge J. Efficacy of statin therapy in chronic systolic cardiac insufficiency: A meta-analysis. Eur J Intern Med. 2011;22(5):478-484. doi:10.1016/j.ejim.2011.06.003.
Zhou, 2014, 8928	Zhou X, Chen J. Is treatment with trimetazidine beneficial in patients with chronic heart failure? PLoS One. 2014;9(5). doi:10.1371/journal.pone.0094660.
Ziff, 2015, 8931	Ziff OJ, Lane DA, Samra M, et al. Safety and efficacy of digoxin: systematic review and meta-analysis of observational and controlled trial data. BMJ. 2015;351:h4451. doi:10.1136/bmj.h4451.
Zou, 2016, 8933	Zou Y, Xiao J, Yang DH, Li J, Chen Q. Efficacy and Safety of an Aclidinium Bromide Treatment for 12 Weeks or Longer in Patients with Moderate-To-Severe COPD: A Meta-Analysis. COPD J Chronic Obstr Pulm Dis. 2016;2555(April):1-10. doi:10.3109/15412555.2015.1074172.

Table S4. Information about included reviews

Author, Year, Review ID	Objective	Disease description	Intervention vs. comparison	No. of RCTs (patients)	Admissions were a primary outcome	AMSTAR score (/11)	Abstract conclusion
Afilalo, 2007, 100	We sought to determine the effect of intensive statin therapy on all-cause mortality compared with moderate statin therapy in patients with recent acute coronary syndromes and in patients with stable coronary heart disease. Secondarily, we examined the effects of intensive statin therapy on major adverse cardiovascular events admissions to hospital for heart failure, and adverse hepatic and muscular events.	Coronary artery disease	Intensive statin therapy vs. Moderate statin therapy	Total: 6 (28505) Admissions data: 4 (27548)	No	6	Compared with moderate statin therapy, intensive statin therapy reduces all-cause mortality in patients with recent ACS but not in patients with stable CHD.
Ahmed, 2014, 129	Our objectives were to conduct a systematic review and to grade the quality of evidence to ascertain the effect of influenza vaccination of healthcare personnel on morbidity and mortality in patients of healthcare facilities.	Patients in healthcare facilities	Influenza vaccination vs. Control	Total: 4 (8468) Admissions data: 2 (5972)	Yes	6	The quality of evidence is higher for mortality than for other outcomes. HCP influenza vaccination can enhance patient safety.
Akioyamen, 2016, 8711	The purpose of this study was to conduct a systematic review and meta-analysis assessing the long-term effects of ARBs as a class on BP control, myocardial infarction, hospitalization for heart failure, cerebrovascular events (ie, stroke), cardiovascular mortality, and all-cause mortality.	Essential hypertension	Long-term angiotensin receptor blockade vs. Placebo or placebo with second-line non-ARB antihypertensive therapy permitted	Total: 7 (16864) Admissions data: 2 (6611)	No	7	Our findings suggest that ARBs, as a class, are more effective than placebo therapy in long-term BP lowering in patients with essential hypertension. Long-term ARB treatment may also confer enhanced protection against stroke but not other cardiovascular outcomes relative to placebo. J Am Soc Hypertens 2016;10(1):55–69. 2016 American Society of Hypertension.
Anderson, 2015, 8714	To assess the efficacy and safety of a long-acting muscarinic antagonist (LAMA) added to any dose of an inhaled corticosteroid (ICS) compared with the same dose of ICS alone for adults whose asthma is not well controlled.	Asthma	Long-acting muscarinic antagonists (LAMA) added to inhaled corticosteroids (ICS) vs. Same dose inhaled corticosteriods	Total: 5 (2563) Admissions data: 5 (2562)	No	11	For adults taking ICS for asthma without a long-acting beta - agonist (LABA), LAMA given as add-on treatment reduces the likelihood of exacerbations requiring treatment with OCS and improves lung function. The benefits of LAMA combined with ICS for hospital admissions, all-cause serious adverse events, quality of life and asthma control remain unknown. Results of this review, along with findings of related reviews conducted to assess the use of LAMA in other clinical scenarios involving asthma, can help to define the role of LAMA in the management of asthma. Trials of longer duration (up to 52 weeks) would provide a better opportunity to observe rare events such as serious adverse events and exacerbations requiring hospital admission.
Assasi, 2009, 378	The aim of this HTA is to evaluate the comparative clinical-effectiveness of anti-TNF-α drugs in patients with CD or UC with an inadequate response to conventional therapy and to determine the economic value of anti-TNF-α drugs compared with that of conventional therapy and surgical interventions.	Crohn's Disease, Ulcerative Colitis	Anti-tumor necrosis factor alpha drugs (Infliximab or Adalimumab) vs. Intra-class drug comparison; inter-class comparison of conventional therap; immunosuppressant drugs; or surgical interventions	Total: 20 (3132) Admissions data: 4 (1137)	No	9	Although infliximab and adalimumab have been shown to provide clinical benefit, the costs associated with these treatments could be perceived as high. Based on the incremental cost-utility findings from our primary economic evaluations, adalimumab and infliximab for the treatment of IBD may not be perceived to be a cost-effective use of health care resources.

Badve, 2011, 446	The aim of this systematic review was to study the benefits and risks of beta-adrenergic antagonists (betablockers) in patients with chronic kidney disease (CKD).	Chronic Kidney Disease	Beta-blocker vs. Placebo	Total: 8 (6949) Admissions data: Unclear (Unclear)	No	5	Treatment with beta-blockers improved all-cause mortality in patients with CKD and chronic systolic heart failure. There is insufficient evidence to conclude whether people with CKD who are not known to have heart failure derive benefit from beta-blockers.
Baigent , 2013, 1653	The main objective was to characterise and quantify the cardiovascular and gastrointestinal risks of particular NSAID regimens among diff erent types of patients, particularly those at increased risk of vascular disease.	Patients at increased risk of vascular disease	Coxib vs. Placebo or another NSAID (diclofenac; ibuprofen; naproxen)	Total: 634 (192981) Admissions data: Unclear (Unclear)	Yes	6	The vascular risks of high-dose diclofenac, and possibly ibuprofen, are comparable to coxibs, whereas high-dose naproxen is associated with less vascular risk than other NSAIDs. Although NSAIDs increase vascular and gastrointestinal risks, the size of these risks can be predicted, which could help guide clinical decision making.
Barr, 2006, 548	To determine the efficacy of tiotropium on clinical endpoints such exacerbations and hospitalisations, symptom scales and pulmonary function compared to placebo and other bronchodilators used for stable COPD.	Chronic Obstructive Pulmonary Disease	Tiotropium vs. Placebo; ipratropium bromide; long-acting ß2-agonists (salmeterol or formoterol)	Total: 9 (6584) Admissions data: 4 (4087)	Yes	10	Tiotropium reduced COPD exacerbations and related hospitalisations compared to placebo and ipratropium. It also improved healthrelated quality-of-life and symptom scores among patients with moderate and severe disease, and may have slowed decline in FEV1. Additional long-term studies are required to evaluate its effect on mortality and change in FEV1 to clarify its role in comparison to, or in combination with, long-acting ß2-agonists and to assess its effectiveness in mild and very severe COPD.
Baumeister , 2011, 593	To determine the effects of psychological and pharmacological interventions for depression in coronary artery disease patients with comorbid depressive disorder.	Coronary artery disease and comorbid depressive disorder	Pharmacological intervention vs. Placebo; no intervention; usual care; other pharmacological medications	Total: 16 (unclear) Admissions data: 4 (2995)	No	11	Psychological interventions and pharmacological interventionswith SSRIsmay have a small yet clinicallymeaningful effect on depression outcomes in CAD patients. No beneficial effects on the reduction of mortality rates and cardiac events were found. Overall, however, the evidence is sparse due to the low number of high quality trials per outcome and the heterogeneity of examined populations and interventions.
Beck, 2013, 615	The purpose of this paper was to estimate the effectiveness of oral nutritional support compared with placebo or usual care in improving re-admissions, survival, nutritional status, functional status, quality of life and morbidity of older (65 years+) medical and surgical patients after discharge from hospital.	Older (65+) patients being discharged from the hospital to the community that are malnourished or at risk of malnourishment	Oral nutritional support vs. Placebo; usual care; standard care	Total: 6 (716) Admissions data: 4 (478)	Yes	5	Although the evidence is limited, we suggest that oral nutritional support may be considered for older malnourished medical and surgical patients after discharge from hospital.
Blitz, 2005, 813	A systematic review of the literature was performed to examine the effect of inhaled MgSO4 in the treatment of patients with asthma exacerbations in the emergency department.	Asthma	Nebulized magnesium sulfate vs. B2-agonist alone	Total: 6 (296) Admissions data: 1 (52)	No	5	The use of nebulized MgSO4, particularly in addition to a 2-agonist, in the treatment of an acute asthma exacerbation appears to produce benefits with respect to improved pulmonary function and may reduce the number of hospital admissions.
Bonsu, 2016, 8729	This study aims to compare lipophilic and hydrophilic statin therapy on clinical outcomes of heart failure (HF) using a systematic review and an adjusted indirect comparison meta-analysis.	Heart failure	Lipophilic Statin vs. Placebo	Total: 13 (10966) Admissions data: 10 (10665)	Yes	9	Lipophilic statin treatment shows significant decreases in all-cause mortality, cardiovascular mortality and hospitalization for worsening HF compared with rosuvastatin treatment. This meta-analysis provides preliminary evidence that lipophilic statins offer better clinical outcomes in HF till data from head to head comparisons are available.
Briasoulis, 2015, 8731	The present meta-analysis was designed to systematically evaluate prospective controlled trials and observational cohorts and assess the effects of carvedilol versus metoprolol types (succinate and tartrate) on all-cause mortality and rehospitalization.	Heart failure	Carvedilol vs. Metoprolol	Total: 4 (4776) Admissions data: 2 (4544)	No	6	Neither all-cause mortality nor hospitalizations were significantly different between carvedilol and metoprolol succinate in the cohort studies. In conclusion, in patients with HFrEF, carvedilol and metoprolol succinate have similar effects in reducing all-cause mortality.

Brophy, 2001, 1021	Congestive heart failure is an important cause of patient morbidity and mortality. Although several randomized clinical trials have compared b-blockers with placebo for treatment of congestive heart failure, a meta-analysis quantifying the effect on mortality and morbidity has not been performed recently.	Congestive Heart Failure	Beta-blocker vs. Placebo	Total: 22 (10132) Admissions data: 22 (10076)	No	4	B-Blocker therapy is associated with clinically meaningful reductions in mortality and morbidity in patients withstable congestive heart failure and should be routinely offered to all patients similar to those included in trials.
Burch, 2009, 1080	The objective of this review is to evaluate the clinical effectiveness (including adverse events) and cost-effectiveness of antivirals for the treatment of naturally acquired influenza. This evaluation considers these issues for at-risk and otherwise healthy populations. It is important to note that this health technology assessment was carried out to address the use of antiviral treatments for influenza within the context of a seasonal outbreak, not a pandemic.	Influenza	Zanamivir or oseltamivir vs. Placebo; antiviral drugs for treatment licensed in the UK (intravenous and nebulised zanamivir excluded); best symptomatic care.	Total: 34 (unclear) Admissions data: 14 (5694)	No	8	The clinical effectiveness data for population subgroups, used to inform the multiparameter evidence synthesis and cost-effectiveness modelling were, in places, limited and this should be borne in mind when interpreting the findings of this review. Trials were often not designed to determine clinical effectiveness in population subgroups and hence, although the direction of effect was clear, estimates of differences in symptom duration tended to be subject to greater uncertainty in subgroups. This limitation was more apparent for data on the rates of complications: studies with sample size and duration not designed to detect these outcomes resulted in low event rates and relatively weak evidence, even when available data were combined in meta-analyses. However, despite these concerns, the use of NIs in at-risk populations appeared to be a cost-effective approach to the treatment of influenza.
Cammarano, 2016, 8735	The goal of this review was to pool data from ivabradine studies in all patients with stable CAD to compare cardiovascular and safety-related outcomes.	Stable coronary artery disease with and without left ventricular dysfunction	Ivabradine vs. Placebo	Total: 3 (36524) Admissions data: 3 (36524)	Yes	4	Unselective use of ivabradine in patients with stable CAD is not supported by evidence and can be associated with new-onset atrial fibrillation, bradycardia,and drug-related nuisance adverse events.
Campschroer, 2014, 1164	This review aimed to answer the following question: does medical treatment with alpha-blockers compared to other pharmacotherapy or placebo impact on stone clearance rate, in adult patients presenting with symptoms of ureteral stones less than 10 mm confirmed by imaging? Other clinically relevant outcomes such as stone expulsion time, hospitalisation, pain scores, analgesic use and adverse effects have also been explored.	Ureteral stones	Alpha-blocker vs. Any other pharmacotherapy including: standard therapy (e.g. NSAIDs, corticosteroids); calcium channel blockers; placebo.	Total: 30 (6155) Admissions data: 4 (313)	No	10	The use of alpha-blockers in patients with ureteral stones results in a higher stone-free rate and a shorter time to stone expulsion. Alphablockersshould therefore be offered as part of medical expulsive therapy as one of the primary treatment modalities.
Cates, 2013, 1249	To assess the effects of holding chambers (spacers) compared to nebulisers for the delivery of beta - agonists for acute asthma.	Asthma	Treatment with beta2-agonists via Spacer (chamber) vs. Treatment with beta2-agonists via nebuliser (multiple-treatment studies)	Total: 39 (2626) Admissions data: 9 (582)	Yes	9	Nebuliser delivery produced outcomes that were not significantly better than metered-dose inhalers delivered by spacer in adults or children, in trials where treatments were repeated and titrated to the response of the participant. Spacers may have some advantages compared to nebulisers for children with acute asthma. The studies excluded people with life-threatening asthma; therefore, the results of this meta-analysis should not be extrapolated to this patient population.
Cawood, 2012, 1264	Therefore, this systematic review was undertaken to examine whether high protein ONS have beneficial effects in clinical practice and the extent to which these are associated with increased protein intake.	Patients in hospital or community settings that are malnourished or at risk of disease-related malnutrition	High protein oral nutritional supplements vs. Placebo or usual care	Total: 36 (3790) Admissions data: 2 (525)	No	7	The systematic review and meta-analysis provides evidence that high protein supplements produce clinical benefits, with economic implications.
Ceron-Litvoc, 2009, 1281	This paper reports a systematic review and metaanalysis of all randomized controlled trials (RCTs) that evaluate the use of carbamazepine in acute and maintenance phases of BD compared to lithium.	Bipolar disorder 1 and 2	Maintenance treatment with carbamazepine vs. Carbamazepine	Total: 15 (unclear) Admissions data: 2 (202)	No	5	Conclusion This review suggests that carbamazepine might be comparable to lithium in terms of efficacy and safety, and therefore a valuable option in the treatment of both manic and maintenance phases.

Chauhan, 2012, 1348	1. To compare the safety and efficacy of daily oral antileukotrienes with that of inhaled corticosteroids; 2. to determine the dose of inhaled corticosteroids equivalent to the effect of anti-leukotrienes in the management of asthma in adults and children; and 3. to explore different factors such as patients' age group, disease severity, anti-leukotriene used, intervention duration, hydrofluoroalkane-propelled beclomethasone or equivalent (HFA-BDP eq) dose of inhaled corticosteroids, methodological quality, publication status and funding that could influence the magnitude of effect.	Asthma	Anti-leukotriene vs. Inhaled glucocorticoids (in hydrofluorocarbon-beclomethasone dipropionate equivalent)	Total: 56 (13338) Admissions data: 12 (2715)	No	11	As monotherapy, inhaled corticosteroids display superior efficacy to anti-leukotrienes in adults and children with persistent asthma; the superiority is particularly marked in patients with moderate airway obstruction. On the basis of efficacy, the results support the current guidelines' recommendation that inhaled corticosteroids remain the preferred monotherapy.
Chauhan, 2014, 8681	To compare the safety and efficacy of adding LABA versus LTRA in children and adults with asthma who remain symptomatic in spite of regular treatment with ICS. We specifically wished to examine the relative impact of the two agents on asthma exacerbations, lung function, symptoms, quality of life, adverse health events and withdrawals.	Asthma treated with inhaled corticosteroids	Long acting beta agonists with inhaled corticosteroids vs. Leukotriene receptor antagonists plus inhaled corticosteroids	Total: 18 (7126) Admissions data: 5 (4345)	No	9	In adults with asthma that is inadequately controlled by predominantly low-dose ICS with significant bronchodilator reversibility, the addition of LABA to ICS is modestly superior to the addition of LTRA in reducing oral corticosteroid-treated exacerbations, with an absolute reduction of two percentage points. Differences favouring LABA over LTRA as adjunct therapy were observed in lung function and, to a lesser extend, in rescue medication use, symptoms and quality of life. The lower overall withdrawal rate and the higher proportion of participants satisfied with their therapy indirectly favour the combination of LABA + ICS over LTRA + ICS. Evidence showed a slightly increased risk of SAE with LABA compared with LTRA, with an absolute increase of one percentage point. Our findings modestly support the use of a single inhaler for the delivery of both LABA and low- or medium-dose ICS. Because of the paucity of paediatric trials, we are unable to draw firm conclusions about the best adjunct therapy in children.
Chen, 2015, 8742	We aimed to summarize the evidence for the efficacy of MRAs in patients with either heart failure with PEF (HF-PEF) or myocardial infarction with PEF (MI-PEF).	Patients with preserved ejection fraction ≥40%	Aldosterone antagonists vs. Placebo or control	Total: 14 (6428) Admissions data: 4 (4551)	Yes	8	MRA treatment in PEF patients led to reduced hospitalization for heart failure, quantifiable improvements in quality of life and diastolic function, and reversal of cardiac remodeling, but did not provide any all-cause mortality benefit.
Cheyne, 2015, 8744	To compare the relative effects of tiotropium to ipratropium bromide on markers of quality of life, exacerbations, symptoms, lung function and serious adverse events in patients with COPD using available randomised controlled trial data.	Chronic obstructive pulmonary disease	Tiotropium vs. Ipratropium bromide	Total: 2 (1073) Admissions data: 2 (1073)	No	10	This review shows that tiotropium treatment, when compared with ipratropium bromide, was associated with improved lung function, fewer hospital admissions (including those for exacerbations of COPD), fewer exacerbations of COPD and improved quality of life. There were both fewer serious adverse events and disease specific events in the tiotropium group, but no significant difference in deaths with ipratropium bromide when compared to tiotropium. Thus, tiotropium appears to be a reasonable choice (instead of ipratropium bromide) for patients with stable COPD, as proposed in guidelines. A recent large double-blind trial of the two delivery devices found no substantial difference in mortality using 2.5 µg or 5 µg of tiotropium via Respimat in comparison to 18 µg via Handihaler.
Chong, 2012, 1438	To compare the relative clinical effects of tiotropium bromide alone versus LABA alone, upon measures of quality of life, exacerbations, lung function and serious adverse events, in people with stable COPD. To critically appraise and summarise current evidence on the costs and cost-effectiveness associated with tiotropium compared to LABA in people with COPD.	Chronic obstructive pulmonary disease	Tiotropium vs. Long acting beta-agonists	Total: 7 (12223) Admissions data: 6 (12123)	No	10	In people with COPD, the evidence is equivocal as to whether or not tiotropium offers greater benefit than LABAs in improving quality of life; however, this is complicated by differences in effect among the LABA types.Tiotropium wasmore effective than LABAs as a group in preventing COPD exacerbations and disease-related hospitalisations, although there were no statistical differences between groups in overall hospitalisation rates or mortality during

							the study periods. There were fewer serious adverse events and study withdrawals recorded with tiotropium compared with LABAs. Symptom improvement and changes in lung function were similar between the treatment groups. Given the small number of studies to date, with high levels of heterogeneity among them, one approach may be to give a COPD patient a substantial trial of tiotropium, followed by a LABA (or vice versa), then to continue prescribing the long-acting bronchodilator that the patient prefers. Further studies are needed to compare tiotropium with different LABAs, which are currently ongoing. The available economic evidence indicates that tiotropium may be cost-effective compared with salmeterol in several specific settings, but there is considerable uncertainty around this finding.
Coeytaux, 2014, 8747	We conducted a systematic review to evaluate the comparative effectiveness and safety of monotherapy or combination therapy for PAH using endothelin receptor antagonists, phosphodiesterase inhibitors, or prostanoids.	Pulmonary aterial hypertension	Combination drug therapy with endothelin receptor antagonist, phosphodiesterase inhibitors, and/or prostanoids<br>vs.<br>Placebo; Monotherapy	Total:<br>28 (3613)<br><br>Admissions data:<br>9 (1918)	No	5	Although no studies were powered to detect a mortality reduction, monotherapy was associated with improved 6MWD and reduced hospitalization rates. Our findings also suggest an improvement in 6MWD when a second drug is added to monotherapy.
Cordina, 2005, 1606	To assess the effects of pharmacological cardioversion of atrial fibrillation in adults on the annual risk of stroke, peripheral embolism, and mortality.	Paroxysmal, sustained or permanent atrial fibrillation or flutter	Rhythm (antiarrhythmic drugs)<br>vs.<br>Rate control (rate control drugs)	Total:<br>2 (4167)<br><br>Admissions data:<br>2 (4312)	No	8	There is no evidence that pharmacological cardioversion of atrial fibrillation to sinus rhythmis superior to rate control. Rhythm control is associated with more adverse effects and increased hospitalisation. It does not reduce the risk of stroke. The conclusions cannot be generalised to all people with atrial fibrillation. Most of the patients included in these studies were relatively older (>60 years) with significant cardiovascular risk factors.
Costa, 2013, 1629	To evaluate patients with IBD treated with infliximab and providing data on the rate of complications (hospitalizations and/or surgery).	Irritable bowel disease	Infliximab<br>vs.<br>Placebo; no treatment; other active non-biologic drug, non-adherence to infliximab therapy and episodic/nonpersistence infliximab therapy	Total:<br>9 (1912)<br><br>Admissions data:<br>6 (1736)	No	8	The best evidence available points toward a reduction of the risk of hospitalization and major surgery requirement in patients with IBD treated with infliximab. This impact is clinically and economically relevant because hospitalization and surgery are considered to be markers of disease severity and significantly contribute to the total direct costs associated with IBD.
Danchin, 2006, 1752	To provide information complementary to that provided by previous meta-analyses in patients who have CAD and either signs of heart failure or impaired systolic function	Stable coronary artery disease	Angiotensin-converting enzyme inhibitors<br>vs.<br>Placebo	Total:<br>7 (33960)<br><br>Admissions data:<br>6 (33500)	No	8	Angiotensin-converting enzyme inhibitors reduce total mortality and major cardiovascular end points in patients who have CAD and no left ventricular systolic dysfunction or heart failure.
DiNicolantonio<br>,<br>2013, 1991	To compare carvedilol against the most frequently prescribed b1-selective BBs.	Acute myocardial infarction (AMI) and hear tfailure (HF)	Carvedilol<br>vs.<br>Beta-blockers	Total:<br>8 (4563)<br><br>Admissions data:<br>2 (3099)	Yes	5	Compared to b1-selective BBs used in HF (8 trials, n [4,563), carvedilol significantly reduced all-cause mortality (risk ratio 0.85, 95% confidence interval 0.78 to 0.93, p [0.0006). In 3 trials of patients with AMI (n [644), carvedilol significantly reduced all-cause mortality by 45% (fixed-effects model: risk ratio 0.55, 95% confidence interval 0.32 to 0.94, p [0.03, random-effects model: risk ratio 0.56, 95% confidence interval 0.26 to 1.12, p = 0.10), with no reduction in non-fatal MI (risk ratio 0.61, 95% confidence interval 0.31 to 1.22, p [0.16). In conclusion, carvedilol, as compared against atenolol, bisoprolol, metoprolol and nebivolol in randomized direct comparison trials, significantly reduced all-cause mortality in systolic HF patients. Additionally, carvedilol significantly reduced all-cause mortality compared with b1-selective

Doyle, 2009, 2055	Investigated the effects of long-term amiodarone therapy on mortality, rhythm control, incidence of hospitalization, and drug intolerance leading to withdrawal of treatment in patients with persistent AF of more than 30 days' duration.	Persistent atrial fibrillation (>30 days' duration)	Amiodarone vs. CALCIUM CHANNEL BLOCKERS; CARDIAC THERAPY; BETA BLOCKING AGENTS	Total: 20 (5060) Admissions data: 5 (2932)	No	8	Amiodarone, as part of a strategy to achieve and maintain sinus rhythm, appears to be safe and effective in patients with persistent AF. However, some patients may not tolerate the adverse effects of this agent.
Ducharme, 2010, 2095	To compare the relative benefit and safety profile of the combination of long-acting ß2 agonists (LABAs) and inhaled corticosteroids (ICS) with a higher dose of inhaled corticosteroids in asthmatic patients with or without previous treatment with inhaled corticosteroids.	Recurrent or chronic asthma	Long-acting beta2-agonists and inhaled corticosteroids vs. Higher dose of inhaled corticosteroids	Total: 48 (15155) Admissions data: 33 (12573)	No	11	In adolescents and adults with sub-optimal control on low dose ICS monotherapy, the combination of LABA and ICS is modestly more effective in reducing the risk of exacerbations requiring oral corticosteroids than a higher dose of ICS. Combination therapy also led to modestly greater improvement in lung function, symptoms and use of rescue ß2 agonists and to fewer withdrawals due to poor asthma control than with a higher dose of inhaled corticosteroids. Apart from an increased rate of tremor and less oral candidiasis with combination therapy, the two options appear relatively safe in adults although adverse effects associated with long-term ICS treatment were seldommonitored. In children, combination therapy did not lead to a significant reduction, but rather a trend towards an increased risk, of oral steroid-treated exacerbations and hospital admissions. These trends raised concern about the safety of combination therapy in view of modest improvement in children under the age of 12 years.
Ebell, 2013, 2148	To determine the effect of oseltamivir on the duration of illness and prevention of serious complications and hospitalizations in adults using both published and unpublished data, by a group of authors with no ties to the manufacturer and considering an appropriate definition for complications requiring antibiotics.	Influenza	Oseltamivir vs. Placebo	Total: 11 (4769) Admissions data: 8 (4327)	Yes	9	There is no evidence that oseltamivir reduces the likelihood of hospitalization, pneumonia or the combined outcome of pneumonia, otitis media and sinusitis in the ITT population.
Edmonds, 2012, 2158	To determine the effectiveness of Inhaled CorticoSteriods on outcomes in the treatment of acute asthma following discharge from the ED. To quantify the effectiveness of ICS therapy on acute asthma following ED discharge, when used in addition to, or as a substitute for, systemic corticosteroids	Acute asthma	Any inhaled corticosteroids plus oral corticosteroid vs. Placebo or standard oral corticosteroid therapy	Total: 12 (2205) Admissions data: 5 (1059)	No	9	There is insufficient evidence that ICS therapy provides additional benefit when used in combination with standard systemic corticosteroid therapy upon ED discharge for acute asthma. There is some evidence that high-dose ICS therapy alone may be as effective as oral corticosteroid therapy when used in mild asthmatics upon ED discharge; however, the confidence intervals were too wide to be confident of equal effectiveness. Further research is needed to clarify whether ICS therapy should be employed in acute asthma treatment following ED discharge. The review does not suggest any reason to stop usual treatment with ICS following ED discharge, even if a course of oral corticosteroids are prescribed.
Edmonds, 2012, 2159	To determine the benefit of ICS for the treatment of patients with acute asthma managed in the emergency department (ED).	Acute asthma	Early use of inhaled corticosteroids in the emergency department vs. Placebo or systemic corticosteroids	Total: 32 (2374) Admissions data: 5 (377)	Yes	9	ICS therapy reduces hospital admissions in patients with acute asthma who are not treated with oral or intravenous corticosteroids. They may also reduce admissions when they are used in addition to systemic corticosteroids; however, the most recent evidence is conflicting. There is insufficient evidence that ICS therapy results in clinically important changes in pulmonary function or clinical scores when used in acute asthma in addition to systemic corticosteroids. Also, there is insufficient evidence that ICS therapy can be used in place of systemic corticosteroid therapy when treating acute asthma. Further research is needed to clarify the most appropriate drug dosage and delivery device, and to define which patients are most likely to benefit from ICS therapy. Use of similar measures and reporting methods of lung

							function, and a common, validated, clinical score would be helpful in future versions of this meta-analysis.
Ezekowitz, 2009, 2304	To summarize the evidence on the efficacy of spironolactone (SP), eplerenone (EP), or canrenoate (CAN) in patients with left ventricular dysfunction.	Left ventricular dysfunction (heart failure, mycardial infarction)	Aldosterone antagonists vs. Placebo or usual care or active comparator (metoprolol or ramipril)	Total: 19 (10807) Admissions data: 9 (8699)	No	6	We demonstrated a 20% reduction in all-cause mortality with the use of aldosterone blockade in a clinically heterogeneous group of clinical trial participants with heart failure and post-MI. In addition, we found a 3.1% improvement in EF. Further study in those with less severe symptoms or preserved systolic function is warranted.
Farne, 2015, 8767	To compare the relative effects on markers of quality of life, exacerbations, symptoms, lung function and serious adverse events in people with COPD randomised to LABA plus tiotropium versus tiotropium alone; or LABA plus tiotropium versus LABA alone.	Chronic obstructive pulmonary disease	Long-acting beta2-agonists (LABA) plus tiotropium vs. Tiotropium or long-acting beta2-agonist alone	Total: 10 (10894) Admissions data: 4 (4856)	Yes	10	The results from this review indicated a small mean improvement in health-related quality of life and FEV1 for participants on a combination of tiotropium and LABA compared to either agent alone, and this translated into a small increase in the number of responders on combination treatment. In addition, adding tiotropium to LABA reduced exacerbations, although adding LABA to tiotropium did not. Hospital admission and mortality were not altered by adding LABA to tiotropium, although there may not be enough data. While it is possible that this is affected by higher attrition in the tiotropium group, one would expect that participants withdrawn from the study would have had less favourable outcomes; this means that the expected direction of attrition bias would be to reduce the estimated benefit of the combination treatment. The results were largely from studies of olodaterol and there was insufficient information to assess whether the other LABAs were equivalent to olodaterol or each other.
Filippini, 2003, 2402	To find out whether recombinant interferons reduced the number of patients who had clinical exacerbations and disease progression, compared with placebo.	Multiple sclerosis	Interferons vs. Placebo	Total: 7 (1215) Admissions data: 2 (391)	No	7	Recombinant interferons slightly reduce the number of patients who have exacerbations during first year of treatment. Their clinical effect beyond 1 year is uncertain and new trials are needed to assess their long-term effectiveness and side-effects.
Fisher, 2014, 8769	The critical evaluation of clinical evidence on the safety and efficacy of autologous adult bone marrow-derived stemcells (BMSC) as a treatment for chronic ischaemic heart disease (IHD) and heart failure.	Chronic ischaemic heart disease and congestive heart failure	Bone marrow stem cells vs. No intervention or a placebo	Total: 23 (1255) Admissions data: 5 (402)	No	11	This systematic review and meta-analysis found moderate quality evidence that BMSC treatment improves LVEF. Unlike in trials where BMSC were administered following acute myocardial infarction (AMI), we found some evidence for a potential beneficial clinical effect in terms of mortality and performance status in the long term (after at least one year) in people who suffer from chronic IHD and heart failure, although the quality of evidence was low.
Fisher, 2015, 8770	To assess available clinical evidence on the safety and efficacy of cell-based therapies for HF.	Heart failure	Bone marrow stem cells vs. No intervention or placebo	Total: 31 (1521) Admissions data: 11 (574)	Yes	7	This study shows evidence that autologous cell therapy may be beneficial for patients having HF, but further evidence is required.
Fisher, 2015, 8771	To determine the safety and efficacy of autologous adult bone marrow stem cells as a treatment for acute myocardial infarction (AMI), focusing on clinical outcomes.	Acute myocardial infarction	Autologous adult bone marrow-derived cells following successful revascularisation by angioplasty or cardiac surgery vs. No intervention or placebo	Total: 41 (2732) Admissions data: 13 (1194)	No	11	The results of this review suggest that there is insufficient evidence for a beneficial effect of cell therapy for AMI patients. However, most of the evidence comes from small trials that showed no difference in clinically relevant outcomes. Further adequately powered trials are needed and until then the efficacy of this intervention remains unproven.
Fox, 2011, 2496	To examine whether combination therapy is more efficacious than monotherapy therapy for treatment of pulmonary artery hypertension.	Pulmonary arterial hypertension (PAH)	Combination therapy (Tadalafil and Bosentan; INH treprostinil and Bosentan or sildenafil; Sildenafil and Epoprostenol; INH iloprost and Bosentan) vs.	Total: 6 (729) Admissions data: 5 (634)	No	5	CT did not decrease the combined end point of mortality, admission for worsening PAH, lung transplantation, or escalation of PAH therapy (RR 0.42, 95% CI 0.17 to 1.04). In conclusion, this meta-analysis suggests that in PAH CT does not offer an advantage over MT apart from modestly increasing exercise capacity. However,

			Placebo (Monotherapy when combined with co-intervention)				given the paucity of good-quality data, more studies are required to define the efficacy of CT in this population before establishing final guidelines.
Fu, 2012, 2549	The aim of this meta-analysis was to assess the efficacy of ACE inhibitors in HFPEF patients, based on the results of the most recently published prospective studies.	Heart failure with preserved left ventricular ejection fraction defined as signs or symptoms of heart failure and EF≥40%)	Angiotensin converting enzyme inhibitor vs. Placebo or 'other classes of drugs such as monotherapy or first line therapy' for heart failure with preserved ejection fraction.	Total: 7 (2554) Admissions data: 4 (1803)	No	7	In patients with chronic heart failure with preserved ejection fraction, ACE inhibitors reduced all-cause mortality without affecting mortality due to heart failure and any-cause rehospitalization.
Gandhi, 2014, 2599	We have performed a systematic review of studies assessing the efficacy of HSS in combination with furosemide for the treatment of acute advanced CHF.	Heart Failure	Hypertonic saline and furosemide vs. Furosemide alone	Total: 10 (2845) Admissions data: 4 (1012)	Yes	8	The results of this meta-analysis demonstrate that in patients with advanced CHF concomitant hypertonic saline administration improved weight loss, preserved renal function, and decreased length of hospitalization, mortality and heart failure rehospitalization. Pending further validation, there is promise for hypertonic saline as an advanced therapy for the management of acute advanced CHF.
Gao, 2014, 8775	We conducted a systematic review and meta-analysis to evaluate the impacts of macrolides on the number of bronchiectasis exacerbations and other clinical measures, i.e. admission for exacerbations, QoL, spirometric indices and adverse events.	Non-cystic fibrosis bronchiectasis	Macrolide vs. Placebo	Total: 9 (559) Admissions data: 2 (224)	No	6	Macrolide maintenance therapy, both in adults and children, was effective and safe in reducing bronchiectasis exacerbations, but not the admissions for exacerbations. In addition, macrolide administration in adults was associated with improvement in QoL and spirometry, but not 6WMT. Future studies are warranted to verify the optimal populations and clarify its potential effects on antimicrobial resistance.
Garside, 2007, 2640	To establish the effectiveness and cost-effectiveness of cinacalcet for the treatment of SHPT for people on dialysis due to ESRD.	Hyperparathyroidism secondary to ESRD	Cinacalcet vs. Placebo or 'standard care', which may include: phosphate binders, vitamin D and/or parathyroidectomy	Total: 7 (846) Admissions data: 4 (1184)	No	10	Cinacalcet in addition to standard care is more effective than placebo plus standard care at reducing PTH levels without compromising calcium levels. However, there is limited information about the impact of this reduction on patient-relevant clinical outcomes. Given the short follow-up in the trials, it is unclear how data should be extrapolated to the long term.
Grimwade, 2003, 2933	To assess the effects of routinely administered cotrimoxazole on death and illness episodes in HIV infected adults.	Human immunodeficiency virus	Cotrimoxazole vs. Placebo or usual treatment	Total: 4 (1476) Admissions data: 3 (764)	No	9	In the trials included in the review, cotrimoxazole prophylaxis had a beneficial effect in preventing death and illness episodes in adults with both early and advanced HIV disease. However, the wider applicability of these findings is unclear, in particular to areas with higher background bacterial resistance to cotrimoxazole. Further trials would be required in differing settings to widen applicability.
Grooten, 2015, 8780	The aim of the present systematic review and meta-analysis is to summarize the available evidence on the effectiveness of CCS therapy for HG.	Pregnant women with hyperemesis gravidarum	Corticosteroids (Prednisolone; Methylprednisolone; Hydrocortisone) vs. Prevailing treatment or a placebo	Total: 5 (310) Admissions data: 4 (214)	No	7	Meta-analysis yielded no effect of CCS therapy on readmission rates. Single small studies indicated possible beneficial effects on other outcomes. Future high-quality trials are necessary and would benefit from consensus on HG definition and core outcomes of HG therapy.
Hemkens, 2016, 8784	To evaluate potential cardiovascular benefits and harms of a continuous long-term treatment with colchicine in any population, and specifically in people with high cardiovascular risk.	Any condition or disease	Colchicine vs. Placebo, inactive control, or active control	Total: 39 (4992) Admissions data: 2 (599)	No	11	Overall, we found that further research would probably change our assessment of the benefits and harms of colchicine. Our findings should therefore be interpreted with caution. However, new treatments in heart diseases are urgently needed. Although there is much uncertainty around the benefits and harms of colchicine treatment, it may be associated with cardiovascular benefits, especially on myocardial infarction. We therefore think that large high-quality clinical trials should be conducted to further investigate colchicine in heart disease.

Heran, 2012, 3275	To assess the benefit and harm of ARBs compared with ACE inhibitors (ACEIs) or placebo on mortality, morbidity and withdrawals due to adverse effects in patients with symptomatic HF and left ventricular systolic dysfunction or preserved systolic function.	Heart failure with and without preserved ejection fraction	Angiotensin II receptor blocker with or without angiotensin converting enzyme inhibitor vs. Placebo, in addition to standard therapy (ACE-inhibitors)	Total: 24 (25051) Admissions data: 9 (14337)	Yes	10	(? Monotherapy; - dual therapy)In patients with symptomatic HF and systolic dysfunction or with preserved ejection fraction, ARBs compared to placebo or ACEIs do not reduce total mortality or morbidity Adding an ARB in combination with an ACEI does not reduce total mortality or total hospital admission but increases withdrawals due to adverse effects compared with ACEI alone.
Hood, 2014, 3415	To examine the effectiveness of digitalis glycosides in treating HF in patients with normal sinus rhythm. To examine the effects of digitalis in patients taking diuretics and angiotensin-converting enzyme inhibitors; in patients with varying severity and duration of disease; in patients with prior exposure to digitalis versus no prior exposure; and in patients with “HF due to systolic dysfunction” versus “HF with preserved ejection fraction.”	Heart failure	Digitalis (digoxin or digitoxin) vs. Placebo	Total: 13 (7896) Admissions data: 4 (7262)	Yes	7	The literature indicates that digitalis may have a useful role in the treatment of patients with HF who are in normal sinus rhythm. New trials are needed to elucidate the importance of the dosage of digitalis and its usefulness in the era of beta-blockers and other agents shown to be effective in treating HF.
Jaeschke, 2008, 3639	To determine the safety of long-acting b-agonists among patients with asthma using corticosteroids.	Asthma	Long acting beta agonists with inhaled corticosteroids vs. Inhaled corticosteroids	Total: 62 (29401) Admissions data: 44 (22396)	No	5	In patients with asthma using ICS, LABA did not increase the risk of asthma-related hospitalizations. There were very few asthma-related deaths and intubations, and events were too infrequent to establish LABA’s relative effect on these outcomes.
Jaeschke, 2008, 3640	To assess the safety of formoterol in patients with asthma using inhaled corticosteriods.	Asthma	Long acting beta agonists with inhaled corticosteroids vs. Inhaled corticosteroids	Total: 16 (10638) Admissions data: 16 (10638)	No	4	In patients with asthma using inhaled corticosteroids formoterol decreased the risk of asthma-related hospitalizations. There were too few asthma-related deaths and intubations to establish formoterol’s relative impact on these outcomes.
James, 2008, 3657	To examine whether topical or intraluminal antibiotics reduce catheter-related bloodstream infection compared with no antibiotic therapy in adults undergoing hemodialysis.	Long-term hemodialysis using a central venous catheter	Topical prophylaxtic antibiotics vs. Another or no antimicrobial agent	Total: 16 (1395) Admissions data: 2 (305)	No	7	Both topical and intraluminal antibiotics reduced the rate of bacteremia as well as the need for catheter removal secondary to complications. Whether these strategies will lead to antimicrobial resistance and loss of efficacy over longer periods remains unclear.
Jankowska, 2016, 8790	The aim of our study was to summarize the evidence in the form of a meta-analysis of all randomized controlled trials that investigated the effects of i.v. iron therapy in iron-deficient patients with systolic HF (also analysed separately in anaemic and non-anaemic subjects).	Iron-deficient patients with systolic heart failure	Intravenous iron therapy vs. Placebo	Total: 5 (509) Admissions data: 4 (835)	No	6	The evidence indicates that i.v. iron therapy in iron-deficient patients with systolic HF improves outcomes, exercise capacity, and quality of life, and alleviates HF symptoms.
Jefferson, 2014, 3696	To describe the potential benefits and harms of NIs for influenza in all age groups by reviewing all clinical study reports of published and unpublished randomised, placebo-controlled trials and regulatory comments.	Influenza	Oseltamivir for influenza prophylaxis or treatment vs. Placebo	Total: 46 (24251) Admissions data: 11 (7828)	No	11	Oseltamivir and zanamivir have small, non-specific effects on reducing the time to alleviation of influenza symptoms in adults, but not in asthmatic children. Using either drug as prophylaxis reduces the risk of developing symptomatic influenza. Treatment trials with oseltamivir or zanamivir do not settle the question of whether the complications of influenza (such as pneumonia) are reduced, because of a lack of diagnostic definitions. The use of oseltamivir increases the risk of adverse effects, such as nausea, vomiting, psychiatric effects and renal events in adults and vomiting in children. The lower bioavailability may explain the lower toxicity of zanamivir compared to oseltamivir. The balance between benefits and harms should be considered when making decisions about use of both NIs for either the prophylaxis or treatment of influenza. The influenza virus-specific mechanism of action proposed by the producers does not fit the clinical evidence.

Jong, 2002, 3781	To determine the effect of ARBs on the survival and hospitalization rates in patients with HF.	Heart failure	Angiotensin receptor blockers (Angiotensin II receptor blocker and angiotensin converting enzyme inhibitor) vs. Angiotensin converting enzyme inhibitors alone	Total: 17 (12469) Admissions data: 6 (10031)	No	6	This meta-analysis cannot confirm that ARBs are superior in reducing all-cause mortality or HF hospitalization in patients with symptomatic HF, particularly when compared with ACEIs. However, the use of ARBs as monotherapy in the absence of ACEIs or as combination therapy with ACEIs appears promising.
Kang, 2007, 3877	The aim of this study was to investigate the effectiveness and tolerability of G-CSF treatment with regard to global left ventricular function in patients with myocardial infarction (MI).	Myocardial infarction	Granulocyte colony-stimulating factor (G-CSF) treatment vs. Placebo or blank control	Total: 7 (364) Admissions data: 8 (364)	No	6	Based on the studies included in this meta-analysis, G-CSF treatment improved the LVEF in AMI (but not OMI) at 3 to 12 months follow-up. Treatment with G-CSF was generally well tolerated.
Kang, 2008, 3879	Therefore, we conducted a systematic review and metaanalysis of available prospective randomized controlled trials (RCTs) to analyze the efficacy and safety of bone marrow-derived stem/progenitor cells (BMC) treatment with global left ventricular function in acute myocardial infarction.	Acute myocardial infarction	intracoronary bone marrow-derived stem/progenitor cells vs. Placebo or blank control	Total: 6 (517) Admissions data: 6 (517)	No	7	On the basis of present evidence, intracoronary BMC infusion in patients with AMI seems to be safe and associated with slight improvement of the left ventricular ejection fraction at 3–6 months' follow-up.
Kansagara, 2013, 3885	To evaluate the benefits and harms of treatments for anemia in adults with heart disease.	Heart disease	Erythropoiesis stimulating agent vs. Placebo or those comparing more intensive with less intensive interventions	Total: 26 (9695) Admissions data: 4 (3901)	No	8	Higher transfusion thresholds do not consistently improve mortality rates, but large trials are needed. Intravenous iron may help to alleviate symptoms in patients with heart failure and iron deficiency and also warrants further study. Erythropoiesisstimulating agents do not seem to benefit patients with mild to moderate anemia and heart disease and may be associated with serious harms.
Karner, 2011, 3908	To assess the relative effects of inhaled corticosteroid and long-acting beta2-agonist combination therapy in addition to tiotropium compared to tiotropium or combination therapy alone in patients with chronic obstructive pulmonary disease.	Chronic obstructive pulmonary disease	Tiotropium plus long acting beta 2 agonists and or inhaled corticosteroid vs. Inhaled tiotropium bromide alone or inhaled combination corticosteroid and long-acting beta2- agonist	Total: 3 (1051) Admissions data: 2 (961)	Yes	10	To date there is uncertainty regarding the long-termbenefits and risks of treatment with tiotropium in addition to inhaled corticosteroid and long-acting beta2-agonist combination therapy onmortality, hospitalisation, exacerbations of COPDand pneumonia. The addition of combination treatment to tiotropium has shown improvements in average health-related quality of life and lung function.
Karner, 2014, 8692	To evaluate data from randomised controlled trials (RCTs) comparing the efficacy of tiotropium and placebo in patients with chronic obstructive pulmonary disease (COPD), upon clinically important endpoints.	Chronic obstructive pulmonary disease	Tiotropium vs. Placebo	Total: 22 (23309) Admissions data: 21 (22852)	Yes	10	This review shows that tiotropium treatment was associated with a significant improvement in patients' quality of life and it reduced the risk of exacerbations, with a number needed to treat to benefit (NNTB) of 16 to prevent one exacerbation. Tiotropium also reduced exacerbations leading to hospitalisation but no significant difference was found for hospitalisation of any cause or mortality. Thus, tiotropium appears to be a reasonable choice for the management of patients with stable COPD, as proposed in guidelines. The trials included in this review showed a difference in the risk of mortality when compared with placebo depending on the type of tiotropium delivery device used. However, these results have not been confirmed in a recent trial when 2.5mcg or 5mcg of tiotropium via Respimat was used in a direct comparison to the 18 mcg Handihaler.
Kaur, 2014, 3937	To determine whether raising HDL-C with pharmacologic therapies translates into beneficial cardiovascular outcomes and to find out if this change was proportional to the percentage change in HDL levels.	Cardiovascular disease	High density lipoprotein raising therapeutic agents as monotherapy or co-administered with statins vs. The control arm should have had an intervention which	Total: 12 (53721) Admissions data: 7 (40155)	No	7	Increasing HDL levels via pharmacological manipulation beyond optimal lipid lowering therapy for secondary prevention is not beneficial.

			would permit appropriate attribution of the results to HDL targeting drug.				
Kew, 2014, 4002	To assess the safety and efficacy of IVMgSO4 in adults treated for acute asthma in the emergency department.	Acute asthma	Intravenous magnesium sulfate<br>vs.<br>Placebo	Total:<br>14 (2313)<br><br>Admissions data:<br>11 (972)	Yes	10	This review provides evidence that a single infusion of 1.2 g or 2 g IV MgSO4 over 15 to 30 minutes reduces hospital admissions and improves lung function in adults with acute asthma who have not responded sufficiently to oxygen, nebulised short-acting beta2-agonists and IV corticosteroids. Differences in the ways the trials were conducted made it difficult for the review authors to assess whether severity of the exacerbation or additional co-medications altered the treatment effect of IV MgSO4. Limited evidence was found for other measures of benefit and safety. Studies conducted in these populations should clearly define baseline severity parameters and systematically record adverse events. Studies recruiting participants with exacerbations of varying severity should consider subgrouping results on the basis of accepted severity classifications.
Kew, 2013, 4003	To assess the effects of twice-daily long-acting beta2-agonists compared with placebo for patients with COPD on the basis of clinically important endpoints, primarily quality of life and COPD exacerbations.	Chronic obstructive pulmonary disease	Long-acting beta2-agonists<br>vs.<br>Not reported	Total:<br>26 (14939)<br><br>Admissions data:<br>7 (3804)	Yes	11	Moderate-quality evidence from 26 studies showed that inhaled long-acting beta2-agonists are effective over the medium and long term for patients with moderate to severe COPD. Their use is associated with improved quality of life and reduced exacerbations, including those requiring hospitalisation. Overall, findings showed that inhaled LABAs did not significantly reduce mortality or serious adverse events.
Kew, 2014, 4004	To assess the risk of pneumonia associated with the use of fluticasone and budesonide for COPD.	Chronic obstructive pulmonary disease	Fluticasone (with or without long acting beta2-agonist)<br>vs.<br>Placebo or long acting beta2-agonist	Total:<br>43 (31397)<br><br>Admissions data:<br>24 (25976)	Yes	10	Budesonide and fluticasone, delivered alone or in combination with a LABA, are associated with increased risk of serious adverse pneumonia events, but neither significantly affected mortality compared with controls. The safety concerns highlighted in this review should be balanced with recent cohort data and established randomised evidence of efficacy regarding exacerbations and quality of life. Comparison of the two drugs revealed no statistically significant difference in serious pneumonias, mortality or serious adverse events. Fluticasone was associated with higher risk of any pneumonia when compared with budesonide (i.e. less serious cases dealt with in the community), but variation in the definitions used by the respectivemanufacturers is a potential confounding factor in their comparison. Primary research should accurately measure pneumonia outcomes and should clarify both the definition and the method of diagnosis used, especially for new formulations and combinations for which little evidence of the associated pneumonia risk is currently available. Similarly, systematic reviews and cohorts should address the reliability of assigning 'pneumonia' as an adverse event or cause of death and should determine how this affects the applicability of findings.
Kew, 2016, 8802	To assess the effects of adding a long-acting muscarinic antagonist (LAMA) to combination long-acting beta2-agonists (LABA) and inhaled corticosteroids (ICS) in adults whose asthma is not well controlled by LABA/ICS.	Asthma	Long-acting muscarinic antagonists (LAMA) added to combination long-acting beta2-agonists and inhaled corticosteroids (LABA/ICS)<br>vs.<br>Long-acting beta2-agonists and inhaled corticosteroids (LABA/ICS)	Total:<br>4 (1197)<br><br>Admissions data:<br>3 (1191)	No	10	Tiotropium add-on may have additional benefits over LABA/ICS alone in reducing the need for rescue oral steroids in people with severe asthma. The effect was imprecise, and there was no evidence for other LAMA preparations. Possible benefits on quality of life were negligible, and evidence for the effect on serious adverse events was inconsistent. There are likely to be small added benefits for tiotropium Respimat 5 µg daily on lung function and asthma control over LABA/ICS alone and fewer non-serious adverse events. The benefit of tiotropium add-on on the frequency of hospital admission is still unknown, despite year-long trials.

							Ongoing and future trials should clearly describe participants' background medications to help clinicians judge how the findings relate to stepwise care. If studies test LAMAs other than tiotropium Respimat for asthma, they should be at least six months long and use accepted and validated outcomes to allow comparisons of the safety and effectiveness between different preparations.
Kew, 2015, 8803	To assess the efficacy and safety of adding a LAMA to ICS compared with adding a LABA for adults whose asthma is not well controlled on ICS alone.	Asthma not well controlled with inhaled corticosteroids alone	Long acting muscarinic antagonists added to inhaled corticosteroids<br>vs.<br>Long-acting beta2-agonists (LABA) added to inhaled corticosteroids (ICS)	Total:<br>8 (unclear)<br><br>Admissions data:<br>4 (2022)	No	11	Direct evidence of LAMA versus LABA as add-on therapy is currently limited to studies of less than six months comparing tiotropium (Respimat) to salmeterol, and we do not know how they compare in terms of exacerbations and serious adverse events. There was moderate quality evidence that LAMAs show small benefits over LABA on some measures of lung function, and high quality evidence that LABAs are slightly better for quality of life, but the differences were all small. Given the much larger evidence base for LABA<br>versus placebo for people whose asthma is not well controlled on ICS, the current evidence is not strong enough to say that LAMA can be substituted for LABA as add-on therapy. The results of this review, alongside pending results from related reviews assessing the use of LAMA in other clinical scenarios, will help to define the role of these drugs in asthma and it is important that they be updated as results from ongoing and planned trials emerge.
Kew, 2015, 8805	To assess the effects of macrolides for managing chronic asthma.	Chronic asthma	Macrolides<br>vs.<br>Placebo	Total:<br>23 (1513)<br><br>Admissions data:<br>2 (143)	No	11	Existing evidence does not show macrolides to be better than placebo for the majority of clinical outcomes. However, they may have a benefit on some measures of lung function, and we cannot rule out the possibility of other benefits or harms because the evidence is of very low quality due to heterogeneity among patients and interventions, imprecision and reporting biases. The reviewhighlights the need for researchers to report clinically relevant outcomes accurately and completely using guideline definitions of exacerbations and validated scales. The possible benefit of macrolides in patients with non-eosinophilic asthma based on subgroup analyses in two of the included studies may require further investigation.
Kishimoto, 2013, 4065	Few controlled trials compared second-generation antipsychotics (SGAs) with first-generation antipsychotics (FGAs) regarding relapse prevention in schizophrenia. We conducted a systematic review/meta-analysis of randomized trials, lasting >6 months comparing SGAs with FGAs in schizophrenia.	Schizophrenia	Second generation antipsychotics<br>vs.<br>First generation antipsychotics	Total:<br>23 (4504)<br><br>Admissions data:<br>11 (2869)	No	6	In conclusion, results from this meta-analysis suggest that, whereas individually SGAs were not consistently superior to FGAs, as a group, SGAs were associated with less study-defined relapse, overall treatment failure and hospitalization than FGAs, having a modest but clinically relevant effect size. Future relapse prevention studies should carefully assess EPS and adherence. Moreover, additional studies with a variety of SGAs using non-haloperidol<br>FGA comparators at low-medium doses that do not produce significantly greater EPS than SGAs47 are needed to extend these findings. In particular, sufficiently large data sets are needed to allow examination of the relative merits of individual SGAs, and to guide an individualized and evidence-based maintenance treatment selection in schizophrenia.
Kishimoto, 2014, 4067	While long-acting injectable antipsychotics (LAIs) are hoped to reduce high relapse rates in schizophrenia, recent randomized controlled trials (RCTs) challenged the benefits of LAIs over oral antipsychotics (OAPs).	Schizophrenia	Long-acting injectable antipsychotics<br>vs.<br>Oral Antipsychotics	Total:<br>21 (5176)<br><br>Admissions data:<br>10 (2341)	No	8	In RCTs, which are less representative of real-world patients than naturalistic studies, pooled LAIs did not reduce relapse compared with OAPs in schizophrenia patients. The exceptions were FGA-LAIs, mostly consisting of fluphenazine-LAI studies, which were all conducted through 1991. Because this finding is vulnerable to a cohort bias, studies comparing FGA-LAI vs second-generation

							antipsychotics-LAI and LAI vs OAP RCTs in real-world patients are needed.
Komossa, 2010, 4123	To evaluate the effects of olanzapine compared to other atypical antipsychotics for people with schizophrenia and schizophrenia-like psychosis.	Schizophrenia and schizophrenia-like psychosis	Olanzapine or other atypical antipsychotic drugs vs. Second generation (atypical) antipsychotics	Total: 50 (9100) Admissions data: 4 (1932)	No	9	Olanzapine may be a somewhat more efficacious drug than some other second generation antipsychotic drugs. This small superiority in efficacy needs to be weighed against a larger weight gain and associated metabolic problems than most other second generation antipsychotic drugs, except clozapine. These conclusions are tentative due to the large number of people leaving the studies early which possibly limits the validity of the findings. Further large, well-designed trials are necessary to establish the relative effects of different second generation antipsychotic drugs.
Kuenzli, 2010, 4206	To investigate the effect of adding ARBs to ACE inhibitor therapy alone in terms of clinically relevant beneficial and adverse patient important outcomes including hospital readmissions for any reason.	Left ventricular dysfunction or congestive heart failure and background therapy with ACE inhibitor therapy in at least 90% of patients (i.e. patients on ACE inhibitors)	Angiotensin II receptor blocker and angiotensin converting enzyme inhibitor vs. Angiotensin enzyme converting inhibitors alone	Total: 8 (18061) Admissions data: 6 (17948)	No	7	Combination therapy with ARBs and ACE inhibitors reduces admissions for heart failure in patients with congestive heart failure when compared to ACE inhibitor therapy alone, but does not reduce overall mortality or all-cause hospitalization and is associated with more adverse events. Thus, based on current evidence, combination therapy with ARBs and ACE inhibitors may be reserved for patients who remain symptomatic on therapy with ACE inhibitors under strict monitoring for any signs of worsening renal function and/or symptomatic hypotension.
Kumar, 2014, 8815	We undertook a systematic review and metaanalysis to assess whether the maintenance of anaesthesia using propofol TIVA is associated with fewer unplanned hospital admissions than maintenance with the inhalational agents sevoflurane or desflurane.	Ambulatory surgery	Total intravenous anaesthesia using propofol with sevoflurane vs. Inhalational agents sevoflurane or desflurane	Total: 18 (1621) Admissions data: 10 (1621)	Yes	6	Therefore, based on the published evidence to date, maintenance of anaesthesia using propofol appeared to have no bearing on the incidence of unplanned admission to hospital and was more expensive, but was associated with a decreased incidence of early postoperative nausea and vomiting compared with sevoflurane or desflurane in patients undergoing ambulatory surgery.
Kuswardhani, 2011, 4239	To confirm the beneficial effect of BMCs therapy over placebo in AMI patients with inclusion only to the randomized double blind placebo-controlled trials	Acute myocardial infarction	Bone Marrow-derived Stem Cells vs. Control/placebo	Total: 10 (686) Admissions data: 3 (319)	No	6	The resulting meta-analysis concluded that BMCs therapy consistently improves cardiac performance parameters (LVEF, LVESV, and LVEDV) when compared to placebo, even after the establishment of primary intervention. It is also safe to use and prevents the development of recurrent MI and HF.
La Mantia, 2010, 4260	To verify the clinical efficacy of glatiramer acetate in the treatment of MS patients with relapsing remitting (RR) and progressive (P) course.	Multiple sclerosis	Glatiramer acetate vs. Placebo	Total: 6 (3233) Admissions data: 2 (449)	No	9	Glatiramer acetate did show a partial efficacy in RR MS in term of relapse -related clinical outcomes, without any significant effect on clinical progression of disease measured as sustained disability. The drug is not effective in progressive MS patients.
Lasserson, 2011, 4362	To assess the relative effects of fluticasone/salmeterol and budesonide/formoterol in people with asthma.	Chronic asthma	Long acting beta agonists with inhaled corticosteroids vs. Fixed dose combination budesonide and formoterol	Total: 5 (5537) Admissions data: 4 (4879)	Yes	9	Statistical imprecision in the effect estimates for exacerbations and serious adverse events do not enable us to conclude that either therapy is superior. The uncertainty around the effect estimates justify further trials to provide more definitive conclusions; the overall quality of evidence based on GRADE recommendations for the three primary outcomes and withdrawals due to serious adverse events was moderate. We rated the quality of evidence for mortality to be low. Results for lung function outcomes showed that the drugs were sufficiently similar that further research is unlikely to change the effects. No trials were identified in the under-12s and research in this population is a high priority. Evaluation of quality of life is a priority for future research.
Le, 2016, 8817	Our study aimed to assess the efficacy of AA on SCD, hospitalization admission and several common adverse events in patients with HF or post MI.	Heart failure and post-myocardial infarction	Aldosterone Antagonists vs. Placebo or routine treatment	Total: 25 (19333)	No	8	Aldosterone antagonists appear to be effective in reducing SCD and other mortality events, compared with placebo or standard medication in patients with HF and/or after a MI.

				Admissions data: 14 (8151)			
Leucht, 2011, 4505	Non-adherence is a major problem in the treatment of schizophrenia. Depot antipsychotic drugs are thought to reduce relapse rates by improving adherence, but a systematic review of long-term studies in outpatients is not available.	Schizophrenia	Depot antipsychotic drugs vs. Oral formulations of antipsychotic drugs	Total: 10 (1700) Admissions data: 7 (1476)	No	7	Depot antipsychotic drugs significantly reduced relapse. Due to a number of methodological problems in the single trials the evidence is, nonetheless, subject to possible bias.
Leucht, 2012, 4508	To review the effects of maintaining antipsychotic drug treatment for people with schizophrenia compared to withdrawing these agents.	Schizophrenia	Maintenance/continous treatment with antipsychotic drugs vs. Active or inactive placebo, or no treatment	Total: 65 (6493) Admissions data: 16 (2090)	No	11	The results clearly demonstrate the superiority of antipsychotic drugs compared to placebo in preventing relapse. This effect must be weighed against the side effects of antipsychotic drugs. Future studies should focus on outcomes of social participation and clarify the long-term morbidity and mortality associated with these drugs.
Levy, 2010, 4518	Aim of this systematic review was to examine the current published evidence concerning the impact of differing perioperative analgesic regimens on the shortterm outcomes following laparoscopic colorectal surgery.	Patients getting a colorectal resection	Patient controlled analgesia vs. Epidural	Total: 6 (227) Admissions data: 3 (132)	No	3	There is a paucity of data assessing the benefits of postoperative analgesic regimes following laparoscopic colorectal surgery and none of the protocols were shown to be clearly superior. Further studies, including the assessment of spinal analgesia are required to determine the most appropriate analgesic regime following laparoscopic colorectal surgery.
Li, 2016, 8822	To examine the association between dipeptidyl peptidase-4 (DPP-4) inhibitors and the risk of heart failure or hospital admission for heart failure in patients with type 2 diabetes.	Type 2 diabetes	Dipeptidyl peptidase-4 inhibitors vs. Placebo or active antidiabetic drugs (glimepiride)	Total: 38 (31680) Admissions data: 5 (37095)	Yes	9	The relative effect of DPP-4 inhibitors on the risk of heart failure in patients with type 2 diabetes is uncertain, given the relatively short follow-up and low quality of evidence. Both randomised controlled trials and observational studies, however, suggest that thes drugs may increase the risk of hospital admission for heart failure in those patients with existing cardiovascular diseases or multiple risk factors for vascular diseases, compared with no use.
Liew, 2014, 4577	We performed a meta-analysis of all these trials in order to obtain best estimates of the efficacy and safety of warfarin as compared with antiplatelet therapy, in patients with systolic heart failure who are in sinus rhythm.	Heart failure	Warfarin vs. Antiplatelet therapy	Total: 4 (4187) Admissions data: 4 (4187)	No	4	Warfarin as compared with antiplatelet therapy reduces risk of ischemic stroke, does not significantly affect death, myocardial infarction, hospitalization due to heart failure or intracranial hemorrhage and increases major hemorrhage in heart failure patients who are in sinus rhythm.
Lipinski, 2009, 4615	The goal of this study was to systematically review randomized trials comparing statins to placebo for HF and compare the impact of different statins.	Heart failure	Statins vs. Placebo	Total: 10 (10203) Admissions data: 10 (10193)	No	7	In conclusion, meta-analysis of randomized controlled trials demonstrated that statins are safe and improve LVEF and decrease hospitalization for worsening HF.
Liu, 2014, 4629	Given the limited evidence and uncertain effects of betablockers in the patients with HFpEF, this meta-analysis summarized the current data from randomized controlled trials (RCTs) and observational studies (OSs) to determine the impact of the beta-blockers treatment on mortality and hospitalization in the patients with HFpEF (an EF < 40%).	Heart failure with preserved ejection fraction (an EF >/= 40%).	Beta-blockers vs. Non-beta-blocker control	Total: 2 (888) Admissions data: 2 (888)	Yes	4	The beta-blockers treatment for the patients with HFpEF was associated with a lower risk of all-cause mortality, but not with a lower risk of hospitalization. These finding were mainly obtained from observational studies, and further investigations are needed to make an assertion.
Liu, 2014, 8826	Therefore, we performed a meta-analysis of all prospective RCTs to assess the effects of lipophilic statins, including simvastatin, atorvastatin, and pitavastatin, on mortality, hospitalisation for worsening HF, LVEF, and low-density lipoprotein (LDL) cholesterol in HF patients.	Heart failure: (left ventricular ejection fraction < 45%)	Lipophilic Statins vs. No statin or placebo	Total: 13 (1532) Admissions data: 9 (1087)	Yes	7	It appears that further studies are needed to determine if lipophilic statins are beneficial for HF patients.
Liu, 2014, 8827	To assess the feasibility and safety of early oral feeding (EOF) after gastrectomy for gastric cancer through a systematic review and meta-analysis based on randomized controlled trials.	Gastrointestinal cancer surgery	Early oral feeding vs. Traditional postoperative oral feeding	Total: 6 (454)	Yes	7	The result of this meta-analysis showed that EOF after gastric cancer surgery seems feasible and safe, even started at the day of surgery irrespective of the extent of the gastric resection and the type of surgery. However, more prospective, well-designed

				Admissions data: 5 (372)			multicenter RCTs with more clinical outcomes are needed for further validation.
Lopez, 2015, 8829	The aim of this meta-analysis was to evaluate the clinical efficacy (clinical response, clinical remission, and mucosal healing rates), and for the first time the need for colectomy and UC-related hospitalisations of all TNF antagonists (infliximab, adalimumab and golimumab) that have been evaluated in randomised, placebocontrolled phase III trials in adults with moderately to severely active UC. Safety was also evaluated.	Ulcerative colitis	Tumour necrosis factor antagonists (anti-TNF): Infliximab, Adalimumab vs. Placebo	Total: 5 (3654) Admissions data: 2 (1691)	No	8	Anti-tumour necrosis factor therapy is more effective than placebo to induce and maintain clinical remission and mucosal healing. Both infliximab and adalimumab are associated with less hospitalisations. Infliximab reduces the need for colectomy. Anti-tumour necrosis factor therapy does not increase the risk of adverse events.
Magee, 2003, 4812	To assess whether oral beta-blockers are better than placebo, or no beta-blocker, and have advantages over other antihypertensives, for women with mild to moderate pregnancy hypertension.	Mild to moderate hypertension during pregnancy	Beta-blockers vs. Placebo, no therapy, or other antihypertensive drug therapy	Total: 29 (2500) Admissions data: 5 (563)	No	9	Improvement in control of maternal blood pressure with use of beta-blockers would be worthwhile only if it were reflected in substantive benefits for mother and/or baby, and none have been clearly demonstrated. The effect of beta-blockers on perinatal outcome is uncertain; the worrying trend to an increase in SGA infants is partly dependent on one small outlying trial. Large randomised trials are needed to determine whether antihypertensive therapy in general (rather than beta-blocker therapy specifically) results in greater benefit than risk, for treatment of mild-moderate pregnancy hypertension. If so, then it would be appropriate to consider which antihypertensive is best, and beta-blockers should be evaluated.
Magee, 2000, 4815	This overview was undertaken to relate measurable characteristics of participants, interventions, and outcome definitions to demonstrated effects of b-blockers for pregnancy hypertension.	Pregnancy hypertension	Beta-blockers vs. Non-b-blocker or no therapy (placebo, no treatment)	Total: 32 (2474) Admissions data: 5 (563)	No	9	It is not clear that the benefits outweigh the risks when b-blockers are used to treat mild to moderate chronic or pregnancy-induced hypertension, given the unknown overall effect on perinatal outcomes. For severe 'late-onset' pregnancy hypertension, i.v. labetalol is safer than i.v. hydralazine or diazoxide.
Makani, 2013, 4838	We compared the long term efficacy of dual blockade of the renin-angiotensin system (any two of ACE inhibitors, angiotensin receptor blockers, or aliskiren) with monotherapy and evaluated adverse events in patients receiving dual therapy compared with monotherapy.	Not reported	Dual blockade of the renin-angiotensin system (any two of ACE inhibitors, angiotensin receptor blockers, or aliskiren) vs. Single renin-angiotensin blockade (Angiotensin converting enzyme inhibitor or angiotensin receptor blockers or direct renin inhibitors)	Total: 33 (68405) Admissions data: 5 (42071)	No	6	Although dual blockade of the renin-angiotensin system may have seemingly beneficial effects on certain surrogate endpoints, it failed to reduce mortality and was associated with an excessive risk of adverse events such as hyperkalaemia, hypotension, and renal failure compared with monotherapy. The risk to benefit ratio argues against the use of dual therapy.
Makrides, 2014, 4844	To assess the effects of magnesium supplementation during preg- nancy on maternal, neonatal/infant and paediatric outcomes, us- ing the best available evidence.	Pregnancy	Magnesium vs. Not specified	Total: 10 (9090) Admissions data: 3 (1158)	No	9	There is not enough high-quality evidence to show that dietary magnesium supplementation during pregnancy is beneficial.
Mannucci, 2008, 4889	The aim of this meta-analysis of randomized clinical trials (RCT) was to assess whether pioglitazone is also associated with increased cardiovascular risk, as recently reported for rosiglitazone.	Patients at relatively low risk of cardiovascular risk, such as those with metabolic end-points	Pioglitazone vs. Any other treatment with a duration of at least 4 weeks (Placebo, sulphonylureas, or dual peroxisome proliferator-activated receptor-alpha, gamma agonists (glitazars))	Total: 94 (21180) Admissions data: 40 (10322)	No	5	The use of pioglitazone does not appear to be harmful in terms of cardiovascular events and all-cause deaths.
Medic, 2016, 8842	The objective of this study was to estimate the relative efficacy and safety of fixed-dose combination aclidinium/formoterol 400/12 lg twice daily compared	Moderate-to-severe chronic	Aclidinium, Formoterol, Tiotropium	Total: 17 (21954)	No	7	Based on the ITC, aclidinium/formoterol is expected to be more efficacious than tiotropium in terms of lung function and symptom

	to tiotropium 18 lg once daily in adult patients with moderate-to-severe chronic obstructive pulmonary disease (COPD).	obstructive pulmonary disease	vs.<br>Placebo; tiotropium	Admissions data: unclear (unclear)			control while providing comparable HRQoL results and safety profile.
Mohammed, 2007, 5325	To estimate the effect of intravenous and nebulised magnesium sulphate upon hospital admissions and pulmonary function in adults and children with acute asthma.	Acute asthma	Intravenous or nebulised magnesium sulphate<br>vs.<br>Not pre-specified: Saline solution used in most included studies	Total:<br>24 (1669)<br><br>Admissions data:<br>18 (1359)	Yes	6	Intravenous magnesium sulphate appears to be an effective treatment in children. Further trials are needed of intravenous and nebulised magnesium sulphate in adults and nebulised magnesium sulphate in children.
Møiniche, 2003, 5330	The aim of this systematic review was to critically appraise the existing data on the incidence of perioperative bleeding complications caused by NSAIDs and to quantify the potential impact of NSAIDs on bleeding.	Post-operative tonsillectomy	Nonsteroidal antiinflammatory drugs<br>vs.<br>Non-NSAID treatment	Total:<br>25 (1853)<br><br>Admissions data:<br>8 (506)	Yes	3	Of four bleeding end points (intraoperative blood loss, postoperative bleeding, hospital admission, and reoperation because of bleeding), only reoperation happened significantly more often with NSAIDs: Petoodds ratio, 2.33 (95% confidence interval [CI], 1.12– 4.83) and number-needed-to-treat, 60 (95% CI, 34–277). Compared with opioids, NSAIDs were equianalgesic, and the risk of emesis was significantly decreased (relative rate 0.73; 95% CI, 0.63– 0.85; numbers-needed-totreat, 9; 95% CI, 5–19).
Moore, 2006, 5371	The aim of this review was to systematically examine the available evidence concerning MMF in lupus nephritis from randomised trials and observational studies.	Lupus nephritis	Mycophenolate mofetil<br>vs.<br>Cyclophosphamide and steroid	Total:<br>6 (370)<br><br>Admissions data:<br>2 (220)	No	6	The results form a basis on which to plan future studies and provide a guide for the use of MMF in lupus nephritis until results of larger studies are available. At least one such study is under way.
Nair, 2012, 5515	To determine whether intravenous aminophylline has an additional bronchodilation effect in adult patients with acute asthma when used in conjunction with inhaled beta2-agonists with or without systemic corticosteroids (intravenous, oral, inhaled or combinations of these).	Acute asthma	Aminophylline<br>vs.<br>Placebo or inhaled beta2-agonists alone	Total:<br>19 (817)<br><br>Admissions data:<br>6 (315)	No	9	The use of intravenous aminophylline did not result in significant additional bronchodilation compared to standard care with inhaled beta2-agonists in patients experiencing an asthma exacerbation in the ED setting, or in a significant reduction in the risk of hospital admission.
Nannini, 2013, 5533	To determine the efficacy and safety of combined ICS and LABA for stable COPD in comparison with placebo.	Chronic obstructive pulmonary disease	Combined inhalers<br>vs.<br>Placebo	Total:<br>19 (10400)<br><br>Admissions data:<br>12 (9492)	Yes	10	Combined inhaler therapy led to around a quarter fewer COPD exacerbations than were seen with placebo. A significant reduction in all-cause mortality was noted, but this outcome was dominated by one trial (TORCH), emphasising the need for further trials of longer duration. Increased risk of pneumonia is a concern; however, this did not translate into increased exacerbations, hospitalisations or deaths.
Nannini, 2013, 8694	To assess the efficacy and safety of combined long-acting beta2- agonist and inhaled corticosteroid (LABA/ICS) preparations, as measured by clinical endpoints and pulmonary function testing, compared with inhaled corticosteroids (ICS) alone, in the treatment of adults with chronic obstructive pulmonary disease (COPD).	Chronic obstructive pulmonary disease	Combined corticosteroid and long-acting beta2-agonist in one inhaler<br>vs.<br>Inhaled corticosteroids alone	Total:<br>15 (7814)<br><br>Admissions data:<br>10 (7060)	Yes	10	Combination ICS and LABA offer some clinical benefits in COPDcompared with ICS alone, especially for reduction in exacerbations. This review does not support the use of ICS alone when LABAs are available. Adverse events were not significantly different between treatments. Further long-term assessments using practical outcomes of current and new 24-hour LABAs will help determine their efficacy and safety. For robust comparisons as to their relative effects, long-term head-to-head comparisons are needed.
Ngo, 2010, 5606	To assess the benefit and risk of ESAs for chronic heart failure patients with anaemia.	Chronic heart failure and anaemia of chronic disease	Erythropoiesis stimulating agent<br>vs.<br>Placebo or no treatment	Total:<br>11 (794)<br><br>Admissions data:<br>9 (734)	No	10	Meta-analysis of small RCTs suggests that ESA treatment in patients with symptomatic CHF and mild anaemia (haemoglobin more than 10g/dL) can improve anaemia and exercise tolerance, reduce symptoms and have benefits on clinical outcomes. Confirmation requires well-designed studies with careful attention to dose, haemoglobin treatment target and associated iron therapy.

Ni, 2009, 5611	To examine the safety and efficacy of initiating a combination of long-acting ß2-agonists and inhaled corticosteroids compared to a similar dose or a higher dose of inhaled corticosteroids alone, in steroid-naive children and adults with persistent asthma.	Asthma	Addition of long-acting beta2-agonists to inhaled corticosteroids as first line therapy<br>vs.<br>Same dose of inhaled corticosteroids alone	Total:<br>28 (8050)<br><br>Admissions data:<br>12 (4872)	No	9	In patients with asthma who require daily anti-inflammatory therapy, there is insufficient evidence to support initiating therapy with a combination of inhaled corticosteroids (ICS) and long-acting ß2-agonist (LABA) rather than with inhaled corticosteroids alone.
Ni, 2014, 8850	To assess the efficacy and safety of aclidinium bromide in stable COPD.	Stable chronic obstructive pulmonary	Aclidinium bromide<br>vs.<br>Placebo; long-acting muscarinic antagonist	Total:<br>12 (9547)<br><br>Admissions data:<br>10 (5624)	No	11	Aclidinium is associated with improved quality of life and reduced hospitalisations due to severe exacerbations in patients with moderate to severe stable COPD compared to placebo. Overall, aclidinium did not significantly reduce mortality, serious adverse event or exacerbations requiring oral steroids or antibiotics, or both. Currently, the available data are insufficient and of very low quality in comparisons of the efficacy of aclidinium versus tiotropium. The efficacy of aclidinium versus LABAs cannot be assessed due to inaccurate data. Thus additional trials are recommended to assess the efficacy and safety of aclidinium compared to other LAMAs or LABAs.
Ni, 2015, 9197	Hence, in order to add more information and evidence to clinical practice, we performed an updated meta-analysis to evaluate the efficacy and safety of prophylactic macrolide therapy on the prevention of acute exacerbations of chronic obstructive pulmonary disease.	Chronic obstructive pulmonary disease	Prophylactic use of macrolide antibiotics<br>vs.<br>Placebo	Total:<br>9 (1666)<br><br>Admissions data:<br>5 (1424)	No	6	Our results suggest 6-12 months erythromycin or azithromycin therapy could effectively reduce the frequency of exacerbations in patients with COPD. However, Long-term treatment may bring increased adverse events and the emergence of macrolide-resistance. A recommendation for the prophylactic use of macrolide therapy should weigh both the advantages and disadvantages
Pizzi, 2011, 6161	We carried out a meta-analysis to summarize evidence on the effects of SSRI versus placebo or no antidepressants in all-cause mortality and readmission for CHD in patients with CHD and depression.	Cardiovascular disease and depression	Selective serotonin reuptake inhibitors<br>vs.<br>Placebo or no anti-depressant	Total:<br>4 (2461)<br><br>Admissions data:<br>3 (707)	Yes	9	When only properly randomized trials were considered (n 734 patients), patients on SSRIs showed no significant differences in mortality (risk ratio 0.39, 95% confidence interval 0.08 to 2.01) or CHD readmission rates (0.74, 0.44 to 1.23) compared to controls. Conversely, when all studies were included, SSRI use was associated with a significant decrease in CHD readmission (0.63, 0.46 to 0.86) and mortality rates (0.56, 0.35 to 0.88). A significantly greater improvement in depression symptoms was always apparent in patients on SSRIs with all selected indicators. In conclusion, in patients with CHD and depression, SSRI medication decreases depression symptoms and may improve CHD prognosis.
Poole, 2015, 8867	Primary objective: To determine whether treatment with mucolytics reduces frequency of exacerbations and/or days of disability in patients with chronic bronchitis or COPD. Secondary objectives: To assess whether mucolytics lead to improvement in lung function or quality of life. To determine the frequency of adverse effects associated with use of mucolytics.	Chronic bronchitis or chronic obstructive pulmonary disease	Mucolytic agents<br>vs.<br>Placebo	Total:<br>26 (6233)<br><br>Admissions data:<br>4 (1788)	No	10	In participants with chronic bronchitis or COPD, we are moderately confident that treatment with mucolytics may produce a small reduction in acute exacerbations and a small effect on overall quality of life. Our confidence in the results is reduced by the fact that<br>effects on exacerbations shown in early trials were larger than those reported by more recent studies, possibly because the earlier smaller trials were at greater risk of selection or publication bias, thus benefits of treatment may not be as great as was suggested by previous evidence.
Powell, 2015, 8868	To compare the effects of mepolizumab with placebo on exacerbations and HRQoL in adults and children with chronic asthma.	Asthma	Mepolizumab<br>vs.<br>Placebo	Total:<br>8 (1707)<br><br>Admissions data:<br>2 (690)	Yes	11	It is not possible to draw firm conclusions from this review with respect to the role of mepolizumab in patients with asthma. Our confidence in the results of this review are limited by the fact that the intravenous route is not currently licensed for mepolizumab, and the evidence for the currently licenced subcutaneous route is limited to a single study in participants with severe eosinophilic asthma. The currently available studies provide evidence that

							mepolizumab can lead to an improvement in health-related quality of life scores and reduce asthma exacerbations in people with severe eosinophilic asthma. Further research is needed to clarify which subgroups of patients with asthma could potentially benefit from this treatment. Dosage, ideal dosing regimens and duration of treatment need to be clarified, as the studies included in this review differed in their protocols. There are no studies reporting results from children, so we cannot comment on treatment for this age group. At the present time, larger studies using licenced treatment regimens are required to establish the role of mepolizumab in the treatment of severe asthma.
Rajagopalan, 2011, 6347	Hence, we planned to conduct a systematic review to answer the question whether pharmacotherapy has any beneficial role in the management of HFNEF.	Heart failure with normal ejection fraction (EF of 40%)	Angiotensin II receptor blocker or angiotensin converting enzyme inhibitor; Digoxin vs. Placebo	Total: 6 (8410) Admissions data: 4 (4857)	No	8	There was no significant benefit of pharmacotherapy in HFNEF. This might have been because of a lack of stringent inclusion criteria for patients in the trials and lower power of the studies. Hence trials with well defined inclusion criteria, better power, longer follow-up periods and with echocardiographic parameters as endpoints are required to shed further light on this topic.
Renner, 2012, 6442	To identify, assess, meta-analyze and summarize the evidence concerning the efficacy and safety of primary prophylactic CSFs (GCSFs or GM-CSFs) compared to placebo or no treatment for the prevention of FN, early mortality and infection-related mortality in patients with breast cancer undergoing chemotherapy.	Breast cancer undergoing chemotherapy	Primary prophylactic colony-stimulating factors vs. No treatment or placebo	Total: 8 (2156) Admissions data: 4 (1149)	No	11	In patients with breast cancer receiving chemotherapy,CSFs have shown evidence of bene t in the prevention of FN. There is evidence, though less reliable, of a decrease of all-cause mortality during chemotherapy and a reduced need for hospital care. No reliable evidence was found for a reduction of infection-related mortality, a higher dose intensity of chemotherapy with CSFs or diminished rates of severe neutropenia and infections. The majority of adverse events reported from CSF use were bone pain and injection-site reactions but no conclusions could be drawn regarding late-term side effects.
Rodrigo, 1999, 6564	The objective of this review was to reevaluate the literature on the effectiveness of CCS administration in the treatment of adult patients with acute asthma presenting to an acute-care setting (ie, usually the ED).	Acute asthma	Parenteral or inhaled corticosteroids vs. Parenteral (IV, IM), oral, or inhaled administration of CCSs or placebo	Total: 21 (1049) Admissions data: 6 (480)	Yes	8	This evidence-based evaluation suggests that the administration of parenteral CCSs to the patient on arrival at the emergency department (ED) neither improves airflow obstruction nor reduces the need for hospitalization. Parenteral CCSs probably require > 6 to 24 h to begin to act. Comprehensible conclusions about admission rates in the ED setting are difficult to make. At the 3-h assessment, only high doses of inhaled CCSs (in one study) significantly improved pulmonary function compared with placebo. IV and oral CCSs appear to have equivalent effects, and there is a tendency toward improvement in pulmonary function with medium or high doses.
Rodrigo, 2000, 6566	The purpose of this article was to review the literature to determine whether MgSO 4 provides an additive improvement in adults with acute asthma.	Acute asthma	Nebulised or parenteral magnesium sulfate vs. Placebo	Total: 5 (374) Admissions data: 4 (326)	No	7	The existing evidence reveals that the addition of $MgSO_4$ to ED patients with moderate to severe asthmatic exacerbations does not alter treatment outcomes. Nevertheless, the number and size of studies being pooled remain small.
Rodrigo, 2005, 6568	A review was undertaken to incorporate the more recent evidence available about the effectiveness of treatment with a combination of b2 agonists and anticholinergics compared with b2 agonists alone in the treatment of acute asthma.	Acute asthma	Single or repeated doses of inhaled anticholinergic agents given in combination with inhaled b2 agonists vs. Inhaled b2 agonists alone	Total: 32 (3611) Admissions data: 9 (1556)	Yes	6	This review strongly suggests that the addition of multiple doses of inhaled ipratropium bromide to b2 agonists is indicated as the standard treatment in children, adolescents, and adults with moderate to severe exacerbations of asthma in the emergency setting.
Rodrigo, 2002, 6576	To determine whether continuous nebulization offered an advantage over intermittent nebulization for the treatment of adults with acute asthma in the emergency department (ED).	Acute asthma	Continuous nebulization of B-agonists vs. Intermittent nebulization of B-agonists	Total: 6 (393) Admissions data: 2 (80)	No	6	Overall, this review supports the equivalence of continuous and intermittent albuterol nebulization in the treatment of acute adult asthma.

Rodrigo, 2003, 6578	To determine the effect of the addition of heliox to standard medical care on the course of acute asthma.	Acute asthma presenting to an emergency department (ED) or equivalent care settings	Helium-oxygen gas mixture vs. Oxygen/air	Total: 6 (347) Admissions data: 4 (306)	No	7	The existing evidence does not provide support for the administration of heliumoxygen mixtures to emergency department patients with moderate-to-severe acute asthma. However, these conclusions are based on between-group comparisons and small studies, and these results should be interpreted with caution.
Rowe, 2000, 6670	This systematic review examined the effect of intravenous magnesium sulfate used for patients with acute asthma managed in the emergency department.	Acute asthma presenting to an emergency department	Intravenous magnesium sulfate vs. Placebo	Total: 7 (668) Admissions data: 5 (590)	Yes	8	Current evidence does not clearly support routine use of intravenous magnesium sulfate in all patients with acute asthma presenting to the ED. However, magnesium sulfate appears to be safe and beneficial for patients who present with severe acute asthma. Practice guidelines need to be changed to reflect these results.
Rowe, 1992, 6672	The objective of this study was to determlne the effect of steroid therapy on pulmonary function, admission rates, and relapse rates in patients presenting with acute exacerbations of asthma.	Acute exacerbations of asthma	Parenteral or oral glucocoritcoids vs. Not specified	Total: 30 (unclear) Admissions data: 3 (252)	No	6	The authors conclude that overall, steroid therapy provides Important benefits to patients presenting to emergency departments with acute exacerbations of asthma. Further research into dosage, alternative routes of adminlstratien, and alternative outcome measures is needed.
Rowe, 2001, 6676	To determine the benefit of treating patients with acute asthma with systemic corticosteroids within an hour of presenting to the emergency department (ED).	Acute asthma in the emergency department	Systemic corticosteroids vs. IV saline or placebo	Total: 12 (863) Admissions data: 12 (863)	Yes	9	Use of corticosteroids within 1 hour of presentation to an ED significantly reduces the need for hospital admission in patients with acute asthma. Benefits appear greatest in patients with more severe asthma, and those not currently receiving steroids. Children appear to respond well to oral steroids.
Rowe, 2007, 6677	To determine the benefit of corticosteroids (oral, intramuscular, or intravenous) for the treatment of asthmatic patients discharged from an acute care setting (i.e. usually the emergency department) after assessment and treatment of an acute asthmatic exacerbation.	Asthmatic exacerbations	Outpatient oral or Intramuscular corticosteroid vs. Placebo	Total: 6 (374) Admissions data: 4 (210)	No	8	A short course of corticosteroids following assessment for an asthma exacerbation significantly reduces the number of relapses to additional care, hospitalizations and use of short-acting beta2-agonist without an apparent increase in side effects. Intramuscular and oral corticosteroids are both effective.
Saab, 2015, 8884	The aim of this study was to evaluate the efficacy of probiotics in the management of minimal hepatic encephalopathy HE (MHE) and overt HE (OHE) in comparison to no treatment/placebo and lactulose.	Hepatic encephalopathy	Probiotics vs. No treatment, placebo, or lactulose	Total: 14 (1152) Admissions data: 5 (524)	Yes	4	Overall the use of probiotics was more effective in decreasing hospitalization rates, improving MHE and preventing progression to OHE in patients with underlying MHE than placebo, but similar to that seen with lactulose. The use of probiotics did not affect mortality rates.
Saha, 2007, 6746	We, therefore, conducted a systematic review and meta-analysis of randomized placebo-controlled clinical trials to evaluate the role of tissue ACE inhibitors for secondary prevention of cardiovascular events in patients with preserved ventricular function.	Patients with preserved left ventricular function	Tissue angiotensin-converting enzyme inhibitors vs. Placebo	Total: 4 (31555) Admissions data: 4 (31555)	No	4	Tissue ACE inhibitors have demonstrated benefit when used for secondary prevention of cardiovascular disease in patients with preserved left ventricular function in randomized placebo-controlled clinical trials.
Saha , 2008, 6747	The aim of this study was to determine the role of tissue angiotensin-converting enzyme (ACE) inhibitors in the prevention of cardiovascular disease in patients with diabetes mellitus without left ventricular systolic dysfunction or clinical evidence of heart failure in randomized placebo-controlled clinical trials using pooled meta-analysis techniques.	Patients with diabetes mellitus without left ventricular systolic dysfunction or clinical evidence of heart failure	Tissue angiotensin-converting enzyme inhibitors vs. Placebo	Total: 4 (10328) Admissions data: 4 (9991)	No	4	Pooled meta-analysis of randomized placebo-controlled trials suggests that tissue ACE inhibitors modestly reduce the risk of myocardial infarction and cardiovascular death and tend to reduce overall mortality in diabetic patients without left ventricular systolic dysfunction or heart failure.
Salpeter, 2006, 6795	The objective of our study was to assess the effect of long-acting -agonists on severe asthma exacerbations requiring hospitalization, life-threatening asthma attacks, and asthma-related deaths. We used subgroup analyses to compare results for salmeterol and formoterol and for children and adults.	Asthma	Long acting beta agonists vs. Placebo	Total: 47 (unclear) Admissions data: 9 (3772)	Yes	6	Long-acting beta-agonists have been shown to increase severe and life-threatening asthma exacerbations, as well as asthma related deaths.

Salpeter, 2006, 6797	The objective of this meta-analysis is to compare the effects of b2-agonists and anticholinergics on exacerbations requiring withdrawal from the trial, severe exacerbations requiring hospitalization, and respiratory deaths in patients with COPD.	Chronic obstructive pulmonary disease	B2-agonists or anticholinergics vs. Placebo or anticholinergics	Total: 22 (15276)<br><br>Admissions data: unclear (unclear)	Yes	6	Inhaled anticholinergics significantly reduced severe exacerbations and respiratory deaths in patients with COPD, while b2-agonists were associated with an increased risk for respiratory deaths. This suggests that anticholinergics should be the bronchodilator of choice in patients with COPD, and b2-agonists may be associated with worsening of disease control
Sampson, 2013, 6806	To review the effects of different intermittent drug techniques compared with maintenance treatment (as defined by the trial authors) in people with schizophrenia or related disorders.	Schizophrenia and other types of schizophrenia-like psychoses	Any intermittent drug technique vs. Maintenance therapy	Total: 17 (2252)<br><br>Admissions data: 6 (661)	Yes	11	Results of this reviewsupport the existing evidence that intermittent antipsychotic treatment is not as effective as continuous,maintained antipsychotic therapy in preventing relapse in people with schizophrenia. More research is needed to assess any potential benefits or harm of intermittent treatment regarding adverse effects typically associated with maintained antipsychotic treatment, as well as any cost-effectiveness of this experimental treatment.
Shan, 2013, 7029	We therefore aimed to undertake a systematic review and meta-analysis of both intravenous and nebulized magnesium sulfate to determine their roles in adults and children with acute asthma.	Asthma	Intravenous or nebulized magnesium sulfate as an adjuvant in combination with b2-agonists vs. B2-agonists & placebo	Total: 25 (1754)<br><br>Admissions data: 6 (383)	Yes	6	The use of intravenous magnesium sulfate, in addition to b2-agonists and systemic steroids, in the treatment of acute asthma appears to produce benefits with respect to improve pulmonary function and reduce the number of hospital admissions for children, and only improve pulmonary function for adults. However, the use of nebulized magnesium sulfate just appears to produce benefits for adults.
Stratton, 2013, 7484	Therefore, this systematic review aimed to critically reviewand synthesise the literature to assess the impact of oral nutri-tional supplements used in the community setting across all patientgroups on hospital admissions and readmissions (indicated as (re)admissions).	Malnourished patients being discharged from the hospital to the community	Oral nutritional supplements vs. Usual care or dietary counselling	Total: 9 (1190)<br><br>Admissions data: 6 (852)	Yes	6	This systematic review shows that ONS significantly reduce hospital (re)admissions, particularly in older patient groups, with economic implications for health care.
Su, 2014, 7504	In order to provide a scientific basis for clinical use of nitroprusside, it is necessary to evaluate the drug efficacy and safety in preventing no-flow using the method of the Cochrane systematic review.	Acute myocardial infarction	Nitroprusside vs. Placebo (including the blank control, saline, and nitroglycerin)	Total: 4 (169)<br><br>Admissions data: 2 (190)	No	6	The results of the meta-analyses showed that intracoronary nitroprusside is beneficial in preventing no-reflow/slow-flow, in reducing corrected TIMI frame count, and in improving left ventricular ejection fraction. It also likely reduces adverse reactions in patients after PCI and rehospitalization due to cardiovascular events.
Susantitaphon g, 2013, 7543	The Canadian Heart and Stroke Foundation clinical guidelines now recommend that combined RAAS blockade therapy be discontinued for the treatment of hypertension.21 In light of scarce data on the potentially deleterious effect of combined RAAS blockade therapy on kidney-related endpoints in patients with CKD, we conducted a meta-analysis of all randomized controlled trials (RCTs) comparing the efficacy and safety of combined vs. single RAAS blockade therapy in patients with CKD.	Chronic kidney disease	Combined renin angiotensin aldosterone system blockade therapy vs. Single renin-angiotensin aldosterone system blockade	Total: 59 (4975)<br><br>Admissions data: 5 (326)	No	7	Although combined RAAS blockade therapy in CKD is associated with a decrease in albuminuria and proteinuria, it is associated with a decrease in GFR and a higher incidence of hyperkalemia and hypotension relative to monotherapy. The potential long-term kidne benefits of combined RAAS blockade therapy require further study.
Taylor, 2001, 7647	To examine the benefits and risks of long term anticoagulation (warfarin) compared with antiplatelet treatment (aspirin/indoprofen) in patients with non-rheumatic atrial fibrillation.	Nonrheumatic atrial fibrillation	Long term anticoagulation vs. Antiplatelet treatment	Total: 5 (3298)<br><br>Admissions data: 5 (3298)	No	6	The heterogeneity between the trials and the limited data result in considerable uncertainty about the value of long term anticoagulation compared with antiplatelet treatment. The risks of bleeding and the higher cost of anticoagulation make it an even less convincing treatment option.
Tonelli, 2008, 7787	The aim of this review was to assess the evidence for clinical efficacy and harms and the economic implications of ESA use in adult patients with anemia of CKD.	Patients with anemia of chronic kidney disease	Erythropoiesis-Stimulating Agents (Epoetin (alpha and beta) or darbepoetin) targeting high haemoglobin vs.	Total: 10 (1553)<br><br>Admissions data: 4 (3143)	No	11	In an environment where decision makers are willing to reimburse ESA, our base case analysis suggests that treatment to a target Hb of 110 g/L is most likely to be cost-effective. This strategy, however, will lead to higher costs (mainly due to ESA acquisition) compared to the low Hb target strategy, and it is based on the assumption that the intermediate target will improve QoL

			Intermediate or low target haemoglobin protocols				compared with the low target, which is unproven. Given the generally modest clinical benefit of ESA and the direct relationship between dose and cost, it may be prudent to consider a maximum ESA dose (above which the dose would not be increased further, even if the Hb target were not reached). Future research should focus on this comparison. In the interim, decision makers might reasonably choose to reimburse only the low Hb strategy because of the uncertainty in the quality-of-life gains associated with the intermediate strategy. Because of the higher cost of IV epoetin, the merits of reimbursing only SC epoetin (or darbopoetin by either route) should be explored. Lastly, because even small differences in potency per unit cost of ESA can translate into large differences in total costs, head-tohead comparisons of epoetin and darbepoetin should be considered.
Walters, 2014, 8704	To assess the effects of corticosteroids administered orally or parenterally for treatment of acute exacerbations of COPD, and to compare the efficacy of parenteral versus oral administration	Chronic obstructive pulmonary disease	Intravenous corticosteroid vs. Oral corticosteroid	Total: 20 (2078) Admissions data: 5 (unclear)	Yes	10	There is high-quality evidence to support treatment of exacerbations of COPD with systemic corticosteroid by the oral or parenteral route in reducing the likelihood of treatment failure and relapse by one month, shortening length of stay in hospital inpatients not requiring assisted ventilation in ICU and giving earlier improvement in lung function and symptoms. There is no evidence of benefit for parenteral treatment compared with oral treatment with corticosteroid on treatment failure, relapse or mortality. There is an increase in adverse drug effects with corticosteroid treatment, which is greater with parenteral administration compared with oral treatment.
Wilt, 2007, 8367	To evaluate the effectiveness of COPD management strategies.	Stable chronic obstructive pulmonary disease	1) inhaled medications (2-agonists, anticholinergics, combination 2-agonists and anticholinergics, inhaled corticosteroids, and combination inhaled corticosteroids and long-acting 2-agonists or anticholinergics), 2) pulmonary rehabilitation, 3) disease management programs, and 4) oxygen therapy. vs. Placebo; long-acting beta2-agonists; inhaled corticosteroids; tiotropium	Total: 42 (41932) Admissions data: 12 (20540)	No	8	Long-acting inhaled therapies, supplemental oxygen, and pulmonary rehabilitation are beneficial in adults who have bothersome respiratory symptoms, especially dyspnea, and FEV1 less than 60% predicted.
Xie, 2016, 8916	Renin–angiotensin–aldosterone system(RAAS) blockers are effective therapies for heart failure and reduced ejection fraction (HFrEF) or left ventricular dysfunction (LVD). Weaimed to assess the efficacy and safety of RAAS blockers in these patients.	Heart failure with reduced ejection fraction or left ventricular dysfunction	Renin–angiotensin–aldosterone system blockade: Angiotensin converting enzyme inhibitor; Angiotensin II receptor blocker; Aldosterone antagonists ; Direct renin inhibitor vs. Angiotensin-converting enzyme inhibitor; angiotensin II receptor blockers	Total: 21 (69229) Admissions data: 20 (68976)	No	5	ARNI has the highest probability of being the most efficacious therapy for HFrEF in reducing death and hospitalization for heart failure. ARA has themost favorable benefit–risk profile as an adjunct to background ACEI and/or ARB therapy.

Yang, 2015, 8918	To assess the use of current prophylactic antibiotics in patients undergoing TRPB, we conducted a systematic review and meta-analysis to clarify which type, dose, route, and duration of antibiotics are the most appropriate and effective.	Prostate bopsy	Short course, single dose, or oral prophylactic antibiotics vs. Long course, multiple dose, systemically administered, or combined prophylactic antibiotics	Total: 22 (3846) Admissions data: 13 (2739)	No	8	Prophylactic antibiotics could be beneficial for the reduction of infective complications after TRPB. Single-dose or short-course oral administration with any type of antibiotic appears to be optimal. One additional type of antibiotic added to the basic antibiotic agent may contribute to the minimization of severe infection and drug resistance.
Yohannes, 2011, 8511	Therefore, we undertook a meta-analysis of randomized controlled trials (RCTs) to evaluate the efficacy of tiotropium on clinically relevant outcomes to patients with COPD, including health- related quality of life, dyspnea, adverse events, and clini- cal events (COPD exacerbations and related hospitaliza- tions) compared to placebo, ipratropium bromide, and long- acting □2 agonists (LABA).	Chronic obstructive pulmonary disease	Tiotropium vs. Placebo, ipratropium bromide, or long-acting beta agonists (LABA, salmeterol, or formoterol)	Total: 16 (16301) Admissions data: 9 (12379)	Yes	7	)In stable COPD, tiotropium showed superior efficacy in improving quality of life and dyspnea, compared to placebo and ipratropium. However, tiotropium's differences with salmeterol were less clear.
Zhang, 2011, 8604	This study aimed to investigate the effect of statins on clinical outcomes of chronic systolic heart failure (CHF) by a meta- analysis based on randomized controlled trials (RCTs).	Chronic systolic heart failure	Statins vs. Placebo or blank in addition to contemporary standard therapy of CHF (no statin treatment).	Total: 13 (10447) Admissions data: 11 (10135)	No	6	Although statin has little impact on clinical outcomes in overall CHF patients, statin administration if needed is feasible to CHF patients, and the treatment might be effective when restricted to specific statins or populations.
Zhang, 2016, 8922	This meta-analysis was designed to assess the role of RAAS inhibitors on mortality, hospitalization, diastolic function, and exercise capacity in patients with HFpEF.	Heart failure with preserved ejection fraction (defined as signs or symptoms of heart failure with an EF > 40 %)	Renin–angiotensin–aldosterone system inhibitors vs. Placebo or diuretics	Total: 13 (12532) Admissions data: 11 (12308)	No	5	This meta-analysis shows that RAAS inhibitors could significantly reduce heart failure-related hospitalization and improve the E/e' index in patients with HFpEF. Further large-scale randomized controlled trials, especially on diastolic function, are needed to confirm the effects of RAAS inhibitors in patients with HFpEF.
Zhang, 2014, 9143	In this systematic review and meta-analysis, we weighed the relative effect size of montelukast for preventing asthma exacerbations under different standard teratment conditions and determined whether montelukast is effective in the treatment of acute asthma in adults	Chronic asthma or acute asthma	Montelukast and long acting beta 2 agonists vs. Long acting beta 2 agonists alone	Total: 26 (unclear) Admissions data: unclear (unclear)	No	6	Montelukast had low risk in hoarseness and insomnia. Our meta-analysis suggests that montelukast significantly reduces mild, moderate, and part of severe exacerbations in chronic mild to moderate asthma, but it has inferior efficacy to ICS or ICS plus LABA.
Zhou, 2014, 8928	We therefore performed a meta-analysis of randomized controlled trials (RCTs) to evaluate the effects of TMZ treatment in CHF patients.	Chronic heart failure	Trimetazidine vs. Placebo	Total: 19 (1042) Admissions data: 4 (130)	No	4	TMZ treatment in CHF patients may improve clinical symptoms and cardiac function, reduce hospitalization for cardiac causes, and decrease serum levels of BNP and CRP.
Ziff, 2015, 8931	To clarify the impact of digoxin on death and clinical outcomes across all observational and randomised controlled trials, accounting for study designs and methods.	Not specified (RCTs were all heart failure patients)	Digoxin vs. Placebo	Total: 7 (8406) Admissions data: 2 (7788)	No	7	Digoxin is associated with a neutral effect on mortality in randomised trials and a lower rate of admissions to hospital across all study types. Regardless of statistical analysis, prescription biases limit the value of observational data.
Zou, 2016, 8933	This meta-analysis assessed the efficacy of aclidinium bromide with respect to clinical events, health-related quality of life and symptom scales, pulmonary function, and safety among patients with stable COPD.	Moderate-to-severe chronic obstructive pulmonary disease	Aclidinium Bromide vs. Placebo	Total: 7 (7001) Admissions data: 7 (2601)	Yes	8	Aclidinium reduced the incidence of exacerbation-related hospitalizations and improved quality of life, COPD symptoms and pulmonary function. In addition, aclidinium did not increase the incidence of non-fatal serious adverse events, cardiac adverse events, or COPD exacerbations and was not associated with increased mortality.

Table S5. Full Results for AMSTAR Review Quality Assessment

Author	Year	Review ID	Criteria 1<br>Was an a priori design provided?	Criteria 2<br>Was there duplicate study selection and extraction?	Criteria 3<br>Was a comprehensive literature search performed?	Criteria 4<br>Was there a search for grey or unpublished literature?	Criteria 5<br>Was a list of included and excluded studies provided?	Criteria 6<br>Were the characteristics of included studies provided?	Criteria 7<br>Was the scientific quality of included studies assessed and documented?	Criteria 8<br>Was the scientific quality of included studies used appropriately in formulating conclusions?	Criteria 9<br>Were the methods used to combine the results of included studies appropriate?	Criteria 10<br>Was the likelihood of publication bias assessed?	Criteria 11<br>Were conflicts of interest acknowledged?	Overall<br>Summary AMSTAR score
Afilalo	2007	100	No	Yes	Yes	Yes	No	Yes	Yes	No	Yes	No	No	6
Ahmed	2014	129	No	Yes	Yes	No	No	Yes	Yes	Yes	Yes	No	No	6
Akioyamen	2016	8711	Yes	No	Yes	No	No	Yes	Yes	Yes	Yes	Yes	No	7
Anderson	2015	8714	Yes	Yes	Yes	Yes	Yes	Yes	Yes	Yes	Yes	Yes	Yes	11
Assasi	2009	378	Yes	No	Yes	Yes	Yes	Yes	Yes	Yes	Yes	No	Yes	9
Badve	2011	446	No	No	Yes	Yes	No	Yes	No	Yes	Yes	No	No	5
Baigent	2013	1653	No	Yes	Yes	Yes	No	No	Yes	Yes	Yes	No	No	6
Barr	2006	548	Yes	Yes	Yes	Yes	Yes	Yes	Yes	Yes	Yes	Yes	No	10
Baumeister	2011	593	Yes	Yes	Yes	Yes	Yes	Yes	Yes	Yes	Yes	Yes	Yes	11
Beck	2013	615	No	No	No	No	Yes	Yes	Yes	Yes	Yes	No	No	5
Blitz	2005	813	No	No	Yes	Yes	No	Yes	Yes	No	Yes	No	No	5
Bonsu	2016	8729	Yes	Yes	Yes	Yes	No	Yes	Yes	Yes	Yes	Yes	No	9
Briasoulis	2015	8731	No	No	Yes	No	No	Yes	Yes	Yes	Yes	Yes	No	6
Brophy	2001	1021	No	No	No	No	No	Yes	Yes	Yes	Yes	No	No	4
Burch	2009	1080	Yes	No	Yes	Yes	Yes	Yes	Yes	Yes	Yes	No	No	8
Cammarano	2016	8735	No	No	No	No	No	Yes	Yes	Yes	Yes	No	No	4
Campschroer	2014	1164	Yes	Yes	Yes	Yes	Yes	Yes	Yes	Yes	Yes	Yes	No	10
Cates	2013	1249	Yes	No	Yes	Yes	Yes	Yes	Yes	Yes	Yes	Yes	No	9
Cawood	2012	1264	No	No	Yes	Yes	Yes	Yes	Yes	Yes	Yes	No	No	7
Ceron-Litvoc	2009	1281	No	No	Yes	Yes	Yes	No	Yes	No	Yes	No	No	5
Chauhan	2012	1348	Yes	Yes	Yes	Yes	Yes	Yes	Yes	Yes	Yes	Yes	Yes	11
Chauhan	2014	8681	Yes	No	Yes	Yes	Yes	Yes	Yes	Yes	Yes	Yes	No	9
Chen	2015	8742	Yes	No	Yes	Yes	No	Yes	Yes	Yes	Yes	Yes	No	8
Cheyne	2015	8744	Yes	Yes	Yes	Yes	Yes	Yes	Yes	Yes	Yes	No	Yes	10
Chong	2012	1438	Yes	Yes	Yes	Yes	Yes	Yes	Yes	Yes	Yes	No	Yes	10
Coeytaux	2014	8747	Yes	No	No	No	No	Yes	No	Yes	Yes	Yes	No	5
Cordina	2005	1606	No	No	Yes	Yes	Yes	Yes	Yes	Yes	Yes	Yes	No	8
Costa	2013	1629	Yes	No	Yes	Yes	No	Yes	Yes	Yes	Yes	Yes	No	8
Danchin	2006	1752	Yes	No	Yes	Yes	No	Yes	Yes	Yes	Yes	Yes	No	8
DiNicolantonio	2013	1991	No	No	Yes	No	Yes	Yes	Yes	No	Yes	No	No	5
Doyle	2009	2055	Yes	No	Yes	Yes	No	Yes	Yes	Yes	Yes	Yes	No	8
Ducharme	2010	2095	Yes	Yes	Yes	Yes	Yes	Yes	Yes	Yes	Yes	Yes	Yes	11
Ebell	2013	2148	Yes	Yes	Yes	Yes	Yes	Yes	Yes	No	Yes	No	Yes	9
Edmonds	2012	2158	Yes	Yes	Yes	Yes	Yes	Yes	Yes	Yes	Yes	No	No	9
Edmonds	2012	2159	Yes	Yes	Yes	Yes	Yes	Yes	Yes	No	Yes	Yes	No	9
Ezekowitz	2009	2304	No	No	Yes	Yes	No	Yes	Yes	No	Yes	Yes	No	6

Farne	2015	8767	Yes	Yes	Yes	Yes	Yes	Yes	Yes	Yes	Yes	No	Yes	10
Filippini	2003	2402	Yes	Yes	No	Yes	No	Yes	Yes	Yes	Yes	No	No	7
Fisher	2014	8769	Yes	Yes	Yes	Yes	Yes	Yes	Yes	Yes	Yes	Yes	Yes	11
Fisher	2015	8770	No	Yes	No	No	No	Yes	Yes	Yes	Yes	Yes	Yes	7
Fisher	2015	8771	Yes	Yes	Yes	Yes	Yes	Yes	Yes	Yes	Yes	Yes	Yes	11
Fox	2011	2496	No	No	Yes	No	No	Yes	Yes	Yes	Yes	No	No	5
Fu	2012	2549	No	Yes	Yes	Yes	No	No	Yes	Yes	Yes	Yes	No	7
Gandhi	2014	2599	No	Yes	Yes	Yes	No	Yes	Yes	Yes	Yes	Yes	No	8
Gao	2014	8775	No	Yes	No	No	No	Yes	Yes	Yes	Yes	Yes	No	6
Garside	2007	2640	Yes	Yes	Yes	Yes	Yes	Yes	Yes	Yes	Yes	No	Yes	10
Grimwade	2003	2933	Yes	Yes	Yes	Yes	Yes	Yes	Yes	Yes	Yes	No	No	9
Grooten	2015	8780	No	No	Yes	Yes	No	Yes	Yes	Yes	Yes	Yes	No	7
Hemkens	2016	8784	Yes	Yes	Yes	Yes	Yes	Yes	Yes	Yes	Yes	Yes	Yes	11
Heran	2012	3275	Yes	Yes	Yes	Yes	Yes	Yes	Yes	Yes	Yes	Yes	No	10
Hood	2014	3415	Yes	No	Yes	Yes	Yes	Yes	Yes	No	Yes	No	No	7
Jaeschke	2008	3639	No	Yes	Yes	Yes	Yes	Yes	No	No	No	No	No	5
Jaeschke	2008	3640	No	Yes	No	Yes	No	Yes	No	No	Yes	No	No	4
James	2008	3657	No	Yes	Yes	Yes	No	Yes	Yes	No	Yes	Yes	No	7
Jankowska	2016	8790	No	No	Yes	Yes	No	Yes	Yes	Yes	Yes	No	No	6
Jefferson	2014	3696	Yes	Yes	Yes	Yes	Yes	Yes	Yes	Yes	Yes	Yes	Yes	11
Jong	2002	3781	Yes	No	Yes	No	No	Yes	Yes	No	Yes	Yes	No	6
Kang	2007	3877	No	No	Yes	No	Yes	Yes	Yes	No	Yes	Yes	No	6
Kang	2008	3879	No	Yes	Yes	No	Yes	Yes	Yes	No	Yes	Yes	No	7
Kansagara	2013	3885	No	Yes	Yes	Yes	No	Yes	Yes	Yes	Yes	Yes	No	8
Karner	2011	3908	Yes	Yes	Yes	Yes	Yes	Yes	Yes	Yes	Yes	No	Yes	10
Karner	2014	8692	Yes	Yes	Yes	Yes	Yes	Yes	Yes	Yes	Yes	Yes	No	10
Kaur	2014	3937	No	Yes	Yes	Yes	No	Yes	Yes	No	Yes	Yes	No	7
Kew	2014	4002	Yes	Yes	Yes	Yes	Yes	Yes	Yes	Yes	Yes	No	Yes	10
Kew	2013	4003	Yes	Yes	Yes	Yes	Yes	Yes	Yes	Yes	Yes	Yes	Yes	11
Kew	2014	4004	Yes	Yes	Yes	Yes	Yes	Yes	Yes	Yes	Yes	No	Yes	10
Kew	2016	8802	Yes	No	Yes	Yes	Yes	Yes	Yes	Yes	Yes	Yes	Yes	10
Kew	2015	8803	Yes	Yes	Yes	Yes	Yes	Yes	Yes	Yes	Yes	Yes	Yes	11
Kew	2015	8805	Yes	Yes	Yes	Yes	Yes	Yes	Yes	Yes	Yes	Yes	Yes	11
Kishimoto	2013	4065	No	No	Yes	Yes	No	Yes	No	Yes	Yes	Yes	No	6
Kishimoto	2014	4067	No	Yes	Yes	Yes	No	Yes	Yes	Yes	Yes	Yes	No	8
Komossa	2010	4123	Yes	Yes	Yes	Yes	Yes	Yes	Yes	Yes	Yes	No	No	9
Kuenzli	2010	4206	No	Yes	Yes	Yes	No	Yes	Yes	Yes	Yes	No	No	7
Kumar	2014	8815	No	No	No	No	No	Yes	Yes	Yes	Yes	Yes	Yes	6
Kuswardhani	2011	4239	No	No	Yes	No	No	Yes	Yes	Yes	Yes	Yes	No	6
La Mantia	2010	4260	Yes	Yes	Yes	Yes	Yes	Yes	Yes	Yes	Yes	No	No	9
Lasserson	2011	4362	Yes	Yes	Yes	Yes	Yes	Yes	Yes	Yes	Yes	No	No	9
Le	2016	8817	No	Yes	Yes	Yes	No	Yes	Yes	Yes	Yes	Yes	No	8
Leucht	2011	4505	No	No	Yes	Yes	No	Yes	Yes	Yes	Yes	Yes	No	7
Leucht	2012	4508	Yes	Yes	Yes	Yes	Yes	Yes	Yes	Yes	Yes	Yes	Yes	11
Levy	2010	4518	No	No	No	No	No	Yes	Yes	No	Yes	No	No	3
Li	2016	8822	No	Yes	Yes	Yes	No	Yes	Yes	Yes	Yes	Yes	Yes	9

Liew	2014	4577	No	Yes	No	No	No	Yes	Yes	No	Yes	No	No	4
Lipinski	2009	4615	No	Yes	Yes	Yes	No	Yes	Yes	No	Yes	Yes	No	7
Liu	2014	4629	No	Yes	No	No	No	Yes	No	No	Yes	Yes	No	4
Liu	2014	8826	No	Yes	Yes	No	No	Yes	Yes	Yes	Yes	Yes	No	7
Liu	2014	8827	No	No	Yes	Yes	No	Yes	Yes	Yes	Yes	Yes	No	7
Lopez	2015	8829	Yes	Yes	Yes	Yes	No	Yes	Yes	Yes	Yes	No	No	8
Magee	2003	4812	Yes	Yes	Yes	Yes	Yes	Yes	Yes	Yes	Yes	No	No	9
Magee	2000	4815	No	Yes	Yes	Yes	Yes	Yes	Yes	Yes	Yes	Yes	No	9
Makani	2013	4838	No	Yes	No	No	No	Yes	Yes	Yes	Yes	Yes	No	6
Makrides	2014	4844	Yes	Yes	Yes	Yes	Yes	Yes	Yes	Yes	Yes	No	No	9
Mannucci	2008	4889	No	No	Yes	Yes	No	Yes	Yes	No	Yes	No	No	5
Medic	2016	8842	Yes	No	Yes	Yes	No	Yes	Yes	Yes	Yes	No	No	7
Mohammed	2007	5325	No	No	Yes	Yes	No	No	Yes	Yes	Yes	No	Yes	6
Møiniche	2003	5330	No	No	Yes	No	Yes	No	No	No	Yes	No	No	3
Moore	2006	5371	No	No	Yes	Yes	No	Yes	Yes	Yes	Yes	No	No	6
Nair	2012	5515	Yes	Yes	Yes	Yes	Yes	Yes	Yes	Yes	Yes	No	No	9
Nannini	2013	5533	Yes	Yes	Yes	Yes	Yes	Yes	Yes	Yes	Yes	Yes	No	10
Nannini	2013	8694	Yes	Yes	Yes	Yes	Yes	Yes	Yes	Yes	Yes	Yes	No	10
Ngo	2010	5606	Yes	Yes	Yes	Yes	Yes	Yes	Yes	Yes	Yes	Yes	No	10
Ni	2009	5611	Yes	No	Yes	Yes	Yes	Yes	Yes	Yes	Yes	Yes	No	9
Ni	2014	8850	Yes	Yes	Yes	Yes	Yes	Yes	Yes	Yes	Yes	Yes	Yes	11
Ni	2015	9197	No	Yes	No	No	No	Yes	Yes	Yes	Yes	Yes	No	6
Pizzi	2011	6161	No	No	Yes	Yes	Yes	Yes	Yes	Yes	Yes	Yes	Yes	9
Poole	2015	8867	Yes	No	Yes	Yes	Yes	Yes	Yes	Yes	Yes	Yes	Yes	10
Powell	2015	8868	Yes	Yes	Yes	Yes	Yes	Yes	Yes	Yes	Yes	Yes	Yes	11
Rajagopalan	2011	6347	No	Yes	Yes	No	Yes	Yes	Yes	Yes	Yes	Yes	No	8
Renner	2012	6442	Yes	Yes	Yes	Yes	Yes	Yes	Yes	Yes	Yes	Yes	Yes	11
Rodrigo	1999	6564	No	Yes	Yes	No	Yes	Yes	Yes	Yes	Yes	Yes	No	8
Rodrigo	2000	6566	No	No	Yes	Yes	No	Yes	Yes	Yes	Yes	Yes	No	7
Rodrigo	2005	6568	No	No	Yes	Yes	No	Yes	Yes	Yes	Yes	No	No	6
Rodrigo	2002	6576	No	No	Yes	No	Yes	Yes	Yes	Yes	Yes	No	No	6
Rodrigo	2003	6578	No	No	Yes	Yes	Yes	Yes	Yes	Yes	Yes	No	No	7
Rowe	2000	6670	Yes	Yes	Yes	Yes	No	Yes	Yes	Yes	Yes	No	No	8
Rowe	1992	6672	No	No	Yes	Yes	No	Yes	Yes	Yes	Yes	No	No	6
Rowe	2001	6676	Yes	Yes	Yes	Yes	Yes	Yes	Yes	Yes	Yes	No	No	9
Rowe	2007	6677	Yes	No	Yes	Yes	Yes	Yes	Yes	Yes	Yes	No	No	8
Saab	2015	8884	No	No	No	No	No	Yes	Yes	Yes	Yes	No	No	4
Saha	2007	6746	No	No	No	No	No	Yes	Yes	Yes	Yes	No	No	4
Saha	2008	6747	No	No	No	No	No	Yes	Yes	Yes	Yes	No	No	4
Salpeter	2006	6795	No	No	Yes	Yes	No	Yes	No	No	Yes	Yes	Yes	6
Salpeter	2006	6797	No	No	Yes	Yes	No	Yes	No	No	Yes	Yes	Yes	6
Sampson	2013	6806	Yes	Yes	Yes	Yes	Yes	Yes	Yes	Yes	Yes	Yes	Yes	11
Shan	2013	7029	No	Yes	Yes	No	No	Yes	Yes	No	Yes	Yes	No	6
Stratton	2013	7484	No	No	Yes	Yes	No	Yes	Yes	Yes	Yes	No	No	6
Su	2014	7504	No	Yes	Yes	No	No	Yes	Yes	Yes	Yes	No	No	6
Susantitaphong	2013	7543	No	No	Yes	Yes	No	Yes	Yes	Yes	Yes	Yes	No	7

Taylor	2001	7647	No	No	Yes	Yes	No	Yes	Yes	Yes	Yes	No	No	6
Tonelli	2008	7787	Yes	Yes	Yes	Yes	Yes	Yes	Yes	Yes	Yes	Yes	Yes	11
Walters	2014	8704	Yes	Yes	Yes	Yes	Yes	Yes	Yes	Yes	Yes	Yes	No	10
Wilt	2007	8367	Yes	Yes	Yes	Yes	No	Yes	Yes	Yes	No	No	Yes	8
Xie	2016	8916	No	No	No	No	Yes	Yes	Yes	Yes	Yes	No	No	5
Yang	2015	8918	Yes	Yes	Yes	Yes	No	Yes	Yes	Yes	Yes	No	No	8
Yohannes	2011	8511	No	Yes	Yes	Yes	No	Yes	Yes	Yes	Yes	No	No	7
Zhang	2011	8604	No	Yes	No	No	No	Yes	Yes	Yes	Yes	Yes	No	6
Zhang	2016	8922	No	No	No	No	No	Yes	Yes	Yes	Yes	Yes	No	5
Zhang	2014	9143	No	Yes	Yes	No	No	Yes	Yes	Yes	Yes	No	No	6
Zhou	2014	8928	No	No	No	No	No	Yes	Yes	Yes	Yes	No	No	4
Ziff	2015	8931	Yes	No	Yes	Yes	No	No	Yes	Yes	Yes	Yes	No	7
Zou	2016	8933	No	Yes	Yes	Yes	No	Yes	Yes	Yes	Yes	Yes	No	8

Table S6. Distribution of evidence by patient population

**Patient population**[a]<br>Subgroup[b]	Systematic reviews<br>% (n=140)[c]	Number of unique pharmacotherapies investigated<br>% (n=100)[d]	Description of unique pharmacotherapies and the number of reviews investigating them<br>(n=140)
**Diseases of the respiratory system**	**40% (56)**	**22% (22)**	
Other chronic obstructive pulmonary disease	19% (27)	10% (10)	• Adrenergics in combination with anticholinergics (n=2)<br>• Adrenergics in combination with corticosteroids or other drugs, excl. anticholinergics (n=3)<br>• Adrenergics in combination with corticosteroids or other drugs, excl. anticholinergics AND/OR Adrenergics in combination with anticholinergics AND/OR Anticholinergics (n=1)<br>• Adrenergics in combination with corticosteroids or other drugs, excl. anticholinergics AND/OR Anticholinergics (n=1)<br>• Adrenergics in combination with corticosteroids or other drugs, excl. anticholinergics AND/OR Glucocorticoids (n=2)<br>• Anticholinergics (n=10)<br>• Glucocorticoids (n=3)<br>• Macrolides (n=1)<br>• Mucolytics (n=1)<br>• Selective beta-2-adrenoreceptor agonists (n=3)
Status asthmaticus	14% (20)	10% (10)	• Adrenergics in combination with anticholinergics AND/OR Anticholinergics AND/OR Selective beta-2-adrenoreceptor agonists (n=1)<br>• Corticosteroids for systemic use, plain (n=4)[e]<br>• Electrolyte solutions (n=4)<br>• Electrolyte solutions AND/OR Not otherwise specified (n=1)<br>• Glucocorticoids (n=3)<br>• Leukotriene receptor antagonists (n=1)<br>• Medical gases (n=1)<br>• Nebulized magnesium sulfate (n=3)<br>• Selective beta-2-adrenoreceptor agonists (n=2)<br>• Xanthines (n=1)
Asthma (chronic)	10% (14)	7% (7)	• Adrenergics in combination with anticholinergics AND/OR Anticholinergics alone (n=1)<br>• Adrenergics in combination with corticosteroids or other drugs, excl. anticholinergics (n=6)<br>• Anticholinergics (n=2)<br>• Interleukin inhibitors (1)<br>• Leukotriene receptor antagonists (n=2)<br>• Macrolides (n=1)<br>• Selective beta-2-adrenoreceptor agonists (n=1)
Influenza, virus not identified	1% (2)	1% (1)	• Neuraminidase inhibitors (n=2)
Bronchiectasis	<1% (1)	1% (1)	• Macrolides (n=1)
Other interstitial pulmonary diseases	<1% (1)	1% (1)	• Colchicine (n=1)

Diseases of the circulatory system	38% (53)	31% (31)	
Heart failure	25% (35)	18% (18)	• ACE inhibitors, plain (n=3)<br>• ACE inhibitors, plain AND/OR Angiotensin II antagonists, plain (n=6)<br>• ACE inhibitors, plain AND/OR Angiotensin II antagonists, plain AND/OR POTASSIUM-SPARING AGENTS (n=1)<br>• Aldosterone antagonists (n=4)<br>• Alpha and beta blocking agents (n=1)<br>• Alpha and beta blocking agents AND/OR Beta blocking agents, non-selective (n=1)<br>• Alpha and beta blocking agents AND/OR Beta blocking agents, selective (n=1)<br>• Angiotensin II antagonists, plain (n=4)<br>• Angiotensin II antagonists, other combinations (n=1)<br>• Digitalis glycosides (n=3)<br>• HMG CoA reductase inhibitors (n=4)<br>• Iron, parenteral preparations (n=1)<br>• Iron, parenteral preparations AND/OR Other antianemic preparations (n=1)<br>• Other antianemic preparations (n=1)<br>• Other cardiac preparations (n=1)<br>• Renin-inhibitors (n=1)<br>• Sulfonamides, plain (n=1)<br>• Vitamin K antagonists (n=1)
Chronic ischaemic heart disease	6% (9)	9% (9)	• ACE inhibitors, plain (n=2)<br>• Aldosterone antagonists (n=1)<br>• Colony stimulating factors (n=1)<br>• Fibrates (n=1)<br>• Fibrates AND/OR Nicotinic acid and derivatives (n=1)<br>• HMG CoA reductase inhibitors (n=1)<br>• Lipid modifying agents, plain (n=1)[e]<br>• Nicotinic acid and derivatives (n=1)<br>• Other cardiac preparations (n=1)
Other pulmonary heart diseases	4% (5)	4% (4)	• Antihypertensives AND/OR antithrombotic agents AND/OR urologicals (n=2)[e]<br>• Antihypertensives for pulmonary arterial hypertension (n=1)<br>• Drugs used in erectile dysfunction (n=1)<br>• Platelet aggregation inhibitors excl. heparin (n=1)
Acute myocardial infarction	3% (4)	2% (2)	• Bone marrow stem cells (n=3)<br>• Nitroferricyanide derivatives (n=1)
Chronic ischaemic heart disease AND/OR Heart failure	2% (3)	3% (3)	• Aldosterone antagonists (n=2)<br>• Bone marrow stem cells (n=2)<br>• Other antianemic preparations (n=1)
Atrial fibrillation and flutter	2% (3)	3% (3)	• Antiarrhythmics, class III (n=1)<br>• Antiarrhythmics, class Ia AND/OR Antiarrhythmics, class Ic AND/OR Antiarrhythmics, class III AND/OR Other antiarrhythmics, class I and III (n=1)<br>• Vitamin K antagonists (n=1)

Patients at low risk of cardiovascular disease[f]	<1% (1)	1% (1)	• Thiazolidinediones (n=1)
Essential (primary) hypertension	<1% (1)	1% (1)	• Angiotensin II antagonists, plain (n=1)
Acute myocardial infarction AND/OR Heart failure	<1% (1)	1% (1)	• Alpha and beta blocking agents (n=1)
Other acute ischaemic heart diseases	<1% (1)	1% (1)	• HMG CoA reductase inhibitors (n=1)
**Factors influencing health status and contact with health services**	**6% (8)**	**11% (11)**	
Need for immunization against other single viral diseases	1% (2)	2% (2)	• Influenza vaccines (n=1)<br>• Neuraminidase inhibitors (n=1)
Patients receiving special screening examination for neoplasms	3% (4)	4% (4)	• Antibiotics (n=1)<br>• Penicillins with extended spectrum (n=1)<br>• Quinoline derivatives (n=1)<br>• Sulfonamides (n=1)
Patients receiving other medical care	1% (2)	2% (2)	• Colony stimulating factors (n=1)<br>• Other general anesthetics (n=1)
Patients receiving other surgical follow-up care	1% (2)	2% (2)	• Acetic acid derivatives and related substances AND/OR Propionic acid derivatives (n=1)<br>• Opioids(n=1)[e]
Patients receiving care involving dialysis (long term haemodialysis with a central venous catheter)	<1% (1)	1% (1)	• Other antibiotics for topical use (n=1)
**Diseases of the digestive system**	**6% (8)**	**3% (3)**	
Ulcerative colitis	<1% (1)	1% (1)	• Tumor necrosis factor alpha (TNF-α) inhibitors (n=1)
Crohn disease [regional enteritis]	1% (2)	1% (1)	• Tumor necrosis factor alpha (TNF-α) inhibitors (n=2)
Hepatic failure, not elsewhere classified	<1% (1)	1% (1)	• Probiotics (n=1)
Alcoholic liver disease	<1% (1)	1% (1)	• Colchicine (n=1)
Crohn disease [regional enteritis] AND/OR Ulcerative colitis	<1% (1)	1% (1)	• Tumor necrosis factor alpha (TNF-α) inhibitors (n=1)
**Mental and behavioural disorders**	**5% (7)**	**19% (19)**	
Schizophrenia	19% (26)	18% (18)	• Benzamides (n=1)<br>• Benzamides AND/OR Diazepines, oxazepines, thiazepines and oxepines AND/OR Indole derivatives AND/OR Other antipsychotics (n=1)<br>• Butyrophenone derivatives (n=1)<br>• Butyrophenone derivatives AND/OR Diazepines, oxazepines, thiazepines and oxepines AND/OR Other antipsychotics AND/OR Phenothiazines with aliphatic side-chain AND/OR Phenothiazines with piperazine structure (n=1)<br>• Butyrophenone derivatives AND/OR Diazepines, oxazepines, thiazepines and oxepines AND/OR Other antipsychotics AND/OR Phenothiazines with aliphatic side-chain AND/OR Phenothiazines with piperazine structure AND/OR Thioxanthene derivatives (n=1)

			• Butyrophenone derivatives AND/OR Phenothiazines with aliphatic side-chain AND/OR Phenothiazines with piperazine structure (n=2)<br>• Diazepines, oxazepines, thiazepines and oxepines (n=3)<br>• Diazepines, oxazepines, thiazepines and oxepines AND/OR Other antipsychotics (n=2)<br>• Diazepines, oxazepines, thiazepines and oxepines AND/OR Other antipsychotics AND/OR Phenothiazines with aliphatic side-chain (n=1)<br>• Diazepines, oxazepines, thiazepines and oxepines AND/OR Other antipsychotics AND/OR Phenothiazines with aliphatic side-chain AND/OR Phenothiazines with piperazine structure AND/OR Thioxanthene derivatives (n=2)<br>• Diazepines, oxazepines, thiazepines and oxepines AND/OR Other antipsychotics AND/OR Phenothiazines with aliphatic side-chain AND/OR Thioxanthene derivatives (n=1)<br>• Diazepines, oxazepines, thiazepines and oxepines AND/OR Other antipsychotics AND/OR Phenothiazines with piperazine structure AND/OR Thioxanthene derivatives (n=1)<br>• Indole derivatives (n=1)<br>• Other antipsychotics (n=2)<br>• Phenothiazines with aliphatic side-chain (n=1)<br>• Phenothiazines with piperazine structure (n=3)<br>• Phenothiazines with piperazine structure AND/OR Thioxanthene derivatives (n=1)<br>• Thioxanthene derivatives (n=1)
Bipolar affective disorder	<1% (1)	1% (1)	• Carboxamide derivatives (n=1)
**Diseases of the genitourinary system**	**4% (5)**	**5% (5)**	
Chronic kidney disease	2% (3)	3% (3)	• ACE inhibitors, combinations AND/OR Angiotensin II antagonists, combinations AND/OR Diuretics and potassium-sparing agents in combination (n=1)[e]<br>• Beta-blocking agents (n=1)[e]<br>• Other antianemic preparations (n=1)
Disorders resulting from impaired renal tubular function	<1% (1)	1% (1)	• Other anti-parathyroid agents (n=1)
Calculus of kidney and ureter	<1% (1)	1% (1)	• Alpha-adrenoreceptor antagonists (n=1)
**Pregnancy, childbirth and the puerperium**	**3% (4)**	**3% (3)**	
Gestational hypertension with or without significant proteinuria	<1% (1)	1% (1)	• Beta-blocking agents (n=1)[e]
Excessive vomiting in pregnancy	<1% (1)	1% (1)	• Glucocorticoids (n=1)
Gestational hypertension without significant proteinuria	<1% (1)	1% (1)	• Beta-blocking agents (n=1)[e]
Normal or high risk pregnancy[f]	<1% (1)	1% (1)	• Magnesium (n=1)
**Endocrine, nutritional, and metabolic diseases**	**4% (5)**	**5% (5)**	
Diabetes mellitus	<1% (1)	1% (1)	• ACE inhibitors, plain (n=1)
Non-insulin-dependent diabetes mellitus	<1% (1)	1% (1)	• Dipeptidyl peptidase 4 (DPP-4) inhibitor (n=1)
Malnourished patients being discharged from the hospital to the community	<1% (1)	1% (1)	• Other nutrients (n=1)

Older (65+) patients being discharged from the hospital to the community that are malnourished or at risk of malnourishment	<1% (1)	1% (1)	• General nutrients (n=1)
Patients in hospital or community settings that are malnourished or at risk for disease-related malnutrition	<1% (1)	1% (1)	• Protein supplementation (n=1)
**Mixed patient population[e]**	**2% (3)**	**3% (3)**	
Heart failure or Type 1 diabetes mellitus with renal complications	<1% (1)	1% (1)	• ACE inhibitors, plain AND/OR Angiotensin II antagonists, plain AND/OR Renin-inhibitors (n=1)
Patients indicated for treatment with non-steroidal anti-inflammatory drugs	<1% (1)	1% (1)	• Cox-2 inhibitors (n=1)
Alcoholic liver diseases (alcoholic cirrhosis of the liver) or Other interstitial pulmonary diseases (idiopathic pulmonary fibrosis)	<1% (1)	1% (1)	• Colchicine (n=1)
**Multi-morbidity[h]**	**1% (2)**	**2% (2)**	
Chronic ischaemic heart disease and Recurrent depressive disorder	1% (2)	2% (2)	• Selective serotonin reuptake inhibitors (n=1)<br>• Other antidepressants AND/OR Selective serotonin reuptake inhibitors (n=1)
**Diseases of the nervous system**	**1% (2)**	**2% (2)**	
Multiple sclerosis	1% (2)	1% (2)	• Interferons (n=1)<br>• Other immunostimulants (n=1)
**Diseases of the musculoskeletal system and connective tissue**	**<1% (1)**	**1% (1)**	
Systemic lupus erythematosus	<1% (1)	1% (1)	• Selective immunosuppressants (n=1)
**Infectious and parasitic diseases**	**<1% (1)**	**1% (1)**	
Human immunodeficiency virus [HIV] disease[e]	<1% (1)	1% (1)	• Combinations of sulfonamides and trimethoprim, incl. derivatives (n=1)

[a]Patient populations were classified using the World Health Organisations 10th revision of the International Statistical Classification of Diseases and Related Health Problems (ICD 10). [b]These specific classifications represent the summary and 3-character coding level in the ICD 10. [c]Denominator for this column is the number of systematic reviews. [d]One product class defined as a chemical subgroup in the ATC WHO. Only includes products in the intervention group. The numbers in the column do not add up to 100 as some of the products were used in multiple patient populations. [e]Mixed populations were those that were coded by two or more different ICD summary codes. [f]Patient population(s) not captured in codes available in the ICD-10. [g]Pharmacological sub-group classification not possible because review reported limited detail of the medication. Therefore, this code represents the broader classification based on therapeutic or chemical characteristics. [h]Multi-morbid populations were those that were coded by two or more different ICD summary codes.

Table S7. GRADED estimates showing reductions in admissions.

Author, Year, Estimate ID	Population description	Intervention (Event rate)[a]	Comparison (Event rate)[a]	Patients (RCTs)	Patient age[b]	Outcome (follow-up)[c]	Effect (95% CI)[d,e] NNT (95% CI)[e,f] I^2%	Quality of evidence (GRADE)	Notes
**Disease of the circulatory system**									
**Acute myocardial infarction**									
Su, 2014, 7504	Undergoing percutaneous coronary intervention	Nitroprusside (10 per 100)	Placebo (22 per 100)	190 (2)	58 ±6	Readmission for cardiovascular issues (Unclear)	RR: 0.44 (0.21 to 0.91) NNT: 8 (6 to 51) 0%	Low	Summary estimate
**Chronic ischaemic heart disease**									
Danchin, 2006, 1752a	Stable coronary heart disease and absence of heart failure or left ventricular systolic dysfunction	Angiotensin-Converting Enzyme Inhibitor [Ramipril, Perindopril, Trandolapril, or Enalapril] (2 per 100)	Placebo (3 per 100)	31750 (5)	62 ±3	Admission for heart failure (50 ±15 months)	OR: 0.76 (0.66 to 0.88) NNT: 146 (103 to 294) I²: Unclear	Moderate	Summary estimate
Saha , 2007, 6746b	Patients with a prior cardiovascular event or at high risk for cardiovascular events with preserved left ventricular function	Tissue angiotensin converting enzyme Inhibitors [Ramipril, Perindopril, or Trandolapril] (2.5 per 100)	Placebo (3.2 per 100)	29805 (4)	63 ±3	Admission for heart failure (12 months)	RR: 0.78 (0.67 to 0.9) NNT: 140 (93 to 308) I²: Unclear	High	Summary estimate
Afilalo, 2007, 100c	Stable coronary heart disease	Intensive statin therapy [Atorvastatin or Simvastatin] (2 per 100)	Moderate statin therapy [Pravastatin, Atorvastatin or Simvastatin] (3 per 100)	27547 (4)	61 ±2	Admission for heart failure (41 ±20 months)	OR: 0.72 (0.62 to 0.83) NNT: 115 (84 to 189) I²: 4%	Moderate	Summary estimate
**Chronic ischaemic heart disease or Heart failure**									
Le, 2016, 8817a	Heart failure with left ventricular dysfunction (New York Heart Association 1 to 4) and/or post-acute myocardial infarction with Killip scores between I and IV and indicated at least one assessment criteria	Aldosterone Antagonists [Canrenone, Spironolactone, Eplerenone, or Potassium Canrenoate] (20 per 100)	Placebo (21 per 100)	15786 (13)	63 ±6	Hospital admission (15 ±10 months)	RR: 0.93 (0.88 to 0.98) NNT: 67 (39 to 233) I²: 35%	Moderate	Summary estimate
Le, 2016, 8817d	Heart failure with left ventricular dysfunction (New York Heart Association 1 to 4) and/or post-acute myocardial infarction with Killip scores between I and IV and indicated at least one assessment criteria	Aldosterone Antagonists [Canrenone, Spironolactone, Eplerenone, or Potassium Canrenoate] (12 per 100)	Control/placebo (15 per 100)	16730 (12)	64 ±4	Admission for cardiovascular issues (18 ±12 months)	RR: 0.82 (0.72 to 0.92) NNT: 37 (24 to 83) I²: 51%	Low	Summary estimate
Chen, 2015, 8742a	Heart failure and/or previous myocardial infarction with preserved ejection fraction (>= 40%)	Aldosterone antagonists [Eplerenone or Spironolactone] (4.5 per 100)	Placebo or standard care (5.4 per 100)	4551 (3)	Unclear	Admission for heart failure (16 ±16 months)	RR: 0.83 (0.7 to 0.98) NNT: 108 (61 to 920) I²: 0%	Low	Summary estimate

Fisher, 2014, 8769b	Heart failure and/or previous myocardial infarction	Bone marrow stem cells (4 per 100)	No cells (14 per 100)	198 (2)	58 ±4	Readmission for heart failure (36 ±34 months)	RR: 0.26 (0.07 to 0.94) NNT: 10 (8 to 122) I²: 0%	Low	Summary estimate
**Heart failure**									
Xie, 2016, 8916c	Heart failure with reduced ejection fraction or left ventricular dysfunction	Aldosterone antagonists [Canrenone, Eplerenone, or Spironolactone] (11 per 100)	Placebo (15 per 100)	11477 (4)	65 ±3	Admission for heart failure (18 ±5 months)	OR: 0.67 (0.53 to 0.86) NNT: 22 (15 to 54) I²: 74%	Moderate	Summary estimate
Le, 2016, 8817e	Heart failure with left ventricular dysfunction (New York Heart Association 1 to 4)	Aldosterone antagonists [Canrenone, Spironolactone, or Eplerenone] (11 per 100)	Control/placebo (14 per 100)	9040 (9)	66 ±3	Admission for cardiovascular issues (20 ±13 months)	RR: 0.79 (0.68 to 0.91) NNT: 33 (22 to 78) I²: 49%	Low	Sub-group by disease: Heart failure
Xie, 2016, 8916a	Heart failure with reduced ejection fraction or left ventricular dysfunction	Angiotensin converting enzyme inhibitor [Ramipril, Captopril, Enalapril, or Trandolapril] (13 per 100)	Placebo (18 per 100)	12763 (5)	62 ±4	Admission for heart failure (32 ±12 months)	OR: 0.67 (0.61 to 0.74) NNT: 19 (16 to 25) I²: 0%	Moderate	Summary estimate
Xie, 2016, 8916b	Heart failure with reduced ejection fraction or left ventricular dysfunction	Angiotensin II receptor blocker [Candesartan or Valsartan] (18 per 100)	Placebo (23 per 100)	9878 (4)	65 ±2	Admission for heart failure (26 ±15 months)	OR: 0.72 (0.64 to 0.82) NNT: 19 (14 to 30) I²: 30%	Moderate	Summary estimate
Heran, 2012, 3275d	Heart failure with teduced left ventricular ejection fraction (<=40%)	Angiotensin II receptor blocker [Candesartan] (13 per 100)	Placebo (19 per 100)	2590 (3)	66 ±2	Admission for heart failure (14 ±17 months)	RR: 0.71 (0.61 to 0.82) NNT: 18 (14 to 30) I²: 0%	Low	Sub-group by disease severity: Patients with left ventricular ejection fractions less than or equal to 40%
Heran, 2012, 3275l	Heart failure, any ejection fraction	Angiotensin II receptor blocker and angiotensin converting enzyme inhibitor [ARB (Irbesartan or Candesartan) and ACE inhibitor (Enalapril or other)] (10 per 100)	Angiotensin converting enzyme inhibitors [Enalapril or other] (12 per 100)	8108 (4)	63 ±1	Admission for heart failure (18 ±15 months)	RR: 0.81 (0.74 to 0.89) NNT: 43 (31 to 74) I²: 35%	Very low	Summary estimate
Kuenzli, 2010, 4206a	Congestive heart failure with left ventricular dysfunction	Angiotensin II receptor blocker and angiotensin converting enzyme inhibitor [ARB (Candesartan, Valsartan, Irbesartan, or other) and ACE inhibitor (Enalapril, Captopril, or other)] (19 per 100)	Angiotensin converting enzyme inhibitors [Enalapril, Captopril, or other] (24 per 100)	17895 (6)	64 ±2	Readmission for heart failure (23 ±12 months)	RR: 0.81 (0.72 to 0.91) NNT: 22 (15 to 47) I²: 57%	Moderate	Summary estimate
Jong, 2002, 3781d	New York Heart Association functional class 2 to 7	Angiotensin II receptor blocker and angiotensin converting enzyme inhibitor [ARB (Candesartan, Irbesartan, or Valsartan) and an ACE inhibitor] (4.8 per 100)	Angiotensin converting enzyme inhibitors (6.4 per 100)	5560 (3)	Unclear	Admission for heart failure (12 ±10 months)	OR: 0.74 (0.64 to 0.86) NNT: 63 (45 to 118) I²: p>0.1 heterogeneity	Moderate	Sub-group by intervention: Combination therapy of Angiotensin receptor blockers and angiotensin

									converting enzyme inhibitors vs. angiotensin converting enzyme inhibitors alone
Kuenzli, 2010, 4206c	Heart failure with left ventricular dysfunction	Angiotensin II receptor blocker and angiotensin converting enzyme inhibitor [ARB (Candesartan, Valsartan, or Irbesartan) and ACE inhibitor (Enalapril or other)] (19 per 100)	Angiotensin converting enzyme inhibitors [ACE inhibitor Enalapril or other] (23 per 100)	8049 (4)	64 ±2	Readmission for heart failure (22 ±14 months)	RR: 0.81 (0.72 to 0.91) NNT: 23 (15 to 48) I²: 23%	Moderate	Sub-group by disease: Excluding 2 trials with only acute myocardial infarction patients
Makani, 2013, 4838b	Heart failure with reduced ejection fraction (less than or equal to 40%), New York Heart Association class II-IV, wth or without haemodialysis	Angiotensin II receptor blocker and angiotensin converting enzyme inhibitor [ARB (Candesartan, Telmisartan, or Valsartan) and an ACE inhibitor] (37 per 100)	Angiotensin converting enzyme inhibitors (48 per 100)	7890 (3)	63 ±1	Admission for heart failure (36 ±10 months)	RR: 0.77 (0.68 to 0.88) NNT: 9 (7 to 18) I²: 66%	Moderate	Sub-group by disease: Heart failure
Lipinski, 2009, 4615e	Heart failure with reduced ejection fration (less than or equal to 45%) or heart failure hospitalisation in past year	Atorvastatin (10 per 100)	Placebo (28 per 100)	472 (6)	57 ±12	Admission for heart failure (13 ±10 months)	OR: 0.30 (0.18 to 0.49) NNT: 6 (5 to 6) I²: 0%	Very low	Summary estimate
Zhang, 2011, 8604f	Chronic systolic cardiac insufficiency	Atorvastatin (9 per 100)	Placebo or nothing (19 per 100)	524 (8)	55 ±10	Readmission for heart failure (10 ±10 months)	RR: 0.51 (0.33 to 0.81) NNT: 11 (8 to 28) I²: 0%	Low	Sub-group by intervention: Atorvastatin
Liu, 2014, 4629a	Heart failure with preserved ejection fraction (an EF greater than or equal to 40%)	Beta-blocker [Nebivolol or Carvedilol] (20 per 100)	No beta-blockers (28 per 100)	888 (2)	74 ±3	Hospital admission (30 ±12 months)	RR: 0.71 (0.55 to 0.93) NNT: 12 (8 to 51) I²: 7%	Moderate	Summary estimate
Brophy, 2001, 1021	Heart failure with reduced ejection fraction	Beta-blocker [Metoprolol, Bucindolol, Bisoprolol, or Carvedilol] (7 per 100)	Placebo (12 per 100)	10076 (22)	57 ±6	Admission for heart failure (9 ±6 months)	OR: 0.64 (0.53 to 0.79) NNT: 24 (18 to 43) I²: Unclear	Low	Summary estimate
Fisher, 2015, 8770b	Heart failure secondary to ischemic and nonischemic cardiomyopathy	Bone marrow stem cells (4 per 100)	No cells (10 per 100)	173 (5)	Unclear	Readmission for heart failure (26 ±28 months)	RR: 0.39 (0.22 to 0.7) NNT: 16 (13 to 33) I²: 0%	Low	Summary estimate

Hood, 2014, 3415	Normal sinus rhythm with reduced or preserved ejection fractions	Digoxin (9 per 100)	Placebo (12 per 100)	7262 (4)	61 ±3	Hospital admission (12 ±17 months)	OR: 0.68 (0.61 to 0.75) NNT: 28 (23 to 37) I²: 29%	High	Summary estimate
Ziff, 2015, 8931a	Heart failure, any ejection fraction	Digoxin (63 per 100)	Placebo (67 per 100)	7788 (2)	65 ±2	Hospital admission (37 months)	RR: 0.94 (0.9 to 0.99) NNT: 25 (15 to 150) I²: Unclear	Moderate	Summary estimate
Ngo, 2010, 5606	Heart failure with reduced left ejection fraction (less than or equal to 40%) and anemia	Erythropoiesis stimulating agent [Darbepoetin-alpha, Beta-erythropoietin, or Erythropoietin] (16 per 100)	Placebo or no treatment (25 per 100)	734 (9)	72 ±3	Admission for heart failure (7 ±5 months)	RR: 0.62 (0.44 to 0.87) NNT: 11 (7 to 31) I²: 0%	Moderate	Summary estimate
Gandhi, 2014, 2599a	Acute decompensation	Hypertonic saline solution plus intravenous furosemide (19 per 100)	Furosemide alone, with or without dietary restriction of sodium (37 per 100)	2032 (4)	74 ±0.4	Readmission for heart failure (30 ±28 months)	RR: 0.50 (0.33 to 0.76) NNT: 5 (4 to 11) I²: 61%	Moderate	Summary estimate
Jankowska, 2016, 8790a	Heart failure with reduced left ventricular ejection fraction and Iron deficiency	Intravenous iron [Iron sucrose or Ferric carboxymaltose] (6 per 100)	Placebo (20 per 100)	835 (4)	69 ±5	Admission for heart failure (7 ±5 months)	OR: 0.28 (0.16 to 0.5) NNT: 8 (6 to 11) I²: 0%	Moderate	Summary estimate
Bonsu, 2016, 8729a	Heart failure with reduced ejection fraction (<40%)	Lipophilic statin [Atorvastatin, Simvastatin, Pitavastatin] (13 per 100)	Placebo (24 per 100)	1165 (9)	57 ±9	Admission for heart failure (13 ±12 months)	OR: 0.49 (0.36 to 0.67) NNT: 9 (7 to 15) I²: 38%	High	Summary estimate
Liu, 2014, 8826a	Heart failure with reduced ejection fraction (left ventricular ejection fraction less than or equal to 45%)	Lipophilic statin [Atorvastatin, or Simvastatin, Pitavastatin] (6 per 100)	Placebo or no statin (10 per 100)	1087 (9)	58 ±10	Admission for heart failure (13 ±12 months)	RR: 0.60 (0.45 to 0.80) NNT: 25 (18 to 50) I²: 11%	Moderate	Summary estimate
Zhang, 2016, 8922l	Heart failure with preserved ejection fraction (greater than or equal to 40%)	Renin–angiotensin–aldosterone system inhibitors [Perindopril, Eplerenone, Irbesartan, Spironolactone, Irbesartan + diuretics, Ramipril + diuretics, Candesartan, or Quinapril] (12 per 100)	Placebo (14 per 100)	11765 (8)	72 ±4	Admission for heart failure (20 ±17 months)	RR: 0.89 (0.82 to 0.97) NNT: 66 (41 to 244) I²: 0%	Low	Summary estimate
Lipinski, 2009, 4615d	Heart failure with reduced ejection fraction (less than or equal to 45%) or heart failure hospitalisation in past year	Statin [Atorvastatin, Rosuvastatin, or Simvastatin] (19 per 100)	Placebo (25 per 100)	10193 (10)	60 ±11	Admission for heart failure (17 ±15 months)	OR: 0.67 (0.50 to 0.90) NNT: 15 (9 to 51) I²: 64%	Low	Summary estimate
Zhang, 2011, 8604b	Systolic insufficiency	Statin [Atorvastatin, Rosuvastatin, or Simvastatin] (12 per 100)	Placebo or nothing in addition to contemporary standard therapy of CHF (19 per 100)	5561 (10)	59 ±10	Readmission for heart failure (15 ±15 months)	RR: 0.66 (0.44 to 0.98) NNT: 16 (10 to 270) I²: 29%	Low	Sub-group for robustness to trial exclusion: GISSI-HF study excluded (highest weighted)
Zhou, 2014, 8928	Left ventricular ejection fraction less than or equal to 50%	Trimetazidine (7 per 100)	Placebo (15 per 100)	156 (4)	67 ±8	Admission for cardiovascular issues (7 ±4 months)	RR: 0.43 (0.21 to 0.91) NNT: 11 (8 to 72) I²: 0%	Very low	Summary estimate

**Other pulmonary heart diseases**									
Coeytaux, 2014, 8747a	Pulmonary arterial hypertension	Endothelin receptor antagonist (5 per 100)	Placebo (13 per 100)	606 (3)	Unclear	Hospital admission (Range 2 to 4 months)	OR: 0.34 (0.17 to 0.69) NNT: 12 (9 to 27) I²: 0%	Low	Summary estimate
Coeytaux, 2014, 8747b	Pulmonary arterial hypertension	Phosphodiesterase inhibitors (5 per 100)	Placebo (9 per 100)	1011 (4)	Unclear	Hospital admission (Range 2 to 4 months)	OR: 0.48 (0.25 to 0.91) NNT: 22 (15 to 132) I²: 0%	Low	Summary estimate
**Diseases of the digestive system**									
**Crohn disease [regional enteritis] or Ulcerative colitis**									
Costa, 2013, 1629a	Crohn disease or ulcerative colitis	Infliximab [Bosentan or Ambrisentan] (19 per 100)	Placebo (31 per 100)	1736 (2)	38 ±2	Hospital admission (17 ±13 months)	OR: 0.51 (0.4 to 0.65) NNT: 8 (6 to 12) I²: 0%	Moderate	Summary estimate
**Hepatic failure**									
Saab, 2015, 8884a	Hepatic encephalopathy	Probiotics [Lactobacilli, Bifidobacteria, or Streptococcus thermophiles] (18 per 100)	No treatment, placebo, or lactulose (30 per 100)	276 (4)	Range 16 or older	Hospital admission (7 ±4 months)	OR: 0.53 (0.33 to 0.86) NNT: 9 (6 to 33) I²: 0%	Moderate	Summary estimate
**Ulcerative colitis**									
Lopez, 2015, 8829a	Ulcerative colitis	Tumour necrosis factor antagonists (anti-TNF) (12 per 100)	Placebo (17 per 100)	1691 (2)	Unclear	Admissions for ulcerative colitis (Unclear)	RR: 0.71 (0.56 to 0.9) NNT: 20 (13 to 58) I²: 0%	Moderate	Summary estimate
**Calculus of kidney and ureter**									
Campschroe r, 2014, 1164	Ureteral stones	Alpha-blocker [Tamsulosin or Doxazosin] (13 per 100)	Standard therapy (38 per 100)	313 (4)	36 ±6	Hospital admission (23 ±7 days)	RR: 0.35 (0.13 to 0.97) NNT: 4 (3 to 88) I²: 64%	Very low	Summary estimate
**Disorders resulting from impaired renal tubular function**									
Garside, 2007, 2640b	Hyperparathyroidism secondary to end-stage renal disease in patients on dialysis	Cinacalcet (12 per 100)	Placebo (20 per 100)	1184 (4)	54	Admission for cardiovascular issues (8 ±3 months)	RR: 0.61 (0.43 to 0.86) NNT: 13 (9 to 36) I²: Unclear	Low	Summary estimate
**Systemic lupus erythematosus**									
Moore, 2006, 5371	Systemic lupus erythematosus	Mycophenolate mofetil (2 per 100)	Cyclophosphamide and steroid (19 per 100)	220 (2)	35 ±4	Admission for drug-related adverse events (Range 3 to 84 months)	RR: 0.10 (0.04 to 0.50) NNT: 6 (6 to 11) I²: Unclear	Very low	Summary estimate
**Multiple sclerosis**									
La Mantia, 2010, 4260	Multiple sclerosis	Glatiramer acetate (12 per 100)	Placebo (21 per 100)	489 (2)	34 ±0.3	Hospital admission (23 ±19 months)	RR: 0.60 (0.40 to 0.91) NNT: 12 (8 to 54) I²: 0%	Moderate	Summary estimate
**Diseases of the respiratory system**									
**Asthma / Status Asmaticus**									
Rodrigo, 2005, 6568a	Acute asthma	Beta-2-agonists and anticholinergics [Glycopyrrolate, Ipratropium bromide or Oxitropium bromide] and B_2-	Beta-2-agonists [Salbutamol] (27 per 100)	1556 (9)	Range 18 to 55	Hospital admission (Length of emergency department stay)	RR: 0.68 (0.53 to 0.86) NNT: 11 (8 to 26) I²: 14%	Moderate	Summary estimate

		agonists [Salbutamol]<br>(19 per 100)							
Edmonds, 2012, 2159	Acute asthma	Early use of inhaled corticosteroids in the emergency department [Beclomethasone, Flunisolide, or Fluticasone]<br>(11 per 100)	Placebo<br>(26 per 100)	377<br>(5)	38 ±8	Hospital admission<br>(Unclear)	OR: 0.35 (0.20 to 0.60)<br>NNT: 7 (5 to 12)<br>I²: 0%	Moderate	Summary estimate
Rowe, 2000, 6670	Acute asthma	Intravenous magnesium sulfate<br>(45 per 100)	Placebo<br>(88 per 100)	422<br>(3)	Range 18 to 70	Hospital admission<br>(Length of emergency department stay)	OR: 0.11 (0.03 to 0.34)<br>NNT: 2 (1 to 6)<br>I²: Unclear	Low	Summary estimate
Kew, 2014, 4002h	Acute asthma	Intravenous magnesium sulfate<br>(27 per 100)	Placebo<br>(34 per 100)	1437<br>(8)	Unclear	Hospital admission<br>(3 ±1 hours)	OR: 0.72 (0.57 to 0.91)<br>NNT: 14 (9 to 48)<br>I²: 35%	High	Sub-group by study quality: Removal of studies at high risk of bias for blinding
Jaeschke, 2008, 3639f	Chronic asthma using inhaled corticosteroids	Long acting beta agonists [Formoterol] with inhaled corticosteroids<br>(0.3 per 100)	Inhaled corticosteroids [same dose as intervention]<br>(0.7 per 100)	5371<br>(8)	41 ±4	Admission for asthma<br>(Unclear)	OR: 0.49 (0.25 to 0.95)<br>NNT: 289 (196 to 2960)<br>I²: 0%	Low	Sub-group by intervention: Formoterol only and ICS dose same in both groups
Jaeschke, 2008, 3640d	Chronic asthma using inhaled corticosteroids	Long acting beta agonists [Formoterol] with inhaled corticosteroids<br>(0.47 per 100)	Inhaled corticosteroids [same dose as intervention]<br>(1 per 100)	5334<br>(10)	Unclear	Admission for asthma<br>(Unclear)	OR: 0.46 (0.23 to 0.93)<br>NNT: 183 (128 to 1416)<br>I²: Unclear	Low	Sub-group by intervention: Same dose of ICS in intervention and control groups
Jaeschke, 2008, 3640b	Chronic asthma using inhaled corticosteroids	Long acting beta agonists [Formoterol] with inhaled corticosteroids<br>(0.6 per 100)	Inhaled corticosteroids alone [some same dose, some higher dose]<br>(1 per 100)	1063<br>(16)	Unclear	Admission for asthma<br>(Unclear)	OR: 0.59 (0.37 to 0.93)<br>NNT: 228 (148 to 1338)<br>I²: Unclear	Low	Summary estimate
Shan, 2013, 7029b	Acute asthma	Nebulized magnesium sulfate<br>(12 per 100)	Control<br>(18 per 100)	383<br>(6)	Range 18 to 65	Admission for asthma<br>(Length of emergency department stay)	RR: 0.63 (0.43 to 0.92)<br>NNT: 15 (10 to 68)<br>I²: 0%	Moderate	Summary estimate
Rowe, 2007, 6677	Acute asthma	Oral or Intramuscular corticosteroid (CS)<br>[Methylprednisolone or Dexamethasone]<br>(4 per 100)	Placebo<br>(11 per 100)	184<br>(3)	Range 15 to 45	Hospital admission<br>(9±3 days)	RR: 0.35 (0.12 to 0.95)<br>NNT: 14 (11 to 185)<br>I²: 0%	Low	Summary estimate

Rodrigo, 1999, 6564a	Acute asthma	Parenteral or inhaled corticosteroids [Methylprednisolone] (12 per 100)	Placebo in the emergency department (18 per 100)	480 (6)		Admission for asthma (Range of 2 to 6 hours in the emergency department)	RR: 0.68 (0.47 to 0.99) NNT: 17 (10 to 556) I²:	Low	Summary estimate
**Other chronic obstructive pulmonary disease**									
Ni, 2014, 8850a	Moderate to severe chronic obstructive pulmonary disease	Aclidinium bromide (2 per 100)	Placebo (3 per 100)	5624 (10)	64 ±3	Admission for chronic obstructive pulmonary disease (14 ±8 months)	OR: 0.64 (0.46 to 0.88) NNT: 106 (70 to 321) I²: 0%	High	Summary estimate
Zou, 2016, 8933a	Moderate to severe chronic obstructive pulmonary disease	Aclidinium Bromide (2 per 100)	Placebo (3 per 100)	4788 (5)	63 ±1	Admission for chronic obstructive pulmonary disease (7 ±4 months)	OR: 0.64 (0.47 to 0.89) NNT: 103 (7 to 339) I²: 17%	Moderate	Summary estimate
Nannini, 2013, 5533g	Moderate to severe chronic obstructive pulmonary disease	Combined inhalers with Mometasone Furoate and Formoterol (2 per 100)	Placbeo (4 per 100)	894 (2)	61 ±3	Admission for chronic obstructive pulmonary disease (12 months)	OR: 0.36 (0.15 to 0.86) NNT: 38 (28 to 177) I²: 0%	Low	Sub-group by intervention: Mometasone/formot erol 200/10 mcg
Kew, 2013, 4003e	Moderate to severe chronic obstructive pulmonary disease	Formoterol (3 per 100)	Placebo (4 per 100)	2614 (6)	63 ±2	Admission for chronic obstructive pulmonary disease (14 ±12 months)	OR: 0.73 (0.54 to 0.99) NNT: 91 (53 to 2488) I²: 0%	Low	Summary estimate
Kew, 2013, 4003a	Moderate to severe chronic obstructive pulmonary disease	Long-acting beta2-agonists [Formoterol or Salmeterol] (4 per 100)	Placebo (5 per 100)	3804 (7)	62 ±2	Admission for chronic obstructive pulmonary disease (9 ±3 months)	OR: 0.73 (0.56 to 0.95) NNT: 77 (47 to 420) I²: 10%	Moderate	Summary estimate
Cheyne, 2015, 8744d	Moderate to severe chronic obstructive pulmonary disease	Tiotropium (1 per 100)	Ipratropium bromide (2 per 100)	1073 (2)	64 ±0.2	Admission for chronic obstructive pulmonary disease (6 ±5 months)	OR: 0.56 (0.31 to 0.99) NNT: 102 (65 to 4551) I²: 0%	Moderate	Summary estimate
Yohannes, 2011, 8511a	Moderate to severe chronic obstructive pulmonary disease	Tiotropium (7 per 100)	Placebo (8 per 100)	11844 (9)	65 ±2	Admission for chronic obstructive pulmonary disease (15 ±15 months)	OR: 0.89 (0.80 to 0.98) NNT: 120 (65 to 663) I²: 18%	Low	Summary estimate
Chong, 2012, 1438a	Moderate to severe chronic obstructive pulmonary disease	Tiotropium (3.8 per 100)	Long acting beta-2-agonist (4.8 per 100)	9267 (4)	63 ±1	Admission for chronic obstructive pulmonary disease (7 ±4 months)	OR: 0.87 (0.77 to 0.99) NNT: 183 (103 to 2390) I²: 0%	Moderate	Summary estimate
Karner, 2014, 8692e	Moderate to severe chronic obstructive pulmonary disease	Tiotropium with soft mist inhaler (6 per 100)	Placebo (7 per 100)	6522 (3)	65 ±1	Admission for chronic obstructive pulmonary disease (9 ±5 months)	OR: 0.82 (0.68 to 0.99) NNT: 87 (49 to 1590) I²: 0%	Moderate	Sub-group by intervention: Soft mist inhaler
**Other chronic obstructive pulmonary disease; Simple and mucopurulent chronic bronchitis**									
Poole, 2015, 8867	Chronic obstructive pulmonary disease or Chronic bronchitis	Mucolytic [N-acetylcysteine, Erdosteine, or Carbocysteine] (19 per 100)	Placebo (25 per 100)	1788 (4)	66 ±12	Hospital admission (17 ±13 months)	OR: 0.68 (0.52 to 0.89) NNT: 15 (10 to 47) I²: 58%	Moderate	Summary estimate
**Endocrine, nutritional and metabolic diseases**									
**Malnourishment**									

Stratton, 2013, 7484a	Malnourished patients being discharged from the hospital to the community	Oral nutritional supplements [Nova source liquid, Clinutren Soup and Clinutren, Fortisip, Fresubin, or Fortifresh] (21 per 100)	Usual care or dietary counselling (31 per 100)	852 (6)	Range 51 to 92	Readmission (13 ±4 months)	OR: 0.59 (0.43 to 0.8) NNT: 10 (7 to 22) I²: Unclear	Moderate	Summary estimate
Cawood, 2012, 1264	Malnourished patients or patients at-risk of disease-related malnutrition in hospital or community settings	High protein oral nutritional supplements (31 per 100)	Placebo or usual care (44 per 100)	546 (2)	65 ±17	Readmission (5 ±2 months)	OR: 0.59 (0.41 to 0.84) NNT: 8 (5 to 24) I²: 0%	Low	Summary estimate
**Factors influencing health status and contact with health services**									
**Care involving dialysis**									
James, 2008, 3657	Patients on long term hemodialysis with a central venous catheter	Topical prophylactic antibiotics [Mupirocin or Polysporin triple-antibiotic ointment] (1 per 100)	No antibiotic therapy (22 per 100)	305 (2)	57 ±13	Admission for infection (3 ±3 months)	rR: 0.06 (0.03 to 0.12) NNT: 6 (5 to 8) I²: Unclear	Moderate	Summary estimate
**Hospitalised patients**									
**Other medical care**									
Renner, 2012, 6442a	Breast cancer patients receiving chemotherapy	Primary prophylactic colony-stimulating factors [Granulocyte colony-stimulating factors or Granulocyte-macrophage colony-stimulating factors] (1 per 100)	Placebo or no treatment (10 per 100)	1149 (4)	Range 21 to 88	Admission for febrile neutropenia with antibiotics (19 ±4 months)	RR: 0.13 (0.06 to 0.3) NNT: 12 (11 to 15) I²: 0%	Low	Summary estimate
**Patients receiving surgery**									
Kumar, 2014, 8815a	Patients receiving day surgery	Total intravenous anaesthesia using propofol (0.3 per 100)	Inhalational agents sevoflurane or desflurane (0.8 per 100)	556 (5)	37 ±4	Hospital admission (Unclear)	OR: 0.35 (0.14 to 0.91) NNT: 214 (162 to 1555) I²: 0%	Low	Summary estimate
**Special screening examination for neoplasms**									
Yang, 2015, 8918a	Patients undergoing prostate biopsy	Antibiotics [Ofloxacin, Trimethoprim, Sulfonamide methoxazole, or Ciprofloxacin] (2 per 100)	Placebo or no treatment (13 per 100)	650 (3)	Unclear	Hospital admission (Unclear)	RR: 0.13 (0.03 to 0.55) NNT: 9 (8 to 17) I²: 0%	Moderate	Summary estimate
**Supervision of high-risk pregnancy; Supervision of normal pregnancy**									
Makrides, 2014, 4844	Normal or high-risk pregnancy	Magnesium supplementation (8 per 100)	Placebo (12 per 100)	1158 (3)	Unclear	Hospital admission (Unclear)	RR: 0.65 (0.48 to 0.86) NNT: 24 (16 to 60) I²: 0%	Moderate	Summary estimate
**Human immunodeficiency virus**									
Grimwade, 2009, 2933a	Human Immunodeficieny Virus	Cotrimoxazole (11 per 100)	Placebo (17 per 100)	764 (3)	34 ±2	Hospital admission (Unclear)	RR: 0.66 (0.48 to 0.92) NNT: 17 (11 to 74) I²: 91%	Moderate	Summary estimate
**Mental and behavioural disorders**									
**Schizophrenia and other types of schizophrenia-like psychoses (Acute and transient psychotic disorders; Bipolar affective disorder; Induced delusional disorder; Other nonorganic psychotic disorders; Persistent delusional disorders; Schizoaffective disorders; Schizophrenia; Schizotypal disorder; Unspecified nonorganic psychosis)**									

Kishimoto, 2014, 4067b	Schizophrenia and other types of schizophrenia-like psychoses	Fluphenazine depot [Fulphenazine Decanoate or Fulphenazine Enanthate] (23 per 100)	Oral antipsychotic drugs [Pimozide, Trifluoperazine, or Fulphenazine] (28 per 100)	197 (4)	44 ±7	Hospital admission (12 ±3 months)	RR: 0.82 (0.67 to 0.99) NNT: 20 (11 to 358) I²: 0%	Low	Sub-group by intervention: Fluphenazine depot
Leucht, 2012, 4508a	Schizophrenia and other types of schizophrenia-like psychoses	Maintenance/continuous treatment with antipsychotic drugs [Fluphenazine decanoate, Prochlorpromazine, Perphenazine, Chlorpromazine, Promazine Trifluoperazine, Haloperidol, Olanzapine, Quetiapine, Paliperidone palmitate, Pimozide, Risperidone, Zuclopenthixol] (12 per 100)	Placebo or no treatment (33 per 100)	2090 (16)	38 ±9	Readmission (11 ±7 months)	RR: 0.38 (0.27 to 0.55) NNT: 5 (4 to 7) I²: 45%	High	Summary estimate
Komossa, 2013, 4123i	Schizophrenia and other types of schizophrenia-like psychoses	Olanzapine (11 per 100)	Ziprasidon (17 per 100)	766 (2)	41 ±0.1	Readmission (13 ±9 months)	RD: -0.06 (-0.01 to -0.11) NNT: 17 (9 to 100) I²: 0%	Moderate	Summary estimate
Komossa, 2013, 4123b	Schizophrenia and other types of schizophrenia-like psychoses	Olanzapine (11 per 100)	Quetiapin (20 per 100)	876 (2)	40 ±0.4	Readmission (13 ±9 months)	RR: 0.56 (0.41 to 0.77) NNT: 11 (8 to 22) I²: 0%	Moderate	Summary estimate
Kishimoto, 2013, 4065a	Schizophrenia	Second generation antipsychotics [Amisulpride, Clozapine, Iloperidone, Olanzapine, Quetiapine, Risperidone, Sertindole, or Ziprasidone] (15 per 100)	First generation antipsychotics [Haloperidol] (21 per 100)	2869 (12)	33 ±6	Hospital admission (13 ±4 months)	RR: 0.72 (0.58 to 0.9) NNT: 17 (11 to 48) I²: 18%	Moderate	Summary estimate
**Mixed population**									
Hemkens, 2016, 8784a	Preventing cardiovascular events in patients with alcoholic cirrhosis or idiopathic pulmonary fibrosis	Colchicine (41 per 100)	Control (47 per 100)	599 (2)	61 ±8	Hospital admission (49 ±33 months)	RR: 0.87 (0.77 to 0.99) NNT: 16 (9 to 212) I²: 0%	Low	Summary estimate
Makani, 2013, 4838a	Heart failure; heart failure and haemodialysis; diabetic nephropathy; high risk cardiovascular disease	Dual renin-angiotensin blockade [Angiotensin II receptor blocker (Candesartan, Telmisartan, Valsartan, or other), angiotensin converting enzyme inhibitor (any), direct renin inhibitor (Aliskiren)] (23 per 100)	Single renin-angiotensin blockade [Angiotensin II receptor blocker (Telmisartan), angiotensin converting enzyme inhibitor inhibitor (Any), or direct renin inhibitor (Aliskiren)] (28 per 100)	42071 (5)	64 ±2	Admission for heart failure (38 ±12 months)	RR: 0.82 (0.74 to 0.92) NNT: 20 (14 to 45) I²: 68%	Low	Summary estimate
**Multi-morbidity**									
Baumeister, 2011 593	Depression in patients with recent acute coronary syndromes	Atypical antidepressant or selective serotonin reuptake inhibitor [Mirtazapine; Sertraline; Fluoxetine] (15 per 100)	Placebo (23 per 100)	514 (3)	57 ±0.1	Hospital admission (6 ±0.5 months)	OR: 0.58 (0.39 to 0.85) NNT: 12 (8 to 37) I²: 0%	Very low	Summary estimate
**Pregnancy, childbirth and the puerperium"**									
Magee, 2012, 4812a	Mild to moderate, late-onset pre-eclampsia	Beta-blocker [Oxprenolol, Labetalol, or Metoprolol] (56 per 100)	Placebo or no treatment (73 per 100)	291 (3)	Unclear	Hospital admission (Range from first week to babies birth)	RR: 0.77 (0.64 to 0.93) NNT: 6 (4 to 20) I²: 85%	Low	Summary estimate

[a]Median hospitalisation event rate observed across the trials contributing to the pooled estimate. [b]Means and standard deviations for patient ages in years, unless otherwise specified. Mean of means and median ages in trials contributing to the pooled estimate. Not all included trials reported age in a form that could be averaged. [c]Means and standard deviations for length of follow-up across trials, unless otherwise specified. Mean of means, medians, and total study durations reported in trials contributing to the pooled estimate. Not all included trials reported follow-up in a form that could be averaged. [d]RR= risk ratio; OR=odds ratio; rR= rate ratio; HR= hazard ratio; RD= risk difference. [e]CI=confidence interval. [f]NNT= Number needed to treat to benefit by avoiding one hospital admission.

Table S8. Trial information underpinning GRADED estimates

Review author, year, estimate ID	Trials	Intervention: specific drugs and dosing	Comparator: specific drugs and dosing	Country	Length of follow-up (months)	Follow-up measure	Age	Age measure	Number of intervention group events	Number of control group events	Number of intervention patients	Number of control patients
Afilalo, 2007 100c	Goldbourt U, 1993 (PROVE-IT TIMI 22)	Atorvastatin 80 mg/day	Pravastatin 40 mg/day	Unclear	24	Mean	58	Mean	34	64	2099	2063
	Gottlieb SS, 1998 (A to Z)	Simvastatin 80 mg/day	Simvastatin 20 mg/day	Unclear	24	Mean	61	Mean	72	98	2265	2232
	Dargie HJ, 2001 (IDEAL)	Atorvastatin 80 mg/day	Simvastatin 20 mg/day	Unclear	57.6	Mean	62	Mean	99	123	4439	4449
	Heart Protection Study Collaborative Group, 2002 (TNT)	Atorvastatin 80 mg/day	Atorvastatin 10 mg/day	Unclear	58.8	Mean	61	Mean	122	164	4995	5006
Review author, year, estimate ID	Trials	Intervention: specific drugs and dosing	Comparator: specific drugs and dosing	Country	Length of follow-up (months)	Follow-up measure	Age	Age measure	Number of intervention group events	Number of control group events	Number of intervention patients	Number of control patients
Brophy, 2001 1021	Engelmeier et al (20)	Metoprolol	Placebo	Unclear	12	Mean	50	Mean	1	4	9	16
	Woodley et al. (36)	Bucindolol	Placebo	Unclear	3	Mean	52	Mean	1	2	29	20
	Waagstein et al. (21)	Metoprolol	Placebo	Unclear	18	Mean	49	Mean	37	49	194	189
	Fisher et al. (23)	Metoprolol	Placebo	Unclear	6	Mean	63	Mean	1	8	25	25
	Bristow et al. (24)	Bucindolol	Placebo	Unclear	3	Mean	55	Mean	7	3	105	34
	CIBISI. (25)	Bisoprolol	Placebo	Unclear	23	Mean	60	Mean	54	82	320	321
	Eichhorn et al. (26)	Metoprolol	Placebo	Unclear	3	Mean	48	Mean	0	2	15	9
	Metra et al. (27)	Carvedilol	Placebo	Unclear	4	Mean	51	Mean	0	2	20	20
	Olsen et al. (38)	Carvedilol	Placebo	Unclear	4	Mean	52	Mean	2	0	36	23
	Krum et al. (39)	Carvedilol	Placebo	Unclear	4	Mean	55	Mean	1	2	33	16
	Bristow et al. (29)	Carvedilol	Placebo	Unclear	6	Mean	63	Mean	18	8	261	84
	Packer et al. (30)	Carvedilol	Placebo	Unclear	6	Mean	60	Mean	9	18	133	145

	Colucci et al. (31)	Carvedilol	Placebo	Unclear	15	Mean	54	Mean	9	9	232	134
	Cohn et al. (32)	Carvedilol	Placebo	Unclear	8	Mean	58	Mean	3	1	70	35
	Aust/NZ (28)	Carvedilol	Placebo	Unclear	19	Mean	67	Mean	23	33	208	208
	CIBIS-II (17)	Bisoprolol	Placebo	Unclear	15	Mean	61	Mean	159	232	1327	1320
	MERIT-HF (18)	Metoprolol	Placebo	Unclear	12	Mean	64	Mean	200	294	1990	2001
	RESOLVD (34)	Metoprolol	Placebo	Unclear	6	Mean	62	Mean	15	5	214	212
Review author, year, estimate ID	Trials	Intervention: specific drugs and dosing	Comparator: specific drugs and dosing	Country	Length of follow-up (months)	Follow-up measure	Age	Age measure	Number of intervention group events	Number of control group events	Number of intervention patients	Number of control patients
Cordina, 2005 1606a	AFFIRM	amiodarone, disopyramide, flecainide, moricizine, procainamide, propafenone, quinidine, sotalol, dofetilide or combinations chosen by the treating physician	beta-blockers, calcium-channel blockers, digoxin or combination of these drugs	Unclear	42	Mean	69.7	Mean	1374	1220	2033	2027
	PIAF	amiodarone (600mg for 3 weeks, 200mg maintenance)	diltiazem 90 mg twice or three times daily	Unclear	12	Follow-up duration	61	Mean	87	30	127	125
Review author, year, estimate ID	Trials	Intervention: specific drugs and dosing	Comparator: specific drugs and dosing	Country	Length of follow-up (months)	Follow-up measure	Age	Age measure	Number of intervention group events	Number of control group events	Number of intervention patients	Number of control patients
Costa, 2013 1629a	Hannauer, 2002; Rutgeerts, 2004 (ACCENT 1)	Infliximab: 5 mg/kg to 10mg/kg (15 mg/kg possible in one crossover group)	Placebo	North America, Europe, Israel	13.5	Follow-up duration	35	Median	44	71	192	188
	Sands, 2004; Lichtenstein 2005 (ACCENT 2)	Infliximab: 5 mg/kg (10 mg/kg possible in one crossover group)	Placebo	North America, Europe, Israel	13.5	Follow-up duration	39	Median	15	44	139	143
	Targan, 1997	Infliximab: 5 mg/kg to 20 mg/kg	Placebo	North America, Europe	3	Follow-up duration	38	Median	2	0	83	25
	Rutgeerts, 2005; Sandborn, 2009 (ACT 1/2)	Infliximab: 5 mg/kg to 10 mg/kg	Placebo	USA and Europe	13.5	Follow-up duration	41	Median	97	49	484	244
	Jarnerot, 2005; Gustavsson, 2010 (Swedish-Danish controlled infliximab study)	Infliximab: 5 mg/kg	Placebo	Swedan, Denmark	39	Follow-up duration	37	Mean	2	6	24	21

Review author, year, estimate ID	Trials	Intervention: specific drugs and dosing	Comparator: specific drugs and dosing	Country	Length of follow-up (months)	Follow-up measure	Age	Age measure	Number of intervention group events	Number of control group events	Number of intervention patients	Number of control patients
Danchin, 2006 1752a	Yusuf, 2000 (HOPE)	Ramipril, 10 mg (administered in evening) for 7-10 d at 2.5 mg, followed by placebo for 10-14 d	Placebo	Unclear	60	Mean	66	Mean	141	160	4645	4652
	MacMahon, 2000 (PART 2)	Ramipril, 5-10 mg	Placebo	Unclear	56.4	Mean	61	Mean	7	9	308	309
	EUROPA4	Perindopril, 8 mg, for 4 wk	Placebo	Unclear	50.4	Mean	60	Mean	63	103	6110	6108
	Fox, 2003 (PEACE)	Trandolapril, 2 mg, for 6 mo, then either 2 mg or 4 mg at discretion of investigators if SBP 110 mm Hg, for 2 wk	Placebo	Unclear	57.6	Median	64	Mean	105	134	4158	4132
	Nissen, 2004 (CAMELOT)	Enalapril maleate, 10 mg twice a day (third arm, amlodipine maleate, 5 mg) twice a day	Placebo	Unclear	24	Mean	58	Mean	4	5	673	655
Review author, year, estimate ID	Trials	Intervention: specific drugs and dosing	Comparator: specific drugs and dosing	Country	Length of follow-up (months)	Follow-up measure	Age	Age measure	Number of intervention group events	Number of control group events	Number of intervention patients	Number of control patients

Gandhi, 2014 2599a	Licata, 2002	If sodium b125 mEq/L, given IV 150 cc of 4.6% NaCl, if sodium 126–135 mEq/L, given 150 cc of IV 3.5% NaCl, if sodium N135 mEq/L, given 150 cc of IV 1.4%–2.4% NaCl. All patients supplemented with potassium chloride 20–40 mEq/L as necessary. Given twice daily. IV furosemide infusion 500–1000 mg daily	IV furosemide infusion 500–1000 mg daily	Unclear	31	Mean	74.599	Mean	25	43	53	54
	Paterna, 2000	If sodium b125 mEq/L, given IV 150 cc of 4.6% NaCl, if sodium 126–135 mEq/L, given 150 cc of IV 3.5% NaCl, if sodium N135 mEq/L, given 150 cc of IV 1.4%–2.4% NaCl. All patients supplemented with potassium chloride 20–40 mEq/L as necessary. Given twice daily. IV furosemide infusion 500–1000 mg daily	IV furosemide infusion 500–1000 mg daily	Unclear	6 to 12	Range	73.935	Mean	0	12	30	30
	Paterna, 2005	If sodium b125 mEq/L, given 150 cc of IV 4.6% NaCl, if sodium 126–135 mEq/L, given 150 cc of IV 3.5% NaCl, if sodium N135 mEq/L, given 150 cc of IV 1.4%–2.4% NaCl. All patients supplemented with potassium chloride 20–40 mEq/L as necessary. Given twice daily. IV furosemide bolus 500–1000 mg twice daily	IV furosemide bolus 500–1000 mg twice daily	Unclear	1	Follow-up duration	74.602	Mean	0	12	48	46

	Paterna, 2011	If sodium b125 mEq/L, given 150 cc IV 4.6% NaCl, if sodium 126–135 mEq/L, given 150 cc IV 3.5% NaCl, if sodium N135 mEq/L, given 150 cc IV 1.4%–2.4% NaCl. All patients supplemented with potassium chloride 20–40 mEq/L as necessary. Given twice daily.IV furosemide bolus 250 mg twice daily	IV furosemide bolus 250 mg twice daily	Unclear	57	Mean	74.043	Mean	163	305	881	890
Review author, year, estimate ID	Trials	Intervention: specific drugs and dosing	Comparator: specific drugs and dosing	Country	Length of follow-up (months)	Follow-up measure	Age	Age measure	Number of intervention group events	Number of control group events	Number of intervention patients	Number of control patients
Garside, 2007 2640b	Block, 2004 (Amgen 172)	30 mg cinacalcet orally, once daily. Dose increased, in 20-mg increments from 30 to 180 mg per day at 3- or 4-weekly intervals.	Placebo	North America, Europe and Australia	13	Follow-up duration	Trial level data not reported	Unclear	205	205	Trial specific numbers not reported	Trial specific numbers not reported
	Block, 2004 (Amgen 183)	30 mg cinacalcet orally, once daily. Dose increased, in 30-mg increments from 30 to 180 mg per day at 3- or 4-weekly intervals.	Placebo	North America, Europe and Australia	6.5	Follow-up duration	Trial level data not reported	Unclear	166	165	Trial specific numbers not reported	Trial specific numbers not reported
	Lien, 2005 (Amgen 188)	30 mg cinacalcet orally, once daily. Dose increased, in 30-mg increments from 30 to 180 mg per day at 3- or 4-weekly intervals.	Placebo	USA	6.5	Follow-up duration	Trial level data not reported	Unclear	294	101	Trial specific numbers not reported	Trial specific numbers not reported
	Lindberg, 200372 (Amgen 141)	30 mg cinacalcet orally, once daily. Dose increased, in 30-mg increments from 30 to 180 mg per day at 3- or 4-weekly intervals.	Placebo	USA and Canada	6.5	Follow-up duration	Trial level data not reported	Unclear	32	16	Trial specific numbers not reported	Trial specific numbers not reported
Review author, year, estimate ID	Trials	Intervention: specific drugs and dosing	Comparator: specific drugs and dosing	Country	Length of follow-up (months)	Follow-up measure	Age	Age measure	Number of intervention group events	Number of control group events	Number of intervention patients	Number of control patients

Jaeschke, 2008 3640b	1997 Pauwels (37-3018)	Formoterol with inhaled corticosteroid	Inhaled corticosteroid of similar dose	Unclear	Unclear	Unclear	Unclear	Unclear	3	8	426	427
	1997 van der Molen (37-3008)	Formoterol with inhaled corticosteroid	Inhaled corticosteroid of similar dose	Unclear	Unclear	Unclear	Unclear	Unclear	0	3	125	114
	2001 O'Byrne (SD-037-0345)	Formoterol with inhaled corticosteroid	Inhaled corticosteroid of similar dose	Unclear	Unclear	Unclear	Unclear	Unclear	7	10	869	862
	2002 Chuchalin (MA-037-0001)	Formoterol with inhaled corticosteroid	Inhaled corticosteroid of similar dose	Unclear	Unclear	Unclear	Unclear	Unclear	0	1	111	114
	2002 Price, Part 2 (MA-037-0001)	Formoterol with inhaled corticosteroid	Inhaled corticosteroid of similar dose	Unclear	Unclear	Unclear	Unclear	Unclear	0	1	250	255
	2005 Morice (SD-039-0681)	Formoterol with inhaled corticosteroid	Inhaled corticosteroid of similar dose	Unclear	Unclear	Unclear	Unclear	Unclear	0	1	462	217
	2000 Kips (37-3041)	Formoterol with inhaled corticosteroid	Inhaled corticosteroid of higher dose	Unclear	Unclear	Unclear	Unclear	Unclear	1	0	29	31
	2003 Bateman (SD-039-0618)	Formoterol with inhaled corticosteroid	Inhaled corticosteroid of higher dose	Unclear	Unclear	Unclear	Unclear	Unclear	0	2	168	176
	2003 Lalloo (SD-039-0348)	Formoterol with inhaled corticosteroid	Inhaled corticosteroid of higher dose	Unclear	Unclear	Unclear	Unclear	Unclear	1	0	230	237
	2004 Scicchitano (SD-039-0668)	Formoterol with inhaled corticosteroid	Inhaled corticosteroid of higher dose	Unclear	Unclear	Unclear	Unclear	Unclear	5	10	947	943
	2005 O'Byrne (excl. children <11 years) (SD-039-0673)	Formoterol with inhaled corticosteroid	Inhaled corticosteroid of higher dose	Unclear	Unclear	Unclear	Unclear	Unclear	15	10	1592	818

Review author, year, estimate ID	Trials	Intervention: specific drugs and dosing	Comparator: specific drugs and dosing	Country	Length of follow-up (months)	Follow-up measure	Age	Age measure	Number of intervention group events	Number of control group events	Number of intervention patients	Number of control patients
Jaeschke, 2008 3640d	1997 Pauwels (37-3018)	Formoterol with inhaled corticosteroid	Inhaled corticosteroid of similar dose	Unclear	Unclear	Unclear	Unclear	Unclear	3	8	426	427
	1997 van der Molen (37-3008)	Formoterol with inhaled corticosteroid	Inhaled corticosteroid of similar dose	Unclear	Unclear	Unclear	Unclear	Unclear	0	3	125	114
	2001 O'Byrne (SD-037-0345)	Formoterol with inhaled corticosteroid	Inhaled corticosteroid of similar dose	Unclear	Unclear	Unclear	Unclear	Unclear	7	10	869	862
	2002 Chuchalin (MA-037-0001)	Formoterol with inhaled corticosteroid	Inhaled corticosteroid of similar dose	Unclear	Unclear	Unclear	Unclear	Unclear	0	1	111	114
	2002 Price, Part 2 (MA-037-0001)	Formoterol with inhaled corticosteroid	Inhaled corticosteroid of similar dose	Unclear	Unclear	Unclear	Unclear	Unclear	0	1	250	255
	2005 Morice (SD-039-0681)	Formoterol with inhaled corticosteroid	Inhaled corticosteroid of similar dose	Unclear	Unclear	Unclear	Unclear	Unclear	0	1	462	217

Review author, year, estimate ID	Trials	Intervention: specific drugs and dosing	Comparator: specific drugs and dosing	Country	Length of follow-up (months)	Follow-up measure	Age	Age measure	Number of intervention group events	Number of control group events	Number of intervention patients	Number of control patients
James, 2008 3657	Sesso, 1998	Mupirocin ointment, 2%; heparin, 1000 U/mL	Povidone–iodine, 10%; heparin, 1000 U/mL	Brazil	1.13	Mean	47	Mean	5	14	69	67
	Lok , 2003	Polysporin triple-antibiotic ointment; heparin, 10 000 U/mL	Placebo ointment; heparin, 10 000 U/mL	Canada	4.77	Mean	66	Mean	6	19	83	79
Review author, year, estimate ID	Trials	Intervention: specific drugs and dosing	Comparator: specific drugs and dosing	Country	Length of follow-up (months)	Follow-up measure	Age	Age measure	Number of intervention group events	Number of control group events	Number of intervention patients	Number of control patients
Kew, 2014 4004o	Tashkin 2008 SHINE	Budesonide 160 mcg × 2 inhalations (320 mcg) twice daily	Placebo	USA, Czech Republic, the Netherlands, Poland and South Africa	6	Follow-up duration	63.296	Mean	3	1	275	300
	Shaker 2009	Budesonide 400 mcg twice daily	Placebo	Denmark	24-48	Follow-up duration	63.6	Mean	10	3	127	127
Review author, year, estimate ID	Trials	Intervention: specific drugs and dosing	Comparator: specific drugs and dosing	Country	Length of follow-up (months)	Follow-up measure	Age	Age measure	Number of intervention group events	Number of control group events	Number of intervention patients	Number of control patients
Kew, 2014 4004r	Calverley 2010	Budesonide/formoterol 400/12 mcg twice daily	Formoterol 12 mcg twice daily	25 countries	11	Follow-up duration	63.901	Mean	7	1	242	239
	Fukuchi 2013	Budesonide/formoterol combination 320/9 mcg twice daily	Formoterol 9 mcg twice daily	India, Japan, Republic of Korea, Phillipines, Poland, Russian Federation, Taiwan, Ukraine and Vietnam	3	Follow-up duration	65.059	Mean	5	2	636	657
	Rennard 2009	Budesonide/formoterol 320/9 mcg twice daily	Formoterol 9 mcg twice daily	USA, Europe and Mexico	12	Follow-up duration	63.1	Mean	5	4	494	247
	Shaker 2009	Budesonide 400 mcg twice daily	Placebo	Denmark	24-48	Follow-up duration	63.6	Mean	10	3	127	127

	Sharafkhaneh 2012	Budesonide/formoterol 320/9 mcg twice daily	Formoterol DPI 9 mcg twice daily	United States, Central and South America, and South Africa	12	Follow-up duration	63.369	Mean	13	4	407	202
	Tashkin 2008 SHINE	Budesonide 160 mcg × 2 inhalations (320 mcg) twice daily	PLacebo	USA, Czech Republic, the Netherlands, Poland and South Africa	6	Follow-up duration	63.296	Mean	3	1	275	300
	Tashkin 2008 SHINE	Budesonide/formoterol 160/4.5 mcg × 2 inhalations (320/9 mcg) twice daily OR Budesonide/formoterol 80/4.5 mcg × 2 inhalations (160/9 mcg) twice daily OR Budesonide 160 mcg × 2 inhalations (320 mcg) twice daily plus formoterol DPI 4.5 mcg × 2 inhalations (9 mcg) twice daily	Formoterol DPI 4.5 mcg × 2 inhalations (9 mcg) twice daily	USA, Czech Republic, the Netherlands, Poland and South Africa	6	Follow-up duration	63.5	Mean	3	1	564	142
Review author, year, estimate ID	Trials	Intervention: specific drugs and dosing	Comparator: specific drugs and dosing	Country	Length of follow-up (months)	Follow-up measure	Age	Age measure	Number of intervention group events	Number of control group events	Number of intervention patients	Number of control patients
Kew, 2014 4004z	Hanania 2003	Fluticasone propionate 250 mcg twice daily	Placebo	USA	6	Follow-up duration	63.499	Mean	0	1	178	177

	GSK SCO100470 2006	Salmeterol/fluticasone propionate 50/250 mcg twice daily	Salmeterol 50 mcg twice daily	Australia (10), Bulgaria (5),Croatia (1),Czech Republic (8), France (14), Germany (18), Greece (4), Italy (16), Latvia (5), Lithuania (2), Netherlands (12), Philippines (3), Poland (5), Romania (3), Russian Federation (8) , Slovakia (4), Slovenia (4), Sweden (4), Thailand (4) and United Kingdom (5))	6	Follow-up duration	63.601	Mean	2	4	518	532
	Kerwin 2013	Fluticasone furoate 100 mcg daily	Placebo	Chile, Estonia, Germany, Japan, Korea, Phillipines, Poland, Russian Federation and the United States	6	Follow-up duration	62.66	Mean	4	4	618	412
	GSK SCO30002 2005	Fluticasone propionate 500 mcg twice daily	Placebo	Italy and Poland	12	Follow-up duration	65.137	Mean	1	1	131	125
	Martinez 2013	Fluticasone furoate 100 mcg or 200 mcg daily	Placebo	Czech Republic,Germany, Japan, Poland, Romania, Russian Federation, Ukraine and the United States	6	Follow-up duration	61.617	Mean	5	2	816	408
	Anzueto 2009	Fluticasone/salmeterol 250/50 mcg twice daily	Salmeterol 50 mcg twice daily	USA and Canada	12	Follow-up duration	65.349	Mean	13	8	394	403
	Calverley 2007 TORCH	Fluticasone 500 mcg twice daily	Placebo	25 countries	36	Follow-up duration	65.05	Mean	138	82	1546	1542
	Calverley 2007 TORCH	Fluticasone/salmeterol 500/50 mcg twice daily	Salmeterol 50 mcg twice daily	25 countries	36	Follow-up duration	65	Mean	121	69	1552	1545

	GSK FLTA3025 2005	Fluticasone propionate 250 mcg or 500 mcg twice daily	Placebo	USA	6	Follow-up duration	64.393	Mean	4	1	434	206
	Hanania 2003	Fluticasone/salmeterol 250/50 mcg twice daily	Salmeterol 50 mcg twice daily	USA	6	Follow-up duration	64.005	Mean	1	0	183	185
	GSK SCO104925 2008	Fluticasone propionate 500 mcg twice daily	Placebo	Russian Federation, 4 centres in the United States, 2 centres in Chile and 1 centre in Estonia	3	Follow-up duration	64.7	Mean	1	0	42	42
	Dransfield 2013	Fluticasone furoate/vilanterol 50/25 mcg daily; 100/25 mcg daily; or 200/25 mcg daily	Vilanterol 25 daily	Argentina, Australia, Canada, Chile, Estonia, Germany, Italy, Mexico, Netherlands, Peru, Phillipines, South Africa, Sweden, the United Kingdom and the United States	12	Follow-up duration	63.675	Mean	72	8	2437	818
	Kardos 2007	Salmeterol/fluticasone 50/500 mcg twice daily	Salmeterol 50 mcg twice daily	Germany	10	Follow-up duration	63.898	Mean	13	3	507	487
Review author, year, estimate ID	Trials	Intervention: specific drugs and dosing	Comparator: specific drugs and dosing	Country	Length of follow-up (months)	Follow-up measure	Age	Age measure	Number of intervention group events	Number of control group events	Number of intervention patients	Number of control patients
Kew, 2014 4004y	Tashkin 2008 SHINE	Budesonide 160 mcg × 2 inhalations (320 mcg) twice daily	Placebo twice daily	USA, Czech Republic, the Netherlands, Poland and South Africa	6	Follow-up duration	68.045	Mean	5	1	845	284
	Fukuchi 2013	Budesonide/formoterol combination 320/9 mcg twice daily	Formoterol 9 mcg twice daily	India, Japan, Republic of Korea, Phillipines, Poland, Russian Federation, Taiwan, Ukraine and Vietnam	3	Follow-up duration	65.059	Mean	5	2	636	657

	Tashkin 2008 SHINE	Budesonide/formoterol 160/4.5 mcg × 2 inhalations (320/9 mcg) twice daily OR Budesonide/formoterol 80/4.5 mcg × 2 inhalations (160/9 mcg) twice daily OR Budesonide 160 mcg × 2 inhalations (320 mcg) twice daily plus formoterol DPI 4.5 mcg × 2 inhalations (9 mcg) twice daily	Formoterol DPI 4.5 mcg × 2 inhalations (9 mcg) twice daily	USA, Czech Republic, the Netherlands, Poland and South Africa	6	Follow-up duration	63.296	Mean	3	1	275	300
	Calverley 2010	Budesonide/formoterol 400/12 mcg twice daily	Formoterol 12 mcg twice daily	25 countries	11	Follow-up duration	63.902	Mean	7	1	242	238

Review author, year, estimate ID	Trials	Intervention: specific drugs and dosing	Comparator: specific drugs and dosing	Country	Length of follow-up (months)	Follow-up measure	Age	Age measure	Number of intervention group events	Number of control group events	Number of intervention patients	Number of control patients
Kishimoto, 2013 4065a	Kahn, 2008	Amisulpride 450.8 (200-800) Mean dose (range/fixed (mg day 1))	Haloperidol 3 (1-4) Mean dose (range/fixed (mg day 1))	Europe and Israel	12	Mean	26	Mean	14	14	88	64
	Essock, 1996	Clozapine 496 Mean dose (range/fixed (mg day 1))	First generation antipsychotics 1386 mean dose (range/fixed, mg day)	USA	26	Mean	41.2	Mean	13	20	76	48
	Lieberman, 2003	Clozapine 600 flexibe median mg day	Not reported	China	10	Mean	28.7	Mean	6	5	80	80
	Kane, 2008 (study 1-3)	Iloperidone 11.8 (4-16) Mean dose (range/fixed (mg day 1))	Haloperidol 13.2 (5-20) Mean dose (range/fixed (mg day 1))	International	11.5	Mean	34.7	Mean	27	7	359	114
	Kahn, 2008	Olanzapine 12.6 (5-20) Mean dose (range/fixed (mg day 1))	Haloperidol 3 (1-4) Mean dose (range/fixed (mg day 1))	Europe and Israel	12	Mean	26	Mean	18	14	89	64
	Tran, 1998 (study 1)	Olanzapine 12.1 (12) Mean dose (range/fixed (mg day 1))	Haloperidol 14 (14) Mean dose (range/fixed (mg day 1))	International	11.5	Mean	37	Mean	10	2	45	10
	Tran, 1998 (study 2)	Olanzapine 11.5 (12) Mean dose (range/fixed (mg day 1))	Haloperidol 16.4 (16) Mean dose (range/fixed (mg day 1))	International	11.5	Mean	37	Mean	6	3	48	14
	Tran, 1998 (study 3)	Olanzapine 13.9 (5-20) Mean dose (range/fixed (mg day 1))	Haloperidol 13.2 (5-20) Mean dose (range/fixed (mg day 1))	International	5.5 - 21	Range	37	Mean	71	29	534	156

	Kahn, 2008	Quetiapine 498.6 (250-700) Mean dose (range/fixed (mg day 1))	Haloperidol 3 (1-4) Mean dose (range/fixed (mg day 1))	Europe and Israel	12	Mean	26	Mean	14	14	60	64
	Csernansky, 2002	Risperidone 4.9 (2-8) Mean dose (range/fixed (mg day 1))	Haloperidol 11.7 (5-20) Mean dose (range/fixed (mg day 1))	USA	12	Mean	40.2	Mean	20	36	177	188
	de Sena, 2003	Risperidone 4.0 (flexible) median dose ((mg day 1))	Haloperidol 10 (flexible) Median dose (mg day 1))	Brazil	12	Mean	27.7	Mean	6	3	20	13
	Gaebel, 2007	Risperidone 4.2 (2-4) Mean dose (range/fixed (mg day 1))	haloperidol 4.1 (2-4) Mean dose (range/fixed (mg day 1))	Germany	12	Mean	31.6	Mean	7	7	77	74
	Daniel, 1998	Sertindole 24 Mean dose (range/fixed (mg day 1))	Haloperidol 10 mean dose (mg day 1))	USA	12	Mean	37	Mean	2	12	94	109
	Kahn, 2008	Ziprasidone 107.2 (40-160) Mean dose (range/fixed (mg day 1))	Haloperidol 3 (1-4) Mean dose (range/fixed (mg day 1))	Europe and Israel	12	Mean	26	Mean	4	14	60	64

Review author, year, estimate ID	Trials	Intervention: specific drugs and dosing	Comparator: specific drugs and dosing	Country	Length of follow-up (months)	Follow-up measure	Age	Age measure	Number of intervention group events	Number of control group events	Number of intervention patients	Number of control patients
Kishimoto, 2014 4067b	Barnes 1983	Fulphenazine decanoate (dose not reported, same dose as before the trial)	Pimozide (dose not reported, same dose as before the trial)	UK	12	Follow-up duration	49.5	Mean	3	3	19	17
	Crawford 1974	Fulphenazine decanoate (dose not reported, same dose as before the trial)	Trifluoperazine 10 mg/d (fixed)	UK	9.3	Follow-up duration	20 to 65	Range	0	4	14	15
	Del Giudice 1975	Fulphenazine Enanthate 25 mg/2 wks (fixed)	Fulphenazine 21.7 mg (5–80 mg)	USA	16	Follow-up duration	20 to 50	Range	21	59	27	61
	Falloon 1978	Fulphenazine decanoate 25 mg/2 wks (flexible)	Pimozide 8 mg/d (flexible)	UK	12	Follow-up duration	39	Mean	7	7	20	24

Review author, year, estimate ID	Trials	Intervention: specific drugs and dosing	Comparator: specific drugs and dosing	Country	Length of follow-up (months)	Follow-up measure	Age	Age measure	Number of intervention group events	Number of control group events	Number of intervention patients	Number of control patients
Kuenzli, 2010 4206a	RESOLVD 1999	Candesartan 16mg once daily and Enalapril 10mg twice	Enalapril 10mg twice daily	Unclear	9.6	Follow-up duration	63.753	Mean	31	7	332	109
	Arutiunov 2000	ARB and an ACE inhibitor (not specified)	Not specified	Unclear	30	Follow-up duration	64	Mean	15	57	35	70

	CHARM added 2003	Candesartan 32mg once daily and ACE inhibitors	No recommended fixed dose, but investigators were advised of the doses of ACE inhibitors known to reduce morbidity and mortality in patients with congestive heart failure	Unclear	40.8	Follow-up duration	64	Mean	323	382	1276	1272
	VALIANT 2003	Valsartan 80mg twice daily and Captopril 50mg three times daily	Captopril 50mg three times daily	Unclear	24	Follow-up duration	65	Mean	834	945	4862	4879
	Kum 2008	Irbesartan 300mg and an ACE inhibitor	Not specified	Unclear	12	Follow-up duration	67.5	Mean	4	7	25	25
	ValHeFT 2001	Valsartan 160mg twice daily and an ACE inhibitor	Not specified	Unclear	24	Follow-up duration	62.499	Mean	346	455	2511	2499
Review author, year, estimate ID	Trials	Intervention: specific drugs and dosing	Comparator: specific drugs and dosing	Country	Length of follow-up (months)	Follow-up measure	Age	Age measure	Number of intervention group events	Number of control group events	Number of intervention patients	Number of control patients
Lipinski, 2009 4615d	Hamaad 2005	atorvastatin (40 mg/day)	Placebo	Unclear	3	Mean	67	Mean	0	1	13	10
	Sola 2006	atorvastatin (20 mg/day)	Placebo	Unclear	12	Mean	53	Mean	8	13	54	54
	Vrtovec 2008	atorvastatin (10 mg/day)	Placebo	Unclear	12	Mean	63	Mean	8	20	55	55
	Wojnicz 2006	atorvastatin (40 mg/day)	Placebo	Unclear	6	Mean	38	Mean	0	2	36	38
	Xie 2008	atorvastatin (10–20 mg/day)	Placebo	Unclear	12	Mean	Unclear	Unclear	10	18	78	41
	Yamada 2007	atorvastatin (10 mg/day)	Placebo	Unclear	31	Mean	66	Mean	2	6	19	19
	CORONA 2007	rosuvastatin (10 mg/day)	Placebo	Unclear	32.8	Mean	73	Mean	622	669	2514	2497
	GISSI-HF 2008	rosuvastatin (10 mg/day)	Placebo	Unclear	46.8	Mean	68	Mean	629	634	2285	2289
	Krum 2007	rosuvastatin (10–40 mg/day)	Placebo	Unclear	6.1	Mean	61	Mean	0	3	40	45
	Node 2003	simvastatin (5–10 mg/day)	Placebo	Unclear	3.5	Mean	54	Mean	1	1	24	27

Review author, year, estimate ID	Trials	Intervention: specific drugs and dosing	Comparator: specific drugs and dosing	Country	Length of follow-up (months)	Follow-up measure	Age	Age measure	Number of intervention group events	Number of control group events	Number of intervention patients	Number of control patients
Lipinski, 2009 4615e	Hamaad 2005	atorvastatin (40 mg/day)	Placebo	Unclear	3	Mean	67	Mean	0	1	13	10
	Sola 2006	atorvastatin (20 mg/day)	Placebo	Unclear	12	Mean	53	Mean	8	13	54	54
	Vrtovec 2008	atorvastatin (10 mg/day)	Placebo	Unclear	12	Mean	63	Mean	8	20	55	55
	Wojnicz 2006	atorvastatin (40 mg/day)	Placebo	Unclear	6	Mean	38	Mean	0	2	36	38
	Xie 2008	atorvastatin (10–20 mg/day)	Placebo	Unclear	12	Mean	NR	Mean	10	18	78	41
	Yamada 2007	atorvastatin (10 mg/day)	Placebo	Unclear	31	Mean	66	Mean	2	6	19	19
Review author, year, estimate ID	Trials	Intervention: specific drugs and dosing	Comparator: specific drugs and dosing	Country	Length of follow-up (months)	Follow-up measure	Age	Age measure	Number of intervention group events	Number of control group events	Number of intervention patients	Number of control patients
Liu, 2014 4629a	SENIORS 2009	Nebivolol starting with 1.25mg/d to 10mg/d	No beta-blocker	Unclear	21	Mean	76	Mean	127	130	380	372
	J-DHF 2013	Carvedilol 7.5 mg/d	No beta-blocker	Unclear	38.4	Mean	72	Mean	21	27	120	125
Review author, year, estimate ID	Trials	Intervention: specific drugs and dosing	Comparator: specific drugs and dosing	Country	Length of follow-up (months)	Follow-up measure	Age	Age measure	Number of intervention group events	Number of control group events	Number of intervention patients	Number of control patients
Makani, 2013 4838a	CHARM Added 2003	ACE inhibitor+candesartan	Any ACE inhibitor	Unclear	41.4	Mean	64	Mean	309	356	1276	1272
	Cice 2010	ACE inhibitor+telmisartan	Any ACE inhibitor	Unclear	36.3	Mean	63	Mean	56	92	165	167
	Val HeFT 2001	ACE inhibitor+valsartan	Any ACE inhibitor	Unclear	23.3	Mean	63	Mean	923	1189	2511	2499
	ALTITUDE 2012	ACE inhibitor or ARB+aliskiren	Aliskiren	Unclear	32.3	Mean	65	Mean	205	219	4274	4287
	ONTARGET 2008	Ramipril+telmisartan	Ramipril or telmisartan	Unclear	56.5	Mean	67	Mean	332	748	8502	17118

Review author, year, estimate ID	Trials	Intervention: specific drugs and dosing	Comparator: specific drugs and dosing	Country	Length of follow-up (months)	Follow-up measure	Age	Age measure	Number of intervention group events	Number of control group events	Number of intervention patients	Number of control patients
Makani, 2013 4838b	CHARM Added 2003	Any ACE inhibitor and candesartan	Any ACE inhibitor	Unclear	44.5	Mean	64	Mean	309	356	1276	1272
	Cice 2010	Any ACE inhibitor and telmisartan	Any ACE inhibitor	Unclear	39	Mean	63	Mean	56	92	162	167
	Val HeFT 2001	Any ACE inhibitor and valsartan	Any ACE inhibitor	Unclear	25	Mean	63	Mean	923	1189	2511	2499
Review author, year, estimate ID	Trials	Intervention: specific drugs and dosing	Comparator: specific drugs and dosing	Country	Length of follow-up (months)	Follow-up measure	Age	Age measure	Number of intervention group events	Number of control group events	Number of intervention patients	Number of control patients
Moore, 2006 5371	Chan 2005	oral mycophenolate mofetil 2 g daily plus oral prednisolone (32), 1.5g daily at 6 months, and 1 g daily at 12 months	oral cyclophosphamide 2.5 mg/kg/day plus prednisolone (30), replaced by azathioprine at 6 months	Unclear	84	Maximum duration	37	Mean	2	9	30	32
	Ginzler 2005	Oral mycophenolate mofetil 1 g daily to maximum of 3 g daily	IV cyclophosphamide 0.5 g/sq metre increasing to 1.0 g/sq metre, at monthly intervals	Unclear	3 to 6	Follow-up duration	32	Mean	0	7	83	75
Review author, year, estimate ID	Trials	Intervention: specific drugs and dosing	Comparator: specific drugs and dosing	Country	Length of follow-up (months)	Follow-up measure	Age	Age measure	Number of intervention group events	Number of control group events	Number of intervention patients	Number of control patients
Rodrigo, 1999 6564a	Stein and Cole 4	IV methylprednisolone (125 mg) within 30 min of arrival in the emergency department	IV Placebo	Unclear	Range of 2 to 6 hours in the emergency department	Follow-up duration	Unclear	Unclear	8	6	44	47
	Rodrigo and Rodrigo 5	IV methylprednisolone (500 mg) within 30 min of arrival in the emergency department	IV Placebo	Unclear		Follow-up duration	Unclear	Unclear	5	4	49	49
	Lin et al 6	IV methylprednisolone (125 mg) within 30 min of arrival in the emergency department	IV Placebo	Unclear		Follow-up duration	Unclear	Unclear	7	4	23	22

	Littenberg and Gluck 30	IV methylprednisolone (125 mg) within 30 min of arrival in the emergency department	IV placebo	Unclear		Follow-up duration	Unclear	Unclear	9	23	48	49
	Schneider et al 33	IV methylprednisolone (30 mg/kg) within 30 min of arrival in the emergency department	IV placebo	Unclear		Follow-up duration	Unclear	Unclear	5	12	27	27
Review author, year, estimate ID	Trials	Intervention: specific drugs and dosing	Comparator: specific drugs and dosing	Country	Length of follow-up (months)	Follow-up measure	Age	Age measure	Number of intervention group events	Number of control group events	Number of intervention patients	Number of control patients
Rodrigo, 2005 6568a	Cydulka and Emerman (1994)	Glycopyrrolate 2 mg nebulised (once) and Salbutamol 2.5 mg every 60 min nebulised (three times)	Salbutamol 2.5 mg every 60 min nebulised (three times)	USA	Length of emergency department stay, exact lengths not reported	Unclear	Over 18	Cut-off	18	15	60	65
	FitzGerald et al (1997)	Ipratropium bromide 0.5 mg nebulised (once) and Salbutamol 3 mg nebulised (once)	Salbutamol 3 mg nebulised (once)	Canada	Length of emergency department stay, exact lengths not reported	Unclear	18 to 50	Range	9	17	156	155
	Lin et al (1998)	Ipratropium bromide 0.5 mg nebulised (once) and Salbutamol 2.5 mg every 20 min nebulised (three times)	Salbutamol 2.5 mg every 20 min nebulised (three times)	USA	Length of emergency department stay, exact lengths not reported	Unclear	over 18	Cut-off	3	10	27	28
	Karpel et al (1996)	Ipratropium bromide 0.5 mg every 45 min nebulised (twice) and Salbutamol 2.5 mg every 45 min nebulised (twice)	Salbutamol 2.5 mg every 45 min nebulised (twice)	USA	Length of emergency department stay, exact lengths not reported	Unclear	18 to 55	Range	22	25	192	192
	Garret et al (1997)	Ipratropium bromide 0.5 mg every 45 min nebulised (twice) and Salbutamol 2.5 mg every 45 min nebulised (twice)	Salbutamol 2.5 mg every 45 min nebulised (twice)	New Zealand	Length of emergency department stay, exact lengths not reported	Unclear	18 to 55	Range	26	37	171	167

	Weber et al (1999)	Ipratropium bromide 1 mg every 60 min nebulised (three times) and Salbutamol 10 mg every 60 min nebulised (three times)	Salbutamol 10 mg every 60 min nebulised (three times)	USA	Length of emergency department stay, exact lengths not reported	Unclear	over 18	Cut-off	8	13	34	33
	Rodrigo and Rodrigo (2000) (moderate]	Ipratropium bromide 0.08 mg every 10 min metered dose inhaler (for three hours) and Salbutamol 0.4 mg every 10 min metered dose inhaler (for three hours)	Salbutamol 0.4 mg every 10 min metered dose inhaler (for three hours)	Uruguay	Length of emergency department stay, exact lengths not reported	Unclear	18 to 50	Range	2	6	28	22
	Rodrigo and Rodrigo (1995)	Ipratropium bromide 0.08 mg every 10 min metered dose inhaler (three times) and Salbutamol 0.4 mg every 10 min metered dose inhaler (three times)	Salbutamol 0.4 mg every 10 min metered dose inhaler (for three hours)	Uruguay	Length of emergency department stay, exact lengths not reported	Unclear	18 to 50	Range	1	3	11	11
	Nakano (2000)	Oxitropium bromide 0.4 mg every 20 min metered dose inhaler (for three hours) and Salbutamol 0.4 mg every 20 min metered dose inhaler (for three hours)	Salbutamol 0.4 mg every 20 min metered dose inhaler (for three hours)	Japan	Length of emergency department stay, exact lengths not reported	Unclear	over 18	Cut-off	5	10	38	36
	Rodrigo and Rodrigo (2000) [severe]	Ipratropium bromide 0.08 mg every 10 min metered dose inhaler (for three hours) and Salbutamol 0.4 mg every 10 min metered dose inhaler (for three hours)	Salbutamol 0.4 mg every 10 min metered dose inhaler (for three hours)	Uruguay	Length of emergency department stay, exact lengths not reported	Unclear	18 to 50	Range	16	30	60	70
Review author, year, estimate ID	Trials	Intervention: specific drugs and dosing	Comparator: specific drugs and dosing	Country	Length of follow-up (months)	Follow-up measure	Age	Age measure	Number of intervention group events	Number of control group events	Number of intervention patients	Number of control patients
Rowe, 2000 6670	Bloch et al 40	2-g loading dose over 20 minutes, within 30 minutes of arrival to the emergency department	50 mL saline solution	USA	Length of emergency department stay, exact lengths not reported	Unclear	18 to 65	Range	7	11	21	14

	Skobeloff et al 38	1.2-g loading dose over 20 minutes, within 90 minutes of arrival to the emergency department	50 mL saline solution	USA	Length of emergency department stay, exact lengths not reported	Unclear	18 to 70	Range	7	15	19	19
	Silverman et al 42	2-g loading dose over 20 minutes, within 30 minutes of arrival to the emergency department	50 mL saline solution	USA	Length of emergency department stay, exact lengths not reported	Unclear	18 to 60	Range	unclear	127	122	127
Review author, year, estimate ID	Trials	Intervention: specific drugs and dosing	Comparator: specific drugs and dosing	Country	Length of follow-up (months)	Follow-up measure	Age	Age measure	Number of intervention group events	Number of control group events	Number of intervention patients	Number of control patients
Saha , 2007 6746b	HOPE 2000	Ramipril	Placebo	Unclear	12	Mean	66	Mean	141	160	4645	4652
	EUROPA 2003	Perindopril	Placebo	Unclear	12	Mean	60	Mean	63	103	6110	6108
	PEACE 2004	Trandolapril	Placebo	Unclear	12	Mean	64	Mean	105	134	4158	4132
Review author, year, estimate ID	Trials	Intervention: specific drugs and dosing	Comparator: specific drugs and dosing	Country	Length of follow-up (months)	Follow-up measure	Age	Age measure	Number of intervention group events	Number of control group events	Number of intervention patients	Number of control patients
Sampson, 2013 6806f	Remington 2011	Intermittent: “same daily dose administered every other day” (n = 6 risperidone, n = 11 olanzapine)	Maintenance: “treatment as usual” (n = 8 risperidone, n = 8 olanzapine, n = 2 loxapine)	Canada	6	Follow-up duration	39	Mean	1	4	17	18
	Herz 1991*	Intermittent: received placebo injection, mean daily dose 149.7 mg ± 179.3 mg chlorpromazine equivalents (average cumulative antipsychotic drug dosage during prodromal episodes over two years = 487.19 mg ± 370.68 mg)	Maintenance: usual dose of antipsychotic medication mean daily dose 290.0 mg ± 146.7 mg chlorpromazine equivalents (average cumulative antipsychotic drug dosage during prodromal episodes over two years = 424.84 mg ± 333.05 mg)	USA	24	Follow-up duration	36	Mean	12	8	50	51

	Carpenter 1990*	Intermittent: targeted administration of medication: medication administered on an 'as-needed basis' to participants who were otherwise drug-free. Participants remained drug-free until symptoms appeared that were suggestive of a prodromal phase of a psychotic episode, mean daily dose 173 mg ± 69 mg chlorpromazine/4.4 mg ± 1.1 mg haloperidol equivalents	Maintenance: continuous administration of medication: continued receiving medication - dose levels were raised when prodromal symptoms appeared and lowered when re-stabilisation occurred, mean daily dose of 433 mg ± 46 mg chlorpromazine/11.8 mg ± 4.4 mg haloperidol equivalents	USA	24	Follow-up duration	28.1	Mean	30	21	57	59
	Carpenter 1987	Intermittent: targeted medication: medication administered on an 'as-needed basis' to participants who were otherwise drug-free. Antipsychotic treatment was initiated at moderate to high doses when prodromal experiences occurred, and discontinued when the participant returned to a stable clinical state. Participants were treated within context of psychosocial intervention and were assigned to a primary therapist (e.g. psychiatric social worker or masters-level psychologist) for weekly sessions of approximately 45 minutes - mean daily dose 196 mg ± 163 mg (chlorpromazine equivalents)	Maintenance: continuous medication: minimum daily chlorpromazine-equivalent doses of 300mg* were administered combined with brief visits with a pharmacotherapist on alternate weeks - mean daily dose 720 mg ± 732 mg (chlorpromazine equivalents)	USA	24	Follow-up duration	31	Mean	11	10	21	21

	Schooler 1997	Intermittent: fluphenazine decanoate: targeted, early intervention - an injection of sesame oil or miglioil “vehicle” (placebo) every 2 weeks (if subjects showed prodromal signs of relapse, open-label rescue medication was added; either oral fluphenazine or fluphenazine decanoate)	Maintenance: fluphenazine decanoate: continuous standard dose - 12.5-50mg every 2 weeks, n = 52 (AFM) n = 55 (SFM); Maintenance: fluphenazine decanoate: continuous low dose - 2.5-10mg every 2 weeks, n = 54 (AFM) n = 52 (SFM)	USA	24	Follow-up duration	36.5	Mean	46	54	100	213
	Jolley 1989/1990	Intermittent: equivalent doses of placebo injections, with brief intermittent course of antipsychotics begun at the earliest sign of relapse (prodromal symptoms - appearance of non-psychotic symptoms), average cumulative antipsychotic drug dosage (haloperidol equivalents) over two years = 298 mg ± 249 mg	Maintenance: continued to receive fluphenazine decanoate in clinically optimal (pretrial) doses, average cumulative antipsychotic drug dosage (mg haloperidol equivalents) over two years = 1616 mg ± 598 mg	UK	24	Follow-up duration	41	Mean	11	4	27	27
Review author, year, estimate ID	Trials	Intervention: specific drugs and dosing	Comparator: specific drugs and dosing	Country	Length of follow-up (months)	Follow-up measure	Age	Age measure	Number of intervention group events	Number of control group events	Number of intervention patients	Number of control patients
Shan, 2013 7029b	Nannini 2000	3 ml isotonic MgSO4 (7.5 g/100 ml) single dose with b-agonist (salbutamol)	3 ml saline solution. Salbutamol.	Argentina	Length of emergency department stay, exact lengths not reported	Unclear	Over 18	Cut-off	1	1	19	16
	Hughes 2003	2.5 ml isotonic MgSO4 (151 mg/dose)(3 doses at 30 min intervals) with b-agonist 2.5 mg (salbutamol) and 100 mg hydrocortisone intravenous	2.5 ml isotonic saline solution. B-agonist 2.5 mg (salbutamol) and 100 mg hydrocortisone intravenous	New Zealand	Length of emergency department stay, exact lengths not reported	Unclear	16 to 65	Range	12	17	28	24

	Kokturk 2005	Iso-osmolar MgSO4 (6.3%, 145 mg/dose) (20 min intervals) with b-agonist 2.5 mg (salbutamol) and 1 mg/kg methylprednisolone intravenous	2.5 ml isotonic saline solution b-agonist 2.5 mg (salbutamol) and 1 mg/kg methylprednisolone intravenous	Turkey	Length of emergency department stay, exact lengths not reported	Unclear	18 to 60	Range	1	2	14	12
	Aggarwal 2006	1 ml MgSO4 (500 mg) (3 doses, 20 min apart) with b-agonist (salbutamol) 1 ml or more and intravenous hydrocortisone at discretion of physician	1.5 ml distilled water 7.5 ml normal saline b-agonist (Salbutamol) 1 ml or more and intravenous hydrocortisone at discretion of physician	India	Length of emergency department stay, exact lengths not reported	Unclear	13 to 60	Range	9	10	50	50
	Drobina 2006	125 mg MgSO4 0.25 ml of 50% solution (3 doses, 20 min apart) with b-agonist 5 mg/ml (albuterol) and 50 mg oral prednisolone	0.25 ml saline solution b-agonist 5 mg/ml albuterol and 50 mg oral prednisolone	USA	Length of emergency department stay, exact lengths not reported	Unclear	12 to 60	Range	2	1	60	50
	allegos-Solorzano 2010	3 ml (333 mg) of 10% isotonic MgSO4 (3 doses, 20 min apart) with b-agonist 7.5 mg of (albuterol) and 125 mg methylprednisolone intravenous	3 ml of isotonic saline solution and b-agonist 7.5 mg of albuterol and 125 mg methylprednisolone intravenous.	Mexico	Length of emergency department stay, exact lengths not reported	Unclear	over 18	Cut-off	5	13	30	30
Review author, year, estimate ID	Trials	Intervention: specific drugs and dosing	Comparator: specific drugs and dosing	Country	Length of follow-up (months)	Follow-up measure	Age	Age measure	Number of intervention group events	Number of control group events	Number of intervention patients	Number of control patients
Stratton, 2013 7484a	Chapman et al. (2009)	Nova source liquid (Novartis) + dietary advice (oral nutritional supplement given several hours after meals in divided doses) 475 kcal, 21.4 g protein. oral nutritional supplement energy density 2 kcal/ml.	No treatment group (standard care + placebo testosterone + dietary advice)	Unclear	12	Follow-up duration	65 or older	Cut off	4	5	13	13
	Gariballa et al. (2006)	Liquid oral nutritional supplement 995 kcal, 49.75 g protein. oral nutritional supplement energy density 2.49 kcal/ml.	Placebo 60 kcal (0.15 kcal/ml) but no micronutrients/protein	Unclear	1.5	Follow-up duration	65 to 92	Range	65	89	223	222

	Gazzotti et al. (2003)	Clinutren Soup and Clinutren 1.5 (2 supplements/ day) (Nestle) 500 kcal, 21 g (Intake 407 kcal, 16.9 g). oral nutritional supplement energy density (1.25 kcal/ml)	Standard care – no supplementation	Unclear	2	Follow-up duration	75 or older	Cut-off	4	3	39	41
	Miller et al. (2006)	Fortisip (Nutricia) Administered as 4 doses of equal volume. 870–1200 kcal, 34.8–48 g (Intake 583–804 kcal, 23.3–32.2 g, calculated from 67% reported compliance) oral nutritional supplement energy density (1.5 kcal/ml).	Control: (equal number of visits as oral nutritional supplement group when given general advice on nutrition and exercise)	Unclear	1.5	Follow-up duration	70 or older	Cut-off	2	4	25	26
	Norman et al. (2011)	Fresubin Protein energy drink, (Fresenius) plus dietary counselling (Advised to take oral nutritional supplement slowly and in between meals) Up to 900 kcal 60 g (Intake = 2.4 ± 0.8 oral nutritional supplement, calculated as 720 kcal, 48 g) oral nutritional supplement energy density (1.5 kcal/ml)	Dietary counselling only	Unclear	3	Follow-up duration	51	Mean	17	26	60	54
	Price et al. (2005)	Fortisip and Fortifresh (Nutricia) 600 kcal, 24 g (Intake 372 kcal, 14.9 g calculated from 62% reported compliance) oral nutritional supplement energy density (1.5 kcal/ml)	Receiving usual care	Unclear	2	Follow-up duration	75	Cut-off	10	17	66	70
Review author, year, estimate ID	Trials	Intervention: specific drugs and dosing	Comparator: specific drugs and dosing	Country	Length of follow-up (months)	Follow-up measure	Age	Age measure	Number of intervention group events	Number of control group events	Number of intervention patients	Number of control patients
Kew, 2014 4004e	Verhoeven 2002	Fluticasone propionate 500 mcg twice daily	Placebo	Netherlands	6	Follow-up duration	55.13	Mean	0	0	10	13
	Mahler 2002	Fluticasone 500 mcg twice daily	Placebo	Unclear	6	Follow-up duration	64.193	Mean	2	0	168	181
	Hanania 2003	Fluticasone propionate 250 mcg twice daily	Placebo	USA	6	Follow-up duration	64.005	Mean	1	0	183	185
	Calverley 2003 TRISTAN	Fluticasone 500 mcg twice daily	Placebo	25 countries	12	Follow-up duration	63.451	Mean	9	3	375	363

	GSK FLTA3025 2005	Fluticasone propionate 250 mcg or 500 mcg twice daily	Placebo	USA	6	Follow-up duration	64.424	Mean	4	1	434	206
	GSK SCO30002 2005	Fluticasone propionate 500 mcg twice daily	Placebo	Italy and Poland	12	Follow-up duration	65.137	Mean	1	1	131	125
	Calverley 2007 TORCH	Fluticasone 500 mcg twice daily	Placebo	42 countries	36	Follow-up duration	65	Mean	121	69	1552	1545
	GSK SCO104925 2008	Fluticasone propionate 500 mcg twice daily	Placebo	4 centres in the Russian Federation, 4 centres in the United States, 2 centres in Chile and 1 centre in Estonia	3	Follow-up duration	64.7	Mean	1	0	42	42
	Lapperre 2009	Fluticasone 500 mcg twice daily	Placebo	Netherlands	30	Follow-up duration	60.418	Mean	0	2	26	29
	Kerwin 2013	Fluticasone furoate 100 mcg daily	Placebo	Chile, Estonia, Germany, Japan, Korea, Phillipines, Poland, Russian Federation and the United States	6	Follow-up duration	62.399	Mean	2	1	206	207
	Martinez 2013	Fluticasone furoate 100 mcg or 200 mcg daily	Placebo	Czech Republic,Germany, Japan, Poland, Romania, Russian Federation, Ukraine and the United States	6	Follow-up duration	61.833	Mean	2	0	407	205
	Mahler 2002	Fluticasone/Salmeterol combination 500/50 mcg twice daily	Salmeterol 50 mcg twice daily	65 centres	6	Follow-up duration	62.688	Mean	2	0	165	160
	Calverley 2003 TRISTAN	Fluticasone/salmeterol 500/50 mcg twice daily	Salmeterol 50 mcg twice daily	25 countries	12	Follow-up duration	62.955	Mean	7	9	358	373
	Hanania 2003	Fluticasone/salmeterol 250/50 mcg twice daily	Salmeterol 50 mcg twice daily	USA	6	Follow-up duration	63.499	Mean	0	1	178	177

	GSK SCO100470 2006	Salmeterol/fluticasone propionate 50/250 mcg twice daily	Salmeterol 50 mcg twice daily	Australia (10), Bulgaria (5),Croatia (1),Czech Republic (8), France (14), Germany (18), Greece (4), Italy (16), Latvia (5), Lithuania (2), Netherlands (12), Philippines (3), Poland (5), Romania (3), Russian Federation (8) , Slovakia (4), Slovenia (4), Sweden (4), Thailand (4) and United Kingdom (5)	6	Follow-up duration	63.601	Mean	2	4	518	532
	Kardos 2007	Salmeterol/fluticasone 50/500 mcg twice daily	Salmeterol 50 mcg twice daily	Germany	10	Follow-up duration	63.898	Mean	13	3	507	487
	Calverley 2007 TORCH	Fluticasone/salmeterol 500/50 mcg twice daily	Salmeterol 50 mcg twice daily	42 countries	36	Follow-up duration	65.05	Mean	138	82	1546	1542
	GSK SCO40041 2008	Fluticasone propionate/salmeterol 250/50 mcg twice daily	Salmeterol 50 mcg twice daily	USA	36	Follow-up duration	65.653	Mean	5	4	92	94
	GSK SCO104925 2008	Fluticasone/salmeterol combination 500/50 mcg twice daily	Salmeterol 50 mcg twice daily	4 centres in the Russian Federation, 4 centres in the United States, 2 centres in Chile and 1 centre in Estonia	36	Follow-up duration	63.797	Mean	0	0	39	38
	Ferguson 2008	Fluticasone/salmeterol 250/50 mcg twice daily	Salmterol 50 mcg twice daily	USA and Canada	12	Follow-up duration	64.95	Mean	19	10	394	388
	Anzueto 2009	Fluticasone/salmeterol 250/50 mcg twice daily	Salmeterol 50 mcg twice daily	USA and Canada	12	Follow-up duration	65.349	Mean	13	8	394	403

	Kerwin 2013	Fluticasone 50 mcg or 100 mcg/ vilanterol 25 mcg daily	Fluticasone 100 mcg/ vilanterol 25 mcg daily	Chile, Estonia, Germany, Japan, Korea, Phillipines, Poland, Russian Federation and the United States	6	Follow-up duration	62.832	Mean	2	3	412	205
	Dransfield 2013	Fluticasone furoate/vilanterol 50/25 mcg daily; 100/25 mcg daily; or 200/25 mcg daily	Vilanterol 25 daily	Argentina, Australia, Canada, Chile, Estonia, Germany, Italy, Mexico, Netherlands, Peru, Phillipines, South Africa, Sweden, the United Kingdom and the United States	12	Follow-up duration	63.675	Mean	72	8	2437	818
	Martinez 2013	Fluticasone furoate 100 mcg or 200 mcg/vilanterol 25 mcg daily	Vilanterol 25 mcg	Czech Republic,Germany, Japan, Poland, Romania, Russian Federation, Ukraine and the United States	6	Follow-up duration	61.4	Mean	3	2	409	203

Review author, year, estimate ID	Trials	Intervention: specific drugs and dosing	Comparator: specific drugs and dosing	Country	Length of follow-up (months)	Follow-up measure	Age	Age measure	Number of intervention group events	Number of control group events	Number of intervention patients	Number of control patients
Baumeister, 2011<br>593	MIND-IT 2007	Mirtazapine (30 to 45 mg/day, patients that did not respond and patients with relapse were offered open treatment with citalopram)	Placebo	Netherlands	5.58	Follow-up duration	57.229	Mean	8	10	47	44
	SADHART 2002	Sertraline 50 mg/d for the first 6 weeks, up to 100 mg/d for weeks 6-10, up to 150 mg/d for weeks 10-12, up to 200 mg/d for weeks 12-24	Placebo	USA, Europe, Canada, Australia	5.58	Follow-up duration	57.197	Mean	55	76	186	183

	Strik 2000	Fluoxetine (acute treatment period of 9 weeks, and continuation period of 16 weeks; 20 to 60 mg/d)	Placebo	Netherlands	5.8	Follow-up duration	56.4	Mean	1	6	27	27
Review author, year, estimate ID	Trials	Intervention: specific drugs and dosing	Comparator: specific drugs and dosing	Country	Length of follow-up (months)	Follow-up measure	Age	Age measure	Number of intervention group events	Number of control group events	Number of intervention patients	Number of control patients
Campschroer, 2014 1164	Autorino 2005	Tamsulosin: 0.4 mg once/day and standard therapy (Diclofenac: 100 mg/d)	Standard therapy (Aescin (an anti-oedema extract of the horse chestnut tree): 80 mg/day and Diclofenac: 100 mg/day)	Italy	0.46	Follow-up duration	45.438	Mean	5	11	50	46
	Erturhan 2007	Tamsulosin 0.4 mg/day plus prophylatic antibiotic therapy (cefuroxime axetil 250mg (OD) and 2500ml hydration daily	Prophylatic antibiotic therapy (cefuroxime axetil 250mg OD and 2500ml hydration)	Turkey	0.7	Follow-up duration	32.05	Mean	1	2	30	30
	Yencilek 2010	Tamsulosin: 0.4 mg once/day and standard care (Hyoscine-N-butylbromide: 10 mg 3 times/day)	Standard therapy (Hyoscine-N-butylbromide: 10 mg 3 times/day)	Turkey	0.93	Follow-up duration	34.139	Mean	14	26	42	50
	Zehri 2010	Doxazosin: 2 mg 1 hour before sleep for a maximum of 28 days and standard therapy (Both groups received 50 mg diclofenac twice/d for a maximum of 10 days and were allowed to drink 2 L of fluids daily)	No treatment and standard therapy (Both groups received 50 mg diclofenac twice/d for a maximum of 10 days and were allowed to drink 2 L of fluids daily)	Pakistan	0.93	Follow-up duration	33.092	Mean	1	20	33	32
Review author, year, estimate ID	Trials	Intervention: specific drugs and dosing	Comparator: specific drugs and dosing	Country	Length of follow-up (months)	Follow-up measure	Age	Age measure	Number of intervention group events	Number of control group events	Number of intervention patients	Number of control patients
Chong, 2012 1438a	Briggs 2005	iotropium, 18 µg once daily via the HandiHaler device	Salmeterol, 2 actuations of 25 µg each, twice daily via a metered dose inhaler	Finland, Greece, Italy, Portugal, Sweden, Turkey, the United Kingdom and the United States	3	Follow-up duration	64.399	Mean	4	9	328	325

	Brusasco 2003	18 µg of tiotropium once daily, delivered through the HandiHaler inhalation device, plus MDI placebo	50 µg of salmeterol twice daily, delivered through a pressurised, metered-dose inhaler, plus HandiHaler placebo	18 countries	6	Follow-up duration	63.951	Mean	12	20	402	405
	Vogelmeier 2008	Tiotropium, 18 µg once daily via the HandiHaler device + placebo bid via MDDPI	Formoterol 10 µg twice daily (bid) via multi-dose dry powder inhaler	Germany, Italy, Netherlands, Russian federation, Poland, Czech Republic, Spain and Hungary	6	Follow-up duration	62.62	Mean	9	8	221	210
	Vogelmeier 2011	18 µg of tiotropium once daily, delivered through the HandiHaler inhalation device	50 µg of salmeterol twice daily, delivered through a pressurised, metered-dose inhaler	25 countries	12	Follow-up duration	62.85	Mean	503	553	3707	3669
Review author, year, estimate ID	Trials	Intervention: specific drugs and dosing	Comparator: specific drugs and dosing	Country	Length of follow-up (months)	Follow-up measure	Age	Age measure	Number of intervention group events	Number of control group events	Number of intervention patients	Number of control patients
Grimwade, 2009 2933a	Anglaret 1999	980mg cotrimoxazole daily	placebo	Unclear	Unclear	Unclear	33	Mean	0	0	1	1
	Maynart 2001	480mg cotrimoxazole daily	placebo	Unclear	Unclear	Unclear	36	Median	22	15	51	49
	Wiktor 1999	980mg cotrimoxazole daily	placebo	Unclear	Unclear	Unclear	32	Median	29	47	383	279
Review author, year, estimate ID	Trials	Intervention: specific drugs and dosing	Comparator: specific drugs and dosing	Country	Length of follow-up (months)	Follow-up measure	Age	Age measure	Number of intervention group events	Number of control group events	Number of intervention patients	Number of control patients
Heran, 2012 3275d	ARCH-J 2003	Candesartan 8 mg once daily	Placebo	Japan	6	Follow-up duration	63.7	Mean	8	17	148	144
	SPICE 2000	Candesartan, target dose 16 mg once daily	Placebo	Unclear	2.79	Follow-up duration	65.7	Mean	15	11	179	91
	CHARM-Alternative 2003	Candesartan, target dose 32 mg once daily	Placebo	Unclear	33.7	Median	68.3	Mean	207	286	1013	1015

Review author, year, estimate ID	Trials	Intervention: specific drugs and dosing	Comparator: specific drugs and dosing	Country	Length of follow-up (months)	Follow-up measure	Age	Age measure	Number of intervention group events	Number of control group events	Number of intervention patients	Number of control patients
Heran, 2012 3275e	CHARM-Preserved 2003	Candesartan, target dose 32 mg once daily	Placebo	Unclear	36.6	Median	67.2	Mean	682	643	1514	1509
	I-PRESERVE 2008	Irbesartan, target dose 300 mg once daily	Placebo	Unclear	49.5	Follow-up duration	72	Mean	827	790	2067	2061
	SPICE 2000	Candesartan, target dose 16 mg once daily	Placebo	Unclear	2.79	Follow-up duration	65.7	Mean	8	6	179	91
	CHARM-Alternative 2003	Candesartan, target dose 32 mg once daily	Placebo	Unclear	33.7	Median	68.3	Mean	403	357	1013	1015
Review author, year, estimate ID	Trials	Intervention: specific drugs and dosing	Comparator: specific drugs and dosing	Country	Length of follow-up (months)	Follow-up measure	Age	Age measure	Number of intervention group events	Number of control group events	Number of intervention patients	Number of control patients
Heran, 2012 3275l	Tonkon 2000	Irbesartan, target dose 150 mg once daily	Placebo	USA	2.79	Follow-up duration	63.9	Mean	2	1	57	52
	RESOLVD 1999	Candesartan 4 or 8 mg OD plus enalapril 10 mg twice daily	Enalapril 10 mg twice daily	Unclear	10	Follow-up duration	63.1	Mean	31	7	332	109
	CHARM-Added 2003	Candesartan, target dose 32 mg once daily	Placebo	Unclear	36	Mean	64.1	Mean	309	356	1276	1272
	Val-HeFT 2001	Valsartan 160 mg twice daily	Placebo	Unclear	23	Follow-up duration	62.7	Mean	346	455	2511	2499
Review author, year, estimate ID	Trials	Intervention: specific drugs and dosing	Comparator: specific drugs and dosing	Country	Length of follow-up (months)	Follow-up measure	Age	Age measure	Number of intervention group events	Number of control group events	Number of intervention patients	Number of control patients
Hood, 2014 3415	Dig captopril 1988	Digoxin 0.125 to 0.375 mg/d titrated to serum levels of 0.7 to 2.5 ng/mL	placebo	Unclear	6	Follow-up duration	58	Mean	3	11	96	100
	DIG study 1997	Digoxin median baseline dosage in main trial of 0.25 mg/d	placebo	Unclear	37	Mean	63	Mean	910	1180	3397	3403
	PROVED 1993	Digoxin dosage titrated to mean serum level of 1.2 ng/mL, median digoxin dosage 0.375 mg/d	placebo	Unclear	3	Follow-up duration	64	Mean	3	6	42	46

	RADIANCE 1993	Digoxin dosage titrated to mean serum level of 1.2 ng/mL, mean dig dosage 0.38 mg/d	Placebo	Unclear	3	Study duration, mean/median follow-up not reported	60	Mean	2	9	85	93

Review author, year, estimate ID	Trials	Intervention: specific drugs and dosing	Comparator: specific drugs and dosing	Country	Length of follow-up (days)	Follow-up measure	Age	Age measure	Number of intervention group events	Number of control group events	Number of intervention patients	Number of control patients
Kew, 2014 4002a	Bilaceroglu 2001	IV MgSO4 group: Group 1 given salbutamol + 2 mg IV MgSO4 in 100 cc dextrose solution. Group 2 given salbutamol (2.5 mg nebulised) + corticosteroid (125 mg prednisolone) + 2 mg MgSO4 in 100 cc dextrose solution. Both groups given treatment at the 30th minute of their arrival	Group 1 given salbutamol + placebo (100 cc of 5% dextrose solution). Group 2 given salbutamol (2.5 mg nebulised) + corticosteroid (125 mg prednisolone) + placebo (100 cc of 5% dextrose solution). In both groups, these were given at the 30th minute of the participant’s arrival	Turkey	0.25	Follow-up duration	Unclear	Unclear	10	17	40	41
	Bloch 1995	2 g intravenous magnesium sulfate in 50 mL 0.9% normal saline given 30 minutes after entry and infused over 20 minutes	50 mL of 0.9% normal saline given 30 minutes after entry and infused over 20 minutes	USA	0.0054825	Follow-up duration	Unclear	Unclear	17	24	67	68
	Boonyavorakul 2000	2 g of magnesium sulfate in 50 mL 0.9% normal saline	2 mL of sterile water in 50 mL 0.9% normal saline	Thailand	0.0054825	Follow-up duration	Unclear	Unclear	3	4	17	16
	Bradshaw 2007	1.2 g intravenous magnesium sulfate in 50 mL 0.9% normal saline infused over 15 minutes	50 mL 0.9% normal saline infused over 15 minutes	Scotland	0.0013706	Follow-up duration	Unclear	Unclear	49	52	62	67
	Goodacre 2013	2 g magnesium sulfate in 100 mL 0.9% normal saline infused over 20 minutes	100 mL 0.9% normal saline infused over 20 minutes	UK	0.0054825	Follow-up duration	Unclear	Unclear	279	278	394	358
	Green 1992	IV MgSO4 Group: 2 g IV MgSO4 in 50 mL D5W over 20 minutes within 45 minutes of treatment initiation	Control group: no IV MgSO4 given	USA	Unclear		Unclear	Unclear	13	11	58	62

	Matusiewicz 1994	IV MgSO4 group: 1.2 mg IV MgSO4 in 50 mL 0.9% normal saline infused over 15 minutes<br>Co-interventions: All participants received 5 mg nebulised salbutamol, 500 mcg nebulised ipratropium bromide, oxygen, 200 mg IV hydrocortisone. Aminophylline was given at the discretion of the attending physician	Control group: 50 mL 0.9% normal saline infused over 15 minutes	Scotland	Unclear		Unclear	Unclear	45	47	64	67
	Porter 2001	2 g magnesium sulfate in 50 mL 0.9% normal saline given immediately	50 mL 0.9% normal saline given immediately	USA	0.0013706	Follow-up duration	Unclear	Unclear	5	5	18	24
	Silverman 2002	2 g intravenous magnesium sulfate in 50 mL 0.9% normal saline infused over 10 to 15 minutes and given at 30 minutes	50 mL 'like appearing solution' infused over 10 to 15 minutes and given at 30 minutes	USA	0.0054825	Follow-up duration	Unclear	Unclear	39	41	122	126
	Singh 2008	2 g intravenous magnesium sulfate in 250 mL 0.9% normal saline infused over 20 minutes at 30 minutes	250 mL 0.9% normal saline infused over 20 minutes at 30 minutes	India	0.0041118	Follow-up duration	Unclear	Unclear	2	9	30	30
	Skobeloff 1989	1.2 g intravenous magnesium sulfate in 50 mL of 0.9% normal saline infused over 20 minutes	50 mL 0.9% normal saline infused over 20 minutes	USA	0.0054825	Follow-up duration	Unclear	Unclear	7	15	19	19
Review author, year, estimate ID	Trials	Intervention: specific drugs and dosing	Comparator: specific drugs and dosing	Country	Length of follow-up (days)	Follow-up measure	Age	Age measure	Number of intervention group events	Number of control group events	Number of intervention patients	Number of control patients
Kew, 2014 4002h	Bloch 1995 (2)	2 g intravenous magnesium sulfate in 50 mL 0.9% normal saline given 30 minutes after entry and infused over 20 minutes	50 mL of 0.9% normal saline given 30 minutes after entry and infused over 20 minutes	USA	0.0054825	Follow-up duration	Unclear	Unclear	17	24	67	68
	Boonyavorakul 2000	2 g of magnesium sulfate in 50 mL 0.9% normal saline	2 mL of sterile water in 50 mL 0.9% normal saline	Thailand	0.0054825	Follow-up duration	Unclear	Unclear	3	4	17	16
	Bradshaw 2007 (3)	1.2 g intravenous magnesium sulfate in 50 mL 0.9% normal saline infused over 15 minutes	50 mL 0.9% normal saline infused over 15 minutes	Scotland	0.0013706	Follow-up duration	Unclear	Unclear	49	52	62	67

	Goodacre 2013	2 g magnesium sulfate in 100 mL 0.9% normal saline infused over 20 minutes	100 mL 0.9% normal saline infused over 20 minutes	UK	0.0054825	Follow-up duration	Unclear	Unclear	279	278	394	358
	Porter 2001	2 g magnesium sulfate in 50 mL 0.9% normal saline given immediately	50 mL 0.9% normal saline given immediately	USA	0.0013706	Follow-up duration	Unclear	Unclear	5	5	18	24
	Silverman 2002	2 g intravenous magnesium sulfate in 50 mL 0.9% normal saline infused over 10 to 15 minutes and given at 30 minutes	50 mL 'like appearing solution' infused over 10 to 15 minutes and given at 30 minutes	USA	0.0054825	Follow-up duration	Unclear	Unclear	39	41	122	126
	Singh 2008	2 g intravenous magnesium sulfate in 250 mL 0.9% normal saline infused over 20 minutes at 30 minutes	250 mL 0.9% normal saline infused over 20 minutes at 30 minutes	India	0.0041118	Follow-up duration	Unclear	Unclear	2	9	30	30
	Skobeloff 1989	1.2 g intravenous magnesium sulfate in 50 mL of 0.9% normal saline infused over 20 minutes	50 mL 0.9% normal saline infused over 20 minutes	USA	0.0054825	Follow-up duration	Unclear	Unclear	7	15	19	19

Review author, year, estimate ID	Trials	Intervention: specific drugs and dosing	Comparator: specific drugs and dosing	Country	Length of follow-up (months)	Follow-up measure	Age	Age measure	Number of intervention group events	Number of control group events	Number of intervention patients	Number of control patients
Kew, 2013 4003a	Vogelmeier 2008	Formoterol 10 µg twice daily	Placebo	Germany (30), Italy (19), Netherlands (9), Russian Federation (9), Poland (7), Czech Republic (4), Spain (4) and Hungary (4)	6	Follow-up duration	62.149	Mean	1	3	210	209
	Doherty 2012	Formoterol 10 µg twice daily	Placebo	North, Central and South America, Europe, Africa and Asia	6	Follow-up duration	59.257	Mean	5	12	243	236
	Dahl 2001	Formoterol 12 µg twice daily	Placebo	Denmark, Netherlands, Poland, Russia, UK	12	Follow-up duration	63.678	Mean	2	2	181	86
	Rossi 2002	Formoterol 12 µg twice daily	Placebo	81 centres worldwide	12	Follow-up duration	63	Mean	10	10	211	110

	Tashkin 2012	Formoterol 10 µg twice daily	Placebo	South America, Asia, Africa, Europe andNorth America	6	Follow-up duration	59.197	Mean	6	7	209	212
	Calverley 2003a	Formoterol 12 µg twice daily	Placebo	15 countries or regions	12	Follow-up duration	64.002	Mean	73	79	255	256
	Rossi 2002	Formoterol 24 µg twice daily	Placebo	81 centres worldwide	12	Follow-up duration	62.34	Mean	5	10	214	110
	Dahl 2001	Formoterol 24 µg twice daily	Placebo	Denmark, Netherlands, Poland, Russia, UK	12	Follow-up duration	63.665	Mean	2	2	171	86
	Brusasco 2003	Salmeterol 50 µg twice daily	Placebo	18 countries	6	Follow-up duration	64.348	Mean	20	20	405	400

Review author, year, estimate ID	Trials	Intervention: specific drugs and dosing	Comparator: specific drugs and dosing	Country	Length of follow-up (months)	Follow-up measure	Age	Age measure	Number of intervention group events	Number of control group events	Number of intervention patients	Number of control patients
Kew, 2013 4003e	Dahl 2001	Formoterol 12 µg twice daily	Placebo	Denmark, Netherlands, Poland, Russia, UK	6	Follow-up duration	63.843	Mean	2	4	181	172
	Vogelmeier 2008	Formoterol 10 µg twice daily	Placebo	Germany (30), Italy (19), Netherlands (9), Russian Federation (9), Poland (7), Czech Republic (4), Spain (4) and Hungary (4)	6	Follow-up duration	62.149	Mean	1	3	210	209
	Tashkin 2012	Formoterol 10 µg twice daily	Placebo	South America, Asia, Africa, Europe andNorth America	6	Follow-up duration	59.197	Mean	6	7	209	212
	Doherty 2012	Formoterol 10 µg twice daily	Placebo	North, Central and South America, Europe, Africa and Asia	6	Follow-up duration	59.257	Mean	5	12	243	236
	Rossi 2002	Formoterol 12 µg twice daily	Placebo	81 centres worldwide	12	Follow-up duration	63	Mean	10	20	211	220

	Calverley 2003a	Formoterol 12 µg twice daily	Placebo	15 countries or regions	12	Follow-up duration	64.002	Mean	73	79	255	256
Review author, year, estimate ID	Trials	Intervention: specific drugs and dosing	Comparator: specific drugs and dosing	Country	Length of follow-up (months)	Follow-up measure	Age	Age measure	Number of intervention group events	Number of control group events	Number of intervention patients	Number of control patients
Komossa, 2013 4123b	Stroup 2006	Olanzapine: flexible dose, allowed dose range: 7.5-30 mg/day, mean dose=20.5 mg/ day. N=108.;	Quetiapine: flexible dose, allowed dose range: 200-800 mg/day, mean dose=565.2 mg/day. N=95.	USA	6.5	Follow-up duration	40.047	Mean	12	19	108	95
	Lieberman 2005	Olanzapine: flexible dose, allowed dose range: 7.5-30 mg/day, mean dose=20.1 mg/ day. N=336.	Quetiapine: flexible dose, allowed dose range: 200-800 mg/day, mean dose=543.4 mg/day. N=337.	USA	19.5	Follow-up duration	40.6	Mean	38	68	336	337
Review author, year, estimate ID	Trials	Intervention: specific drugs and dosing	Comparator: specific drugs and dosing	Country	Length of follow-up (months)	Follow-up measure	Age	Age measure	Number of intervention group events	Number of control group events	Number of intervention patients	Number of control patients
Komossa, 2013 4123i	Stroup 2006	Olanzapine: flexible dose, allowed dose range: 7.5-30 mg/day, mean dose=20.5 mg/ day. N=108.;	Ziprasidone: flexible dose, allowed dose range: 40-160 mg/day,mean dose=115.9 mg/ day. N=137	USA	6.5	Follow-up duration	40.727	Mean	12	22	108	137
	Lieberman 2005	Olanzapine: flexible dose, allowed dose range: 7.5-30 mg/day, mean dose=20.1 mg/ day. N=336	Ziprasidone: flexible dose, allowed dose range: 40-160 mg/day,mean dose=112.8 mg/ day. N=185	USA	19.5	Follow-up duration	40.6	Mean	38	33	336	185
Review author, year, estimate ID	Trials	Intervention: specific drugs and dosing	Comparator: specific drugs and dosing	Country	Length of follow-up (months)	Follow-up measure	Age	Age measure	Number of intervention group events	Number of control group events	Number of intervention patients	Number of control patients
Leucht, 2012 4508a	Goldberg 1981	fluphenazine decanoate- Fixed doses. Allowed dose range: n.i. - same dose as before. Mean dose: n.i..	Placebo (all on depot)	Unclear	1.5	Follow-up duration	37	Mean	0	0	14	17

	Keskiner 1968	fluphenazine decanoate - Flexible doses. Allowed dose range: 12.5-75/mg biweekly. Mean dose: n.i..	Placebo	Unclear	3	Follow-up duration	36	Mean	1	2	13	11
	Blackburn 1981	prochlorpromazine, perphenazine, chlorpromazine, promazine or trifluoperazine. Fixed doses continued with the same drug and dose taken before the study. Mean dose: n.i.	Placebo	Unclear	4	Follow-up duration	20-40	Range	0	9	30	15
	Ruskin 1991	haloperidol. Fixed dose (dose before randomisation was maintained). Mean dose: n.i..	Placebo	Unclear	6	Follow-up duration	60.1	Mean	0	1	11	12
	Wistedt 1981	fluphenazine depot (most around 12.5 - 25 mg/3 weeks, mean 21.42mg/3 weeks) or flupenthixol depot (most around 20-40mg/3 weeks, mean 27.5/3 weeks) - Fixed dose.	Placebo	Unclear	6.5	Follow-up duration	43.1	Mean	0	5	24	17
	Beasley 2003	olanzapine - Fixed dose of either 10, 15 or 20 mg/day. Mean dose 13.4 mg/ day.	Placebo	Unclear	7.5	Follow-up duration	35.9	Mean	2	15	224	102
	Chen 2010	quetiapine. Fixed dose of 400mg/day.	Placebo	Hong Kong	12	Follow-up duration	24.2	Unclear	5	14	89	89
	Hirsch 1973	Fixed/flexible dose: Allowed dose range: 25mg/month - no upper limit. Mean dose: 26.4mg/month.	Placebo	Unclear	9	Follow-up duration	43.4	Mean	8	24	41	40
	Hough 2010	paliperidone palmitate depot - Fixed dose: originally 25, 50 or 100mg/4 weeks; this dose was maintained. Mean dose: n.i..	Placebo	Unclear	12	Follow-up duration	39	Mean	3	7	206	204
	Kramer 2007	paliperidone- Flexible doses. Allowed dose range: 3 - 15mg/day Mean dose: 10. 8 mg/day.	Placebo	Unclear	12	Follow-up duration	38.3	Mean	6	13	105	102

	Leff 1971	trifluoperazine or chlorpromazine (depending on the previous medication so that so far as the patient was concerned there was no apparent change in medication) . Flexible dose. Allowed dose range: trifluoperazine 5-25mg/day, chlorpromazine 100-500mg/day.Mean dose: chlorpromazine 157.1 mg/day, trifluoperazine 12.3mg/day.	Placebo	Unclear	12	Follow-up duration	16-55	Range	7	6	20	15
	McCreadie 1989	pimozide once weekly or i.m. flupenthixol. Flexible doses. Allowed dose range: n.i.. Mean dose: n.i..	Placebo	Unclear	12	Follow-up duration	NR	NR	0	4	8	7
	Troshinsky 1962	various phenothiazines, mainly chlorpromazine. Fixed/flexible dose: flexible. Allowed dose range: not limited, but complete discontinuation was not allowed. Mean dose: 150-200mg/day chlorpromazine.	Placebo	Unclear	10.75	Follow-up duration	40-50	Range	0	8	24	19
	Boonstra 2011	olanzapine, risperidone, quetiapine, zuclopenthixol. Flexible doses.Mean dose: n.i.	Placebo	Unclear	24	Follow-up duration	29.8	Mean	1	4	9	11
	Hogarty 1973	chlorpromazine - Flexible dose. Allowed dose range: 100mg/day. Mean dose: ~ 260mg/day.	Placebo	Unclear	24	Follow-up duration	34.4	Mean	50	84	192	182
	Pietzcker 1993	various antipsychotic drugs. Flexible dose, minimum 100mg/day chlorpromazine equivalent. Allowed dose range: 100 - unlimited chlorpromazine equivalents/ day. Mean dose: 201 mg/day.	Taper dosing: Duration of taper: 50% every two weeks, thus after 6 weeks only 12.5% of initial dose left, thus 42 days. Note that participants were not withdrawn after they had received crisis intervention.	Unclear	24	Follow-up duration	34.6	Mean	29	49	122	115

Review author, year, estimate ID	Trials	Intervention: specific drugs and dosing	Comparator: specific drugs and dosing	Country	Length of follow-up (months)	Follow-up measure	Age	Age measure	Number of intervention group events	Number of control group events	Number of intervention patients	Number of control patients
Magee, 2012 4812a	Fr Caribbean 1990	Oxprenolol 80 mg po twice daily (max 320 mg daily)	Placebo	Caribbean	20-36 weeks' gestation until babies birth	Range	Unclear	Unclear	48	46	78	76
	Scotland 1983	Labetalol 100 mg po twice daily (max 1200 mg daily)	Placebo	Scotland	Unclear	Unclear	Unclear	Unclear	16	33	46	39
	Sweden 1984	Metoprolol 50 mg po twice daily (max 200 mg daily)	Placebo 1 table po twice daily	Sweden	Less than 37 weeks' gestation until babies birth	Range	Unclear	Unclear	16	19	26	26
Review author, year, estimate ID	Trials	Intervention: specific drugs and dosing	Comparator: specific drugs and dosing	Country	Length of follow-up (months)	Follow-up measure	Age	Age measure	Number of intervention group events	Number of control group events	Number of intervention patients	Number of control patients
Makrides, 2014 4844	Austria 1997	oral magnesium citrate supplements (365 mg (15 mmol) once daily)	placebo	Austria	Unclear	Unclear	Unclear	Unclear	16	30	240	250
	Italy 1994	15 mmol magnesium hydrochlorate aspartate	placebo	Italy	Unclear	Unclear	Unclear	Unclear	2	5	50	50
	Zurich 1988	15mmol of magnesium aspartate-hydrochloride daily	placebo	Zurich	Unclear	Unclear	Unclear	Unclear	44	65	278	290
Review author, year, estimate ID	Trials	Intervention: specific drugs and dosing	Comparator: specific drugs and dosing	Country	Length of follow-up (months)	Follow-up measure	Age	Age measure	Number of intervention group events	Number of control group events	Number of intervention patients	Number of control patients
Ngo, 2010 5606	Cleland 2005	Treatment: darbepoetin alfa, 2.0, 3.0 or 5.0µg/kg every month (no target)	Placebo	Unclear	2	Follow-up duration	72.5	Mean	3	0	18	6
	Ghali 2008	darbepoetin alfa, starting dose 0.75µg/kg, every 2 weeks to target Hb of 13-15g/dL	Placebo	Unclear	13	Follow-up duration	68.492	Mean	25	31	162	157
	Kourea 2008a	darbepoetin alfa, starting dose 1.5µg/kg, every 20 days to target Hb of 12. 5-14g/dL	Placebo	Unclear	3	Follow-up duration	69.098	Mean	3	5	21	20

	Mancini 2003	erythropoietin, 5000U thrice-weekly to target Hct of 45% and folate 1mg orally daily	Placebo	Unclear	3	Follow-up duration	78.652	Mean	1	4	15	8
	Palazzuoli 2006	beta-EPO, 6000IU twice-weekly to target Hb of 11.5-12g/dL	Placebo	Unclear	12	Follow-up duration	73.421	Mean	4	8	20	18
	Palazzuoli 2007	beta-EPO, 6000IU twice-weekly to target Hb of 12-12.5g/dL	Placebo	Unclear	12	Follow-up duration	73.02	Mean	4	8	26	25
	Parissis 2008	darbepoetin alfa, 1.5mg/kg every 20 days to target Hb of 14g/dL	Placebo	Unclear	3	Follow-up duration	70.969	Mean	2	3	21	11
	Ponikowski 2007	darbepoetin alfa, 0.75mg/kg every 2 weeks to target Hb of 13-15g/dL	Placebo	Unclear	6.5	Follow-up duration	71.073	Mean	2	3	19	22
	van Veldhuisen 2007	darbepoetin alfa, starting dose 0.75μg/kg or 50μg, every 2 weeks to target Hb of 13-15g/dL	Placebo	Unclear	6.5	Follow-up duration	71	Mean	4	4	110	55
Review author, year, estimate ID	Trials	Intervention: specific drugs and dosing	Comparator: specific drugs and dosing	Country	Length of follow-up (months)	Follow-up measure	Age	Age measure	Number of intervention group events	Number of control group events	Number of intervention patients	Number of control patients
Renner, 2012 6442a	Romieu 2007	granulocyte colony-stimulating factors: pegfilgrastim, 6 mg/day; d2 sc	No treatment in cycle 1	Germany, Spain, Italy, France	16	Follow-up duration	65 to 77	Range	0	3	30	29
	Vogel 2005	granulocyte colony-stimulating factors: pegfilgrastim, 6 mg/day; d2 sc	identical placebo	Europe and the USA	Unclear	Unclear	21 to 88 (mean 52)	Range	5	42	463	465
	Hansen 1995	granulocyte-macrophage colony-stimulating factors: molgramostim, 5 μg/kg/dday; d2-11 sc	No treatment	Denmark	Unclear	Unclear	37 to 61	Range	0	1	11	9
	Jones 1996	granulocyte-macrophage colony-stimulating factors: sargramostim, 250 μg/m2; d3-15 sc	Identical placebo	USA	21.5	Follow-up duration	25 to 69 (mean 47)	Range	1	4	70	72
Review author, year, estimate ID	Trials	Intervention: specific drugs and dosing	Comparator: specific drugs and dosing	Country	Length of follow-up (months)	Follow-up measure	Age	Age measure	Number of intervention group events	Number of control group events	Number of intervention patients	Number of control patients

Rowe, 2008 6677	Fiel 1983	Intravenous methylprednisolone 4 mg per kilogram and tapering schedule of oral methylprednisolone starting at the dosage of 32 mg BID and decreasing to 0 mg over 8 days	Placebo group received identical placebo injection and oral tablets	USA	0.32	Follow-up duration	15 to 45	Range	3	9	34	42
	Lee 1993a	Intramuscular injection: 10 mg dexamethasone by intramuscular injection	Placebo intramuscular injection	Unclear	0.23	Follow-up duration	37.923	Mean	1	1	36	16
	McNamara 1993	Intramuscular injection of 240 mg methylprednisolone	Saline placebo	Unclear	0.23	Follow-up duration	18 and 45	Range	1	4	30	26
	Shapiro 1983	Methylprednisolone 32 mg by mouth for 8 days	Placebo tablets	Unclear	0.46	Follow-up duration	Unclear	Unclear	0	0	11	15
Review author, year, estimate ID	Trials	Intervention: specific drugs and dosing	Comparator: specific drugs and dosing	Country	Length of follow-up (months)	Follow-up measure	Age	Age measure	Number of intervention group events	Number of control group events	Number of intervention patients	Number of control patients
Salpeter, 2006 6797a	Brusasco 2003	Tiotropium 18mcg daily	Placebo	Unclear	6	Follow-up duration	64.199	Mean	12	20	402	400
	Casaburi 2002	Tiotropium 18mcg daily	Placebo	Unclear	12	Follow-up duration	65	Mean	30	35	550	371
	Niewoehner 2005	Tiotropium 18mcg daily	Placebo	Unclear	6	Follow-up duration	67.85	Mean	64	87	914	915
Review author, year, estimate ID	Trials	Intervention: specific drugs and dosing	Comparator: specific drugs and dosing	Country	Length of follow-up (months)	Follow-up measure	Age	Age measure	Number of intervention group events	Number of control group events	Number of intervention patients	Number of control patients
Salpeter, 2006 6797b	Brusasco 2003	Tiotropium 18mcg daily	Placebo	Unclear	6	Follow-up duration	64.199	Mean	12	20	402	400
	Casaburi 2002	Tiotropium 18mcg daily	Placebo	Unclear	12	Follow-up duration	65	Mean	30	35	550	371
	Niewoehner 2005	Tiotropium 18mcg daily	Placebo	Unclear	6	Follow-up duration	67.85	Mean	64	87	914	915
	Lung health study	Ipratropium 36mcg three times a day	Placebo	Unclear	60	Follow-up duration	48.5	Mean	136	108	1961	1962

Review author, year, estimate ID	Trials	Intervention: specific drugs and dosing	Comparator: specific drugs and dosing	Country	Length of follow-up (months)	Follow-up measure	Age	Age measure	Number of intervention group events	Number of control group events	Number of intervention patients	Number of control patients
Salpeter, 2006 6797d	Brusasco 2003	Salmeterol 50 mcg twice daily	Tiotropium 18mcg daily	Unclear	6	Follow-up duration	63.951	Mean	20	12	405	402
	Friedman 1999	Albuterol 240 mcg twice daily	Ipratropium 42 mcg daily	Unclear	3	Follow-up duration	64.294	Mean	11	3	437	362
Review author, year, estimate ID	Trials	Intervention: specific drugs and dosing	Comparator: specific drugs and dosing	Country	Length of follow-up (months)	Follow-up measure	Age	Age measure	Number of intervention group events	Number of control group events	Number of intervention patients	Number of control patients
Cheyne, 2015 8744d	Voshaar 2008	tiotropium 5mcg delivered via SPIRIVA Respimat SMI once daily and placebo delivered via pMDI four times daily; tiotropium 10 mcg delivered via SPIRIVA Respimat SMI once daily and placebo delivered via pMDI four times daily	ipratropium bromide 36 mcg, delivered via pMDI four times daily and 2 inhalations of placebo Respimat	Germany, Italy, South Africa and Switzerland, USA, and Canada	3	Follow-up duration	64.331	Mean	1	1	180	89
	Vincken 2002	iotropium 18 mcg once daily (Spiriva HandiHaler taken between 8 am and 10 am) and ipratropium matched placebo four times daily.	tiotropium matched placebo once daily and ipratropium 40 mcg four times daily	Netherlands and Belgium	12	Follow-up duration	63.901	Mean	26	21	356	179
	Voshaar 2008	tiotropium5mcg delivered via SPIRIVA Respimat SMI once daily and placebo delivered via pMDI four times daily; tiotropium 10 mcg delivered via SPIRIVA Respimat SMI once daily and placebo delivered via pMDI four times daily	ipratropium bromide 36 mcg, delivered via pMDI four times daily and 2 inhalations of placebo Respimat	Germany, Italy, South Africa and Switzerland, USA, and Canada	3	Follow-up duration	64.331	Mean	1	2	180	89
Review author, year, estimate ID	Trials	Intervention: specific drugs and dosing	Comparator: specific drugs and dosing	Country	Length of follow-up (months)	Follow-up measure	Age	Age measure	Number of intervention group events	Number of control group events	Number of intervention patients	Number of control patients

Fisher, 2014 8769b	Honold 2012	Bone marrow stem cells. Type of stem cells: endothelial progenitor cells from bone marrow aspirates. Summary of stem cell isolation and type and route of delivery: G-CSF was administered to the participants for 5 days. 270 ml of peripheral blood was drawn. Mononuclear cells were isolated using a Ficoll gradient centrifugation and cells were resuspended in X-vivo 15 medium with 1 ng/ml carrier-free human recombinant VEGF, atorvastin and 20% human serum drawn from each individual participant. Cells were cultured ex vivo for 4 days to enrich in endothelial progenitor cells (uptake of LDL). Dose of stem cells: 29 ± 12 x 10 . Timing of stem cell procedure: % days following G-SCF administration and 4 days following bone marrow aspiration and cell culture G-CSF details: Yes, 5 days prior to cell isolation.	No placebo	Germany	60	Follow-up duration	55.036	Mean	0	2	22	10

	Losordo 2011	Intervention arm: Low Dose (LD) and High Dose (HD) of CD34+ cells. Type of stem cells: CD34+ cells from mobilised peripheral blood. Summary of stem cell isolation and type and route of delivery: G-CSF was given to all participants at 5 µg/kg for 4 - 5 days. On day 5 leukapheresis was performed. The following day mononuclear cells were collected and CD34+ cells enriched using a commercially available device (Isolex 300im) magnetic cell separation system. Cell suspension with > 70% viability and > 50% CD34+ cells were given at 2 doses of body weight with a maximum of 100 kg. Cell suspension was diluted in saline (0.9%NaCl) with 5%autologous plasma. Cells were injected into the myocardium. The injection was performed by NOGA mapping and at 10 sites (0.2 cc/ site) using a NOGA Myostar catheter. Dose of stem cells: 1 x 10 CD34 cells/kg and 5 x 10 CD 34 cells/kg. Timing of stem cell procedure: At least 3 months following MI. G-CSF details: G-CSF was given to all participants at 5 µg/kg for 4 - 5 days	Placebo. G-CSF was given to all participants at 5 µg/kg for 4 - 5 days. No cells were injected, only saline (0.9 % NaCl) with 5% autologous plasma	USA	12	Follow-up duration	60.8	Mean	3	4	111	55
Review author, year, estimate ID	Trials	Intervention: specific drugs and dosing	Comparator: specific drugs and dosing	Country	Length of follow-up (months)	Follow-up measure	Age	Age measure	Number of intervention group events	Number of control group events	Number of intervention patients	Number of control patients

Hemkens, 2016 8784a	Morgan 2005	Colchicine Dose: 2 x 0.6 mg/day for at least 24 months, some up to 72 months	Placebo at least 24 months, some up to 72 months	USA	72	Follow-up duration	55.551	Mean	166	191	274	275
	Antoniou 2006	Colchicine dose: 1 mg/day plus prednisolone 10 mg/day	Interferon-c 1b 200 mg 3 x / week subcutaneously plus prednisolone 10 mg/d interferon gamma-1b	Greece	25	Follow-up duration	67.08	Mean	4	8	18	32
Review author, year, estimate ID	Trials	Intervention: specific drugs and dosing	Comparator: specific drugs and dosing	Country	Length of follow-up (months)	Follow-up measure	Age	Age measure	Number of intervention group events	Number of control group events	Number of intervention patients	Number of control patients
Yohannes, 2011 8511a	Brusasco 2003	Tiotropium 18 ug daily	Placebo	Unclear	6.04	Follow-up duration	64.2	Mean	12	20	402	400
	Casaburi 2002	Tiotropium 18 ug daily	Placebo	Unclear	12	Follow-up duration	65	Mean	30	35	533	371
	Chan 2007	Tiotropium 18 ug daily	Placebo	Unclear	12	Follow-up duration	66.9	Mean	51	25	608	305
	dusser 2006	Tiotropium 18 ug daily	Placebo	Unclear	11.2	Follow-up duration	64.8	Mean	28	33	500	510
	niewoehner 2005	Tiotropium 18 ug daily	Placebo	Unclear	6.04	Follow-up duration	67.9	Mean	64	87	914	915
	tashkin 2008	Tiotropium 18 ug daily	Placebo	Unclear	48.4	Follow-up duration	64.5	Mean	759	811	2968	3006
	vogelmier 2006	Tiotropium 18 ug and placebo twice daily	Placebo twice daily	Unclear	6.04	Follow-up duration	62.6	Mean	5	3	209	203
Review author, year, estimate ID	Trials	Intervention: specific drugs and dosing	Comparator: specific drugs and dosing	Country	Length of follow-up (months)	Follow-up measure	Age	Age measure	Number of intervention group events	Number of control group events	Number of intervention patients	Number of control patients
Zhang, 2011 8604b	Bielecka A 2009	Atorvastatin 10–40 mg/day	Placebo or blank control	Unclear	6	Follow-up duration	57	Mean	8	5	41	27
	CORONA 2007	Rosuvastatin 10 mg/day	Placebo or blank control	Unclear	32.8	Median	73	Mean	622	669	2514	2497
	GISSI-HF 2008	Rosuvastatin 10 mg/day	Placebo or blank control	Unclear	46.8	Median	68	Mean	629	634	2285	2289
	Hamaad 2005	Atorvastatin 40 mg/day	Placebo or blank control	Unclear	3	Follow-up duration	67	Mean	0	1	13	10
	Krum H 2007	Rosuvastatin 10 mg/day	Placebo or blank control	Unclear	5.5	Follow-up duration	61	Mean	0	3	40	45

	Mozaffarian D 2005	Atorvastatin 10 mg/day	Placebo or blank control	Unclear	2	Follow-up duration	51	Mean	0	0	12	10
	Node K 2003	Simvastatin 5–10 mg/day	Placebo or blank control	Unclear	3.5	Follow-up duration	54	Mean	1	1	24	27
	Sola S 2006	Atorvastatin 20 mg/day	Placebo or blank control	Unclear	12	Follow-up duration	53	Mean	8	13	54	54
	Wojnicz R 2006	Atorvastatin 40 mg/day	Placebo or blank control	Unclear	6	Follow-up duration	38	Mean	0	2	36	38
	Xie RQ 2008	Atorvastatin 10 mg/day	Placebo or blank control	Unclear	12	Follow-up duration	NA		6	18	40	41
	Yamada T 2007	Atorvastatin 10 mg/day	Placebo or blank control	Unclear	31	Follow-up duration	64	Mean	2	6	19	19
Review author, year, estimate ID	Trials	Intervention: specific drugs and dosing	Comparator: specific drugs and dosing	Country	Length of follow-up (months)	Follow-up measure	Age	Age measure	Number of intervention group events	Number of control group events	Number of intervention patients	Number of control patients
Zhang, 2011 8604f	Bielecka-Dąbrowa A et al. 2009	Atorvastatin 10–40 mg/day	Placebo or blank control	Unclear	6	Follow-up duration	57	Mean	8	5	41	27
	Hamaad et al. 2005	Atorvastatin 40 mg/day	Placebo or blank control	Unclear	3	Follow-up duration	67	Mean	0	1	13	10
	Mozaffarian D et al. 2005	Atorvastatin 10 mg/day	Placebo or blank control	Unclear	2	Follow-up duration	51	Mean	0	0	12	10
	Sola S et al. 2006	Atorvastatin 20 mg/day	Placebo or blank control	Unclear	12	Follow-up duration	53	Mean	8	13	54	54
	Wojnicz R et al. 2006	Atorvastatin 40 mg/day	Placebo or blank control	Unclear	6	Follow-up duration	38	Mean	0	2	36	38
	Xie RQ et al. 2008	Atorvastatin 10 mg/day	Placebo or blank control	Unclear	12	Follow-up duration	NA		6	18	40	41
	Yamada T et al. 2007	Atorvastatin 10 mg/day	Placebo or blank control	Unclear	31	Follow-up duration	64	Mean	2	6	19	19
Review author, year, estimate ID	Trials	Intervention: specific drugs and dosing	Comparator: specific drugs and dosing	Country	Length of follow-up (months)	Follow-up measure	Age	Age measure	Number of intervention group events	Number of control group events	Number of intervention patients	Number of control patients
Chen, 2015 8742a	RAAM-PEF 2011	Eplerenone: 25 (titrated to 50)mg/day	Placebo	USA	7	Follow-up duration	Unclear	Unclear	1	2	23	23
	TOPCAT 2014	Spironolactone: 15–45 mg/day	Placebo	Northa America, South America, Central America, Russia, Georgia	39.6	Follow-up duration	Unclear	Unclear	206	245	1722	1723

	Kurrelmeyer 2014	Spironolactone: 25 mg/day	Placebo	USA	6	Follow-up duration	Unclear	Unclear	0	0	24	24
	REMINDER 2014	Eplerenone: 25 (titrated to 50) mg/day	Placebo	Europe	10.5	Follow-up duration	Unclear	Unclear	7	11	506	506
Review author, year, estimate ID	Trials	Intervention: specific drugs and dosing	Comparator: specific drugs and dosing	Country	Length of follow-up (months)	Follow-up measure	Age	Age measure	Number of intervention group events	Number of control group events	Number of intervention patients	Number of control patients
Coeytaux, 2014 8747a	Rubin 2002	Bosentan	Placebo or standard therapy	Unclear	2 to 4 months of treatment	Range	Unclear	Unclear	6	9	144	69
	Galie 2008	Ambrisentan	Placebo or standard therapy	Unclear			Unclear	Unclear	4	2	134	67
	Galie 2008b	Ambrisentan	Placebo or standard therapy	Unclear			Unclear	Unclear	5	9	127	65
Review author, year, estimate ID	Trials	Intervention: specific drugs and dosing	Comparator: specific drugs and dosing	Country	Length of follow-up (months)	Follow-up measure	Age	Age measure	Number of intervention group events	Number of control group events	Number of intervention patients	Number of control patients
Coeytaux, 2014 8747b	Galie 2005	Sildenafil	Placebo or standard therapy	Unclear	2 to 4 months of treatment	Range	Unclear	Unclear	6	7	207	70
	Simonneau 2008	Sildenafil	Placebo or standard therapy	Unclear			Unclear	Unclear	8	11	134	131
	Galie 2009	Tadalafil	Placebo or standard therapy	Unclear			Unclear	Unclear	6	2	323	82
	jing 2012	Vardanafil	Placebo or standard therapy	Unclear			Unclear	Unclear	1	2	44	20
Review author, year, estimate ID	Trials	Intervention: specific drugs and dosing	Comparator: specific drugs and dosing	Country	Length of follow-up (months)	Follow-up measure	Age	Age measure	Number of intervention group events	Number of control group events	Number of intervention patients	Number of control patients
Fisher, 2015 8770b	assmus 2013	Intracoronary bone marrow mononuclear cells 123 (69)×10^6 cells and 150 (77)×10^6 cells and Shock-wave	Shock-wave	Unclear	4	Follow-up duration	Unclear	Unclear	9	20	43	39
	bolli 2011	Intracoronary cardiac stem cells 5-10×10^5 cells	Control (no mock infusion or placebo)	Unclear	12	Follow-up duration	Unclear	Unclear	1	0	16	7

	heldman 2014a	Intramuscular bone marrow–derived mesenchymal stem cells (dose not reported)	Placebo	Unclear	12	Follow-up duration	Unclear	Unclear	0	1	19	10
	heldman2014b	Intramuscular bone marrow mononuclear cells (dose not reported)	Placebo	Unclear	12	Follow-up duration	Unclear	Unclear	0	0	19	11
	honold 2012	Bone marrow–derived endothelial progenitor cells 20 (12)×10^6 cells	Control (no placebo)	Unclear	60	Follow-up duration	Unclear	Unclear	0	2	22	10
	menasche 2008	Intramuscular skeletal myoblast Low Dose: 400 x10^6 cells; High Dose: 800 x10^6 cells	Placebo	Unclear	72	Mean	Unclear	Unclear	1	2	4	3
	patila 2014	Bone marrow mononuclear cells Median 8.4×10^8 cells	Placebo	Unclear	12	Follow-up duration	Unclear	Unclear	0	0	20	19

Review author, year, estimate ID	Trials	Intervention: specific drugs and dosing	Comparator: specific drugs and dosing	Country	Length of follow-up (months)	Follow-up measure	Age	Age measure	Number of intervention group events	Number of control group events	Number of intervention patients	Number of control patients
Jankowska, 2016 8790a	toblli 2007	Intravenous iron sucrose 200 mg	Saline	Unclear	5	Follow-up duration	75	Mean	0	5	20	20
	okonko 2008	Intravenous iron sucrose 200 mg weekly until ferritin 500 microgam/L or more	Saline	Unclear	4.2	Follow-up duration	63.371	Mean	1	2	24	11
	anker 2009	Intravenous ferric carboxymaltose 200 mg weekly until repletion dose achived	Saline	Unclear	6 to 6.5	Follow-up duration	67.662	Mean	7	9	305	154
	ponikowski 2015	Intravenous ferric carboxymaltose 500 mg or 1000 mg at baseline and week 6. Maintenance phase 500 mg at weeks 12, 24, 36.	Saline	Unclear	13	Follow-up duration	69.502	Mean	10	32	150	151

Review author, year, estimate ID	Trials	Intervention: specific drugs and dosing	Comparator: specific drugs and dosing	Country	Length of follow-up (months)	Follow-up measure	Age	Age measure	Number of intervention group events	Number of control group events	Number of intervention patients	Number of control patients
Kumar, 2014 8815a	Ashworth 1998	Propofol	desflurane	Unclear	Unclear	Unclear	44	Mean	0	0	30	30
	Jakobssen 1997	Propofol	desflurane	Unclear	Unclear	Unclear	35	Mean	0	0	40	40

	Nathan 1998	Propofol	sevoflurane	Unclear	Unclear	Unclear	Unclear		1	6	26	26
	Raeder 1997	Propofol	sevoflurane	Unclear	Unclear	Unclear	33	Mean	0	0	85	84
	Raeder 1998	Propofol	desflurane	Unclear	Unclear	Unclear	Unclear		0	0	30	30
	Rapp 1992	Propofol	desflurane	Unclear	Unclear	Unclear	34	Mean	0	4	23	68
	Smith 1999	Propofol	sevoflurane	Unclear	Unclear	Unclear	39	Mean	0	1	72	139
	Tan 2010	Propofol	sevoflurane	Unclear	Unclear	Unclear	34	Mean	1	4	40	40
	White 2007	Propofol	sevoflurane	Unclear	Unclear	Unclear	38	Mean	3	6	55	67
Review author, year, estimate ID	Trials	Intervention: specific drugs and dosing	Comparator: specific drugs and dosing	Country	Length of follow-up (months)	Follow-up measure	Age	Age measure	Number of intervention group events	Number of control group events	Number of intervention patients	Number of control patients
Le, 2016 8817a	Boccanelli 2009	Canrenone 25 mg (titrated to 50 mg/ day)	Placebo	Italy	12	Mean	62.502	Mean	37	43	218	227
	chan 2007	Spironolactone 25 mg/day	Placebo	China	12	Mean	63.275	Mean	2	4	23	25
	cicoira 2002	Spironolactone 25 mg (titrated to 50 mg/day)	Routine treatment	Italy	12	Mean	62.108	Mean	1	2	54	52
	deswal 2011	Eplerenone 25 mg (titrated to 50 mg/ day)	Placebo	USA	6	Mean	70.45	Mean	3	6	21	23
	edelmann 2013	Spironolactone 25 mg/day	Placebo	Germany and Austria	12	Mean	67	Mean	60	50	213	209
	gao 2007	Spironolactone 20 mg/day	Placebo	China	6	Mean	54.5	Mean	2	4	58	58
	pitt 2014	Spironolactone (15 to 45 mg/day)	Placebo	International	39.6	Mean	68.7	median	766	792	1722	1723
	pitt 1999	Spironolactone 25 mg (titrated to 50 mg/day)	Placebo	International	24	Mean	65	Mean	614	700	822	841
	zannad 2011	Eplerenone 25 mg (titrated to 50 mg/ day)	Placebo	International	21	Mean	68.65	Mean	408	491	1364	1373
	modena 2001	Potassium canrenoate 50 mg/ day	Placebo	Italy	12	Mean	60.435	Mean	0	4	24	22

	pitt 2003	Eplerenone 25 mg (titrated to 50 mg/ day)	Placebo	International	16	Mean	64	Mean	1493	1526	3319	3313
	uzunhasan 2009	Spironolactone 50 mg/day	Placebo	Turkey	6	Mean	52	Mean	0	1	41	41
Review author, year, estimate ID	Trials	Intervention: specific drugs and dosing	Comparator: specific drugs and dosing	Country	Length of follow-up (months)	Follow-up measure	Age	Age measure	Number of intervention group events	Number of control group events	Number of intervention patients	Number of control patients
Le, 2016 8817d	Boccanelli 2009	Canrenone 25 mg (titrated to 50 mg/ day)	Placebo	Italy	12	Mean	62.502	Mean	14	29	218	227
	Chan 2007	Spironolactone 25 mg/day + candesartan	Placebo + candesartan	China	12	Mean	63.275	Mean	2	4	23	25
	cicoira 2002	Spironolactone 25 mg (titrated to 50 mg/day)	Routine treatment	Italy	12	Mean	62.108	Mean	1	2	54	52
	deswal 2011	Eplerenone 25 mg (titrated to 50 mg/ day)	Placebo	USA	6	Mean	70.45	Mean	1	2	21	23
	edelmann 2013	Spironolactone 25 mg/day	Placebo	Germany and Austria	12	Mean	67	Mean	21	15	213	209
	pitt 2014	Spironolactone (15 to 45 mg/day)	Placebo	International	39.6	Mean	68.7	Median	206	245	1722	1723
	pitt 1999	Spironolactone 25 mg (titrated to 50 mg/day)	Placebo	International	24	Mean	65	Mean	377	448	822	841
	vizzardi 2013	Spironolactone 25 mg (titrated to 100 mg/day)	Placebo	Italy	44	Mean	63	Mean	6	24	65	65
	zannad 2011	Eplerenone 25 mg (titrated to 50 mg/ day)	Placebo	International	21	Mean	68.65	Mean	304	399	1364	1373
	modena 2001	Potassium canrenoate 50 mg/ day	Placebo	Italy	12	Mean	60.435	Mean	0	4	24	22
	montalescot 2014	Eplerenone 25 mg (titrated to 50 mg/ day)	Placebo	International	10.5	Mean	58.15	Mean	7	11	506	506
	pitt 2003	Eplerenone 25 mg (titrated to 50 mg/ day)	Placebo	International	16	Mean	64	Mean	606	649	3319	3313

Review author, year, estimate ID	Trials	Intervention: specific drugs and dosing	Comparator: specific drugs and dosing	Country	Length of follow-up (months)	Follow-up measure	Age	Age measure	Number of intervention group events	Number of control group events	Number of intervention patients	Number of control patients
Le, 2016 8817e	Boccanelli 2009	Canrenone 25 mg (titrated to 50 mg/ day)	Placebo	Italy	12	Mean	62.502	Mean	14	29	218	227
	Chan 2007	Spironolactone 25 mg/day + candesartan	Placebo + candesartan	China	12	Mean	63.275	Mean	2	4	23	25
	cicoira 2002	Spironolactone 25 mg (titrated to 50 mg/day)	Routine treatment	Italy	12	Mean	62.108	Mean	1	2	54	52
	deswal 2011	Eplerenone 25 mg (titrated to 50 mg/ day)	Placebo	USA	6	Mean	70.45	Mean	1	2	21	23
	edelmann 2013	Spironolactone 25 mg/day	Placebo	Germany and Austria	12	Mean	67	Mean	21	15	213	209
	pitt 2014	Spironolactone (15 to 45 mg/day)	Placebo	International	39.6	Mean	68.7	Median	206	245	1722	1723
	pitt 1999	Spironolactone 25 mg (titrated to 50 mg/day)	Placebo	International	24	Mean	65	Mean	377	448	822	841
	vizzardi 2013	Spironolactone 25 mg (titrated to 100 mg/day)	Placebo	Italy	44	Mean	63	Mean	6	24	65	65
	zannad 2011	Eplerenone 25 mg (titrated to 50 mg/ day)	Placebo	International	21	Mean	68.65	Mean	304	399	1364	1373

Review author, year, estimate ID	Trials	Intervention: specific drugs and dosing	Comparator: specific drugs and dosing	Country	Length of follow-up (months)	Follow-up measure	Age	Age measure	Number of intervention group events	Number of control group events	Number of intervention patients	Number of control patients
Liu, 2014 8826a	Abulhul 2012	Atorvastatin 40 mg/day	no statin	Unclear	6	Mean	72	Mean	1	1	28	28
	Bielecka 2009	atorvastatin 10-40 mg/day	no statin	Unclear	6	Mean	57	Mean	0	1	41	27
	Hamaad 2005	Atorvastatin 40 mg/day	placebo	Unclear	3	Mean	67	Mean	0	1	12	10
	Node 2003	simvastatin 5-10 mg/day	placebo	Unclear	3.5	Mean	54	Mean	1	1	23	27
	Sola 2006	atorvastatin 20 mg/day	placebo	Unclear	12	Mean	53	Mean	8	13	46	43
	Takano 2013	pitavastatin 2 mg/day	no statin	Unclear	35.5	Mean	62	Mean	39	47	288	286

	Wojnicz 2006	atorvastatin 40 mg/day	no statin	Unclear	6	Mean	38	Mean	0	2	34	37
	Xie 2010	atorvastatin 10-20 mg/day	no statin	Unclear	12	Mean	NA	Mean	10	18	78	41
	Yamade 2007	atorvastatin 10 mg/day	placebo	Unclear	31	Mean	64	Mean	2	6	19	19
Review author, year, estimate ID	Trials	Intervention: specific drugs and dosing	Comparator: specific drugs and dosing	Country	Length of follow-up (months)	Follow-up measure	Age	Age measure	Number of intervention group events	Number of control group events	Number of intervention patients	Number of control patients
Lopez, 2015 8829a	sandborn 2009	Anti-tumor necrosis factor	Placebo	Unclear	Unclear	Unclear	Unclear	Unclear	64	46	484	244
	feagan 2013	Anti-tumor necrosis factor	Placebo	Unclear	Unclear	Unclear	Unclear	Unclear	53	75	480	483
Review author, year, estimate ID	Trials	Intervention: specific drugs and dosing	Comparator: specific drugs and dosing	Country	Length of follow-up (months)	Follow-up measure	Age	Age measure	Number of intervention group events	Number of control group events	Number of intervention patients	Number of control patients
Ni, 2014 8850a	Jones 2011 (ACCLAIM/COPD I)	aclidinium 200 µg once daily via the Genuair inhaler	placebo	16 European countries (one in Andorra, five in Austria, four in Belgium, 10 in Bulgaria, eight in the Czech Republic, three in Denmark, nine in France, 10 in Germany, eight in Hungary, six in Italy, four in Netherlands, nine in Poland, nine in Romania, 25 in Russia, eight in Spain, 13 in the UK)	13	Follow-up duration	62.422	Mean	40	14	616	210

	Jones 2011 (ACCLAIM/COPD II)	aclidinium 200 µg once daily via the Genuair inhaler	placebo	Seven countries (72 sites in the United States, 13 sites in Argentina, 13 sites in Australia, seven sites in Canada, two sites in Mexico, three sites in New Zealand and nine sites in South Africa)	13	Follow-up duration	65.125	Mean	36	23	594	201
	Chanez 2010	inhaled aclidinium25µg, 50µg, 100µg, 200µg, or 400µg (viamultidose dry-powder inhaler, Genuair)	placebo	Europe and Russia	4	Follow-up duration	61.407	Mean	2	0	335	64
	Maltais 2011	inhaled aclidinium 200 µg once-daily via a multidose dry powder inhaler	placebo	United States and Canada	6	Follow-up duration	64.84	Mean	1	3	86	95
	Kerwin 2012 (ACCORD COPD I)	inhaled aclidinium 200 µg twice daily, inhaled aclidinium 400 µg twice daily at the same time in the morning (between 8:00 and 10:00 AM) and evening (between 8:00 and 10:00 PM) via a multiple-dose dry powder inhaler (Genuair)	placebo	United States and Canada	14	Follow-up duration	64.372	Mean	3	1	374	185
	Rennard 2013 (ACCORD COPD II)	inhaled aclidinium 200 µg twice daily, inhaled aclidinium 400 µg twice daily via a multiple-dose dry powder inhaler (Genuair/Pressair)	Placebo	United States and Canada	14	Follow-up duration	62.763	Mean	6	5	359	182

	Singh 2014 (ACLIFORM)	inhaled aclidinium/formoterol fixed dose combination (FDC) high dose twice daily, inhaled aclidinium/formoterol FDC low dose twice daily, inhaled aclidinium 400 µg twice daily	Placebo	22 countries (two sites in Austria, two in Belgium, five in Bulgaria, two in Croatia, 12 in Czech Republic, four in Denmark, five in Finland, seven in France, 28 in Germany, 15 in Hungary, four in Italy, eight in Repulbic of Korea, seven in Netherlands, 21 in Poland, 12 in Romania, five in Russia, seven in Slovakia, nine in South Africa, seven in Spain, five in Sweden, 14 in Ukraine, 16 in the United Kingdom)	24	Follow-up duration	63.469	Mean	6	5	385	194
	Jones 2012 (ATTAIN)	inhaled aclidinium 200 µg twice daily or 400 µg twice daily via amultipledose dry powder inhaler (Genuair)	Placebo	11 countries (10 sites in the Czech Republic, five in France, 17 in Germany, 13 in Hungary, three in Italy, one in Peru, 21 in Poland, 10 in the Russian Federation, five in Spain, 13 in South Africa and five in the Ukraine)	24	Follow-up duration	72.856	Mean	5	10	546	273
	D’Urzo 2013 (AUGMENT COPD)	inhaled fixed dose combination of aclidinium 400 µg plus formoterol 6 µg or 12 µg twice daily, inhaled aclidinium 400 µg twice daily	Placebo	193 sites in the United States, 11 in Australia, 10 in Canada and eight in New Zealand	24	Follow-up duration	63.953	Mean	7	6	337	332

	Beier 2013	aclidinium bromide 400 µg twice daily in the morning (9:00 ± 1 hour) and evening (21:00 ± 1 hour) via the Genuair/Pressair multidose dry powder inhaler, tiotropium 18 µg once daily in the morning (9:00 ± 1 hour) via the HandiHaler	Placebo	Three sites in the Czech Republic, 23 in Germany, eight in Hungary and 15 in Poland	6	Follow-up duration	61.933	Mean	0	0	171	85
Review author, year, estimate ID	Trials	Intervention: specific drugs and dosing	Comparator: specific drugs and dosing	Country	Length of follow-up (months)	Follow-up measure	Age	Age measure	Number of intervention group events	Number of control group events	Number of intervention patients	Number of control patients
Saab, 2015 8884a	Agrawal 2012	Probiotic: VSL#3 (4 strains of Lactobacilli, 3 strains of Bifidobacteria, 1 strain Streptococcus thermophilus)	No treatment, lactulose	India	12	Follow-up duration	16 or older	Cut-off	21	28	64	65
	Dhiman 2014	Probiotic: VSL#3 (4 strains of Lactobacilli, 3 strains of Bifidobacteria, 1 strain Streptococcus thermophilus)	Placebo	India	6	Follow-up duration	16 or older	Cut-off	16	29	66	64
	Mittal 2011	Probiotic: 110 billion CFU	No treatment, lactulose	India	3	Follow-up duration	16 or older	Cut-off	1	2	40	40
	Pereg 2011	Probiotic: Lactobacillus acidophilus, Lactobacillus bulgaricus, Bifidobacterium bifidum	placebo:	Unclear	6	Follow-up duration	16 or older	Cut-off	3	3	18	18
Review author, year, estimate ID	Trials	Intervention: specific drugs and dosing	Comparator: specific drugs and dosing	Country	Length of follow-up (months)	Follow-up measure	Age	Age measure	Number of intervention group events	Number of control group events	Number of intervention patients	Number of control patients
Poole, 2015 8867	Decramer 2005	N-acetylcysteine 600 mg daily	Placebo	Unclear	36	Follow-up duration	62	Mean	55	69	256	267
	Moretti 2004	Erdosteine 300 mg twice daily	Placebo	Unclear	8	Follow-up duration	67	Mean	10	19	79	76
	Zheng 2014	Carbocysteine 1500 mg daily (2 × 250 mg TDS)	Placebo	Unclear	12	Follow-up duration	65	Mean	33	36	495	495
	Tse 2013	N-acetylcysteine 600 mg twice daily	Placebo	Unclear	12	Follow-up duration	71	Mean	26	45	58	63

Review author, year, estimate ID	Trials	Intervention: specific drugs and dosing	Comparator: specific drugs and dosing	Country	Length of follow-up (months)	Follow-up measure	Age	Age measure	Number of intervention group events	Number of control group events	Number of intervention patients	Number of control patients
Xie, 2016 8916a	AIRE study investigators 1993 (AIRE)	Ramipril (5 to 10 mg/day)	Placebo	Worldwide	15	Mean	65	Mean	143	178	1004	982
	Pfeffer 1992 (SAVE)	Captopril (6.25-150 mg/day)	Placebo	USA and Canada	42	Mean	59	Mean	154	192	1115	1116
	SOLVD investigators 1992 (SOLVD prevention)	Enalapril (2.5-20 mg/day)	Placebo	Worldwide	37	Mean	59	Mean	184	273	2111	2117
	SOLVD investigators 1991 (SOLVD treatment)	Enalapril (2.5-20 mg/day)	Placebo	Worldwide	41	Mean	61	Mean	332	470	1285	1284
	Kober 1995 (TRACE)	Trandolapril (1-4 mg/day)	Placebo	Denmark	26	Mean	68	Mean	125	171	876	873
Review author, year, estimate ID	Trials	Intervention: specific drugs and dosing	Comparator: specific drugs and dosing	Country	Length of follow-up (months)	Follow-up measure	Age	Age measure	Number of intervention group events	Number of control group events	Number of intervention patients	Number of control patients
Xie, 2016 8916b	Matsumori 2003 (ARCH J)	Candesartan cilexetil (8 mg/day)	Placebo	Japan	6	Mean	64	Mean	8	17	148	144
	McMurray 2003 (CHARM ADDED)	Candesartan (4-32 mg/day)	Placebo	Worldwide	41	Median	64	Mean	309	356	1276	1272
	Granger 2003 (CHARM ALTERNATIVE)	Candesartan (4-32 mg/day)	Placebo	Worldwide	34	Median	67	Mean	207	286	1013	1015
	Cohn 2001 (VALHEFT)	Valsartan (80-320 mg/day)	Placebo	Worldwide	23	Mean	63	Mean	346	455	2511	2499
Review author, year, estimate ID	Trials	Intervention: specific drugs and dosing	Comparator: specific drugs and dosing	Country	Length of follow-up (months)	Follow-up measure	Age	Age measure	Number of intervention group events	Number of control group events	Number of intervention patients	Number of control patients
Xie, 2016 8916c	Boccanelli 2009 (AREA IN CHF)	Canrenone (25-50 mg/day)	Placebo	Italy	12	Mean	63	Mean	6	17	218	227

	Zannad 2011 (EMPHASIS HF)	Eplerenone (25-50 mg/day)	Placebo	Worldwide	21	Median	69	Mean	164	253	1364	1373
	Pitt 2003 (EPHESUS)	Eplerenone (25-50 mg/day)	Placebo	Worldwide	16	Median	64	Mean	345	391	3319	3313
	Pitt 1999 (RALES)	Spironolactone (25 mg/day)	Placebo	Worldwide	24	Mean	65	Mean	215	300	822	841

Review author, year, estimate ID	Trials	Intervention: specific drugs and dosing	Comparator: specific drugs and dosing	Country	Length of follow-up (months)	Follow-up measure	Age	Age measure	Number of intervention group events	Number of control group events	Number of intervention patients	Number of control patients
Yang, 2015 8918n	Bosquet Sanz 2006	Single drug: One dose of tobramicin 100 mg im and another one 8 h afterwards	One dose of tobramicin 100 mg im and oral ciprofloxacin 500 mg, one dose 30 min before biopsy and afterwards the ciprofloxacin 12/12h 3d	Spain	0.0986842	Follow-up duration	Unclear	Unclear	15	3	71	86
	Chan 2012	Single drug: 1 g amoxicillin-clavulanate 2 h before and 12/12h 1d after prostate biopsy	Combined drugs: amoxicillin-clavulanate, ciprofloxacin 250 mg 2 h before and 12/12h 1d after prostate biopsy	China	1.2828947	Follow-up duration	Unclear	Unclear	4	0	70	65
	Pace 2012	Single drug: orally ciprofloxacin 1,000 mg every 24 h starting the evening before biopsy until 4 d after	Combined drugs: ciprofloxacin 1,000 mg every 24 h starting the evening before biopsy until 4 d after and a dose of ceftriaxone 1 g was injected locally	Italy	0.1973684	Follow-up duration	Unclear	Unclear	4	1	179	188

Review author, year, estimate ID	Trials	Intervention: specific drugs and dosing	Comparator: specific drugs and dosing	Country	Length of follow-up (months)	Follow-up measure	Age	Age measure	Number of intervention group events	Number of control group events	Number of intervention patients	Number of control patients
Zhang, 2016 8922l	Cleland 2006	Perindopril	Placebo	Unclear	26.2	Mean	75	Mean	64	73	424	426
	Deswal 2011	Eplerenone	Placebo	Unclear	6	Mean	70.37	Mean	1	2	21	23
	Massie 2008	Irbesartan	Placebo	Unclear	49.5	Mean	72	Mean	325	336	2067	2061
	Pitt 2014	Spironolactone	Placebo	Unclear	39.6	Mean	68.7	Mean	206	245	1722	1723

	Yip 2008a	Irbesartan + diuretics	Diuretics	Unclear	12	Mean	74.06	Mean	6	6	56	50
	Yip 2008b	Ramipril + diuretics	Diuretics	Unclear	12	Mean	73.47	Mean	5	6	45	50
	Yusuf 2003	Candesartan	Placebo	Unclear	8.5	Mean	67.15	Mean	241	276	1514	1509
	Zi 2003	Quinapril	Placebo	Unclear	6	Mean	78	Mean	2	5	36	38
Review author, year, estimate ID	Trials	Intervention: specific drugs and dosing	Comparator: specific drugs and dosing	Country	Length of follow-up (months)	Follow-up measure	Age	Age measure	Number of intervention group events	Number of control group events	Number of intervention patients	Number of control patients
Zhou, 2014 8928	Rosano 2003	Trimetazidine	Placebo	Unclear	6	Follow-up duration	65.5	Mean	0	1	16	16
	Vitale 2004	Trimetazidine	Placebo	Unclear	6	Follow-up duration	77.511	Mean	1	4	23	24
	Fragasso 2006	Trimetazidine	Placebo	Unclear	12	Follow-up duration	64.982	Mean	6	11	29	29
	Tuunanen 2008	Trimetazidine	Placebo	Unclear	3	Follow-up duration	58.263	Mean	0	1	12	7
Review author, year, estimate ID	Trials	Intervention: specific drugs and dosing	Comparator: specific drugs and dosing	Country	Length of follow-up (months)	Follow-up measure	Age	Age measure	Number of intervention group events	Number of control group events	Number of intervention patients	Number of control patients
Ziff, 2015 8931a	Digitalis Investigation Group 1997 (DIG trial)	Digoxin	Placebo	Unclear	37.2	Follow-up duration	63.5	Mean	2184	2282	3397	3403
	Ahmed 2006	Digoxin	Placebo	Unclear	37.2	Follow-up duration	67	Mean	332	330	492	496
Review author, year, estimate ID	Trials	Intervention: specific drugs and dosing	Comparator: specific drugs and dosing	Country	Length of follow-up (months)	Follow-up measure	Age	Age measure	Number of intervention group events	Number of control group events	Number of intervention patients	Number of control patients
Zou, 2016 8933a	Jones 2011 (ACCLAIM/COPD 1)	Aclidinium 200 µg once daily	Placebo	Unclear	12	Follow-up duration	62.415	Mean	40	14	616	210
	Jones 2011 (ACCLAIM/COPD 2)	Aclidinium 200 µg once daily	Placebo	Unclear	12	Follow-up duration	65.124	Mean	36	23	594	201
	Kerwin 2012 (ACCORD COPD 1)	AC: 200 µg twice daily (400 µg total)	Placebo	Unclear	2.7906977	Follow-up duration	64.356	Mean	3	1	374	185

	Rennard 2013 (ACCORD COPD 2)	AC: 200 µg twice daily (400 µg total)	Placebo	Unclear	2.7906977	Follow-up duration	62.744	Mean	6	5	359	182
	Singh 2014 (ACLIFORM)	AC: 400 µg twice daily	Placebo	Unclear	5.5813953	Follow-up duration	63.485	Mean	6	5	385	194
	Jones 2012 (ATTAIN)	AC: 200 µg twice daily 400 µg	Placebo	Unclear	5.5813953	Follow-up duration	62.349	Mean	5	10	546	273
	D'Urzo 2014 (AUGMENT COPD)	AC: 400 µg twice daily	Placebo	Unclear	5.5813953	Follow-up duration	63.966	Mean	7	6	337	332
Review author, year, estimate ID	Trials	Intervention: specific drugs and dosing	Comparator: specific drugs and dosing	Country	Length of follow-up (months)	Follow-up measure	Age	Age measure	Number of intervention group events	Number of control group events	Number of intervention patients	Number of control patients
La Mantia, 2010 4260	Johnson 1995	Subcutaneous GA 20 mg daily	Subcutaneous placebo self-administered daily	USA	36	Follow-up duration	34.45	Mean	14	20	125	125
	Comi 2001	Subcutaneous GA 20 mg daily	Placebo	Europe and Canada	9	Follow-up duration	34.05	Mean	16	30	119	120
Review author, year, estimate ID	Trials	Intervention: specific drugs and dosing	Comparator: specific drugs and dosing	Country	Length of follow-up (months)	Follow-up measure	Age	Age measure	Number of intervention group events	Number of control group events	Number of intervention patients	Number of control patients
Nannini, 2013 5533g	Doherty 2012	Mometasone furoate/formoterol 200/10 mcg twice daily	Placebo	North, Central and South America, Europe, Africa and Asia	12	Follow-up duration	59.3	Mean	4	12	239	236
	Tashkin 2012	Mometasone furoate/formoterol 200/10 mcg twice daily	Placebo	South America, Asia, Africa, Europe and North America	12	Follow-up duration	63.3	Mean	3	7	207	212
Review author, year, estimate ID	Trials	Intervention: specific drugs and dosing	Comparator: specific drugs and dosing	Country	Length of follow-up (months)	Follow-up measure	Age	Age measure	Number of intervention group events	Number of control group events	Number of intervention patients	Number of control patients
Su, 2014 7504	Amit 2006	60 ug of nitroprusside diluted in saline solution as a 5-mL bolus	Identical volume of saline solution	Unclear	Unclear	Unclear	62	Mean	5	10	48	50
	Pan 2009	100 ug Nitroprusside (diluted to 20 ug/ ml)	100 ug Nitroglycerin	Unclear	Unclear	Unclear	53	Mean	4	11	46	46

Review author, year, estimate ID	Trials	Intervention: specific drugs and dosing	Comparator: specific drugs and dosing	Country	Length of follow-up (months)	Follow-up measure	Age	Age measure	Number of intervention group events	Number of control group events	Number of intervention patients	Number of control patients
Karner, 2014 8692e	Bateman 2010a	tiotropium 5 mcg (two puffs of 2.5 mcg each) once daily in the morning	placebo	31 countries	12	Follow-up duration	65	Mean	161	198	1989	2002
	Bateman 2010b	1. Orally inhaled tiotropium 5 mcg, 2 actuations of 2.5 mcg tiotropium once daily in the morning 2. Orally inhaled tiotropium 10 mcg, 2 actuations of 5 mcg tiotropium once daily in the morning	placebo	Unclear	12	Follow-up duration	65	Mean	78	44	1337	653
	Voshaar 2008	1. Tiotropium 5 mcg once daily 2. Tiotropium 10 mcg once daily	placebo	Germany, Italy, South Africa, Switzerland, USA, and Canada	3	Follow-up duration	64	Mean	2	0	360	181
Review author, year, estimate ID	Trials	Intervention: specific drugs and dosing	Comparator: specific drugs and dosing	Country	Length of follow-up (months)	Follow-up measure	Age	Age measure	Number of intervention group events	Number of control group events	Number of intervention patients	Number of control patients
Jaeschke, 2008 3639f	Pauwels	Formoterol and inhaled corticosteroid	inhaled corticosteroid same dase	Unclear	Unclear	Unclear	42.254	Mean	3	8	426	427
	van der molen	Formoterol and inhaled corticosteroid	inhaled corticosteroid same dase	Unclear	Unclear	Unclear	42.385	Mean	0	3	125	114
	obyrne	Formoterol and inhaled corticosteroid	inhaled corticosteroid same dase	Unclear	Unclear	Unclear	35.408	Mean	7	10	869	862
	chuchalin	Formoterol and inhaled corticosteroid	inhaled corticosteroid same dase	Unclear	Unclear	Unclear	45.52	Mean	0	1	111	114
	price	Formoterol and inhaled corticosteroid	inhaled corticosteroid same dase	Unclear	Unclear	Unclear	37.505	Mean	0	1	250	255
	buhl	Formoterol and inhaled corticosteroid	inhaled corticosteroid same dase	Unclear	Unclear	Unclear	43.99	Mean	1	0	352	171
	morice	Formoterol and inhaled corticosteroid	inhaled corticosteroid same dase	Unclear	Unclear	Unclear	39.68	Mean	0	1	462	217
	kuna	Formoterol and inhaled corticosteroid	inhaled corticosteroid same dase	Unclear	Unclear	Unclear	44.992	Mean	1	1	409	207

Review author, year, estimate ID	Trials	Intervention: specific drugs and dosing	Comparator: specific drugs and dosing	Country	Length of follow-up (months)	Follow-up measure	Age	Age measure	Number of intervention group events	Number of control group events	Number of intervention patients	Number of control patients
Jong, 2002 3781d	McKelvie 1999 (RESOLVD)	Candesartan 4 mg; 8mg; 16mg once daily and Enalapril 10 mg twice a day	Enalapril 10 mg twice a day	Unclear	10.75	Mean	Unclear	Unclear	31	7	332	109
	Tonkon 2000	Irbesartan 150 mg once daily and an ACE	Placebo and ACE	Unclear	3	Mean	Unclear	Unclear	2	1	57	52
	Cohn 2001 (Val-HeFT)	Valsartan 160 mg twice a day and an ACE	Placebo and ACE	Unclear	23	Mean	Unclear	Unclear	346	455	2511	2499
Review author, year, estimate ID	Trials	Intervention: specific drugs and dosing	Comparator: specific drugs and dosing	Country	Length of follow-up (months)	Follow-up measure	Age	Age measure	Number of intervention group events	Number of control group events	Number of intervention patients	Number of control patients
Edmonds, 2012 2159	Afilalo 1999	Beclomethasone dipropionate by metered dose inhaler 1 mg at 0, 30 min, 1 h, 2 h, 4 h (total 5 mg)	placebo metered dose inhaler at the same time intervals	Canada	Unclear	Unclear	Unclear	Unclear	2	5	28	26
	Guttman 1997	Beclomethasone dipropionate 1 mg by metered dose inhaler plus spacer at 0, 0.5, 1, 2, 4, 6, 8 (total 7 mg)	placebo metered dose inhaler at the same time intervals	Canada	Unclear	Unclear	Unclear	Unclear	8	12	30	30
	Rodrigo 1998	Flunisolide, 1 mg every 10 min by metered dose inhaler with spacer for 3 h (total dose of flunisolide 18 mg)	placebo metered dose inhaler at the same time intervals	Uruguay	Unclear	Unclear	32.45	Mean	4	12	47	47
	Rodrigo 2003	Fluticasone 1000 µg, salmetrol 400 µg, and ipratropium 84 µg every 10 min by metered dose inhaler with spacer for 3 h.	placebo metered dose inhaler at the same time intervals	Uruguay	Unclear	Unclear	33.845	Mean	6	15	58	62
	Starobin 2008	Patients (Flixotide group) received inhalation of FlixotideNebules®(fluticasone propionate) (GlaxoWellcome, Australia) 2 mL (0.5 mg).	placebo metered dose inhaler at the same time intervals	Australia	Unclear	Unclear	47.637	Mean	5	11	26	23
Review author, year, estimate ID	Trials	Intervention: specific drugs and dosing	Comparator: specific drugs and dosing	Country	Length of follow-up (months)	Follow-up measure	Age	Age measure	Number of intervention group events	Number of control group events	Number of intervention patients	Number of control patients

Cawood, 2012 1264	Gariballa et al., 2006	400 ml of supplement; 2.48kcal/ml density; 995 kcal 49.75 g P (calculated) 20 percent energy from protein (comm with author)	Placebo	Unclear	6	Follow-up duration	77	Mean	65	89	222	223
	Norman et al. 2008	Fresubin Protein Energy Drink, Fresenius; 1.5 kcal/ml supplement density; prescribed 900 kcal 60 g protein 27 percent energy from protein	Dietary Counselling	Unclear	3	Follow-up duration	53	Mean	10	20	38	42

Review author, year, estimate ID	Trials	Intervention: specific drugs and dosing	Comparator: specific drugs and dosing	Country	Length of follow-up (months)	Follow-up measure	Age	Age measure	Number of intervention group events	Number of control group events	Number of intervention patients	Number of control patients
Baigent, 2013 1653a	184 trials	Coxib	Placebo	Unclear	Unclear	Unclear	Unclear	Unclear				

Review author, year, estimate ID	Trials	Intervention: specific drugs and dosing	Comparator: specific drugs and dosing	Country	Length of follow-up (months)	Follow-up measure	Age	Age measure	Number of intervention group events	Number of control group events	Number of intervention patients	Number of control patients
Bonsu, 2016 8729a	Bielecka,2009	Atorvastatin 40 mg	Placebo	Unclear	6	Follow-up duration	57	Mean	5	8	41	27
	Hamaad, 2005	Atorvastatin 40 mg	Placebo	Unclear	3	Follow-up duration	67		0	1	13	10
	Node, 2003	Simvastatin 5–10 mg	Placebo	Unclear	3.5	Follow-up duration	54		1	1	24	27
	Sola, 2006	Atorvastatin 20 mg	Placebo	Unclear	12	Follow-up duration	54		8	13	54	54
	Takano, 2013	Pitavastatin 2 mg	Placebo	Unclear	35.5	Follow-up duration	63		39	47	288	286
	Vrtotec, 2008	Atorvastatin 10 mg	Placebo	Unclear	12	Follow-up duration	62		8	20	55	55
	Wojnicz, 2005	Atorvastatin 40 mg	Placebo	Unclear	6	Follow-up duration	38		0	2	36	38
	Xie,2008	Atorvastatin 10–20 mg	Placebo	Unclear	12	Follow-up duration	NR		10	18	78	41
	Yamada,2007	Atorvastatin 10 mg	Placebo	Unclear	31	Follow-up duration	64		2	6	19	19

Review author, year, estimate ID	Trials	Intervention: specific drugs and dosing	Comparator: specific drugs and dosing	Country	Length of follow-up (months)	Follow-up measure	Age	Age measure	Number of intervention group events	Number of control group events	Number of intervention patients	Number of control patients
Kuenzli, 2010 4206c	RESOLVD 1999	Candesartan 16mg once daily and Enalapril 10mg twice	Enalapril 10mg twice daily	Unclear	9.6	Follow-up duration	63.753	Mean	31	7	332	109
	CHARM added 2003	Candesartan 32mg once daily and ACE inhibitors	No recommended fixed dose, but investigators were advised of the doses of ACE inhibitors known to reduce morbidity and mortality in patients with congestive heart failure	Unclear	40.8	Follow-up duration	64	Mean	323	382	1276	1272
	Kum 2008	Irbesartan 300mg and an ACE inhibitor	Not specified	Unclear	12	Follow-up duration	67.5	Mean	4	7	25	25
	ValHeFT 2001	Valsartan 160mg twice daily and an ACE inhibitor	Not specified	Unclear	24	Follow-up duration	62.499	Mean	346	455	2511	2499
Review author, year, estimate ID	Trials	Intervention: specific drugs and dosing	Comparator: specific drugs and dosing	Country	Length of follow-up (months)	Follow-up measure	Age	Age measure	Number of intervention group events	Number of control group events	Number of intervention patients	Number of control patients
Yang, 2015, 8918a	Isen 1999a	Antibiotic (Ofloxacin 400 mg orally single dose) or Antibiotic (trimethoprim/sulfonamide methoxazole 160 mg/800 mg orally single dose)	Placebo	Turkey	Unclear	Unclear	Unclear	Unclear	0	3	42	23
	Kapoor 1998	Antibiotic (ciprofloxacin 500 mg orally single dose)	Placebo	USA	Unclear	Unclear	Unclear	Unclear	1	4	257	260
	Isen 1999b	ATB (ofloxacin 400 mg orally single dose) or ATB (trimethoprim/sulfonamide methoxazole 160 mg/800 mg orally single dose)	Placebo	Turkey	Unclear	Unclear	Unclear	Unclear	0	3	45	23

Table S9. Details of GRADE ratings

Estimate ID	GRADE provided in review	GRADE rating	Risk of bias	Inconsistency	Indirectness	Imprecision	Publication bias
100c	No	Moderate	0	0	0	0	0
593	Yes	Very low	0	0	0	0	0
1021	No	Low	0	0	0	0	0
1164	Yes	Very low	0	0	0	0	0
1264	No	Low	0	0	0	0	0
1438a	Yes	Moderate	0	0	0	0	0
1606a	No	Low	0	0	0	0	0
1629a	No	Moderate	0	0	0	0	0
1653a	No	Moderate	0	0	0	0	0
1752a	No	Moderate	0	0	0	0	0
2159	Yes	Moderate	0	0	0	0	0
2599a	No	Moderate	0	0	0	0	0
2640b	No	Low	0	0	0	0	0
2933a	Yes	Moderate	0	0	0	0	0
3275d	Yes	Low	0	0	0	0	0
3275e	Yes	Very low	0	0	0	0	0
3275l	Yes	Very low	0	0	0	0	0
3415	No	High	0	0	0	0	0
3639f	No	Low	0	0	0	0	0
3640b	No	Low	0	0	0	0	0
3640d	No	Low	0	0	0	0	0
3657	No	Moderate	0	0	0	0	0
3781d	No	Moderate	0	0	0	0	0
4002h	No	High	0	0	0	0	0
4003a	Yes	Moderate	0	0	0	0	0
4003e	No	Low	0	0	0	0	0
4004o	No	Low	0	0	0	0	0
4004r	No	Low	0	0	0	0	0
4004y	No	Low	0	0	0	0	0
4004z	No	High	0	0	0	0	0
4065a	No	Moderate	0	0	0	0	0
4067b	No	Low	0	0	0	0	0
4123b	No	Moderate	0	0	0	0	0
4123i	No	Moderate	0	0	0	0	0
4206a	No	Moderate	0	0	0	0	0
4206c	No	Moderate	0	0	0	0	0
4260	No	Moderate	0	0	0	0	0
4508a	Yes	High	0	0	0	0	0
4615d	No	Low	0	0	0	0	0
4615e	No	Very Low	0	0	0	0	0
4629a	No	Moderate	0	0	0	0	0
4812a	No	Low	0	0	0	0	0
4838a	No	Low	0	0	0	0	0
4838b	No	Moderate	0	0	0	0	0
4844	No	Moderate	0	0	0	0	0
5371	No	Very Low	0	0	0	0	0
5533g	No	Low	0	0	0	0	0
5606	No	Moderate	0	0	0	0	0
6442a	Yes	Low	0	0	0	0	0
6564a	No	Low	0	0	0	0	0
6568a	No	Moderate	0	0	0	0	0
6670	No	Low	0	0	0	0	0
6677	No	Low	0	0	0	0	0

6746b	No	High	0	0	0	0	0
6797a	No	Low	0	0	0	0	0
6797b	No	Low	0	0	0	0	0
6797d	No	Low	0	0	0	0	0
6806f	No	Low	0	0	0	0	0
7029b	No	Moderate	0	0	0	0	0
7484a	No	Moderate	0	0	0	0	0
7504	No	Low	0	0	0	0	0
8511a	No	Low	0	0	0	0	0
8604b	No	Low	0	0	0	0	0
8604f	No	Low	0	0	0	0	0
8692e	No	Low	0	0	-1	0	0
8729a	No	High	0	0	0	0	0
8742a	No	Low	0	0	0	0	0
8744d	No	Moderate	0	0	0	0	0
8747a	No	Low	0	0	0	0	0
8747b	No	Low	0	0	0	0	0
8761a	Yes	High	0	0	0	0	0
8769b	Yes	Low	0	0	0	0	0
8770b	No	Low	0	0	0	0	0
8784a	No	Low	0	0	0	0	0
8790a	No	Moderate	0	0	0	0	0
8815a	No	Low	0	0	0	0	0
8817a	No	Moderate	0	0	0	0	0
8817d	No	Low	0	0	0	0	0
8817e	No	Low	0	0	0	0	0
8826a	No	Moderate	0	0	0	0	0
8829a	No	Moderate	0	0	0	0	0
8850a	Yes	High	0	0	0	0	0
8867	Yes	Moderate	0	0	0	0	0
8884a	No	Low	0	0	-1	0	0
8916a	No	Moderate	0	0	0	0	0
8916b	No	Moderate	0	0	0	0	0
8916c	No	Moderate	0	0	0	0	0
8918a	No	Moderate	0	0	0	0	0
8918n	No	Low	0	0	0	0	0
8922l	No	Low	0	0	0	0	0
8928	No	Very Low	0	0	0	0	0
8931a	No	High	0	0	0	-1	0
8933a	No	Moderate	0	0	0	0	0

Additional table 9 cont. Risk of bias: domain 1 for downgrading

Estimate ID	Risk of bias	Justification for rating	**Decision:** *not serious, serious, very serious*	Risk of bias notes
100c	unclear	Information on sequence generation and allocation concealment not reported but 75% of trials double blind, all were intention treat, and follow-up was near 100% for all trials.	Serious	
593	unclear	Random sequence unclear 66%; allocation conealment unclear 100%; selective reporting unclear 66%, high risk 33%; high risk other bias 100%	Serious	
1021	unclear	The authors stated that "The overall quality of the trials was high; each followed a double-blinded protocol, and only one study had possible irregularities in the randomization process. Follow-up of randomly assigned patients was almost complete. The only minor methodologic flaw was lack of description of the randomization process in 18 of the 22 trials." It is unclear if allocation concealment was adequate in any of the studies and there was no information on selective reporting.	Serious	
1164	high	Unclear risk for random sequence 3/4; allocation concealment 4/4; blinding 2/4; other bias 1/4. High risk blinding 2/4	very serious	
1264	unclear	Sequence generation, allocation concealment, completeness of reporting, and selective reporting unclear in all trials.	Serious	
1438a	low	Unclear risk incomplete outcome data 2/4; High risk blinding participants/personnel 1/4	not serious	
1606a	low	No blinded outcome assessment in one of the two included trials but this is less important for the outcome of hospitalisatiosn and the trial carried little weight in the meta-analysis.	not serious	
1629a	unclear	All elements unclear for all trials.	Serious	
1653a	unclear	The authors reported that studies were only eligible if they used a randomisation method with robust allocation concealment. They also report that "most events occurred in a small number of recent trials that used secure randomisation methods and treatment blinding, sensitivity analyses (available on request) indicated that our results were not materially influenced by uncertainties about the quality of older trials. However, they provide little to no actual information on the risk of bias in included studies.	not serious	
1752a	low	Authors assessed "trials for the adequacy of allocation concealment, blindness of patients and physicians to the treatment, and blind assessment of the outcome of interest". All included studies received 5/5. Authors used completeness of follow-up as an inclusion criteria although "adequate" follow-up not defined.	not serious	
2159	unclear	High risk: random sequence 1/5, blinding (ALL) 1/5; Unclear risk: random sequence 1/5, incomplete outcome data 5/5; selective reporting 5/5	not serious	
2599a	unclear	Newcastle Ottawa scale used but only summary scores were provided as opposed to individual criteria attainment by trial. Only blinding information was available to assess risk of bias. Other source for potential bias is that all included studies came from the same research group, within eight years, and all showed significant reductions in admissions.	serious	
2640b	low	Detailed information relevant for risk of bias assessment were provided.	not serious	
2933a	low	Three studies all described in detail central randomisation with adequate allocation concealment. Only one study (Wiktor 1999) had a small number of exclusions following randomisation (7participants out of a total of 771).	not serious	
3275d	unclear	unclear risk: for random sequence 3/3, allocation concealment 2/3 and high risk for other biases 3/3; however CHARM-Alternative 2003 contribute 90% = unclear risk for random sequence only but high risk for other biases	serious	
3275e	unclear	unclear risk: for random sequence 3/4, allocation concealment 1/4; and high risk for other biases 4/4. Selection bias identified by review authors	serious	
3275l	unclear	All studies unclear risk for random sequence; 3/4 for allocatin concealment; 1/4 unclear for blinding; 1/4 high risk incomplete outcome data and 4/4 high risk other bias	serious	
3415	low	Sequence generation unclear in all trials but groups were even at baseline and all other domains were low risk of bias.	not serious	
3639f	unclear	Sequence generation, follow-up completeness, and selective reporting unclear in 100% of trials.	serious	
3640b	low	Inclusion criteria ensured all included trials had double-blinding or patients and care-givers; adequate follow-up completion and outcome reporting. Authors report that all studies had adeaqaute allocation concealment, outcome assessment was blinded, and all were sponsored by pharma.	not serious	

3640d	low	Inclusion criteria ensured all included trials had double-blinding or patients and care-givers; adequate follow-up completion and outcome reporting. Authors report that all studies had adeaqaute allocation concealment, outcome assessment was blinded, and all were sponsored by pharma.	not serious	
3657	unclear	No blinding in 1 of the 2 included trials although low risk of bias otherwise.	serious	
3781d	unclear	Sequence generation, concealment, and selective reporting unclear in all trials.	serious	
4002h	low		not serious	
4003a				
4003e	unclear	Allocation concealment unclear in 83% of trials.	serious	
4004o	high	Allocation concealment unclear in both studies. High risk of bias for incomplete outcome reporting in one trial: high rate of drop outs. Both industry funded.	serious	
4004r	high	43% of trials had high drop out rates relative to event rates. All industry funded. 71% of trials had unclear allocation concealment methods.	serious	
4004z	high	All trials industry funded. Allocation concealment unclear in 62% of trials although these trials received little weight in the meta-analysis.	not serious	Not downgraded because bias that may affect the outcome occurred in studies that represent a small weight in the overall analysis and the effect estimates span both directions (reductions/increases in admissions).
4004y	unclear	Allocation concealment unclear in 80% of trials.	serious	
4065a	unclear	Sequence generation, allocation concealment, incomplete outcome assessment, and selective reporting unclear in all trials. 50% of trials were unblinded. Funding unclear for all studies.	serious	
4067b	unclear	Sequence generation, allocation concealment, completeness of outcome assessement, and reporting bias unclear in all trials.	serious	
4123b	high	Very high attrition in both trials (greater than 75%)	serious	
4123i	high	Very high attrition in both trials (greater than 75%)	serious	
4206a	unclear	No blinding in 33% of studies. Sequence generation unclear in all trials.	serious	
4206c	unclear	Sequence generation unclear in all trials. Selective reporting unclear in all trials.	serious	
4260	low		not serious	
4508a				
4615d	high	Sequence generation unclear in 50% of trials. Allocation concealment inadequate in 40% of trials. No blinding in 50% of trials. Completeness of outcome data and selective reporting unclear in all trials.	serious	
4615e	high	Sequence generation unclear in 50% of trials. Allocation concealment high risk of bias in 50% of trials. No blinding in 67% of trials. Completeness of outcome data and selective reporting unclear in all trials.	very serious	
4629a	unclear	Allocation concealment and selective reporting unclear in all trials. All other elements unclearin 50% of the trials.	serious	
4812a	unclear	Allocation concealment unclear in 33% of studies. Blinding unclear in 66% of studies.	serious	
4838a	low	Authors used Cochrane criteria to asses risk of bias. All included trials had adequate sequence generation, allocation concealment, and had blinding of patients, providers, and outcome assessors. Incomplete outcome data and selective reporting results not reported although all trials deemed low risk by the authors.	not serious	"The criteria used for quality assessment were sequence generation of allocation; allocation concealment; masking of participants, staff, and outcome assessors; incomplete outcome data; selective outcome reporting; and other sources of bias, as recommended by the Cochrane Collaboration. We classed studies with high or unclear risk of bias for any of the first three components to be of low quality."
4838b	low	Authors used Cochrane criteria to asses risk of bias. All included trials had adequate sequence generation, allocation concealment, and had blinding of patients, providers, and outcome assessors. Incomplete outcome data and selective reporting results not reported although all trials deemed low risk by the authors.	not serious	"The criteria used for quality assessment were sequence generation of allocation; allocation concealment; masking of participants, staff, and outcome assessors; incomplete outcome data; selective outcome reporting; and other sources of bias, as recommended by the Cochrane Collaboration. We classed

				studies with high or unclear risk of bias for any of the first three components to be of low quality."
4844	high	No blinding in 33% of trials. One trial used date of birth for "randomisation". Allocation concealment in the other 66% of trials.	serious	
5371	unclear	JADAD score used. Neither trial was double blinded (unclear if single blinded) and allocation concealment unclear in both. All other criteria relevant for risk of bias unclear.	serious	
5533g	high	Large drop out rates in 50% of trials. Sequence generation, concealment, completeness of follow-up, and selective reporting unclear in 50% of trials	serious	
5606	low	Sequence generation and allocation concealment unclear in 44% of studies. No blinding in 33% of studies. Selective reporting bias in 33% of studies. However, studies with low risk of bias had the majority of weighting in meta-analysis.	not serious	
6442a				
6564a	unclear	Quality scores given but no descriptive information on criteria to assess risk of bias.	serious	
6568a	unclear	Allocation concealment and selective reporting unclear for all trials. Sequence generation and completeness of follow-up unclear in 80% of trials.	serious	
6670	unclear	Allocation concealment and selective reporting unclear in all trials.	serious	
6677	unclear	Allocation concealment, completeness of follow-up, and selective reporting unclear in all trials.	serious	
6746b	low		not serious	
6797a	unclear	Sequence generation, allocation concealment, and selective reporting unclear in all trials.	serious	
6797b	unclear	Sequence generation, allocation concealment, and selective reporting unclear in all trials.	serious	
6797d	unclear	Sequence generation, allocation concealment, and selective reporting unclear in all trials.	serious	
6806f	high	High risk of selective reporting bias in 83% of trials. Allocation concealment unclear in 83% of trials and high risk of bias in remaining 17% of trials. High risk of bias regarding blinding in 50% of trials.	very serious	
7029b	unclear	Allocation concealment and selective reporting unclear in all trials.	serious	
7484a	high	No blinding in 83% of studies. Information on selective reporting unclear in all trials.	serious	
7504	unclear	Allocation concealment, completeness of follow-up, and selective reporting unclear in 100% of trials	serious	
8511a	unclear	Risk of bias for sequence generation, allocation concealment, completeness of follow-up, selective reporting unclear in all but one study.	serious	
8604b	high	73% of studies did not have adequate allocation concealment. Random sequence generation was unclear in 64% of studies. No blinding in 36% of studies. 45% of studies did not account for withdrawals and did not use intention to treat analysis.	serious	
8604f	high	86% of studies did not have adequate allocation concealment. No blinding in 43% of studies. Reasons for withdrawals not reported in 57% of studies.	very serious	
8692e	low		not serious	
8729a	low		not serious	
8742a	unclear	Allocation concealment, completeness of follow-up, and selective reporting unclear in all trials.	serious	
8744d	low		not serious	
8747a	unclear	Domains unclear for all trials.	serious	
8747b	unclear	Domains unclear for all trials.	serious	
8769b				
8770b	high	29% of studies had inadequate sequence generation, allocation concealment, and blinding. One study had high risk of bias for incomplete reporting.	serious	
8784a	high	No blinding in 50% of studies. High loss to follow up in 50% of studies.	serious	
8790a	low		not serious	
8815a	high	No blinding in any of the included trials. Allocation concealment unclear in 56% of trials. Adequate sequence generation unclear in 66% of trials.	serious	
8817a	low		not serious	

8817d	low		not serious	
8817e	low		not serious	
8826a	unclear	Allocation concealment unclear in all trials. Adequacy of sequence generation unclear in 88% of studies. Blinding unclear in 33% of studies.	serious	
8829a	unclear	Adequacy of sequence generation, allocation concealment, completeness of follow-up, and selective reporting unclear in both trials.	serious	
8850a				
8867				
8884a	unclear	No blinding in 50% of trials. All elements unclear for other domains.	serious	
8916a	unclear	Allocation concealment unclear in all trials.	serious	
8916b	unclear	Sequence generation and allocation concealment unclear 50% of trials. All other elements low risk of bias.	serious	
8916c	unclear	Sequence generation and allocation concealment unclear 75% of trials. All other elements low risk of bias.	serious	
8918n	high	No blinding in 66% of studies. Allocation concealment unclear in 66% of studies.	serious	
8918a	low		not serious	
8922l	unclear	Allocation concealment unclear in all studies. Sequence generation unclear in 43% of studies. Completeness of follow-up and selective reporting unclear in all studies.	serious	
8928	unclear	Sequence generation, allocation concealment, completeness of follow-up, selective reporting unclear for all studies.	serious	
8931a	low		not serious	
8933a	low		not serious	

Table S9 cont. Inconsistency: domain 2 for downgrading

Estimate ID	Large variations in the size of effect	Overlap of confidence intervals	Statistical significance of heterogeneity (p<0.1)	**I-squared:** small (0-39%) medium (40-74%) large (≥75%)	**Decision:** *not serious, serious, very serious*	Inconsistency notes
100c	no	yes	no	small	not serious	
593	yes	yes	no	small	not serious	
1021	yes	no	unclear	unclear	serious	
1164	yes	no	yes	medium	serious	
1264	no	unclear	no	small	not serious	
1438a	yes	yes	no	small	not serious	
1606a	yes	no	yes	large	serious	
1629a	no	yes	no	small	not serious	
1653a	unclear	unclear (forrest plot not provided)	unclear	unclear	serious	
1752a	no	yes	no	unclear	not serious	
2159	no	yes	no	small	not serious	
2599a	yes	yes	no	medium	not serious	
2640b	unclear	unclear	unclear	unclear	serious	
2933a	yes	yes	no	small	not serious	
3275d	no	yes	no	small	not serious	
3275e	yes	yes	no	small	not serious	
3275l	yes	yes	no	small	not serious	
3415	no	yes	no	small	not serious	
3639f	no	yes	no	small	not serious	
3640b	unclear	unclear	no	unclear	not serious	
3640d	unclear	unclear	no	unclear	not serious	
3657	unclear	unclear	no	unclear	not serious	
3781d	no	yes	no	unclear	not serious	
4002h	no	yes	no	small	not serious	
4003a						
4003e	no	yes	no	small	not serious	
4004o	no	yes	no	small	not serious	
4004r	yes	yes	no	small	not serious	
4004y	yes	yes	no	small	not serious	
4004z	yes	yes	no	small	not serious	
4065a	no	yes	no	small	not serious	
4067b	no	yes	no	small	not serious	
4123b	no	yes	no	small	not serious	
4123i	no	yes	no	small	not serious	
4206a	no	yes	yes	medium	not serious	
4206c	unclear	0	no	small	not serious	
4260	no	yes	no	small	not serious	
4508a						

4615d	yes	no	yes	medium	serious	
4615e	no	yes	no	small	not serious	
4629a	no	yes	no	small	not serious	
4812a	no	no	yes	large	serious	
4838a	no	yes	yes	medium	serious	
4838b	no	yes	yes	medium	serious	
4844	no	no	no	small	not serious	
5371	unclear	unclear	unclear	unclear	serious	
5533g	no	yes	no	small	not serious	
5606	yes	yes	no	small	not serious	
6442a						
6564a	yes	yes	no	unclear	not serious	
6568a	yes	yes	no	small	not serious	
6670	unclear	unclear	unclear	unclear	serious	
6677	no	yes	no	small	not serious	
6746b	unclear	unclear	no	unclear	not serious	
6797a	no	yes	no	small	not serious	
6797b	unclear	unclear	unclear	unclear	serious	
6797d	no	yes	no	small	not serious	
6806f	yes	yes	no	small	not serious	
7029b	unclear	unclear	no	small	not serious	
7484a	no	yes	unclear	unclear	not serious	
7504	no	yes	no	small	not serious	
8511a	yes	yes	yes	small	not serious	
8604b	yes	no	no	small	not serious	
8604f	unclear	unclear	no	small	not serious	
8692e	no	no	no	small	not serious	
8729a	yes	yes	no	small	not serious	
8742a	no	yes	no	small	not serious	
8744d	no	yes	no	small	not serious	
8747a	yes	yes	no	small	not serious	
8747b	no	yes	no	small	not serious	
8769b						
8770b	yes	yes	no	small	not serious	
8784a	no	yes	no	small	not serious	
8790a	no	yes	no	small	not serious	
8815a	no	yes	no	small	not serious	
8817a	yes	yes	no	small	not serious	
8817d	yes	no	yes	medium	serious	
8817e	yes	no	yes	medium	serious	
8826a	yes	yes	no	small	not serious	
8829a	no	yes	no	small	not serious	
8850a						
8867						
8884a	no	yes	no	small	not serious	

8916a	no	yes	no	small	not serious	
8916b	no	yes	no	small	not serious	
8916c	no	yes	no	small	not serious	
8918a	no	yes	no	small	not serious	
8918n	unclear	unclear	no	small	not serious	
8922l	unclear	unclear	no	small	not serious	
8928	no	yes	no	small	not serious	
8931a	no	yes	unclear	unclear	not serious	
8933a	yes	yes	no	small	not serious	

Table S9 cont. Indirectness: domain 3 for downgrading.

Estimate ID	Direct comparison (A vs. B, not network analysis)	Patients	Interventions	Comparator	Outcome	Majority OECD countries	**Decision:** *no indirectness, serious indirectness, very serious indirections*	Indirectness notes
100c	yes	no (previous use of statins varied from 0% to 76% between trials)	yes	yes	yes	Yes	no indirectness	
593	yes	yes	No, different interventions drugs	yes	yes	Yes	no indirectness	
1021	yes	yes	yes	yes	yes	Yes	no indirectness	
1164	yes	yes	No, different drugs/doses/number of interventions used across studies	No, different comparators/doses used	yes	Yes	no indirectness	
1264	yes	yes	yes	yes	Yes	Yes	no indirectness	
1438a	yes	yes	yes	No, different comparator drugs/doses	yes	Yes	no indirectness	
1606a	yes	yes	no (one rhythm control drug used in one study and a variety of drugs used in the other trial)	yes	no (one rate control drug used in one trial and a variety of drugs used in the other trial)	Yes	serious indirectness	
1629a	yes	yes	yes	yes	yes	Yes	no indirectness	
1653a	yes	yes	yes	yes	yes	Yes	no indirectness	
1752a	yes	yes	yes	yes	yes	Yes	no indirectness	
2159	yes	yes	no, different interventions across studies	no, different comparators across studies	yes	Yes	no indirectness	
2599a	yes	yes	yes	yes	yes	Yes	no indirectness	
2640b	yes	yes	yes	yes	yes	Yes	no indirectness	
2933a	yes	yes	No, 2/3 same dose, 1/3 half-dose	yes	yes	Yes	no indirectness	

3275d	yes	no, different severties of CHF	no, different doses used	yes	no, review does not state outcome was assessed (SPICE 2000). However, only contributes 4.6%% to overall pooled estimate	Yes	no indirectness	
3275e	yes	no, different severties of CHF	no, different doses used	yes	no, review does not state outcome was assessed (SPICE 2000). However, only contributes 0.4% to overall pooled estimate	Yes	no indirectness	
3275l	yes	no, different severties of CHF	no, different doses in all 3 arms	unclear: insufficient info on ACEI therapy regimen or placebo used. Placebo used alongside ACEi in some studies but not others	yes	Yes	serious indirectness	
3415	yes	yes	yes	yes	yes	Yes	no indirectness	
3639f	yes	yes	yes	yes	yes	Yes	no indirectness	
3640b	yes	yes	yes	yes	yes	Yes	no indirectness	
3640d	yes	yes	yes	yes	yes	Yes	no indirectness	
3657	yes	yes	yes	yes	yes	Yes	no indirectness	
3781d	yes	yes	yes	yes	yes	Yes	no indirectness	
4002h	yes	yes	yes	yes	yes	Yes	no indirectness	
4003a								
4003e	yes	yes	yes	yes	yes	Yes	no indirectness	
4004o	yes	yes	yes	yes	yes	Yes	no indirectness	
4004r	yes	yes	yes	yes	yes	Yes	no indirectness	
4004y	yes	yes	yes	yes	yes	Yes	no indirectness	
4004z	yes	yes	yes	yes	yes	Yes	no indirectness	
4065a	yes	yes	yes	yes	yes	Yes	no indirectness	

4067b	yes	yes	yes	yes	yes	Yes	no indirectness	
4123b	yes	yes	yes	yes	yes	Yes	no indirectness	
4123i	yes	yes	yes	yes	yes	Yes	no indirectness	
4206a	yes	yes	yes	yes	yes	Yes	no indirectness	
4206c	yes	yes	yse	yes	yes	Yes	no indirectness	
4260	yes	yes	yes	yes	yes	Yes	no indirectness	
4508a								
4615d	yes	yes	yes	yes	yes	Yes	no indirectness	
4615e	yes	yes	yes	yes	yes	Yes	no indirectness	
4629a	yes	yes	yes	yes	yes	Yes	no indirectness	
4812a	yes	yes	yes	yes	yes	Yes	no indirectness	
4838a	yes	yes	yes	yes	yes	Yes	no indirectness	
4838b	yes	yes	yes	yes	yes	Yes	no indirectness	
4844	yes	yes	yes	yes	yes	Yes	no indirectness	
5371	yes	yes	yes	yes	yes	Yes	no indirectness	
5533g	yes	yes	yes	yes	yes	Yes	no indirectness	
5606	yes	yes	yes	yes	yes	Yes	no indirectness	
6442a								
6564a	yes	yes	yes	yes	yes	Yes	no indirectness	
6568a	yes	yes	yes	yes	yes	Yes	no indirectness	
6670	yes	yes	yes	yes	yes	Yes	no indirectness	
6677	yes	yes	yes	yes	yes	Yes	no indirectness	
6746b	yes	yes	yes	yes	yes	Yes	no indirectness	
6797a	yes	yes	yes	yes	yes	Yes	no indirectness	
6797b	yes	yes	yes	yes	yes	Yes	no indirectness	
6797d	yes	yes	yes	yes	yes	Yes	no indirectness	
6806f	yes	yes	yes	yes	yes	Yes	no indirectness	
7029b	yes	yes	yes	yes	yes	Yes	no indirectness	
7484a	yes	yes	yes	yes	yes	Yes	no indirectness	
7504	yes	yes	yes	yes	yes	Yes	no indirectness	
8511a	yes	yes	yes	yes	yes	Yes	no indirectness	
8604b	yes	yes	yes	yes	yes	Yes	no indirectness	
8604f	yes	yes	yes	yes	yes	Yes	no indirectness	
8692e	yes	yes	yes	yes	yes	No	Serious indirectness	
8729a	yes	yes	yes	yes	yes	Yes	no indirectness	
8742a	yes	yes	yes	yes	yes	Yes	no indirectness	
8744d	yes	yes	yes	yes	yes	Yes	no indirectness	
8747a	yes	yes	yes	yes	yes	Yes	no indirectness	
8747b	yes	yes	yes	yes	yes	Yes	no indirectness	
8769b								
8770b	yes	yes	yes	yes	yes	Yes	no indirectness	
8784a	yes	yes	yes	yes	yes	Yes	no indirectness	
8790a	yes	yes	yes	yes	yes	Yes	no indirectness	
8815a	yes	yes	yes	yes	yes	Yes	no indirectness	

8817a	yes	yes	yes	yes	yes	Yes	no indirectness	
8817d	yes	yes	yes	yes	yes	Yes	no indirectness	
8817e	yes	yes	yes	yes	yes	Yes	no indirectness	
8826a	yes	yes	yes	yes	yes	Yes	no indirectness	
8829a	yes	yes	yes	yes	yes	Yes	no indirectness	
8850a								
8867								
8884a	yes	yes	yes	yes	yes	No	Serious indirectness	
8916a	yes	yes	yes	yes	yes	Yes	no indirectness	
8916b	yes	yes	yes	yes	yes	Yes	no indirectness	
8916c	yes	yes	yes	yes	yes	Yes	no indirectness	
8918a	yes	yes	yes	yes	yes	Yes	no indirectness	
8918n	yes	yes	yes	yes	yes	Yes	no indirectness	
8922l	yes	yes	yes	yes	yes	Yes	no indirectness	
8928	yes	yes	yes	yes	yes	Yes	no indirectness	
8931a	yes	yes	yes	yes	yes	Yes	no indirectness	
8933a	yes	yes	yes	yes	yes	Yes	no indirectness	

Table S9 cont. Imprecision: domain 4 for downgrading					
**Estimate ID**	**Does the 95% CI include the null effect and appreciable benefit/harm?**	**Does the 95% CI include a trivial effect and appreciable benefit/harm?**	**Meets the optimal information size**	**Decision:** *not serious, serious, very serious*	**Imprecision notes**
100c	no	no	yes	not serious	
593	no	no	yes	not serious	
1021	no	no	yes	not serious	
1164	no	yes	yes	serious	
1264	no	no	no	serious	
1438a	no	yes	yes	serious	
1606a	no	no	yes	not serious	
1629a	no	no	yes	not serious	
1653a	no	no	yes	not serious	
1752a	no	no	no	serious	
2159	no	no	yes	not serious	
2599a	no	no	yes	not serious	
2640b	no	no	yes	not serious	
2933a	no	no	yes	not serious	
3275d	no	no	yes	not serious	
3275e	no	yes	yes	serious	
3275l	no	no	yes	not serious	
3415	no	no	yes	not serious	
3639f	no	no	no	serious	
3640b	no	no	no	serious	
3640d	no	no	no	serious	
3657	no	no	yes	not serious	
3781d	no	no	yes	not serious	
4002h	no	no	yes	not serious	
4003a					
4003e	no	yes	yes	serious	
4004o	no	no	no	serious	
4004r	no	no	no	serious	
4004y	no	no	no	serious	
4004z	no	no	yes	not serious	
4065a	no	no	yes	not serious	
4067b	no	yes	no	serious	
4123b	no	no	yes	not serious	
4123i	no	no	yes	not serious	
4206a	no	no	yes	not serious	
4206c	no	no	yes	not serious	
4260	no	no	no	serious	
4508a					
4615d	no	no	yes	not serious	

4615e	no	no	no	serious	
4629a	no	no	yes	not serious	
4812a	no	no	yes	not serious	
4838a	no	no	no	serious	
4838b	no	no	yes	not serious	
4844	no	no	yes	not serious	
5371	no	no	no	serious	
5533g	no	no	no	serious	
5606	no	no	yes	not serious	
6442a					
6564a	no	yes	no	serious	
6568a	no	no	yes	not serious	
6670	no	no	yes	not serious	
6677	no	no	no	not serious	
6746b	no	no	yes	not serious	
6797a	no	no	no	serious	
6797b	no	no	yes	not serious	
6797d	no	no	no	serious	
6806f	no	no	yes	not serious	
7029b	no	no	yes	not serious	
7484a	no	no	yes	not serious	
7504	no	no	no	serious	
8511a	no	yes	no	serious	
8604b	no	yes	yes	serious	
8604f	no	no	yes	no serious	
8692e	no	yes	no	serious	
8729a	no	no	yes	not serious	
8742a	no	yes	no	serious	
8744d	no	yes	no	serious	
8747a	no	no	no	serious	
8747b	no	no	no	serious	
8769b					
8770b	no	no	no	serious	
8784a	no	yes	yes	serious	
8790a	no	no	no	serious	
8815a	no	no	no	serious	
8817a	no	yes	yes	not serious	
8817d	no	no	yes	not serious	
8817e	no	no	yes	not serious	
8826a	no	no	yes	not serious	
8829a	no	no	yes	not serious	
8850a					
8867					
8884a	no	no	yes	not serious	
8916a	no	no	yes	not serious	

8916b	no	no	yes	not serious	
8916c	no	no	yes	not serious	
8918a	no	no	no	serious	
8918n	no	no	no	serious	
8922l	no	yes	no	serious	
8928	no	no	no	serious	
8931a	no	yes	yes	serious	
8933a	no	no	no	serious	

Table S9 cont. Publication bias: domain 5 for downgrading									
**Estimate ID**	**High number of small studies**	**Percentage of included trials with conflicts of interest**	**Funnel plot reported**	**Visual evidence of publication bias**	**Statistical evidence of publication bias**	**RCTs of new therapy and unrobust search**	**The review searched for and present findings from unpublished studies that showed different results**	**Decision:** *undetected, strongly suspected, very strongly suspected*	**Publication bias notes**
100c	no	unclear	no	NR	NR	no	no	undetected	
593	yes	100%	no	NR	NR	no	no	strongly suspected	
1021	yes	unclear	no	no	NR	no	no	undetected	
1164	yes	unclear	no	NR	NR	no	no	undetected	
1264	yes	unclear	no	NR	NR	no	no	undetected	
1438a	no	100%	no	NR	NR	no	no	undetected	
1606a	no	unclear	no (only two studies so formal analysis not possible)	NR	NR	no	no	undetected	
1629a	no	unclear	no	NR	NR	no	no	undetected	
1653a	unclear	unclear	no	NR	NR	no	no (included some unpublished data however, it is unclear if more unpublished data were witheld by participating drug companies)	undetected	
1752a	no	unclear	yes	no	no	no	no	undetected	
2159	yes	unclear	no	NR	NR	no	no	undetected	
2599a	yes	unclear	yes	no	NR	no	no	undetected	asymetrical appearance to the funnel plot but only 4 studies included so difficult to make a judgement
2640b	no	100%	no	NR	NR	no	no	strongly suspected	FDA trial reports utilised
2933a	no	unclear	no	NR	NR	no	no	undetected	
3275d	no	100%	no	NR	NR	no	no	strongly suspected	
3275e	no	100%	no	NR	NR	no	no	undetected	
3275l	no	100%	no	NR	NR	no	no	undetected	
3415	no	unclear	no	NR	NR	no	no	undetected	
3639f	no	100%	no	NR	NR	no	no	undetected	
3640b	no	100%	no	NR	NR	no	no	strongly suspected	In addition to all trials being industry funded, the review authors had potential conflicts of interest with companies that funded some of the studies.
3640d	no	100%	no	NR	NR	no	no	strongly suspected	In addition to all trials being industry funded, the review authors had potential conflicts of interest with companies that funded some of the studies.
3657	no	0%	no	NR	NR	no	no	undetected	
3781d	no	unclear	no	no	no	no	no	undetected	
4002h	yes	0	no	NR	NR	no	no	undetected	
4003a									
4003e	no	100%	no	NR	NR	no	no	undetected	
4004o	no	100%	no	NR	NR	no	no	undetected	

4004r	no	100%	no	NR	NR	no	no	undetected	
4004y	no	80%	no	NR	NR	no	no	undetected	Outcome under-reported across studies of Budesonide vs. control.
4004z	no	100%	no	NR	NR	no	no	undetected	
4065a	yes	unclear	no	NR	NR	no	no	undetected	
4067b	yes	unclear	no	NR	NR	no	no	undetected	
4123b	no	0	no	NR	NR	no	no	undetected	
4123i	no	0	no	NR	NR	no	no	undetected	
4206a	no		no	NR	NR	no	no	undetected	
4206c	no		no	NR	NR	no	no	undetected	
4260	no	0	no	NR	NR	no	no	undetected	
4508a									
4615d	yes	unclear	no	NR	no	no	no	undetected	
4615e	yes	unclear	no	NR	no	no	no	undetected	
4629a	no	unclear	yes	no	no	no	no	undetected	
4812a	yes	unclear	no	NR	NR	no	no	undetected	
4838a	no	unclear	no	no	no	no	no	undetected	
4838b	no	unclear	no	no	no	no	no	undetected	
4844	yes	unclear	no	NR	NR	no	no	undetected	
5371	yes	unclear	no	NR	NR	no	no	undetected	
5533g	no	100%	no	NR	NR	no	no	undetected	
5606	yes	44%	no	NR	NR	no	no	strongly suspected	
6442a									
6564a	yes	unclear	no	NR	no	no	no	undetected	
6568a	yes	unclear	no	NR	no	no	no	undetected	
6670	no	unclear	no	NR	NR	no	no	undetected	
6677	yes	unclear	no	NR	NR	no	no	strongly suspected	
6746b	no	unclear	no	NR	NR	no	no	undetected	
6797a	no	unclear	no	NR	NR	no	no	undetected	
6797b	no	unclear	no	NR	NR	no	no	undetected	
6797d	no	unclear	no	NR	NR	no	no	undetected	
6806f	no	unclear	no	no (but there was a funnel plot with evidence of publication bias for relapse)	no	no	no	undetected	
7029b	yes	unclear	yes	no	NR	no	no	undetected	
7484a	yes	unclear	no	NR	NR	no	no	undetected	
7504	no	unclear	no	NR	NR	no	no	undetected	
8511a	no	unclear	no	NR	NR	no	no	undetected	
8604b	yes	unclear	no	NR	NR	no	no	undetected	
8604f	yes	unclear	no	NR	NR	no	no	undetected	
8692e	no	100%	no	NR	NR	no	no	undetected	
8729a	yes	unclear	no	NR	no	no	no	undetected	
8742a	no	unclear	yes	no	no	no	no	undetected	
8744d	no	100%	no	NR	NR	no	no	undetected	
8747a	no	unclear	no	NR	NR	no	no	undetected	

8747b	no	unclear	no	NR	NR	no	no	undetected	
8769b									
8770b	yes	14%	no	NR	NR	no	no	undetected	
8784a	yes	0	no	NR	NR	no	no	undetected	
8790a	no	unclear	no	NR	NR	no	no	undetected	
8815a	yes	unclear	no	NR	NR	no	no	undetected	
8817a	no	unclear	yes	yes	yes	no	no	strongly suspected	
8817d	no	unclear	yes	yes	yes	no	no	strongly suspected	
8817e	no	unclear	yes	yes	yes	no	no	strongly suspected	
8826a	yes	unclear	no	NR	NR	no	no	undetected	
8829a	no	unclear	no	NR	NR	no	no	undetected	
8850a									
8867									
8884a	yes	unclear	no	NR	NR	no	no	undetected	
8916a	no	unclear	no	NR	NR	no	no	undetected	
8916b	no	unclear	no	NR	NR	no	no	undetected	
8916c	no	unclear	no	NR	NR	no	no	undetected	
8918a	yes	unclear	no	NR	NR	no	no	undetected	
8918n	yes	unclear	no	NR	NR	no	no	undetected	
8922l	yes	unclear	no	NR	NR	no	no	undetected	
8928	yes	unclear	no	NR	NR	no	no	strongly suspected	
8931a	no	unclear	no	NR	no	no	no	undetected	
8933a	no	unclear	no	NR	NR	no	no	undetected	

Table S10. GRADED estimates showing an increase in admissions

Author, Year, Estimate ID	Population description	Intervention (Event rate)[a]	Comparison (Event rate)[a]	Patients (RCTs)	Patient age[b]	Outcome (follow-up)[c]	Effect (95% CI)[d,e] NNT (95% CI)[d,f] I²%	Quality of evidence (GRADE)	Notes
**Disease of the circulatory system**									
**Atrial fibrillation**									
Cordina, 2005, 1606a	Paroxysmal, sustained, or permanent atrial fibrillation or atrial flutter	Rhythm medications (antiarrhythmic drugs) (49 per 100)	Rate control medications (rate control drugs) (42 per 100)	4312 (2)	65 ±6	Hospital admission (27 ±21 months)	RR: 1.16 (1.11 to 1.22) NNT: 15 (11 to 22) I²: 97%	Low	Summary estimate
**Heart Failure**									
Heran, 2012, 3275e	Heart failure, any ejection fraction	Angiotensin II receptor blocker (39 per 100)	Placebo (37 per 100)	9449 (4)	68 ±3	Hospital admission (31 ±20 months)	RR: 1.06 (1.01 to 1.12) NNT: 45 (23 to 272) I²: 0%	Very low	Summary estimate
**Diseases of the respiratory system**									
**Other chronic obstructive pulmonary disease**									
Kew, 2014, 4004o	Moderate to severe chronic obstructive pulmonary disease	Budesonide (4.5 per 100)	Placebo (1.3 per 100)	867 (3)	63 ±0.2	Admission for pneumonia (Range 6 to 48 months)	OR: 3.47 (1.11 to 10.83) NNT: 31 (9 to 685) I²: 0%	Low	Sub-group by comparison: Placebo
Kew, 2014, 4004r	Moderate to severe chronic obstructive pulmonary disease	Budesonide (with or without formoterol) (1.4 per 100)	Controls (placebo or formoterol) (0.7 per 100)	4659 (6)	64 ±1	Admission for pneumonia (8 ±4 months)	OR: 2.02 (1.15 to 3.57) NNT: 141 (57 to 954) I²: 4%	Low	Sub-group by intervention: Budesonide 640 mcg (320 mcg bid)
Kew, 2014, 4004y	Moderate to severe chronic obstructive pulmonary disease	Budesonide (with or without formoterol) (1 per 100)	Controls (placebo or formoterol) (0.3 per 100)	3515 (4)	65 ±2	Admission for pneumonia (7 ±3 months)	OR: 3.28 (1.22 to 8.81) NNT: 133 (40 to 1369) I²: 0%	Low	Sub-group by study quality: Trials at high risk of bias removed
Kew, 2014, 4004z	Moderate to severe chronic obstructive pulmonary disease	Fluticasone (with or without long acting beta2-agonist) (1.4 per 100)	Controls (placebo or long acting beta2-agonist) (0.8 per 100)	16338 (15)	64 ±1	Admission for pneumonia (12 ±11 months)	OR: 1.82 (1.52 to 2.19) NNT: 164 (114 to 259) I²: 0%	High	Sub-group by study quality: Trials at high risk of bias removed
**Factors influencing health status and contact with health services**									
**Patients presenting to primary care**									
Laurant, 2005, 9181a	Patients presenting to primary care	Nurse-led primary care (12 per 100)	Physician-led primary care (11 per 100)	15860 (3)	45	Hospital admission (6 ±4 months)	RR: 1.17 (1.04 to 1.31) NNT: 56 (31 to 238) I²: 40%	Moderate	Summary estimate
**Special screening examination for neoplasms**									

Yang, 2015, 8918n	Patients undergoing prostate biopsy	Single drug (3 per 100)	Combined drugs (0.5 per 100)	659 (3)	Unclear	Hospital admission (0.55 ± 0.66 months)	RR: 5.91 (2.2 to 15.87) NNT: 38 (13 to 157) I²: 0%	Low	Summary estimate
**Mental and behavioural disorders**									
**Schizophrenia and other types of schizophrenia-like psychoses (Acute and transient psychotic disorders; Bipolar affective disorder; Induced delusional disorder; Other nonorganic psychotic disorders; Persistent delusional disorders; Schizoaffective disorders; Schizophrenia; Schizotypal disorder; Unspecified nonorganic psychosis)**									
Marshall, 1998, 4966a	Schizophrenia and schizophrenia-like disorders, bipolar disorder or depression with psychotic features	Case management (34 per 100)	Standard care (22 per 100)	1300 (6)	45 ±5	Hospital admission (13 ±5 months)	OR: 1.84 (1.43 to 2.37) NNT: 8 (6 to 15) I²: 61%	Low	Summary estimate
Sampson, 2013, 6806f	Schizophrenia and other types of schizophrenia-like psychoses	Intermittent drug technique (specific drug) (38 per 100)	Maintenance therapy (24 per 100)	661 (6)	35 ±5	Hospital admission (21 ±7 months)	RR: 1.58 (1.28 to 1.97) NNT: 7 (4 to 15) I²: 19%	Low	Summary estimate
**Severe mental illnesses and disordered personality**									
Buckley, 2015, 8733c	Schizophrenia and other types of schizophrenia-like psychoses	Supportive therapy: interventions provided by a single person with the main purpose of maintaining current functioning or assisting pre-existing coping abilities. A number of common therapies were excluded as they are designed to teach new skills or change pre-existing skills: cognitive behavioural therapy, social skills training, psycho-education, compliance therapy and problem-solving therapy. (21 per 100)	Any other psychological or psychosocial treatment (12 per 100)	306 (4)	Range 18 to 65	Hospital admission (34 ±50 months)	RR: 1.82 (1.11 to 2.99) NNT: 11 (79 to 4) I²: 13%	Very low	Summary estimate
**Mixed population**									
Baigent, 2013, 1653a	Patients indicated for treatment with non-steroidal anti-inflammatory drugs: Rheumatoid arthritis; Osteoarthritis; Colorectal adenomata; Alzheimer's disease	Cox-2-inhibitors (59 per 100)	Placebo (26 per 100)	88367 (184)	Unclear	Admission for heart failure (Unclear)	rR: 2.28 (1.62 to 3.2) NNT: 3 (2 to 6) I²: Unclear	Moderate	Summary estimate
**Neoplasms**									
Gupta, 2014, 8688a	Symptomatic uterine fibroids	Uterine artery embolization (27 per 100)	Myomectomy (13 per 100)	278 (2)	39 ±9	Readmission (9 ±4 months)	RD: 0.14 (0.05 to 0.22) NNT: 7 (5 to 20) I²: 86%	Low	Summary estimate

[a]Median hospitalisation event rate observed across the trials contributing to the pooled estimate. [b]Means and standard deviations for patient ages in years, unless otherwise specified. Mean of means and median ages in trials contributing to the pooled estimate. Not all included trials reported age in a form that could be averaged. [c]Means and standard deviations for length of follow-up across trials, unless otherwise specified. Mean of means, medians, and total study durations reported in trials contributing to the pooled estimate. Not all included trials reported follow-up in a form that could be averaged. [d]RR= risk ratio; OR=odds ratio. [e]CI=confidence interval. [f]NNT= Number needed to treat to benefit by avoiding one hospital admission. [g]As indicated in Table 2 : the symbol (+) denotes when review conclusions indicated an favourable overall balance of benefit to harm or the authors recommend use of intervention; the symbol (-) denotes when review conclusions indicated that there was not a favourable overall balance of benefit to harm or the authors recommended not to use the intervention; and the symbol (?) denotes when review conclusions indicated that the overall balance of benefit to harm was unclear, the authors did not comment on the overall balance of benefit to harm, or the authors indicated that more research was required before a recommendation regarding use of the intervention could be made.
